# Supplementary material for: Metagenome-based metabolic modelling predicts unique microbial interactions in deep-sea hydrothermal plume microbiomes
Source: ISME Commun. 2023 Apr 29;3:42. doi: 10.1038/s43705-023-00242-8 (PMC10148797; doi:10.1038/s43705-023-00242-8)
Supplement: Supplementary file 1 — Supplementary Tables 1-3 [file 43705_2023_242_MOESM1_ESM.pdf]

Metagenome-based metabolic modelling predicts unique microbial interactions in deep-sea hydrothermal plume microbiomes - Supplementary Tables

Supplementary Table Legends

Supplementary Table 1	List of archaea present in the Guaymas microbiome
Supplementary Table 2	List of unique contributing microbial classes in Guaymas microbiome across media
Supplementary Table 3	List of unique contributors in Guaymas microbiome across media

Supplementary Table 1. List of archaea present in the Guaymas microbiome.

S. No	Archaea
1	Candidatus Nitrosopelagicus sp UWMA 0359 (CNP359)
2	Candidatus Pacearchaeota archaeon UWMA 0287 (CPA287)
3	Marine Group II euryarchaeote UWMA 0266 (MGII266)
4	Marine Group II euryarchaeote UWMA 0275 (MGII275)
5	Marine Group II euryarchaeote UWMA 0279 (MGII279)
6	Marine Group II euryarchaeote UWMA 0283 (MGII283)
7	Marine Group II euryarchaeote UWMA 0323 (MGII323)
8	Marine Group II euryarchaeote UWMA 0328 (MGII328)
9	Marine Group II euryarchaeote UWMA 0344 (MGII344)
10	Marine Group II euryarchaeote UWMA 0350 (MGII350)
11	Marine Group II euryarchaeote UWMA 0352 (MGII352)
12	Marine Group II euryarchaeote UWMA 0357 (MGII357)
13	Marine Group III euryarchaeote UWMA 0284 (MGIII284)
14	Marine Group III euryarchaeote UWMA 0340 (MGIII340)
15	Nitrosopumilus sp UWMA 0263 (NPUM263)

Supplementary Table 2. List of unique contributing microbial classes in Guaymas microbiome across media.

S. No	allmedia	GM media	JW1 media	Marine broth 2216
1	Alphaproteobacteria		Alphaproteobacteria	
2			Dehalococcoidia	
3	Gammaproteobacteria		Gammaproteobacteria	Gammaproteobacteria
4				Nitrososphaeria
5		Planctomycetes		
6	Poseidoniia	Poseidoniia	Poseidoniia	Poseidoniia
7				Rhodothermia
8	UBA8108		UBA8108	

Supplementary Table 3. List of unique contributors in Guaymas microbiome across media.

S.No	allmedia	GM media	JW1 media	Marine broth 2216
1	Planctomycetes bacterium UWMA 0276		Planctomycetes bacterium UWMA 0276	
2	Porticoccaceae bacterium UWMA 0313		Porticoccaceae bacterium UWMA 0313	
3	Sulfitobacter sp UWMA 0305		Sulfitobacter sp UWMA 0305	
4	Methylococcaceae bacterium UWMA 0325		Methylococcaceae bacterium UWMA 0325	
5	Marine Group II euryarchaeote UWMA 0352		Marine Group II euryarchaeote UWMA 0352	Marine Group II euryarchaeote UWMA 0352
6	Marine Group III euryarchaeote UWMA 0284	Marine Group III euryarchaeote UWMA 0284	Marine Group III euryarchaeote UWMA 0284	
7			Dehalococcoidia bacterium UWMA 0267	
8				Nitrosopumilus sp UWMA 0263
9				Bacteroidetes bacterium UWMA 0293
10				Gammaproteobacteria bacterium UWMA 0299
