# Supplementary material for: Metagenome-based metabolic modelling predicts unique microbial interactions in deep-sea hydrothermal plume microbiomes
Source: ISME Commun. 2023 Apr 29;3:42. doi: 10.1038/s43705-023-00242-8 (PMC10148797; doi:10.1038/s43705-023-00242-8)
Supplement: Supplementary file 5 — Supplementary File S4 [file 43705_2023_242_MOESM5_ESM.zip › Supplementary File S4 Pairwise interactions/Pairwise interactions/Supplementary Figure S0 legend.pdf]

Legend for pairwise interaction plots

- Microbe under study
- Class Acidimicrobiia
- Class Actinobacteria
- Class Alphaproteobacteria
- Class Bacteroidia
- Class Binatia
- Class Dehalococcoidia
- Class Gammaproteobacteria
- Class Gemmatimondates
- Class Marinisomatia
- Class Nanoarchaeia
- Class Nitrososphaeria
- Class Nitrospinia
- Class Nitrospiria
- Class Planctomycetes
- Class Poseidoniia
- Class Rhodothermia
- Class SAR324
- Class UBA1135
- Class UBA2968
- Class UBA8108
- Class UBA9160
- Class unclassified candidate division Zixibacteria
- Class Verrucomicrobiae
- Class Vicinamibacteria
