# Supplementary figures and images for: Metagenome-based metabolic modelling predicts unique microbial interactions in deep-sea hydrothermal plume microbiomes (part 1 of 2)
Source: ISME Commun. 2023 Apr 29;3:42. doi: 10.1038/s43705-023-00242-8 (PMC10148797; doi:10.1038/s43705-023-00242-8)

# Acidimicrobiia\_bacterium\_UWMA\_0264\_accepting\_metabolites

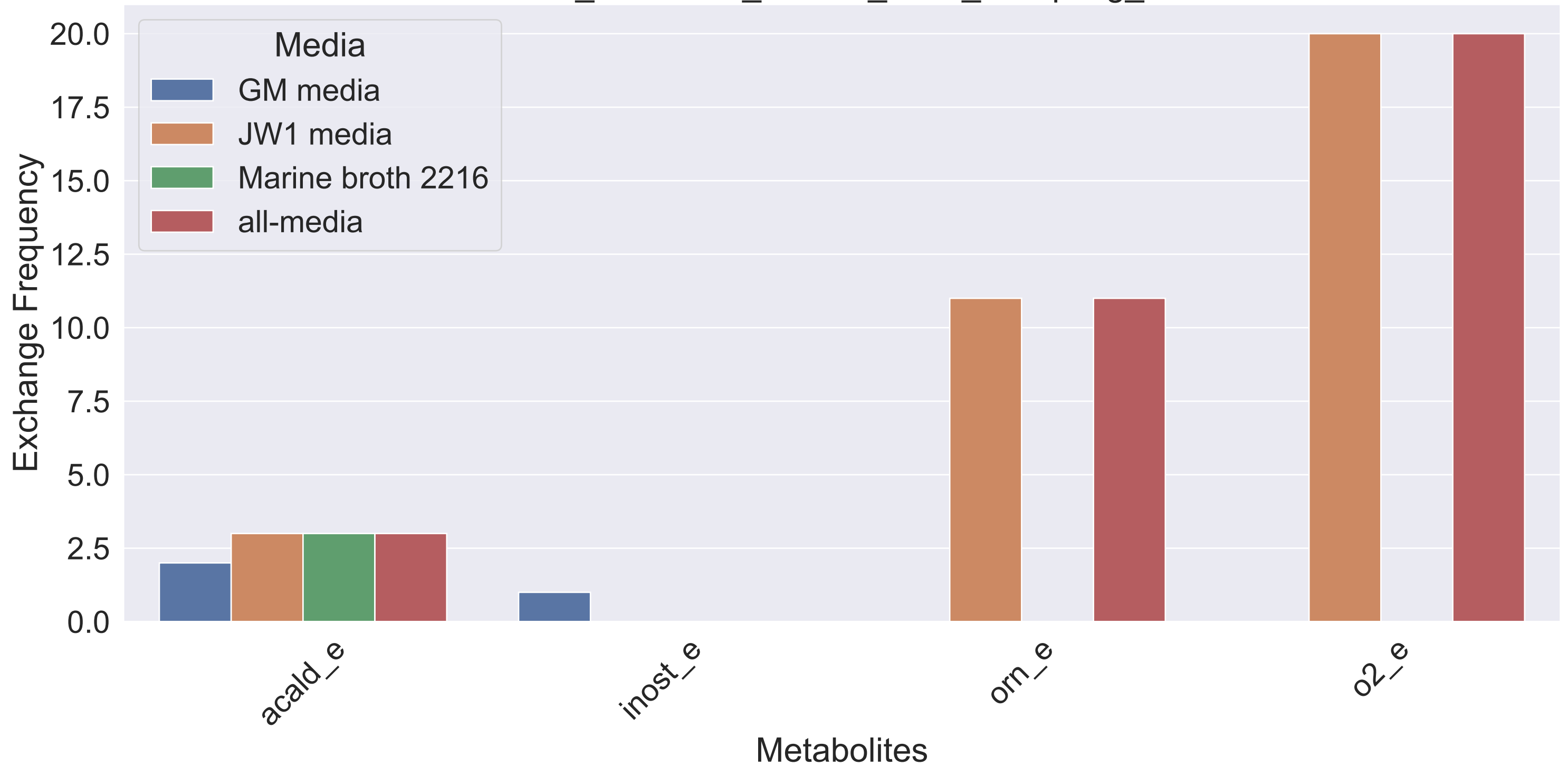

Supplement: Supplementary file 6 — Supplementary File S5 [file 43705_2023_242_MOESM6_ESM.zip › Supplementary Figure S1 Acidimicrobiia bacterium UWMA 0264 accepting metabolites.pdf]

Candidatus\_Handelsmanbacteria\_bacterium\_UWMA\_0286\_accepting\_metabolites

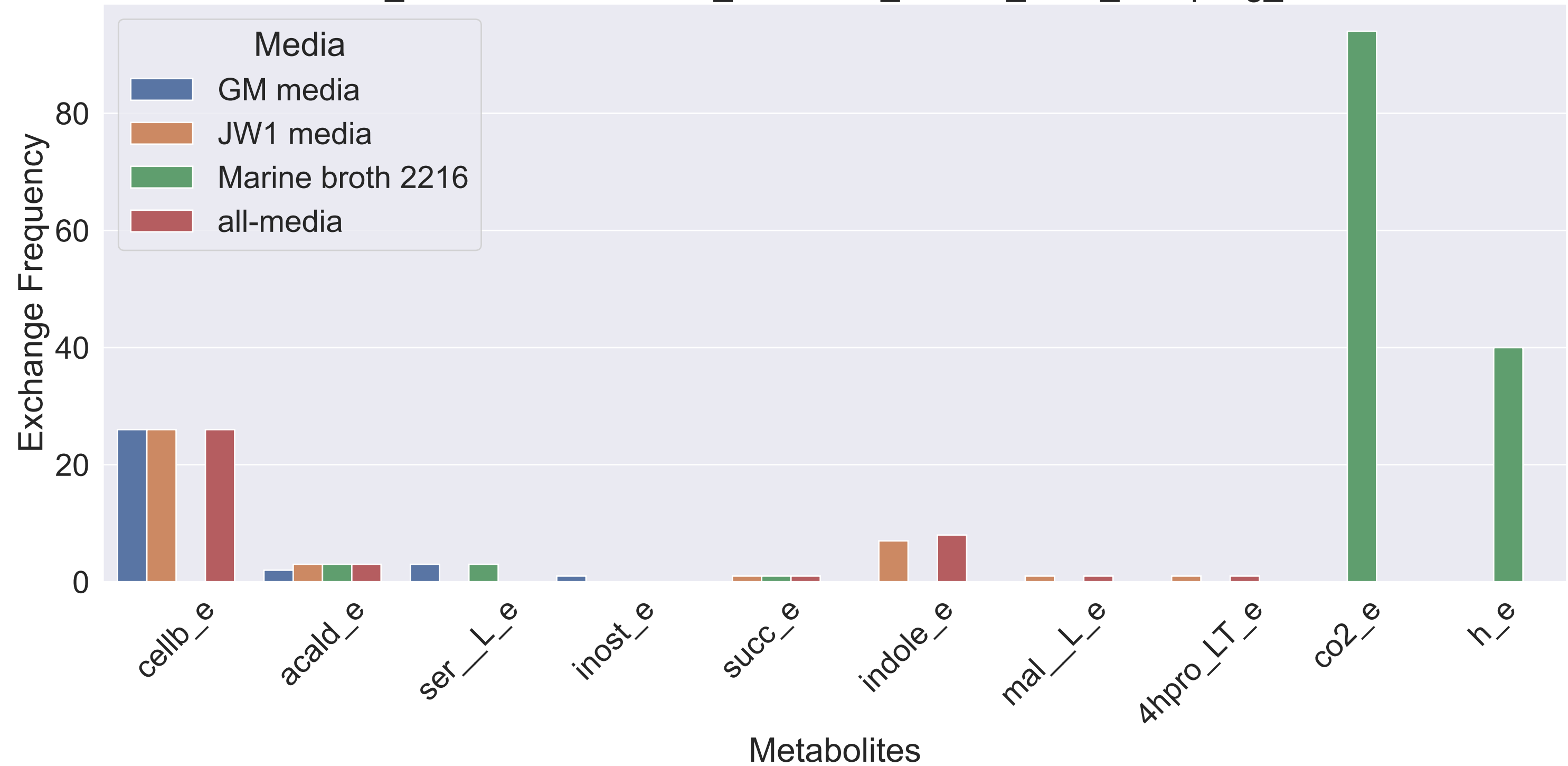

Supplement: Supplementary file 6 — Supplementary File S5 [file 43705_2023_242_MOESM6_ESM.zip › Supplementary Figure S10 Candidatus Handelsmanbacteria bacterium UWMA 0286 accepting metabolites.pdf]

Candidatus\_Handelsmanbacteria\_bacterium\_UWMA\_0300\_accepting\_metabolites

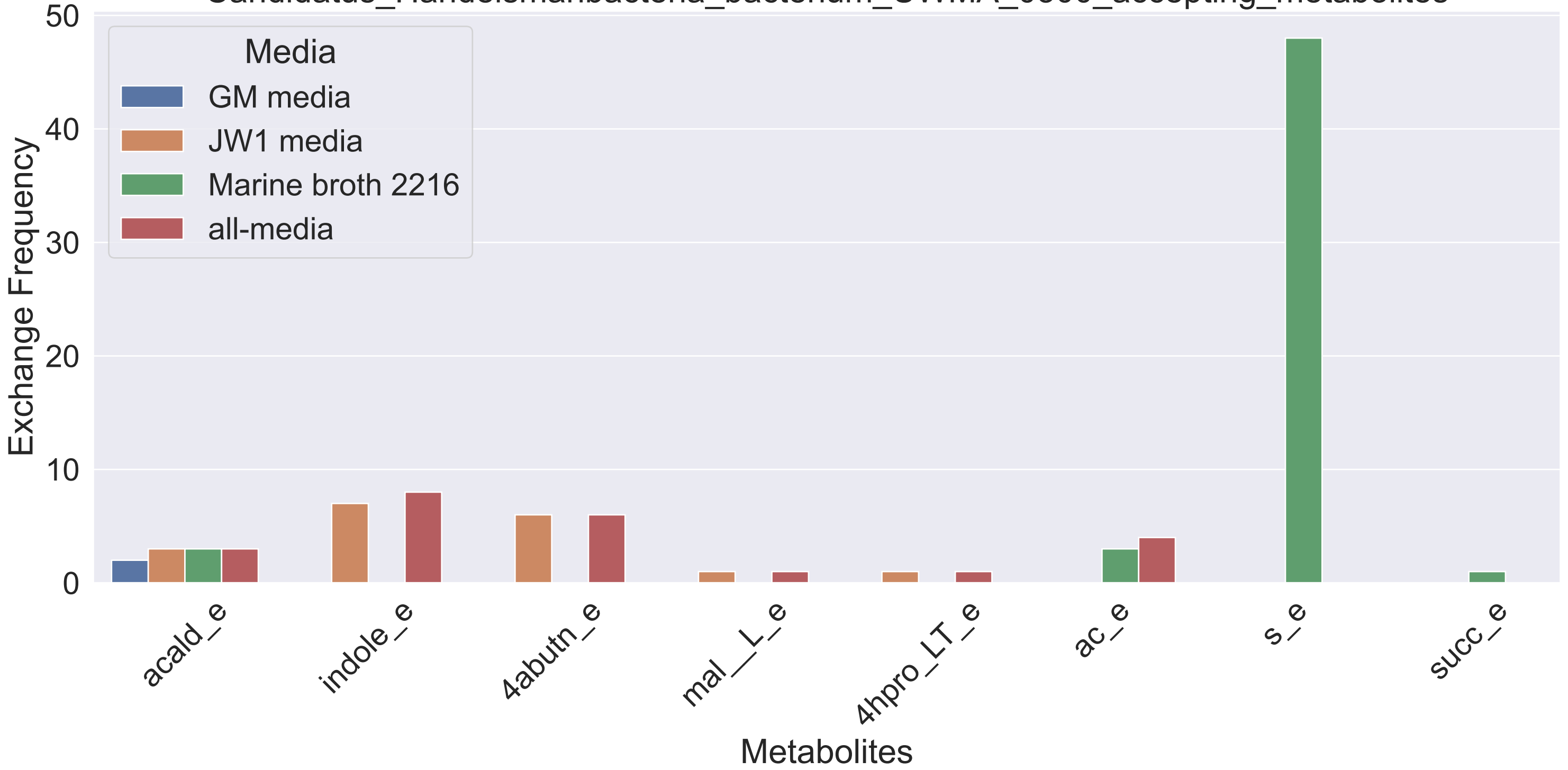

Supplement: Supplementary file 6 — Supplementary File S5 [file 43705_2023_242_MOESM6_ESM.zip › Supplementary Figure S11 Candidatus Handelsmanbacteria bacterium UWMA 0300 accepting metabolites.pdf]

Candidatus\_Lambdaproteobacteria\_bacterium\_UWMA\_0298\_accepting\_metabolites

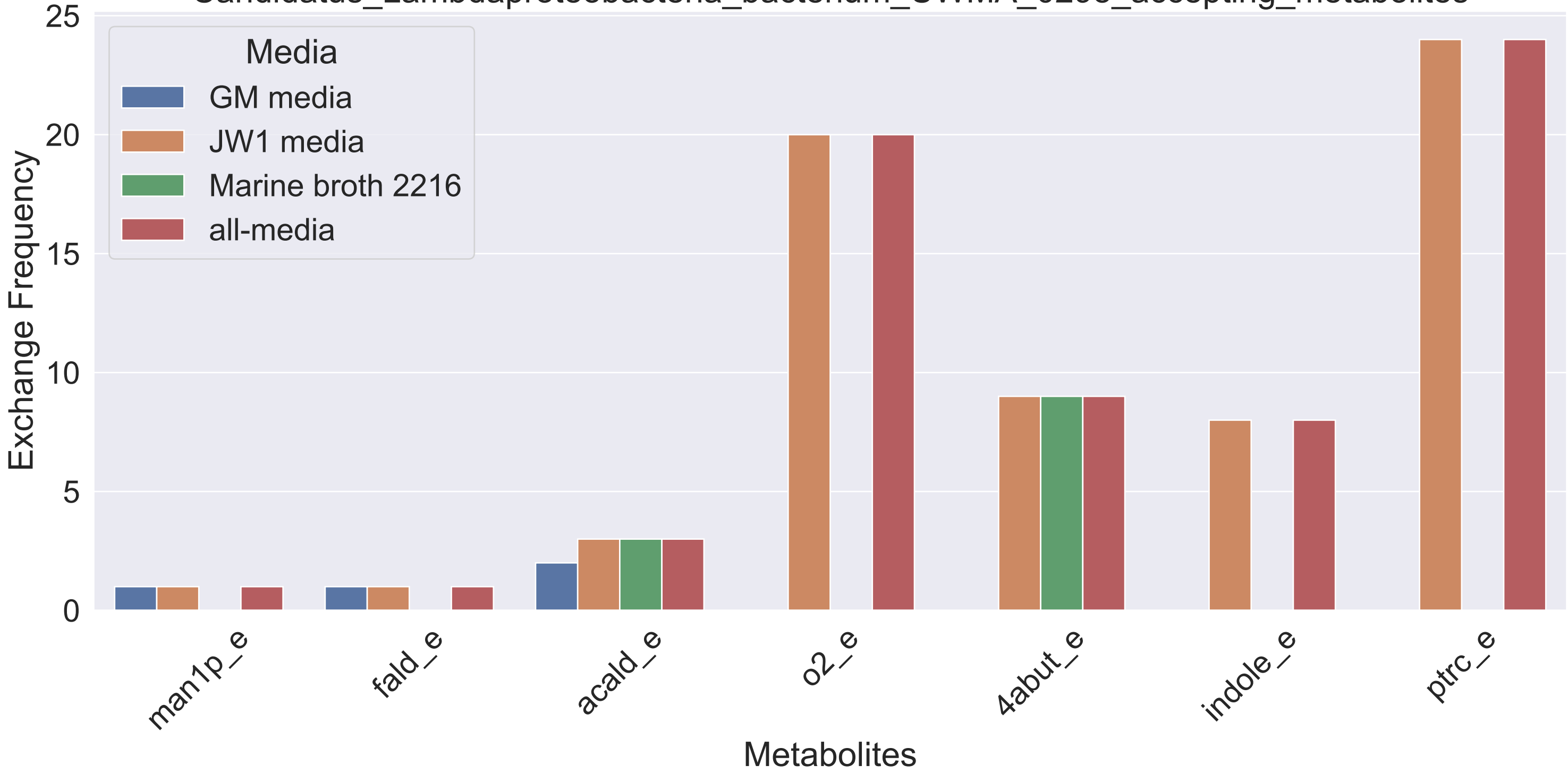

Supplement: Supplementary file 6 — Supplementary File S5 [file 43705_2023_242_MOESM6_ESM.zip › Supplementary Figure S12 Candidatus Lambdaproteobacteria bacterium UWMA 0298 accepting metabolites.pdf]

# Candidatus\_Lambdaproteobacteria\_bacterium\_UWMA\_0318\_accepting\_metabolites

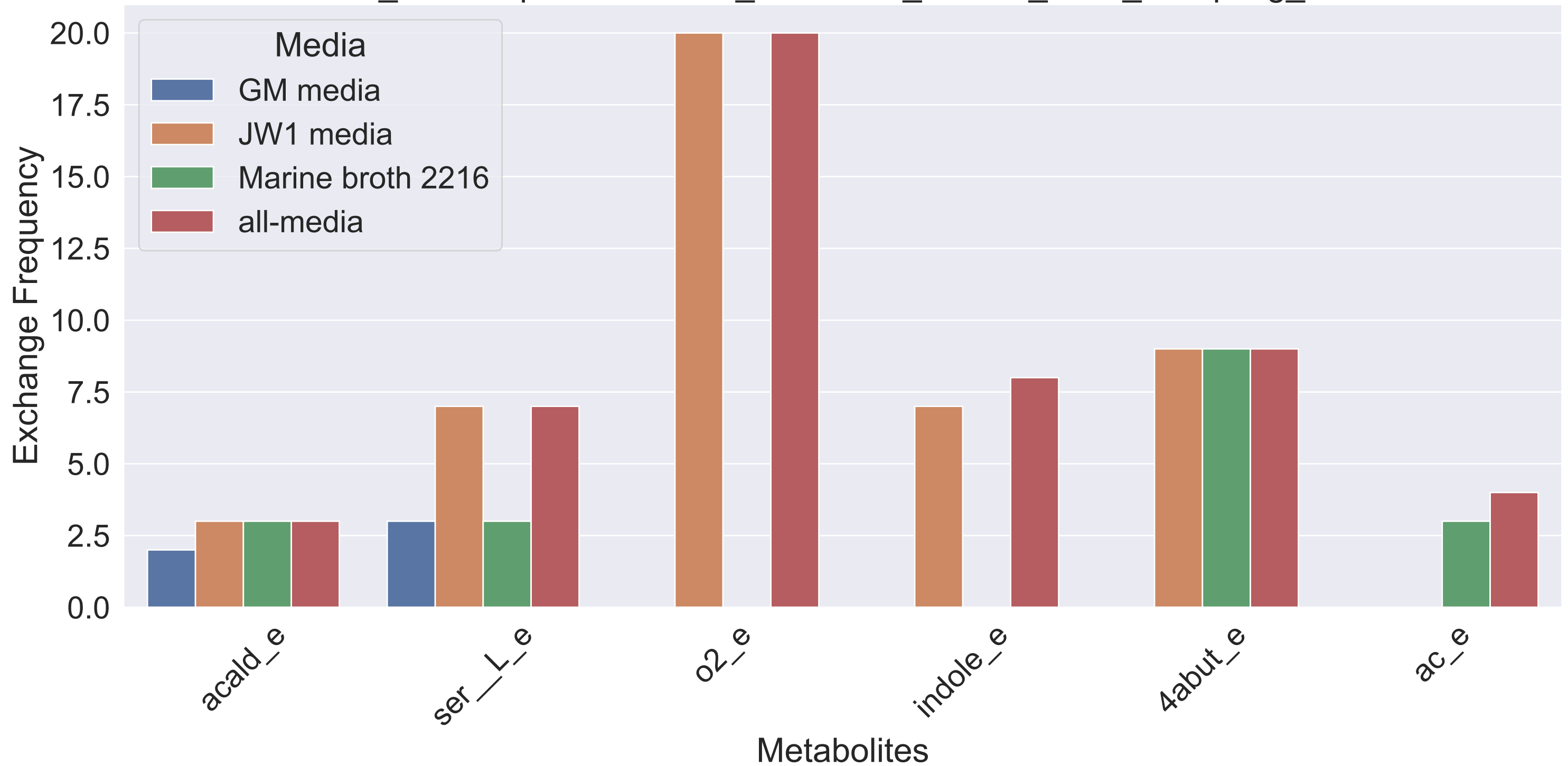

Supplement: Supplementary file 6 — Supplementary File S5 [file 43705_2023_242_MOESM6_ESM.zip › Supplementary Figure S13 Candidatus Lambdaproteobacteria bacterium UWMA 0318 accepting metabolites.pdf]

# Candidatus\_Marinimicrobia\_bacterium\_UWMA\_0285\_accepting\_metabolites

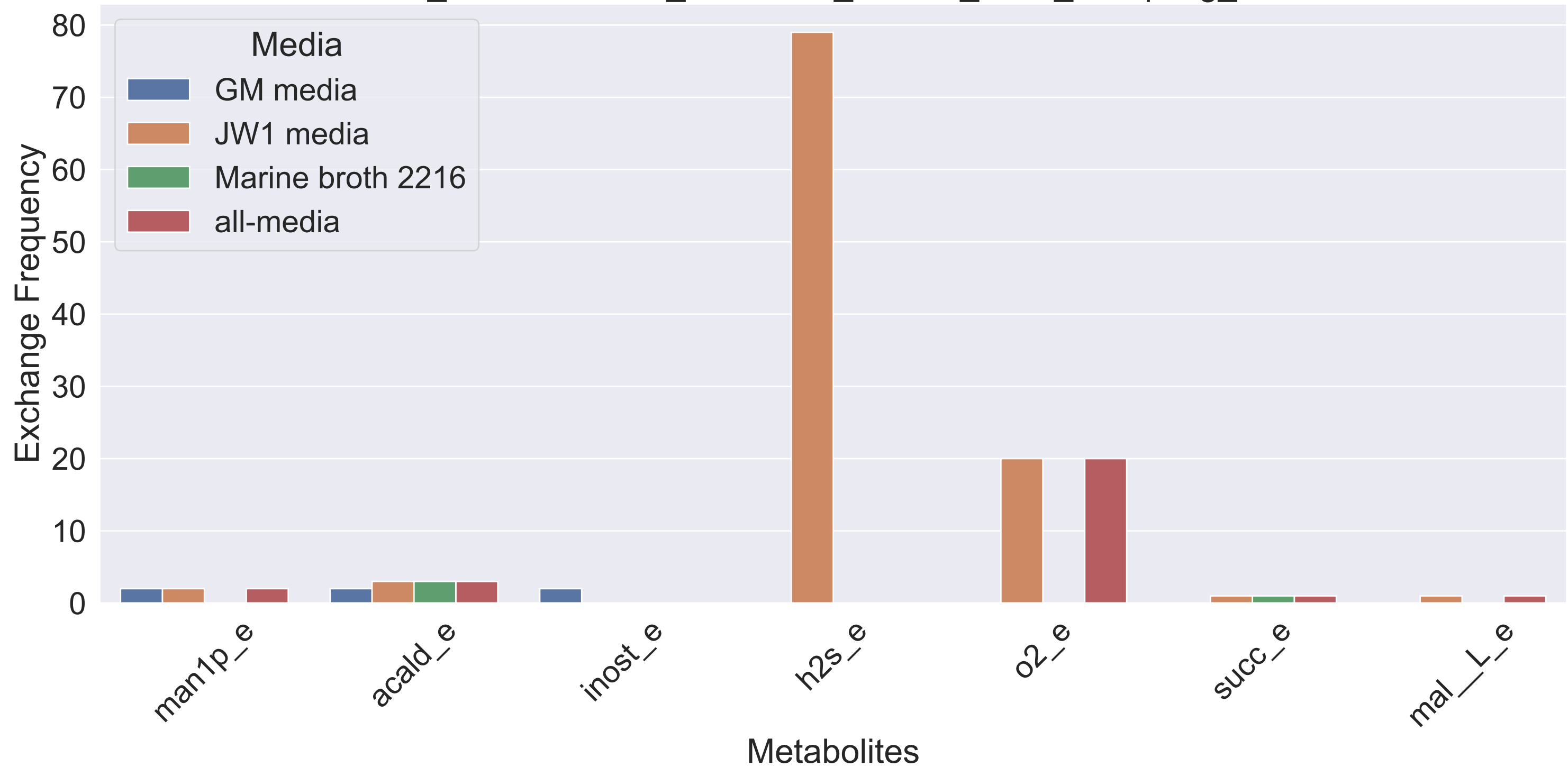

Supplement: Supplementary file 6 — Supplementary File S5 [file 43705_2023_242_MOESM6_ESM.zip › Supplementary Figure S14 Candidatus Marinimicrobia bacterium UWMA 0285 accepting metabolites.pdf]

Candidatus\_Marinimicrobia\_bacterium\_UWMA\_0306\_accepting\_metabolites

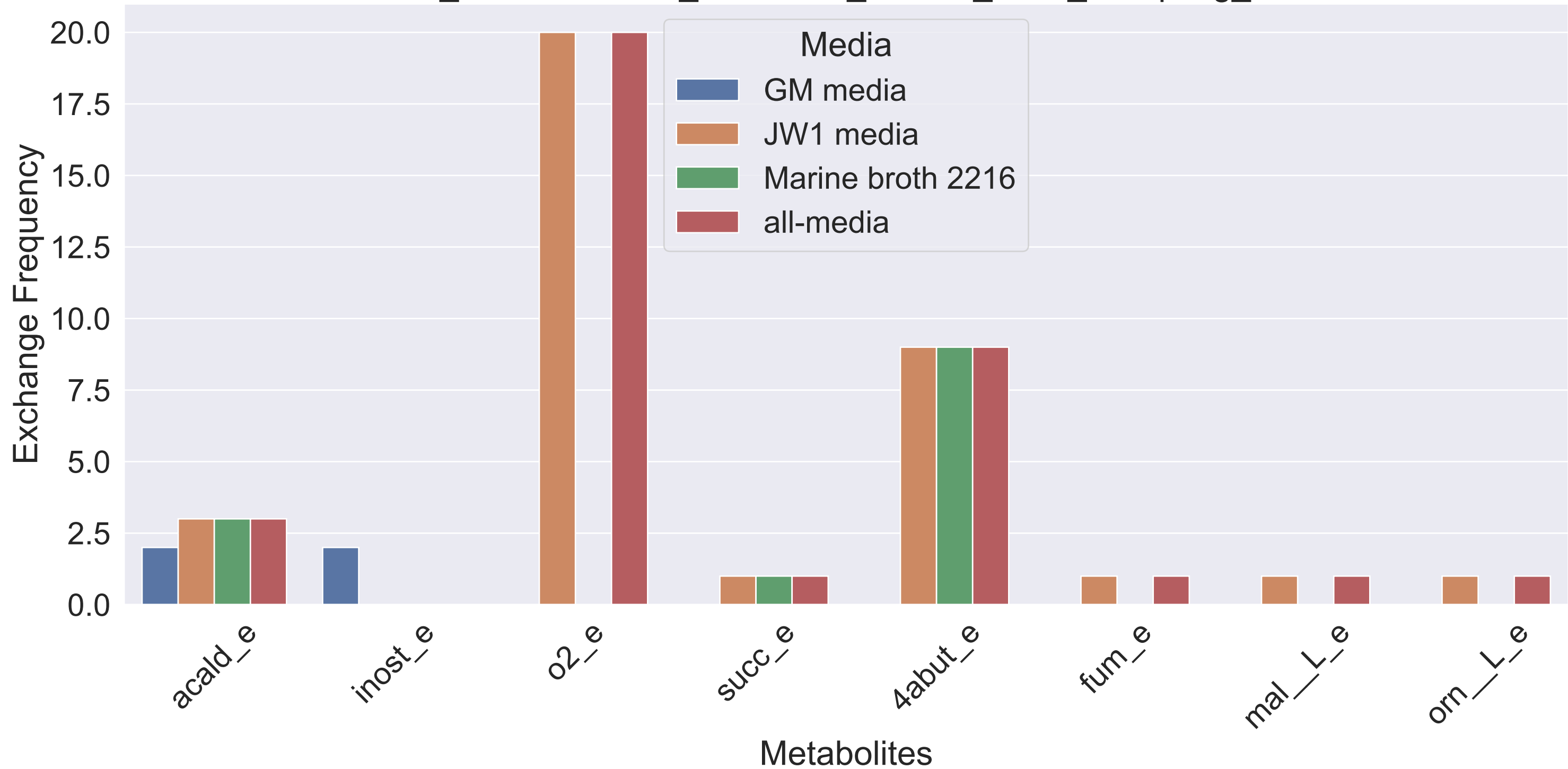

Supplement: Supplementary file 6 — Supplementary File S5 [file 43705_2023_242_MOESM6_ESM.zip › Supplementary Figure S15 Candidatus Marinimicrobia bacterium UWMA 0306 accepting metabolites.pdf]

# Candidatus\_Marinimicrobia\_bacterium\_UWMA\_0309\_accepting\_metabolites

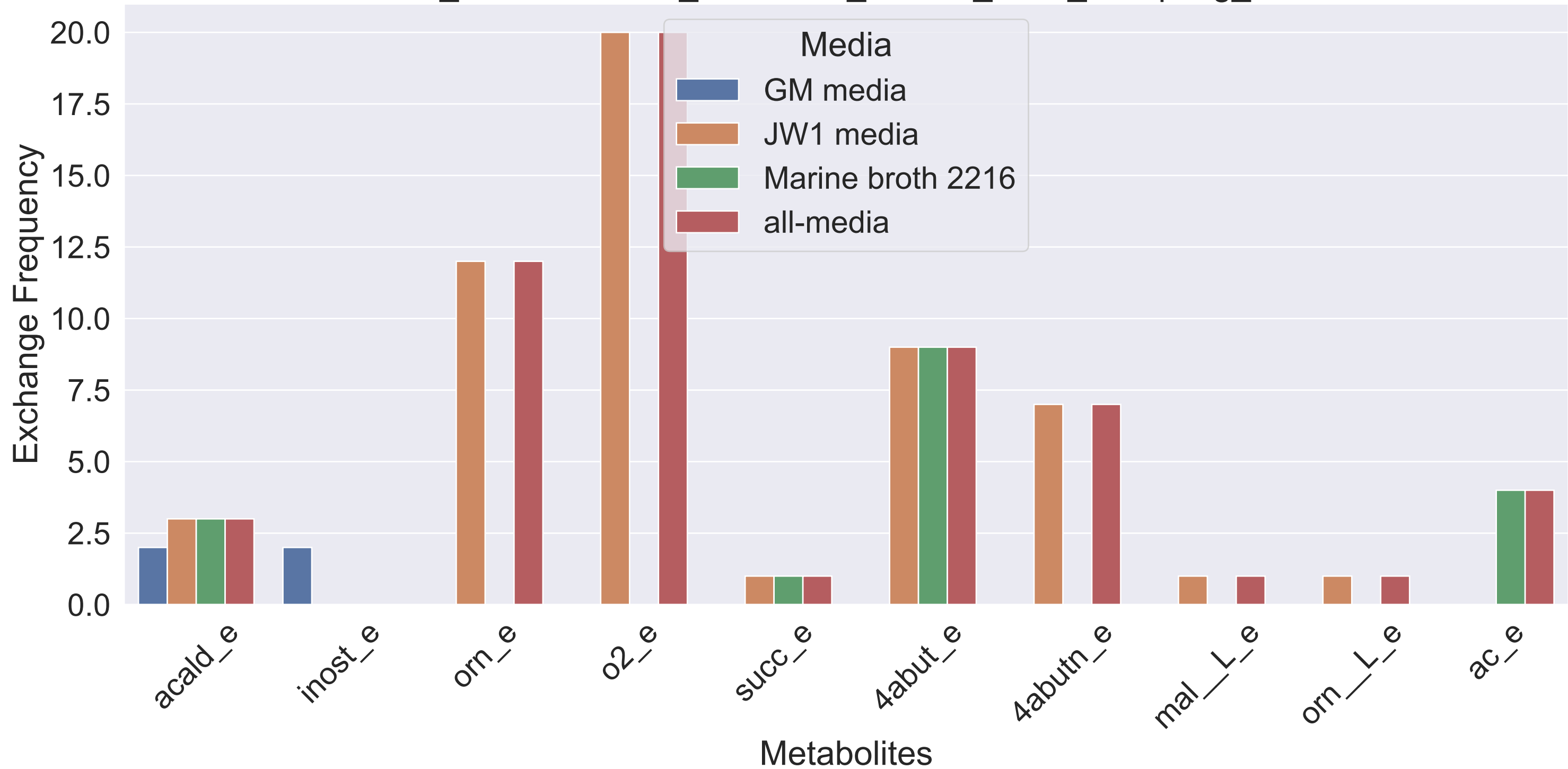

Supplement: Supplementary file 6 — Supplementary File S5 [file 43705_2023_242_MOESM6_ESM.zip › Supplementary Figure S16 Candidatus Marinimicrobia bacterium UWMA 0309 accepting metabolites.pdf]

# Candidatus\_Marinimicrobia\_bacterium\_UWMA\_0347\_accepting\_metabolites

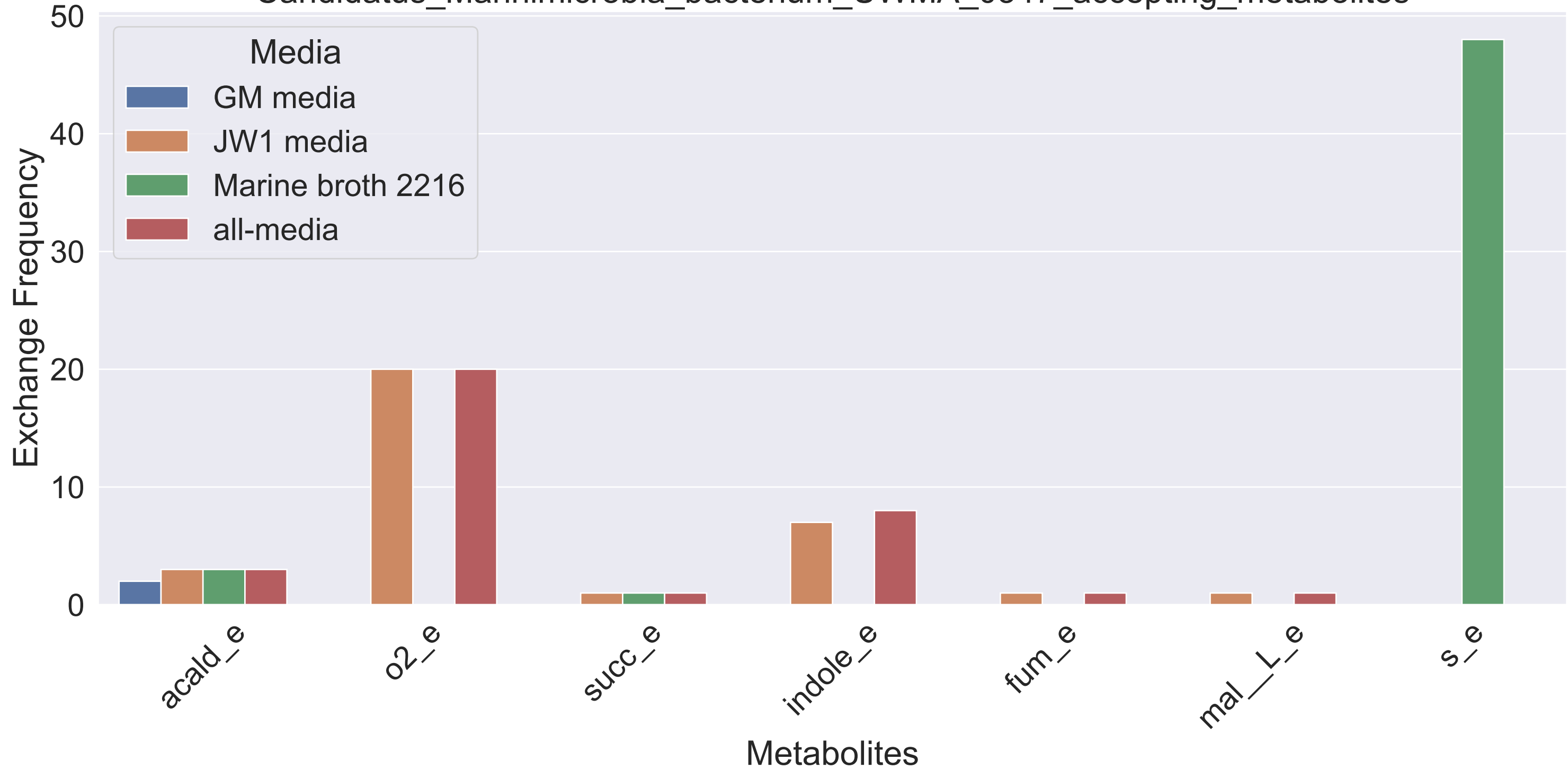

Supplement: Supplementary file 6 — Supplementary File S5 [file 43705_2023_242_MOESM6_ESM.zip › Supplementary Figure S17 Candidatus Marinimicrobia bacterium UWMA 0347 accepting metabolites.pdf]

# Candidatus\_Nitrosopelagicus\_sp\_UWMA\_0359\_accepting\_metabolites

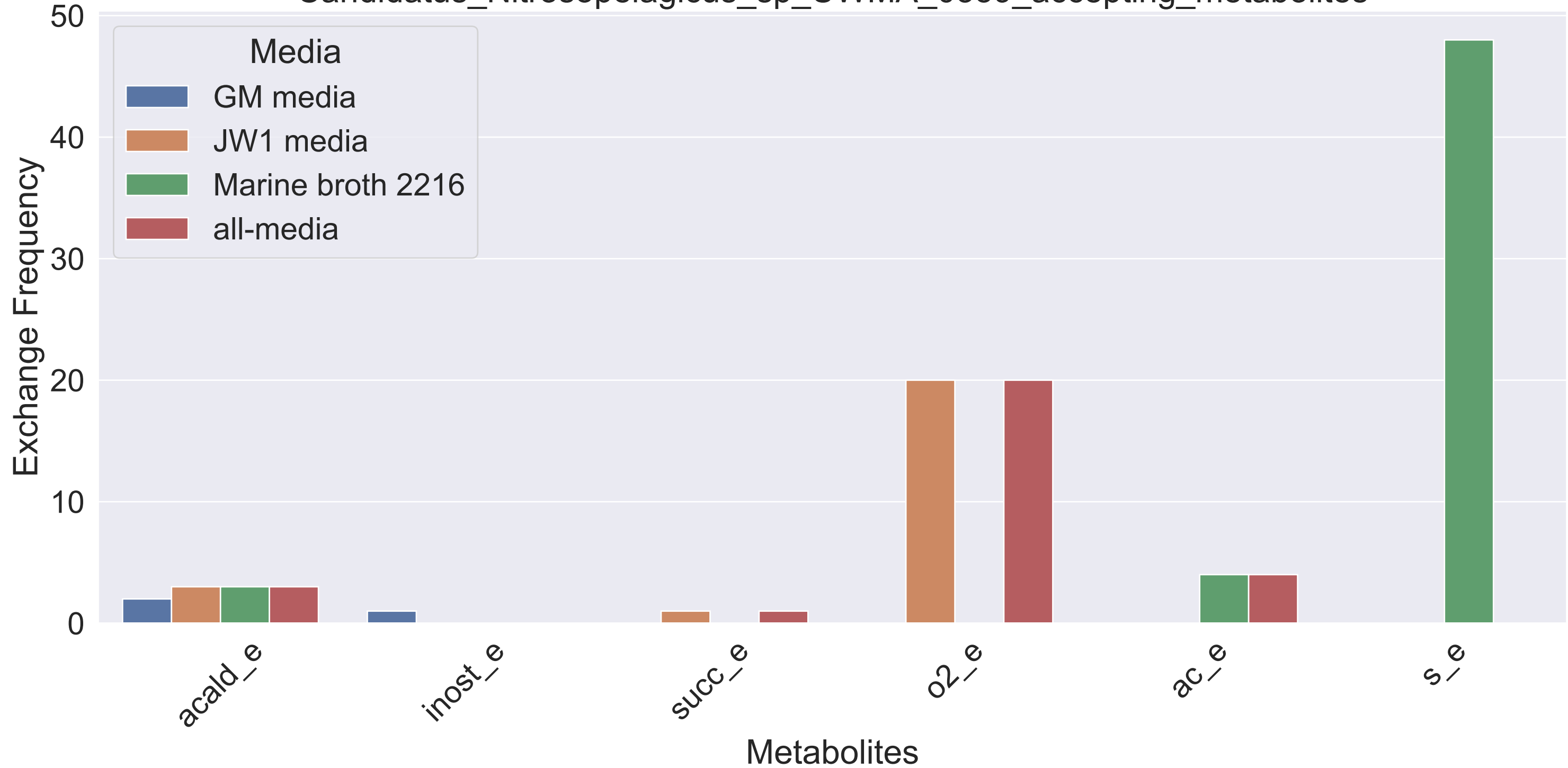

Supplement: Supplementary file 6 — Supplementary File S5 [file 43705_2023_242_MOESM6_ESM.zip › Supplementary Figure S18 Candidatus Nitrosopelagicus sp UWMA 0359 accepting metabolites.pdf]

Candidatus\_Pacearchaeota\_archaeon\_UWMA\_0287\_accepting\_metabolites

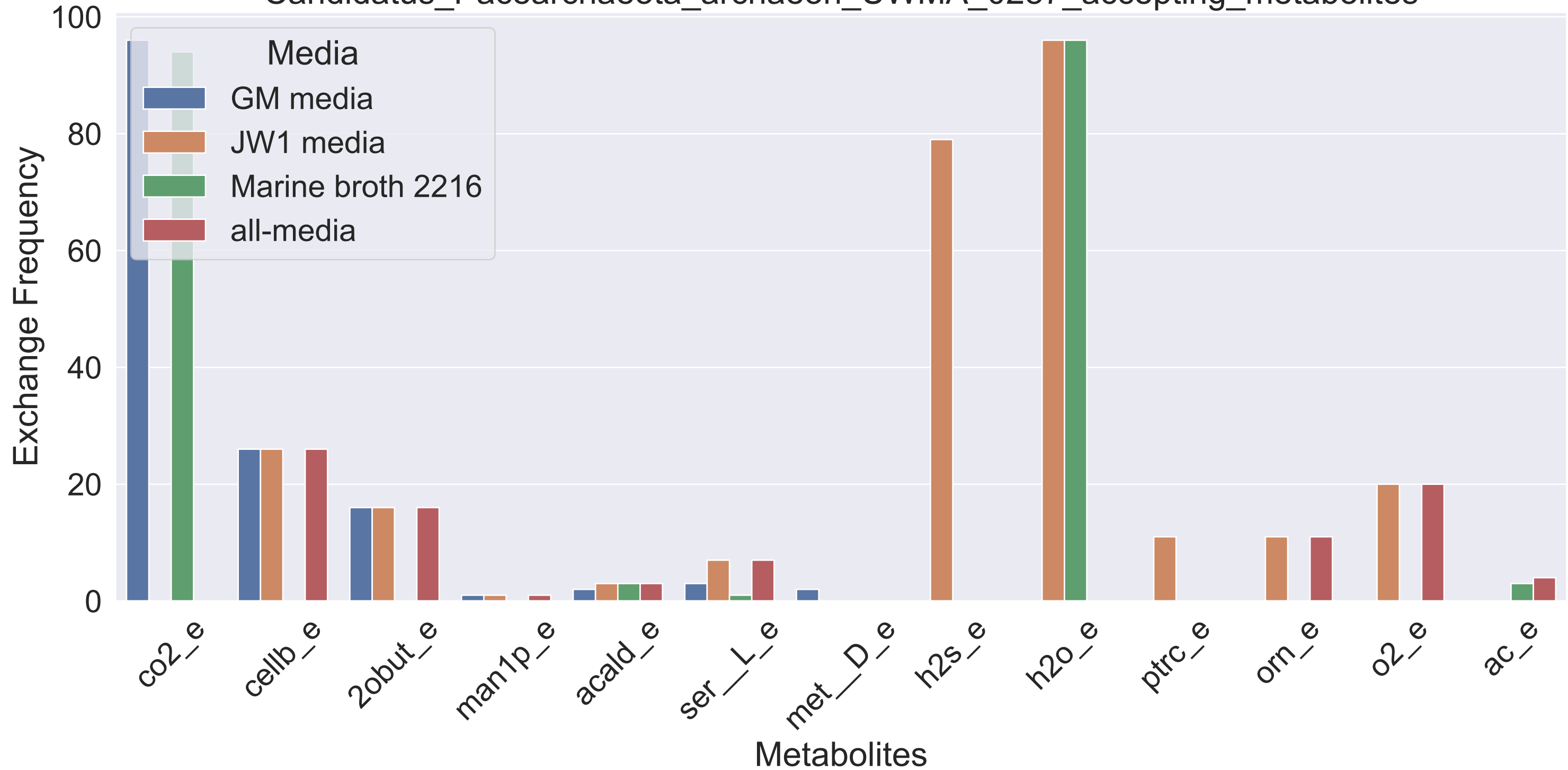

Supplement: Supplementary file 6 — Supplementary File S5 [file 43705_2023_242_MOESM6_ESM.zip › Supplementary Figure S19 Candidatus Pacearchaeota archaeon UWMA 0287 accepting metabolites.pdf]

# Acidimicrobiia\_bacterium\_UWMA\_0303\_accepting\_metabolites

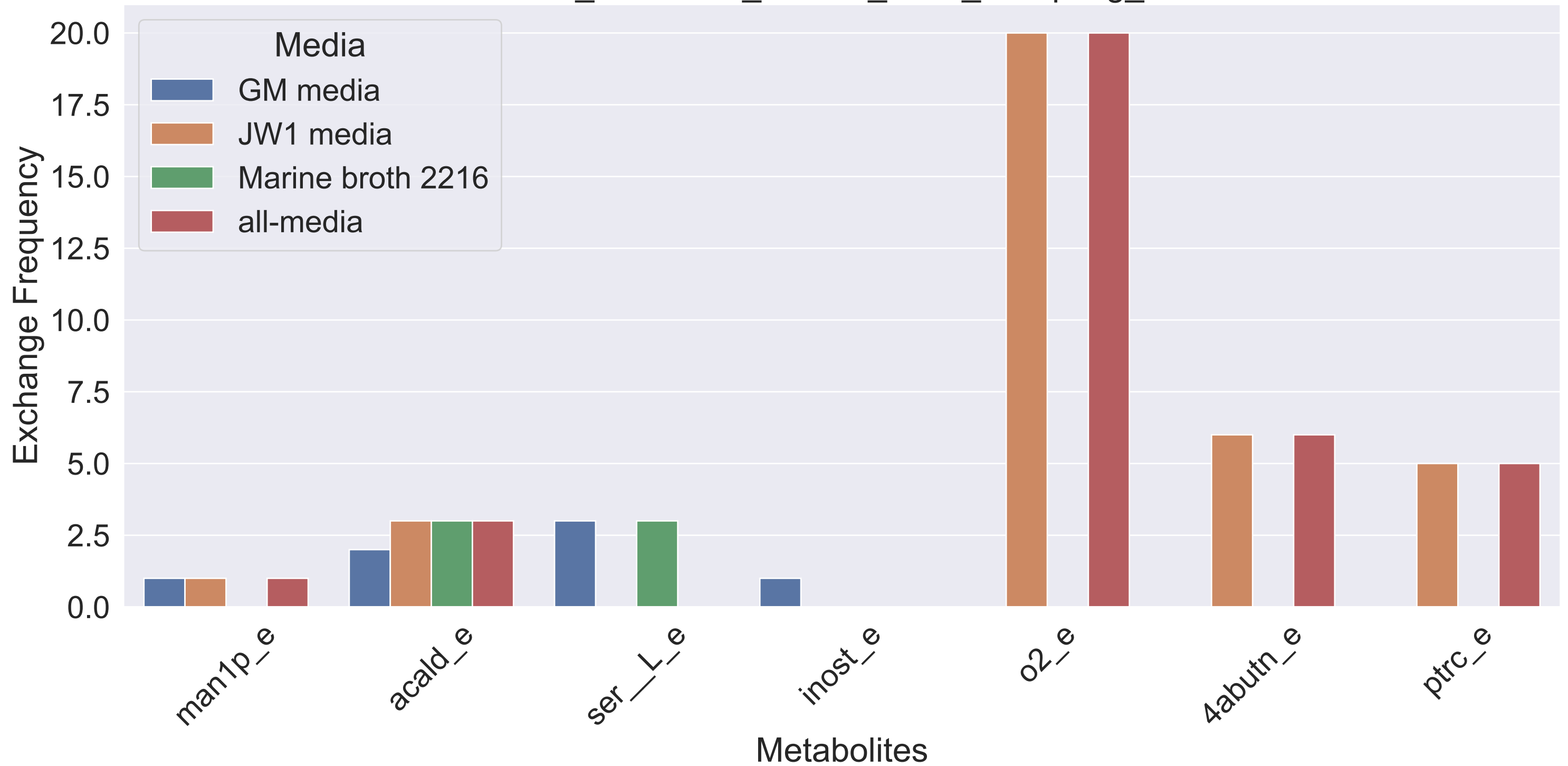

Supplement: Supplementary file 6 — Supplementary File S5 [file 43705_2023_242_MOESM6_ESM.zip › Supplementary Figure S2 Acidimicrobiia bacterium UWMA 0303 accepting metabolites.pdf]

Candidatus\_Pelagibacter\_sp\_UWMA\_0307\_accepting\_metabolites

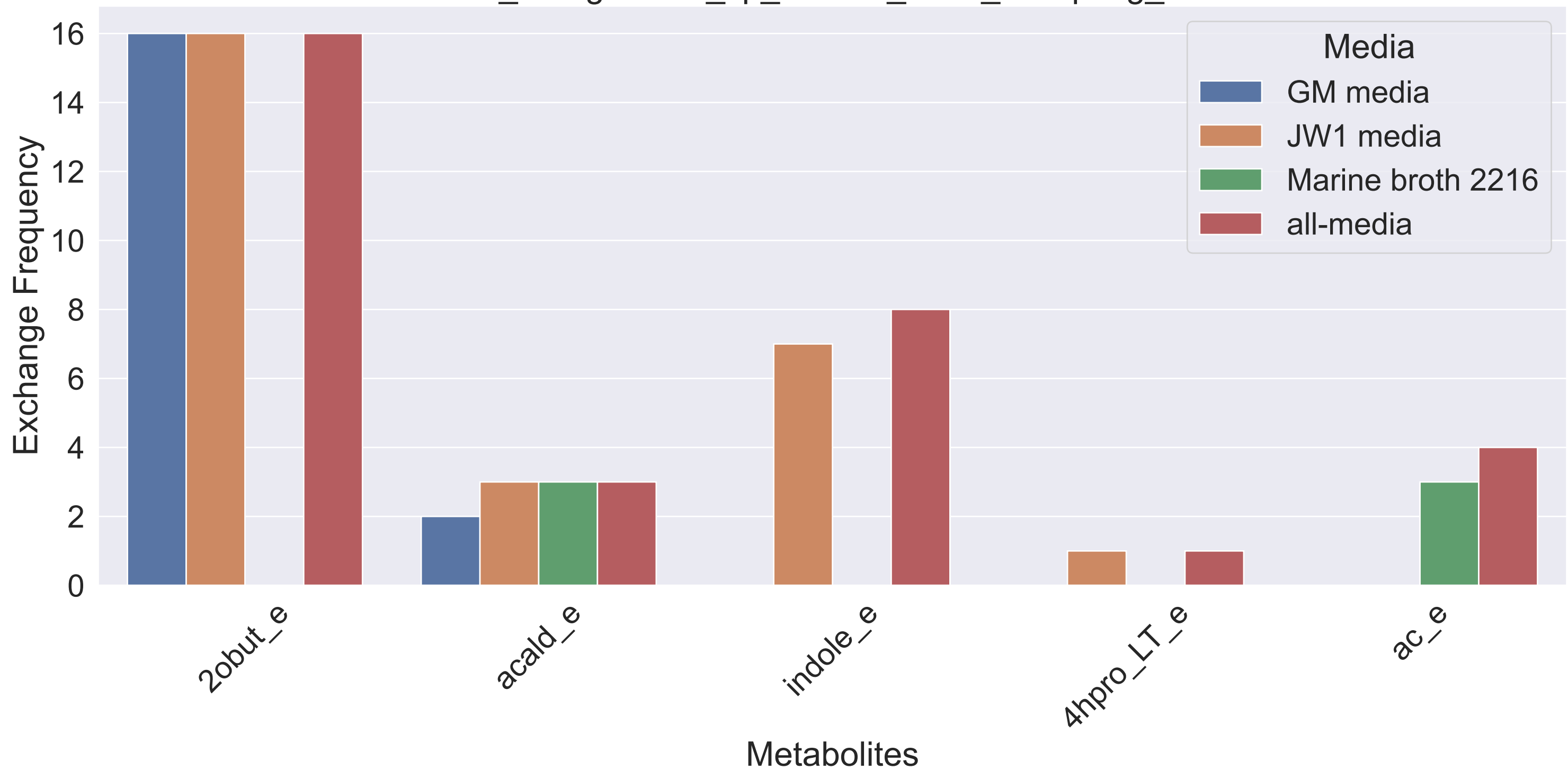

Supplement: Supplementary file 6 — Supplementary File S5 [file 43705_2023_242_MOESM6_ESM.zip › Supplementary Figure S20 Candidatus Pelagibacter sp UWMA 0307 accepting metabolites.pdf]

# Candidatus\_Thioglobus\_sp\_UWMA\_0259\_accepting\_metabolites

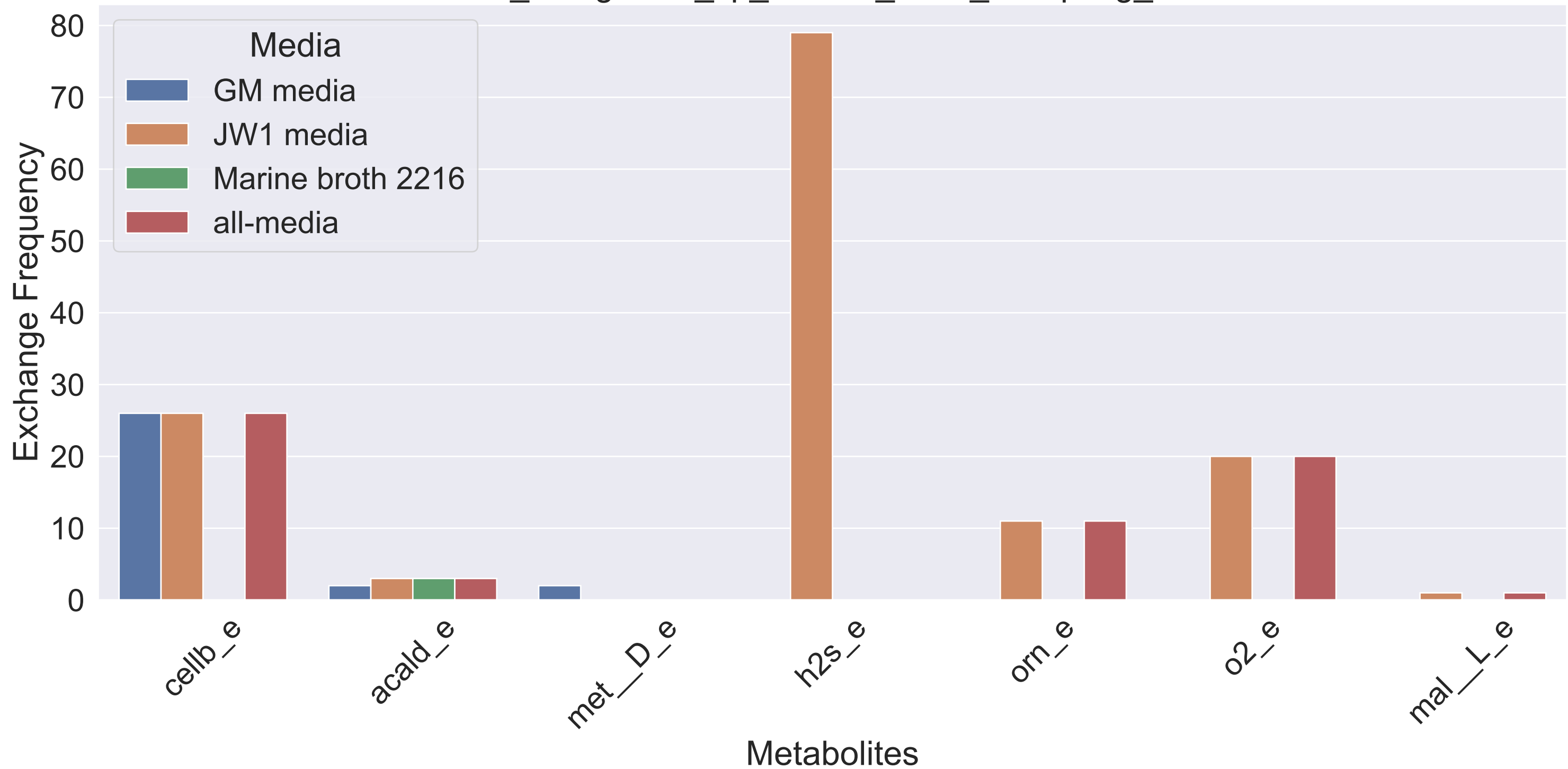

Supplement: Supplementary file 6 — Supplementary File S5 [file 43705_2023_242_MOESM6_ESM.zip › Supplementary Figure S21 Candidatus Thioglobus sp UWMA 0259 accepting metabolites.pdf]

Candidatus\_Thioglobus\_sp\_UWMA\_0272\_accepting\_metabolites

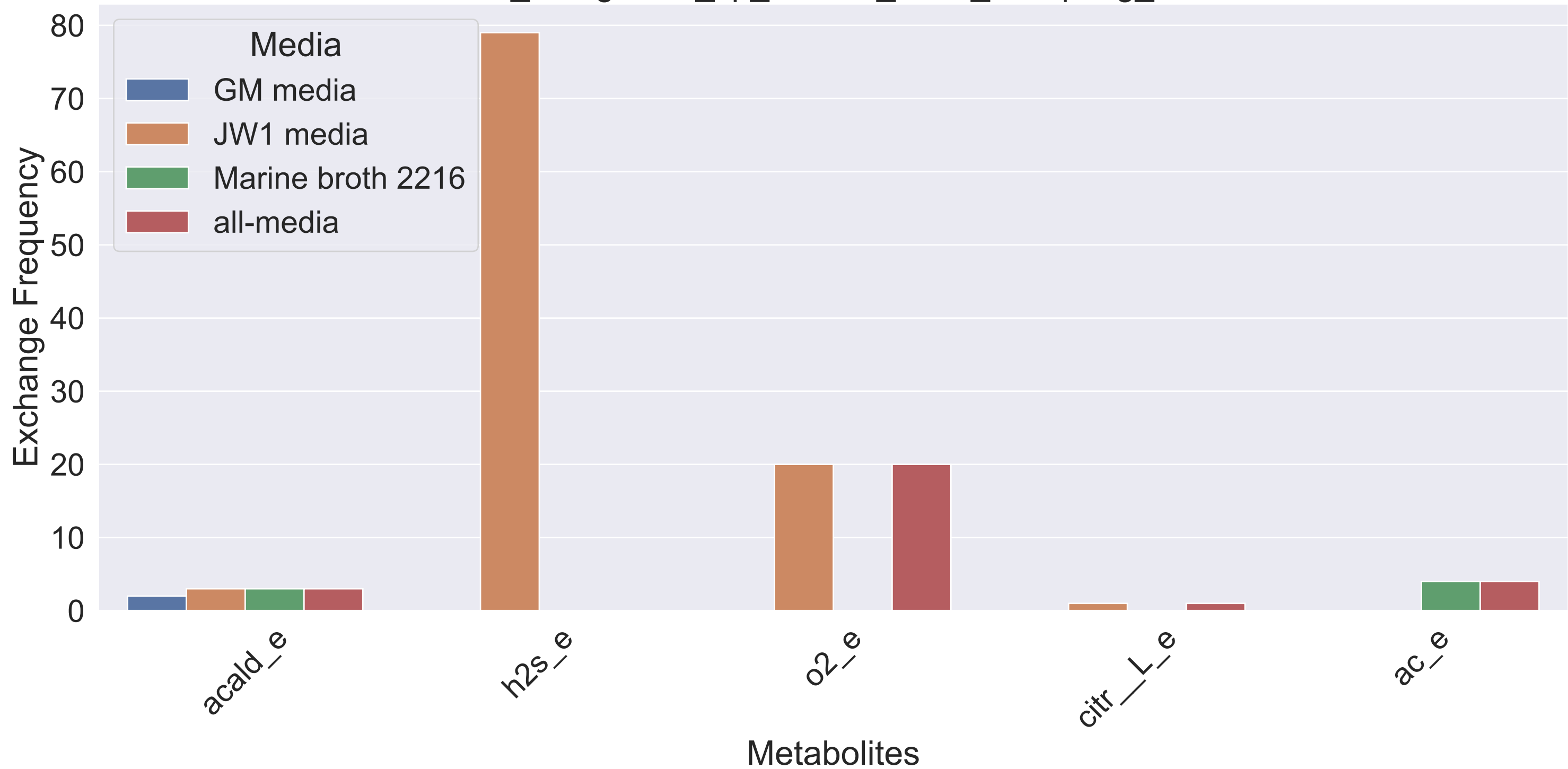

Supplement: Supplementary file 6 — Supplementary File S5 [file 43705_2023_242_MOESM6_ESM.zip › Supplementary Figure S22 Candidatus Thioglobus sp UWMA 0272 accepting metabolites.pdf]

# Candidatus\_Thioglobus\_sp\_UWMA\_0322\_accepting\_metabolites

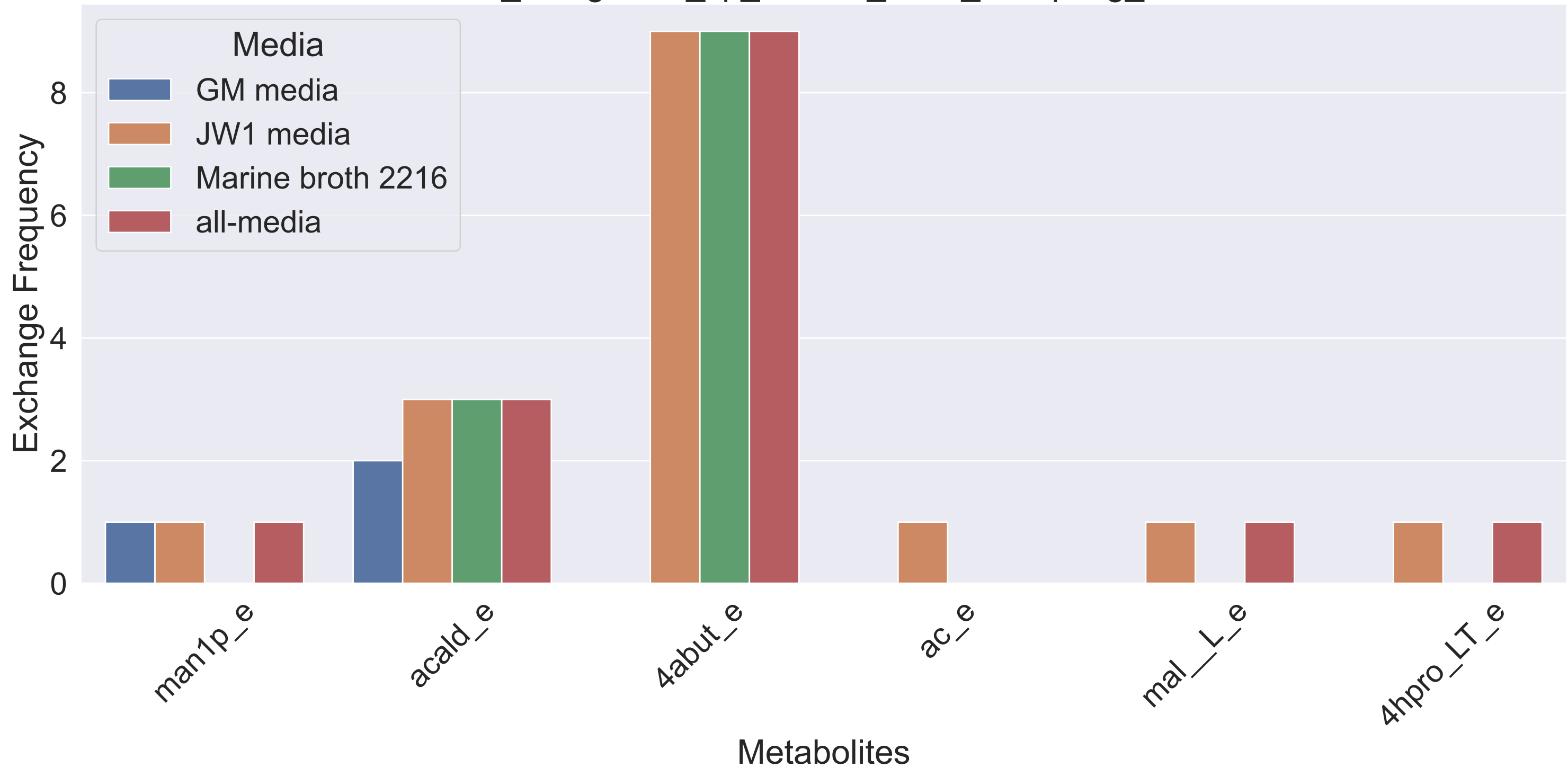

Supplement: Supplementary file 6 — Supplementary File S5 [file 43705_2023_242_MOESM6_ESM.zip › Supplementary Figure S23 Candidatus Thioglobus sp UWMA 0322 accepting metabolites.pdf]

Candidatus\_Thioglobus\_sp\_UWMA\_0342\_accepting\_metabolites

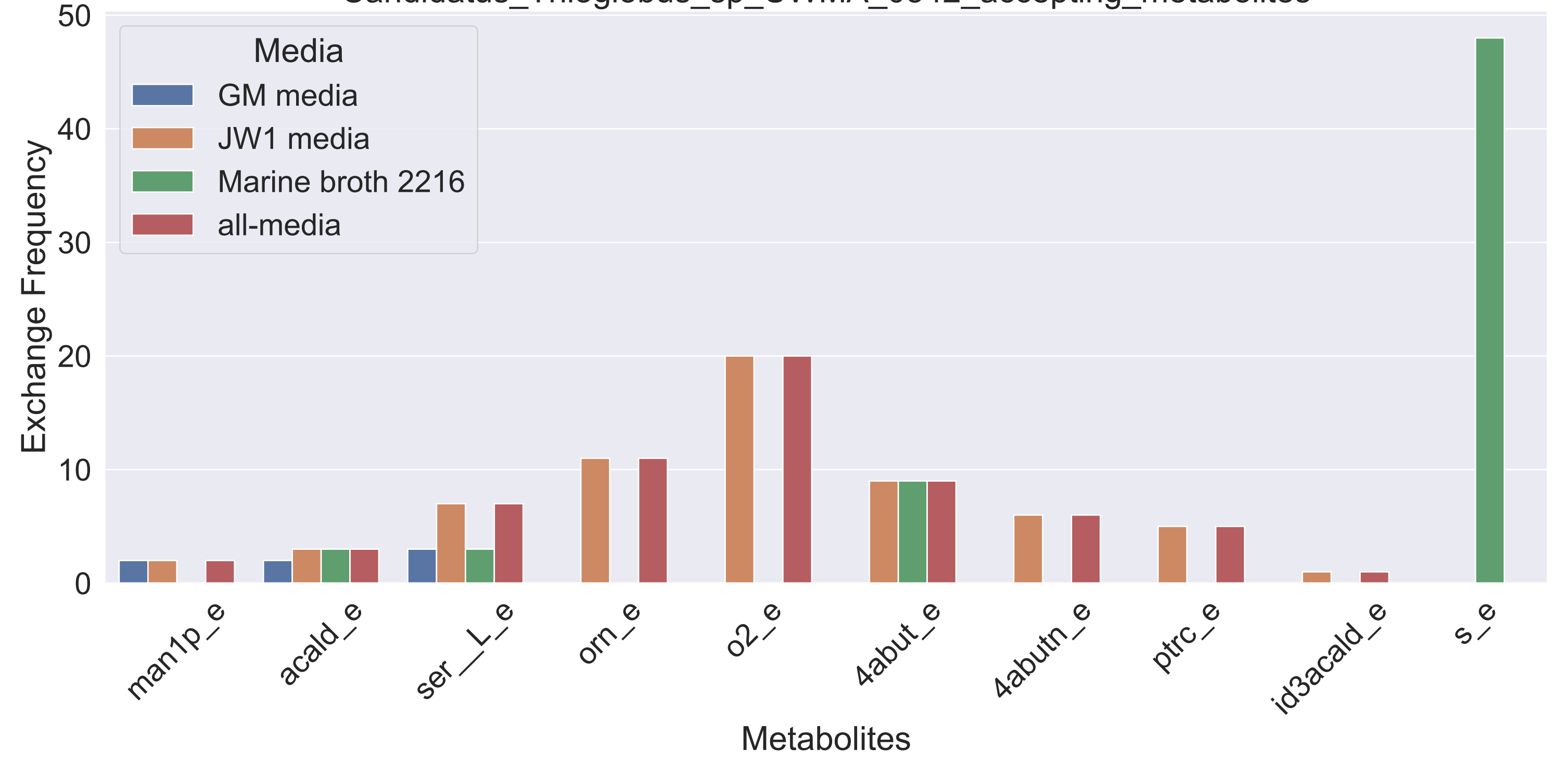

Supplement: Supplementary file 6 — Supplementary File S5 [file 43705_2023_242_MOESM6_ESM.zip › Supplementary Figure S24 Candidatus Thioglobus sp UWMA 0342 accepting metabolites.pdf]

Candidatus\_Thioglobus\_sp\_UWMA\_0360\_accepting\_metabolites

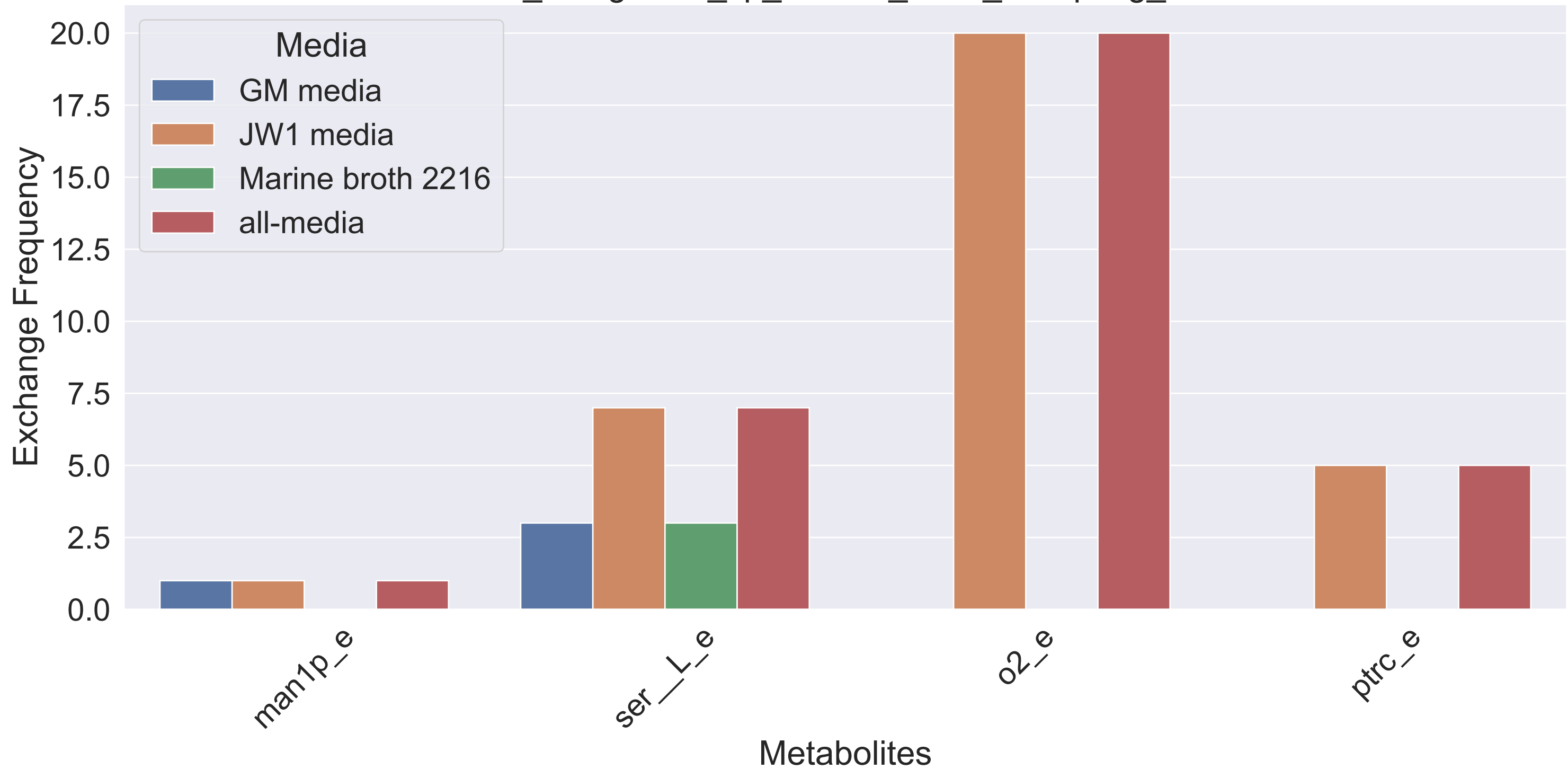

Supplement: Supplementary file 6 — Supplementary File S5 [file 43705_2023_242_MOESM6_ESM.zip › Supplementary Figure S25 Candidatus Thioglobus sp UWMA 0360 accepting metabolites.pdf]

Cycloclasticus\_sp\_UWMA\_0316\_accepting\_metabolites

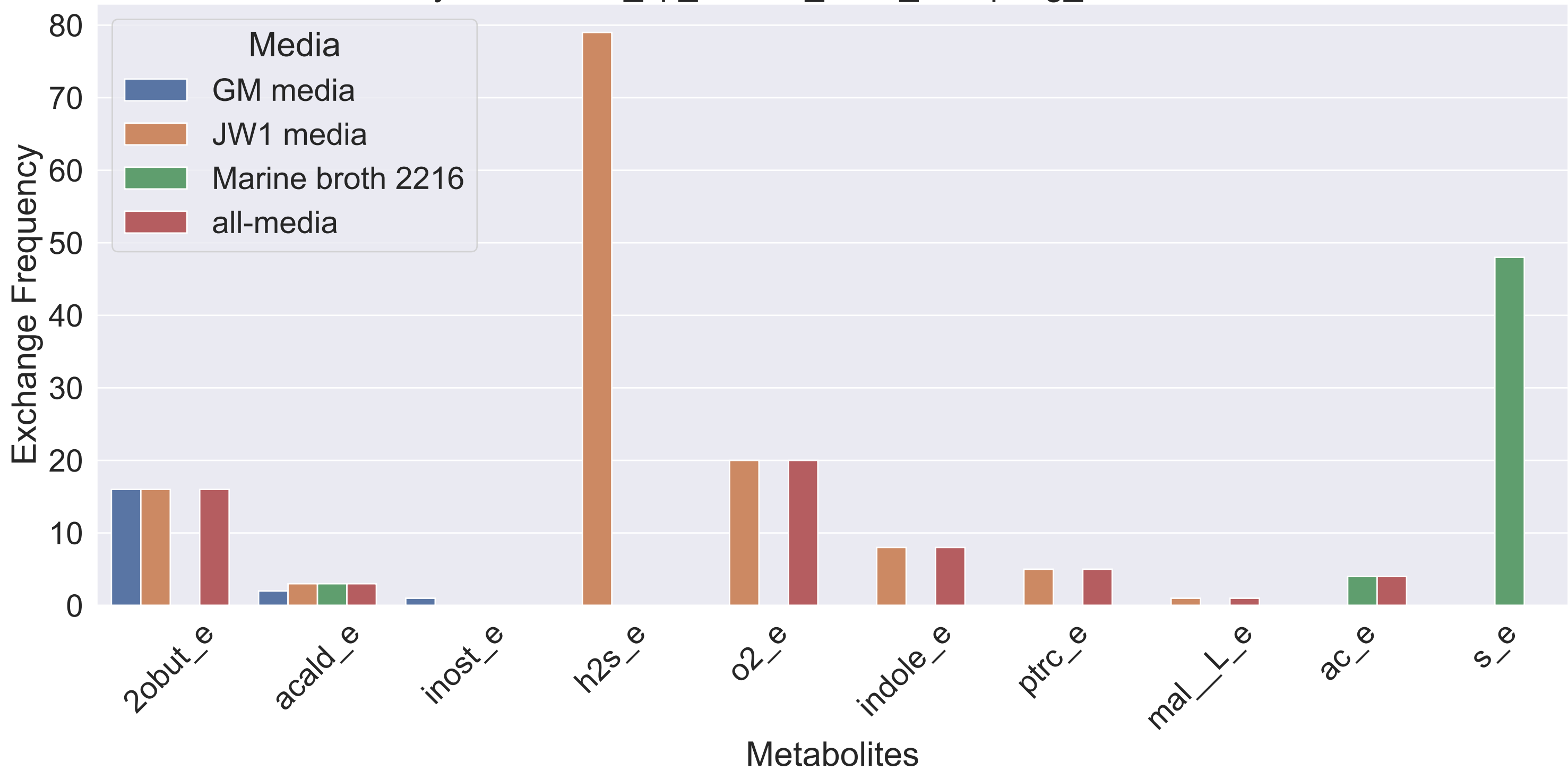

Supplement: Supplementary file 6 — Supplementary File S5 [file 43705_2023_242_MOESM6_ESM.zip › Supplementary Figure S26 Cycloclasticus sp UWMA 0316 accepting metabolites.pdf]

# Cycloclasticus\_sp\_UWMA\_0335\_accepting\_metabolites

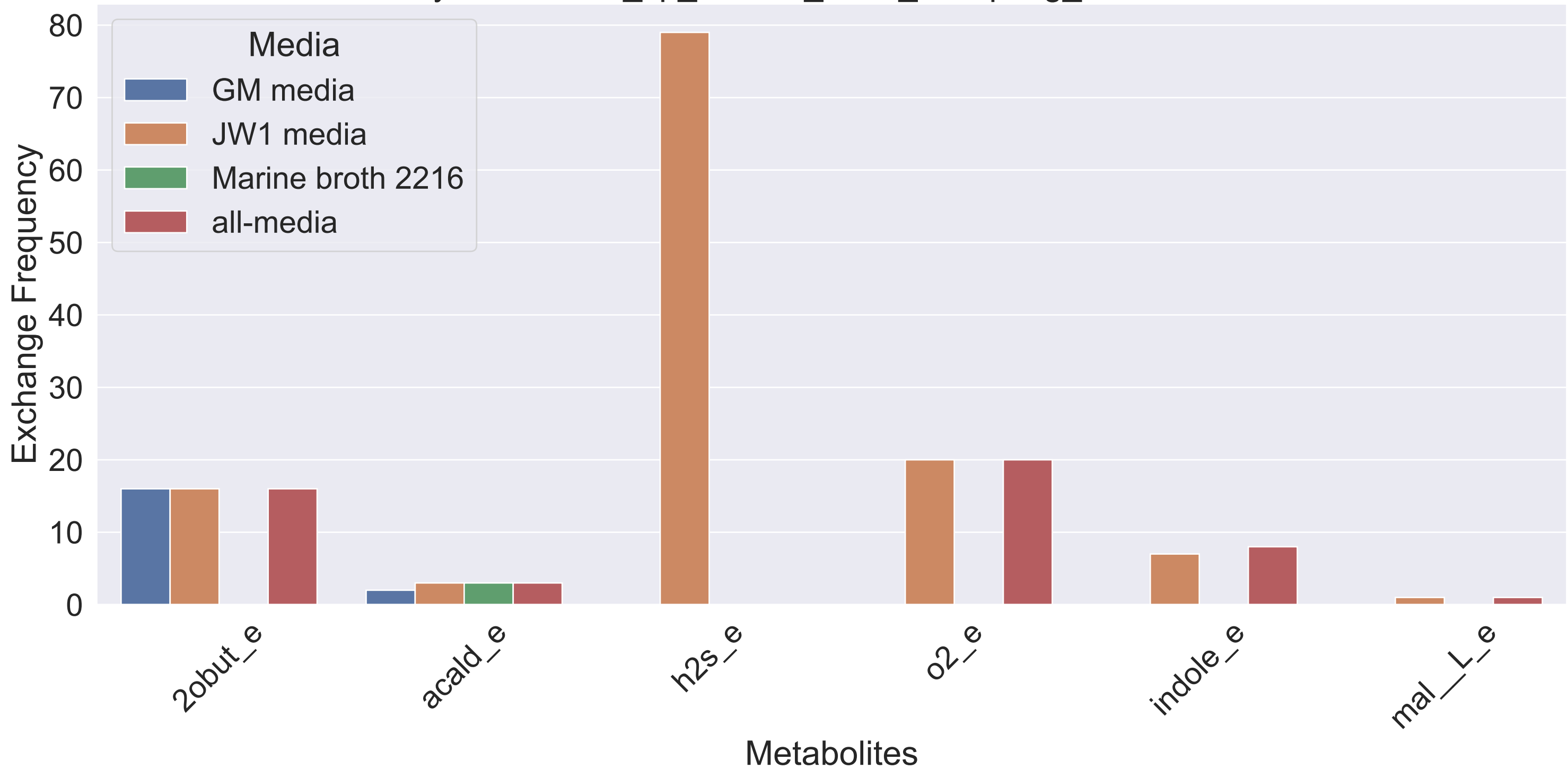

Supplement: Supplementary file 6 — Supplementary File S5 [file 43705_2023_242_MOESM6_ESM.zip › Supplementary Figure S27 Cycloclasticus sp UWMA 0335 accepting metabolites.pdf]

# Cytophagales\_bacterium\_UWMA\_0338\_accepting\_metabolites

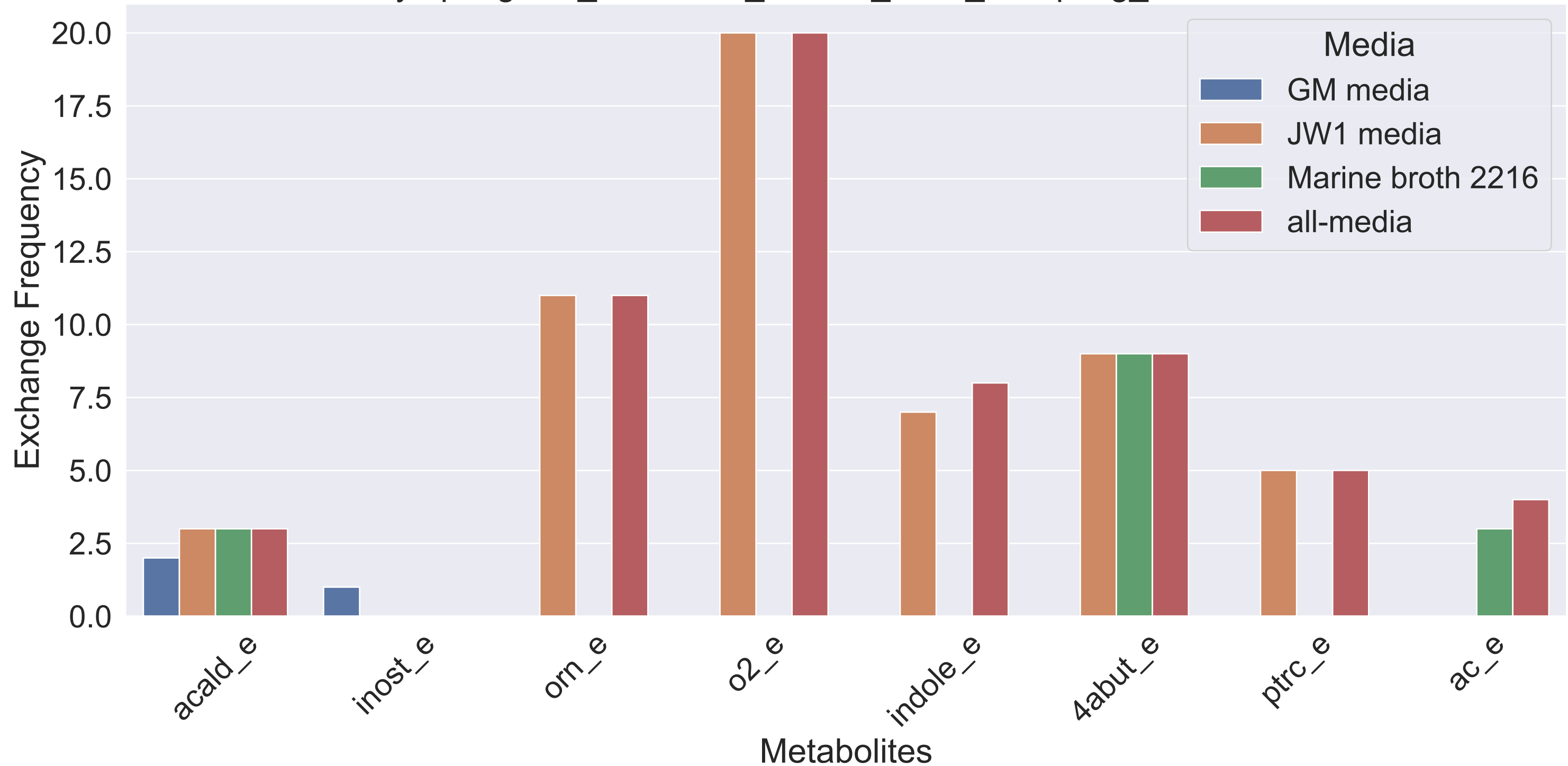

Supplement: Supplementary file 6 — Supplementary File S5 [file 43705_2023_242_MOESM6_ESM.zip › Supplementary Figure S28 Cytophagales bacterium UWMA 0338 accepting metabolites.pdf]

Dehalococcoidia\_bacterium\_UWMA\_0267\_accepting\_metabolites

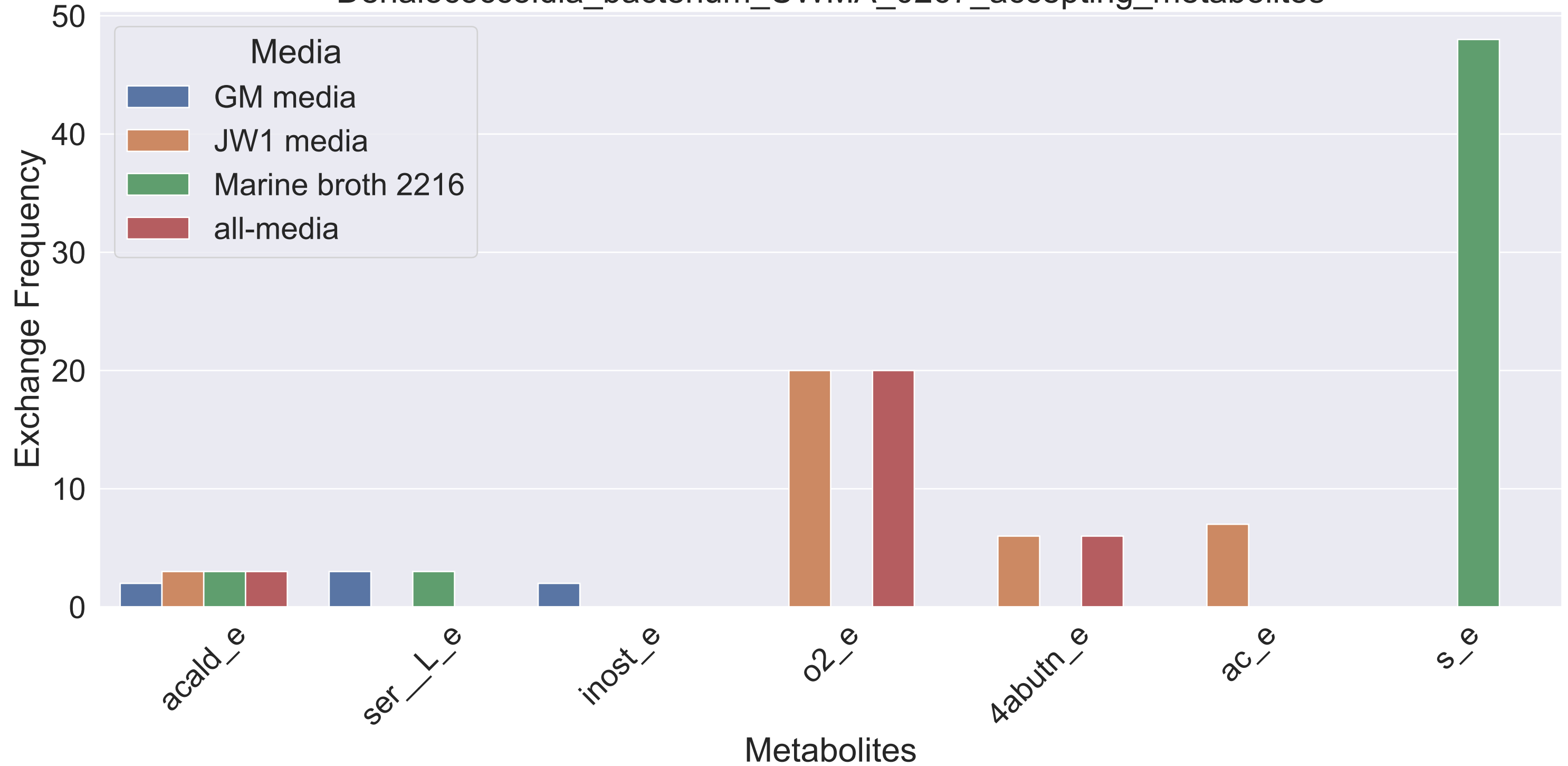

Supplement: Supplementary file 6 — Supplementary File S5 [file 43705_2023_242_MOESM6_ESM.zip › Supplementary Figure S29 Dehalococcoidia bacterium UWMA 0267 accepting metabolites.pdf]

# Acidimicrobiia\_bacterium\_UWMA\_0319\_accepting\_metabolites

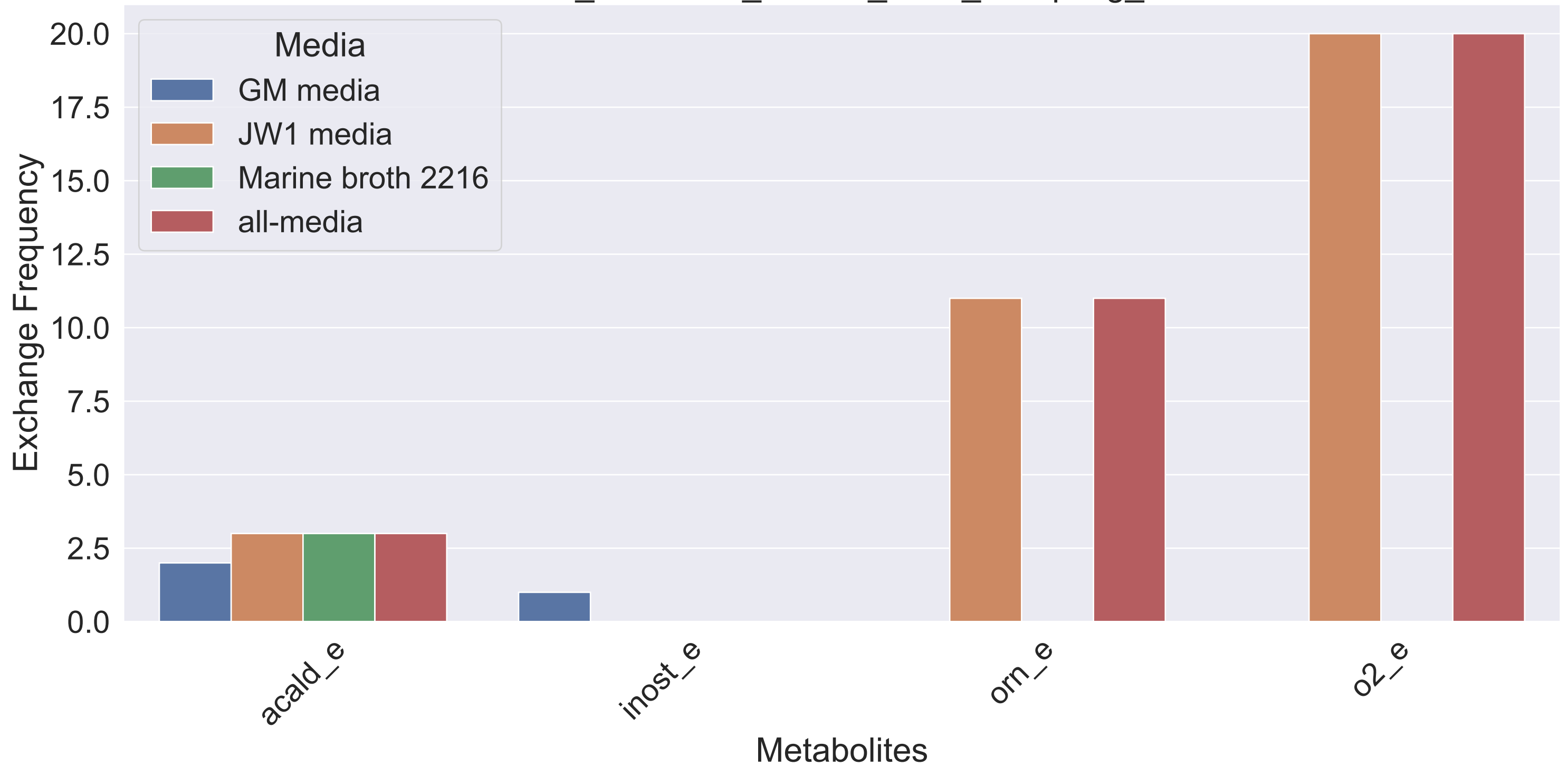

Supplement: Supplementary file 6 — Supplementary File S5 [file 43705_2023_242_MOESM6_ESM.zip › Supplementary Figure S3 Acidimicrobiia bacterium UWMA 0319 accepting metabolites.pdf]

# Dehalococcoidia\_bacterium\_UWMA\_0331\_accepting\_metabolites

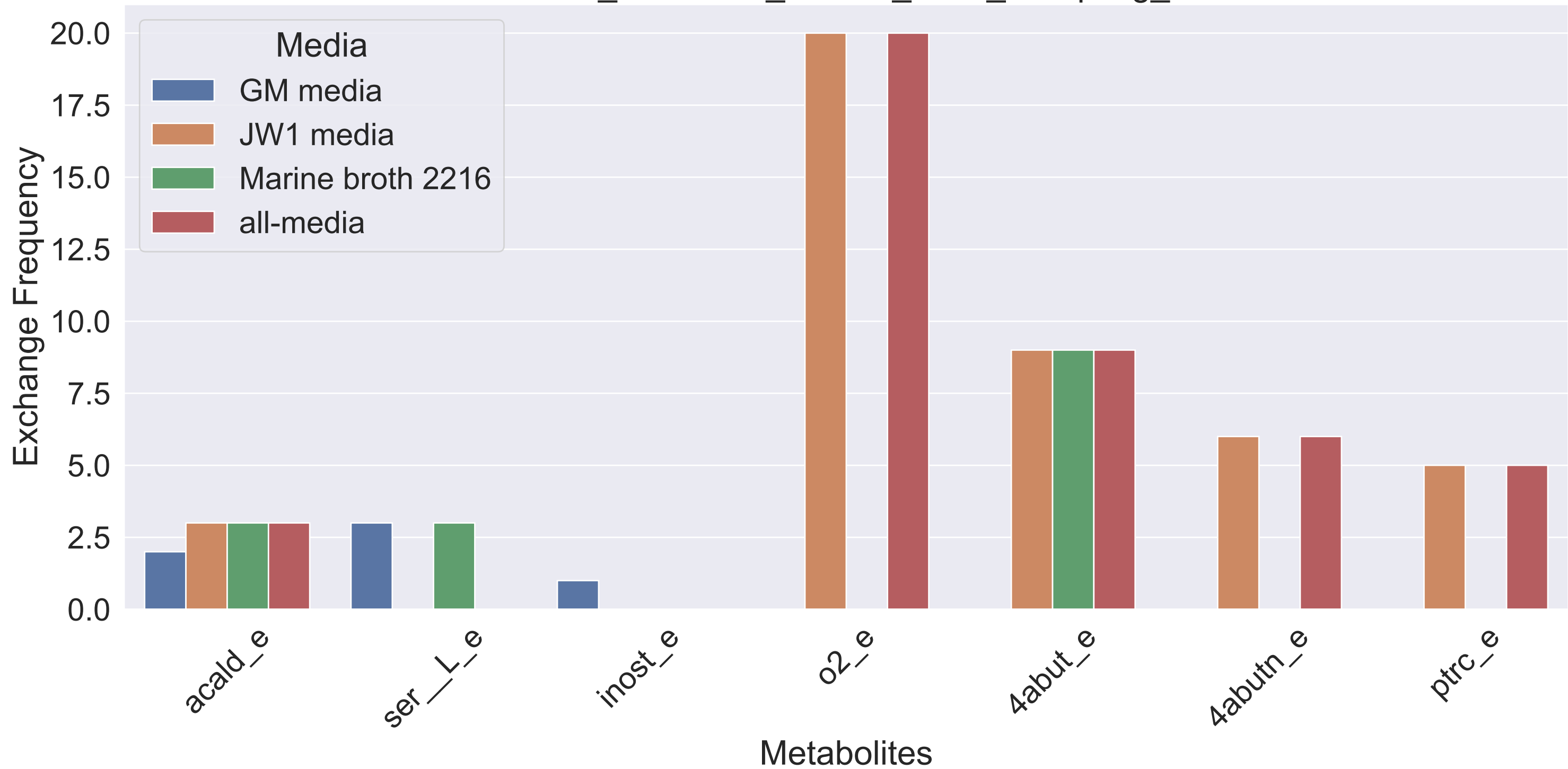

Supplement: Supplementary file 6 — Supplementary File S5 [file 43705_2023_242_MOESM6_ESM.zip › Supplementary Figure S30 Dehalococcoidia bacterium UWMA 0331 accepting metabolites.pdf]

# Dehalococcoidia\_bacterium\_UWMA\_0337\_accepting\_metabolites

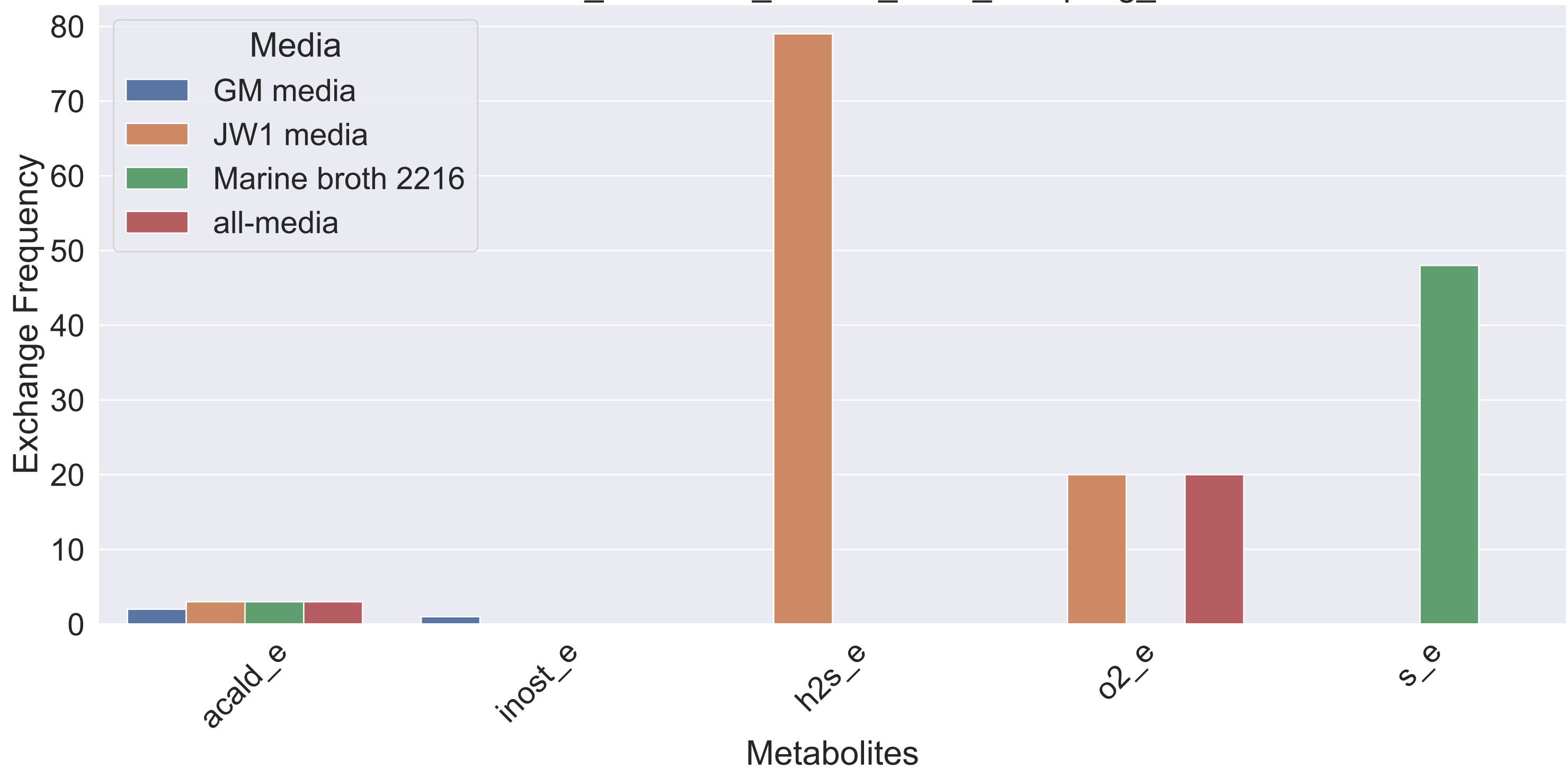

Supplement: Supplementary file 6 — Supplementary File S5 [file 43705_2023_242_MOESM6_ESM.zip › Supplementary Figure S31 Dehalococcoidia bacterium UWMA 0337 accepting metabolites.pdf]

# Dehalococcoidia\_bacterium\_UWMA\_0354\_accepting\_metabolites

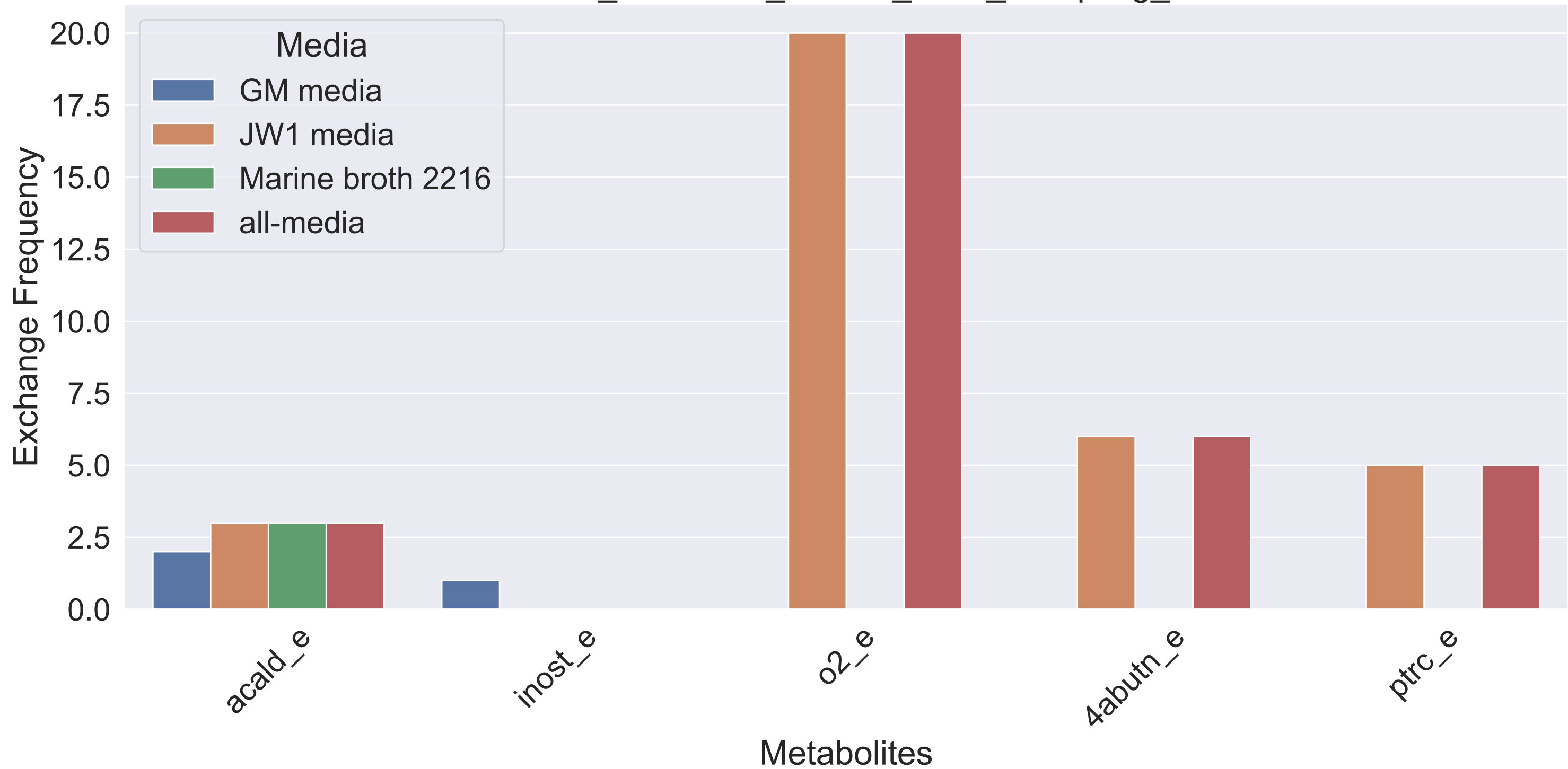

Supplement: Supplementary file 6 — Supplementary File S5 [file 43705_2023_242_MOESM6_ESM.zip › Supplementary Figure S32 Dehalococcoidia bacterium UWMA 0354 accepting metabolites.pdf]

# Flavobacteriaceae\_bacterium\_UWMA\_0314\_accepting\_metabolites

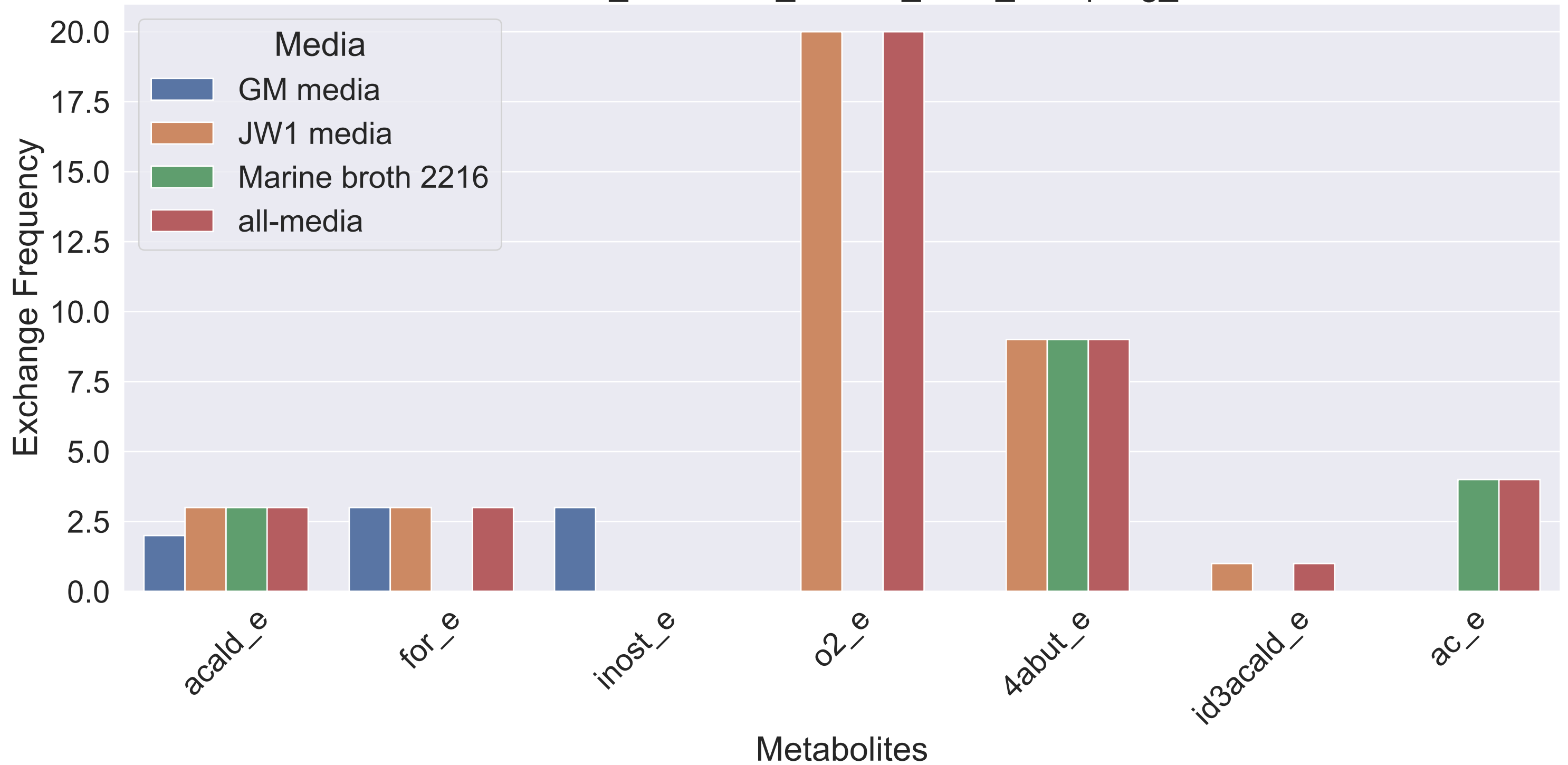

Supplement: Supplementary file 6 — Supplementary File S5 [file 43705_2023_242_MOESM6_ESM.zip › Supplementary Figure S33 Flavobacteriaceae bacterium UWMA 0314 accepting metabolites.pdf]

# Flavobacteriaceae\_bacterium\_UWMA\_0351\_accepting\_metabolites

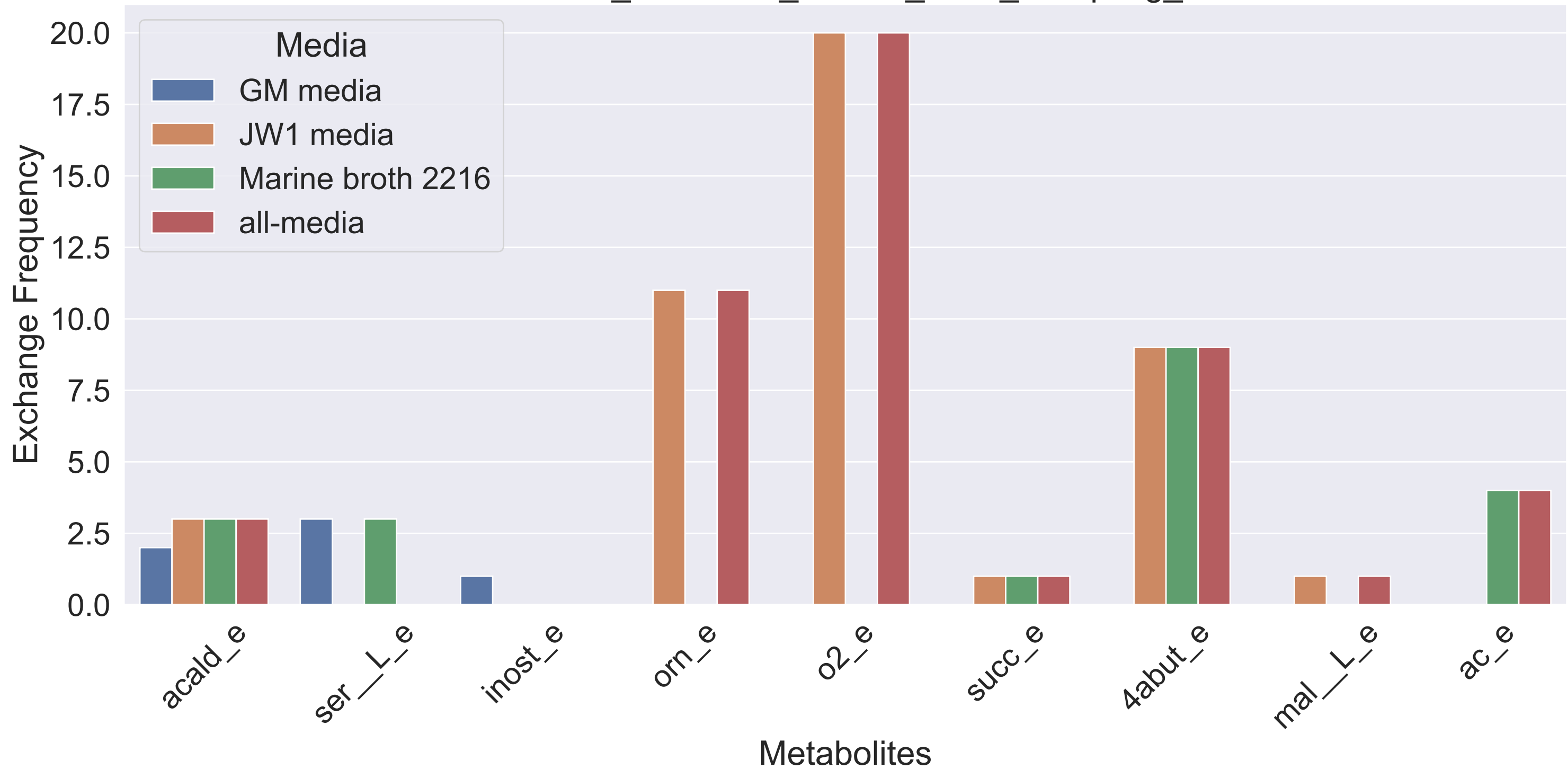

Supplement: Supplementary file 6 — Supplementary File S5 [file 43705_2023_242_MOESM6_ESM.zip › Supplementary Figure S34 Flavobacteriaceae bacterium UWMA 0351 accepting metabolites.pdf]

Flavobacteriales\_bacterium\_UWMA\_0274\_accepting\_metabolites

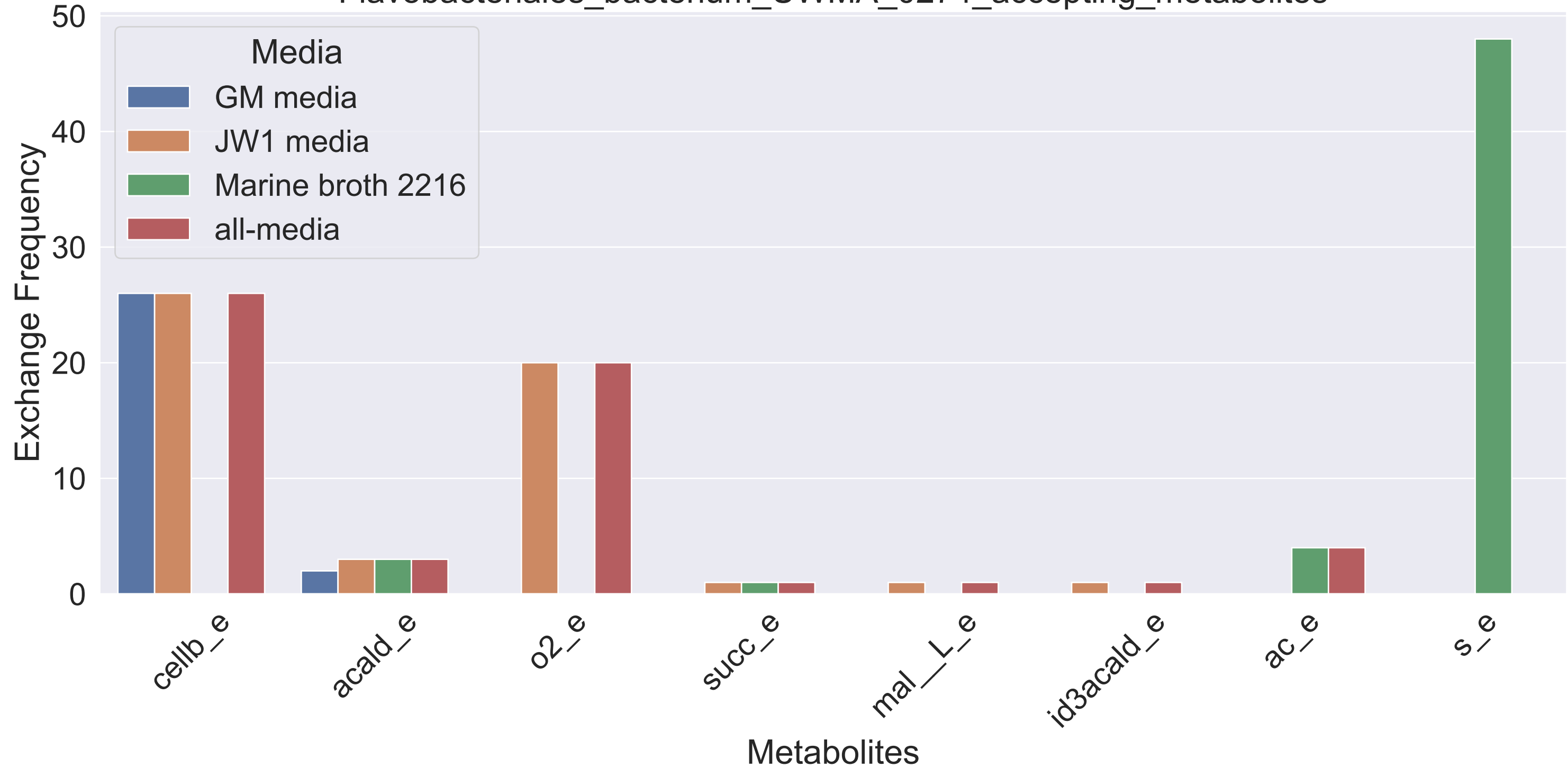

Supplement: Supplementary file 6 — Supplementary File S5 [file 43705_2023_242_MOESM6_ESM.zip › Supplementary Figure S35 Flavobacteriales bacterium UWMA 0274 accepting metabolites.pdf]

Flavobacteriales\_bacterium\_UWMA\_0277\_accepting\_metabolites

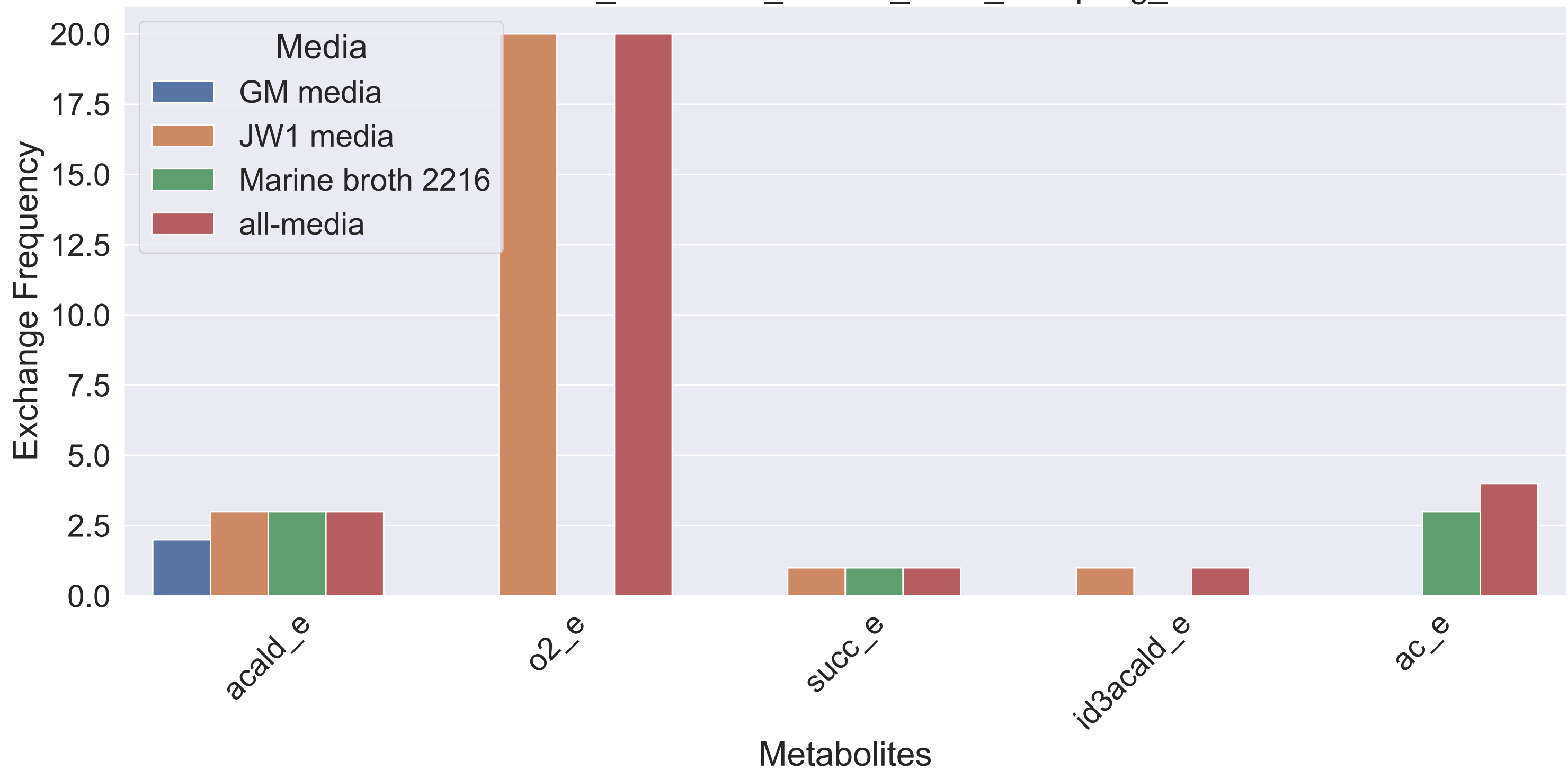

Supplement: Supplementary file 6 — Supplementary File S5 [file 43705_2023_242_MOESM6_ESM.zip › Supplementary Figure S36 Flavobacteriales bacterium UWMA 0277 accepting metabolites.pdf]

Flavobacteriales\_bacterium\_UWMA\_0289\_accepting\_metabolites

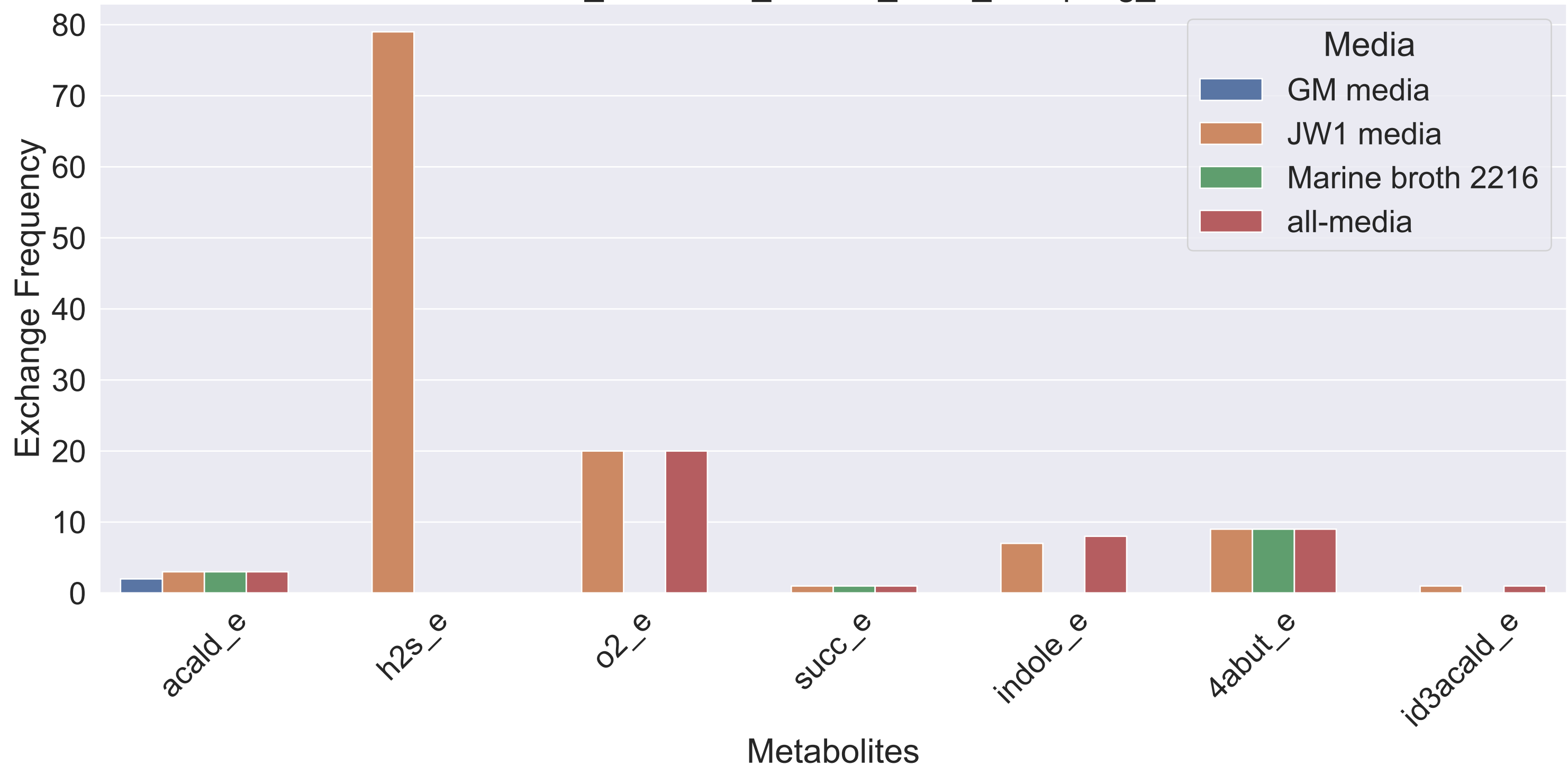

Supplement: Supplementary file 6 — Supplementary File S5 [file 43705_2023_242_MOESM6_ESM.zip › Supplementary Figure S37 Flavobacteriales bacterium UWMA 0289 accepting metabolites.pdf]

Fuerstia\_sp\_UWMA\_0333\_accepting\_metabolites

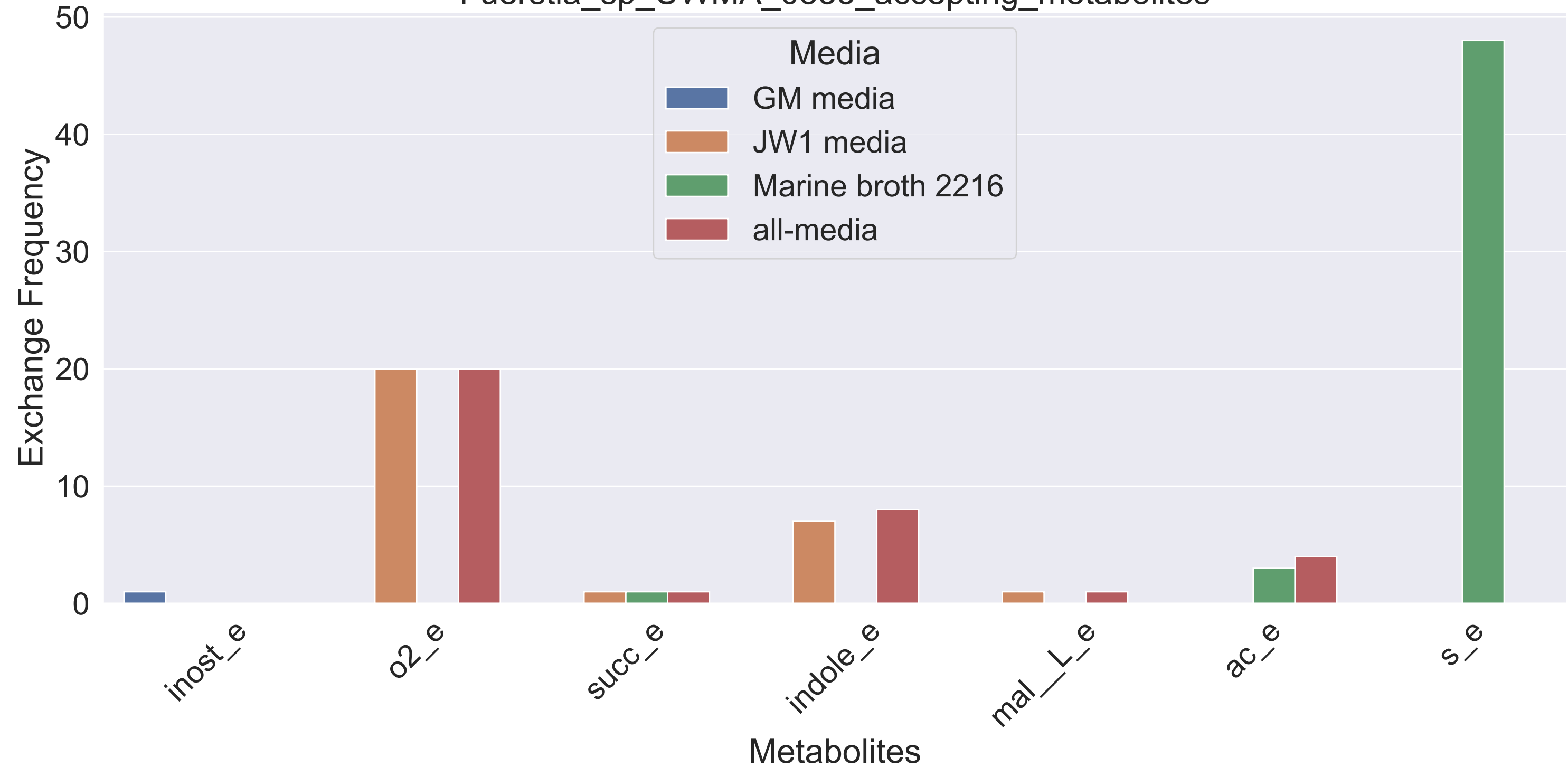

Supplement: Supplementary file 6 — Supplementary File S5 [file 43705_2023_242_MOESM6_ESM.zip › Supplementary Figure S38 Fuerstia sp UWMA 0333 accepting metabolites.pdf]

# Gammaproteobacteria\_bacterium\_UWMA\_0258\_accepting\_metabolites

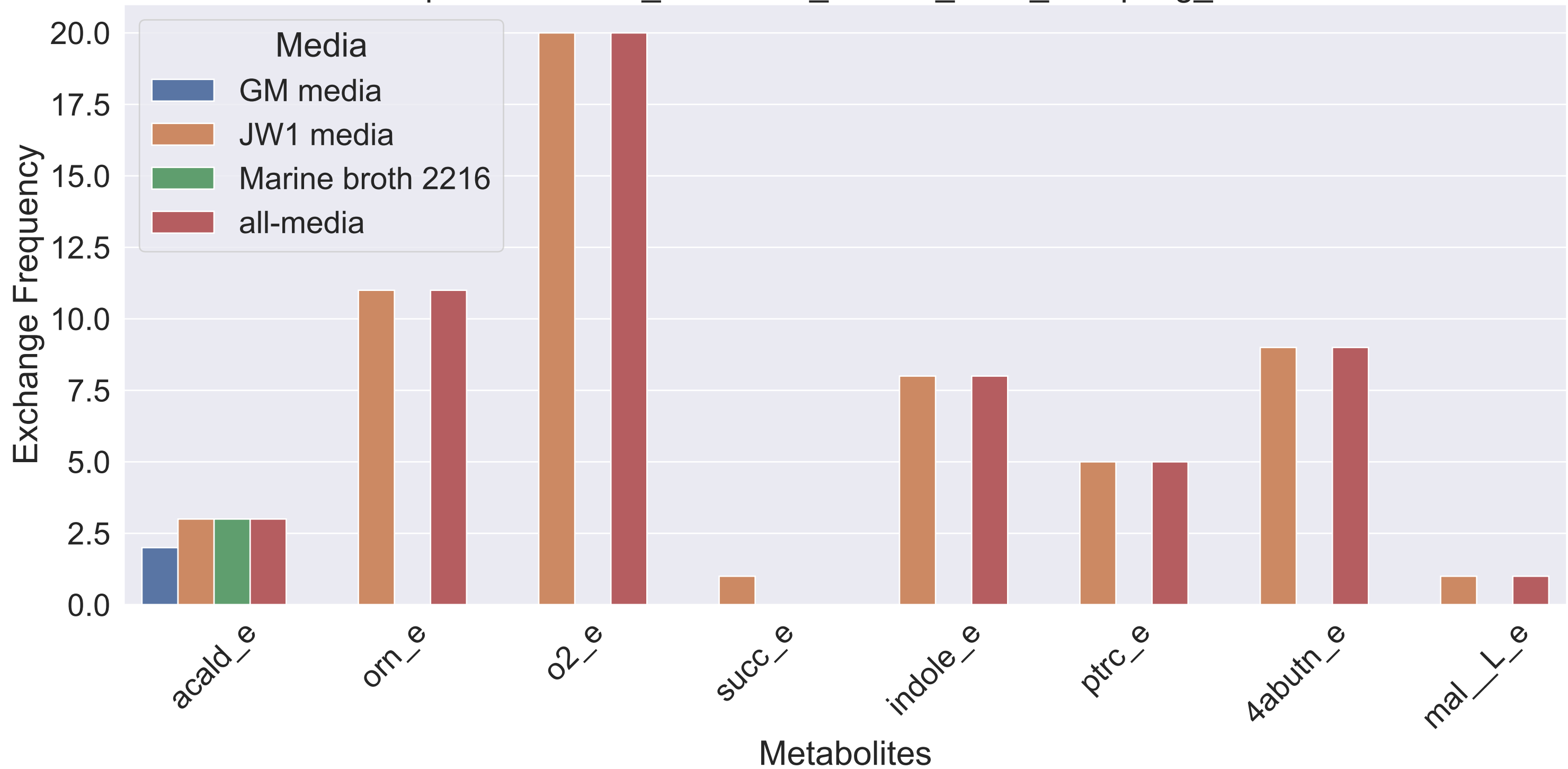

Supplement: Supplementary file 6 — Supplementary File S5 [file 43705_2023_242_MOESM6_ESM.zip › Supplementary Figure S39 Gammaproteobacteria bacterium UWMA 0258 accepting metabolites.pdf]

# Acidobacteria\_bacterium\_UWMA\_0355\_accepting\_metabolites

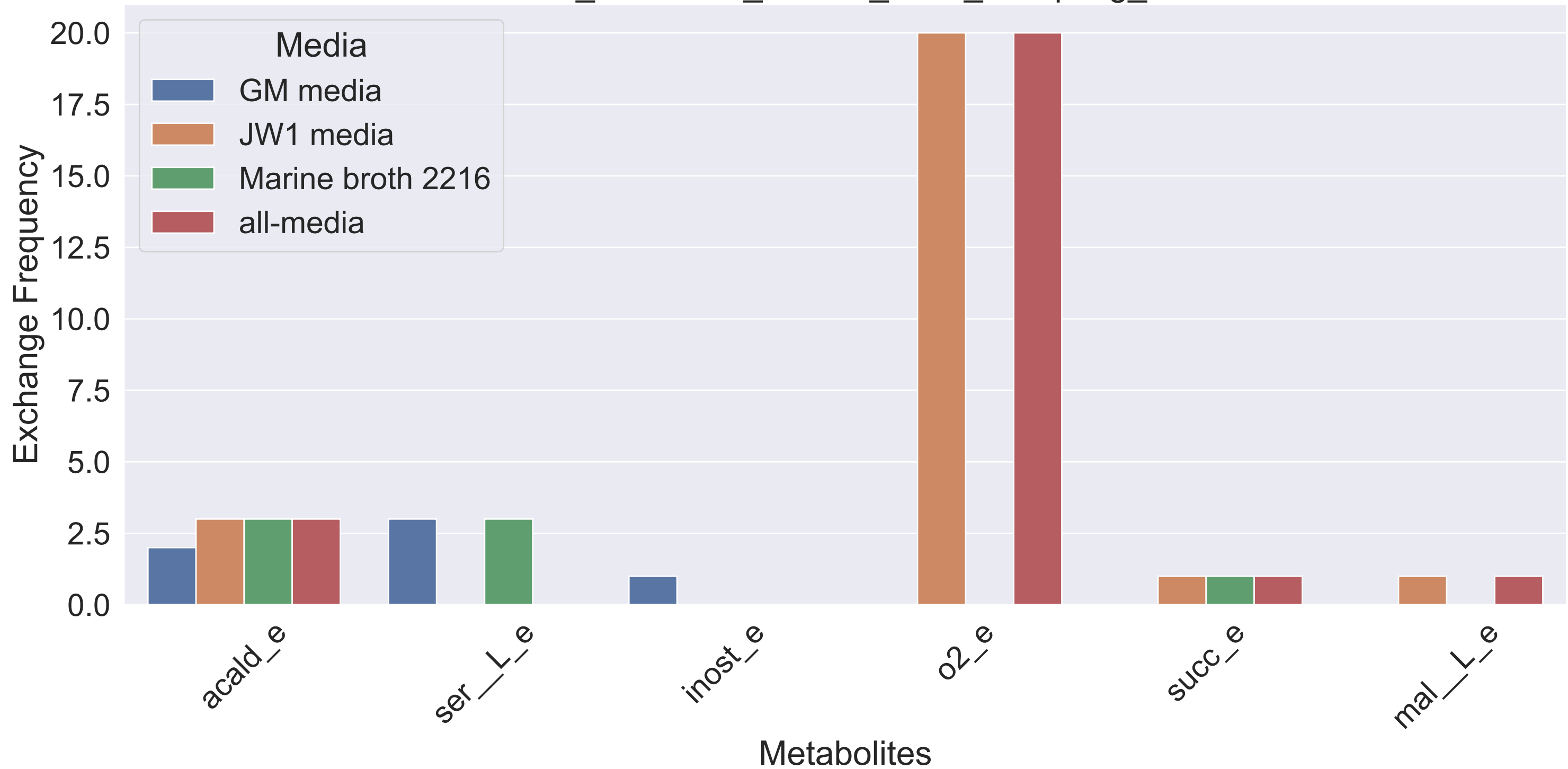

Supplement: Supplementary file 6 — Supplementary File S5 [file 43705_2023_242_MOESM6_ESM.zip › Supplementary Figure S4 Acidobacteria bacterium UWMA 0355 accepting metabolites.pdf]

# Gammaproteobacteria\_bacterium\_UWMA\_0260\_accepting\_metabolites

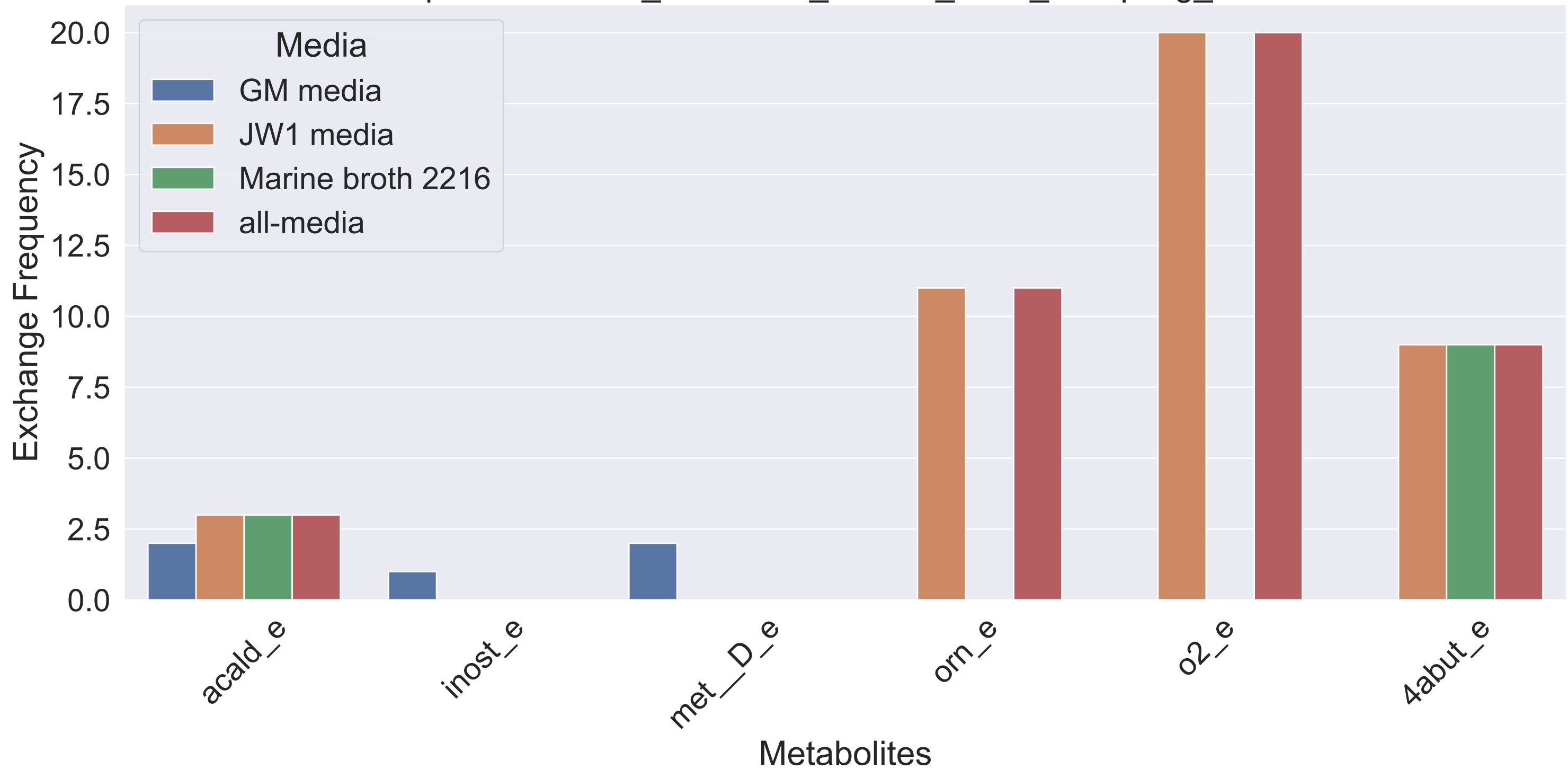

Supplement: Supplementary file 6 — Supplementary File S5 [file 43705_2023_242_MOESM6_ESM.zip › Supplementary Figure S40 Gammaproteobacteria bacterium UWMA 0260 accepting metabolites.pdf]

Gammaproteobacteria\_bacterium\_UWMA\_0261\_accepting\_metabolites

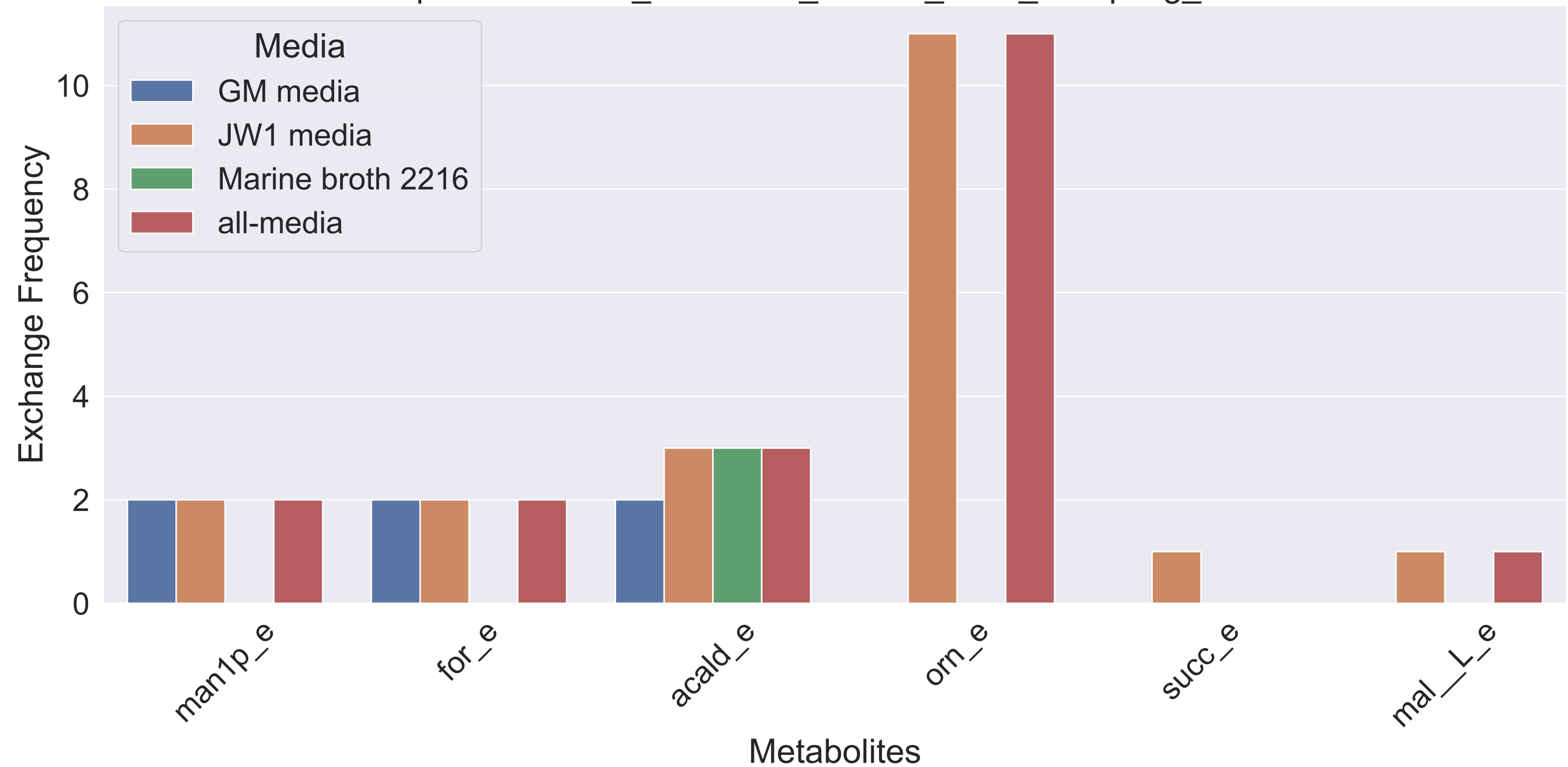

Supplement: Supplementary file 6 — Supplementary File S5 [file 43705_2023_242_MOESM6_ESM.zip › Supplementary Figure S41 Gammaproteobacteria bacterium UWMA 0261 accepting metabolites.pdf]

# Gammaproteobacteria\_bacterium\_UWMA\_0273\_accepting\_metabolites

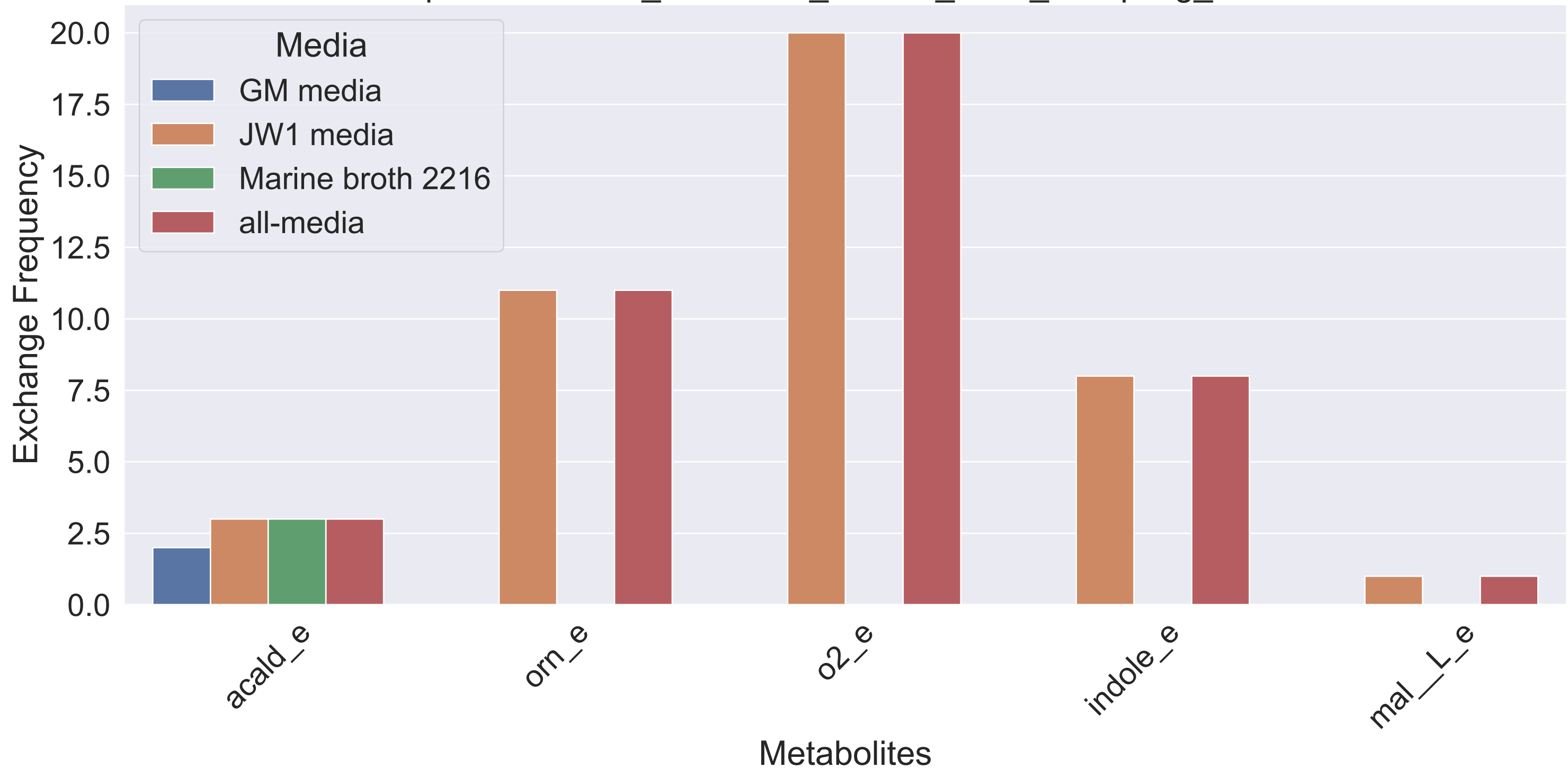

Supplement: Supplementary file 6 — Supplementary File S5 [file 43705_2023_242_MOESM6_ESM.zip › Supplementary Figure S42 Gammaproteobacteria bacterium UWMA 0273 accepting metabolites.pdf]

# Gammaproteobacteria\_bacterium\_UWMA\_0278\_accepting\_metabolites

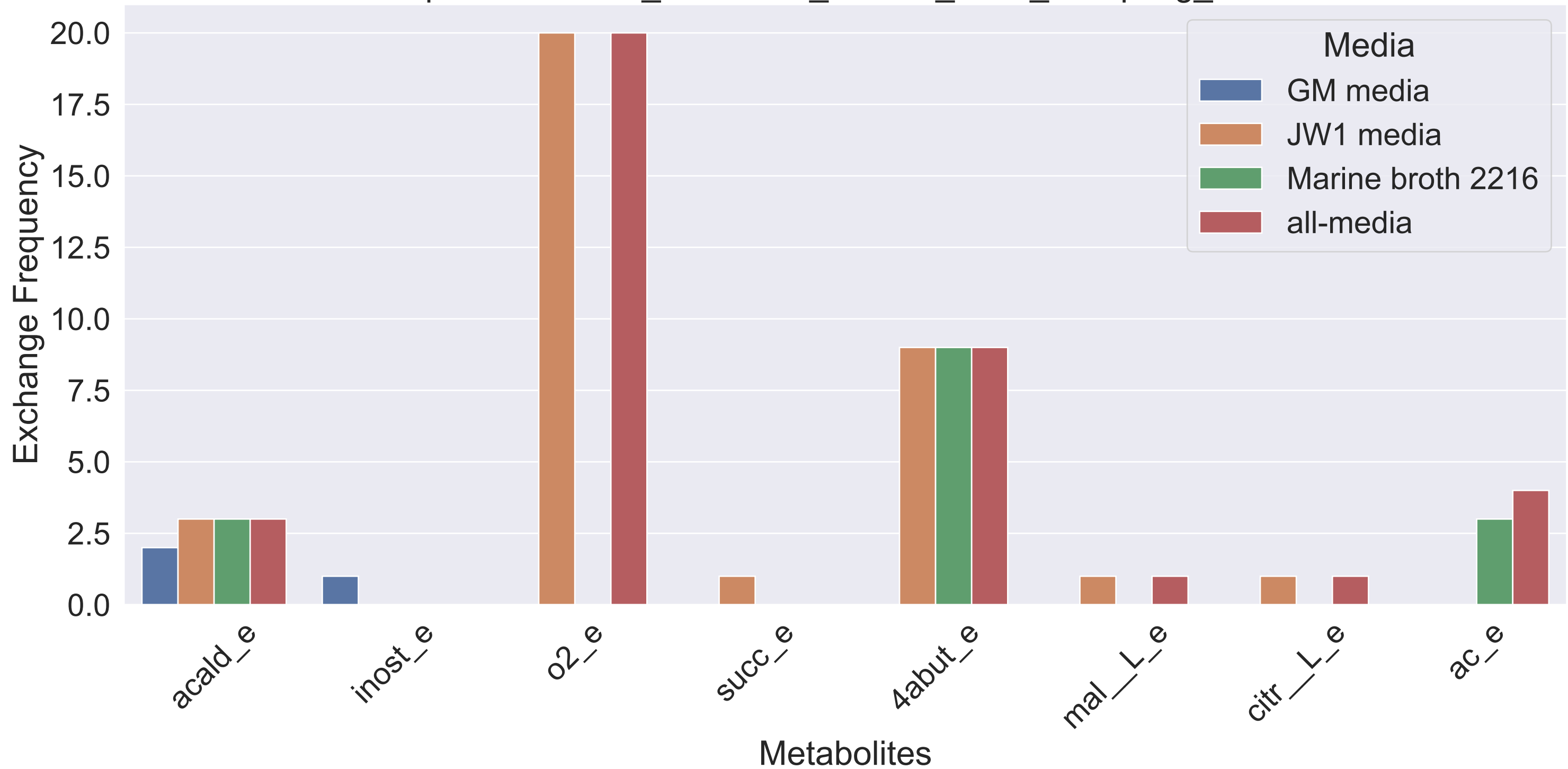

Supplement: Supplementary file 6 — Supplementary File S5 [file 43705_2023_242_MOESM6_ESM.zip › Supplementary Figure S43 Gammaproteobacteria bacterium UWMA 0278 accepting metabolites.pdf]

Gammaproteobacteria\_bacterium\_UWMA\_0281\_accepting\_metabolites

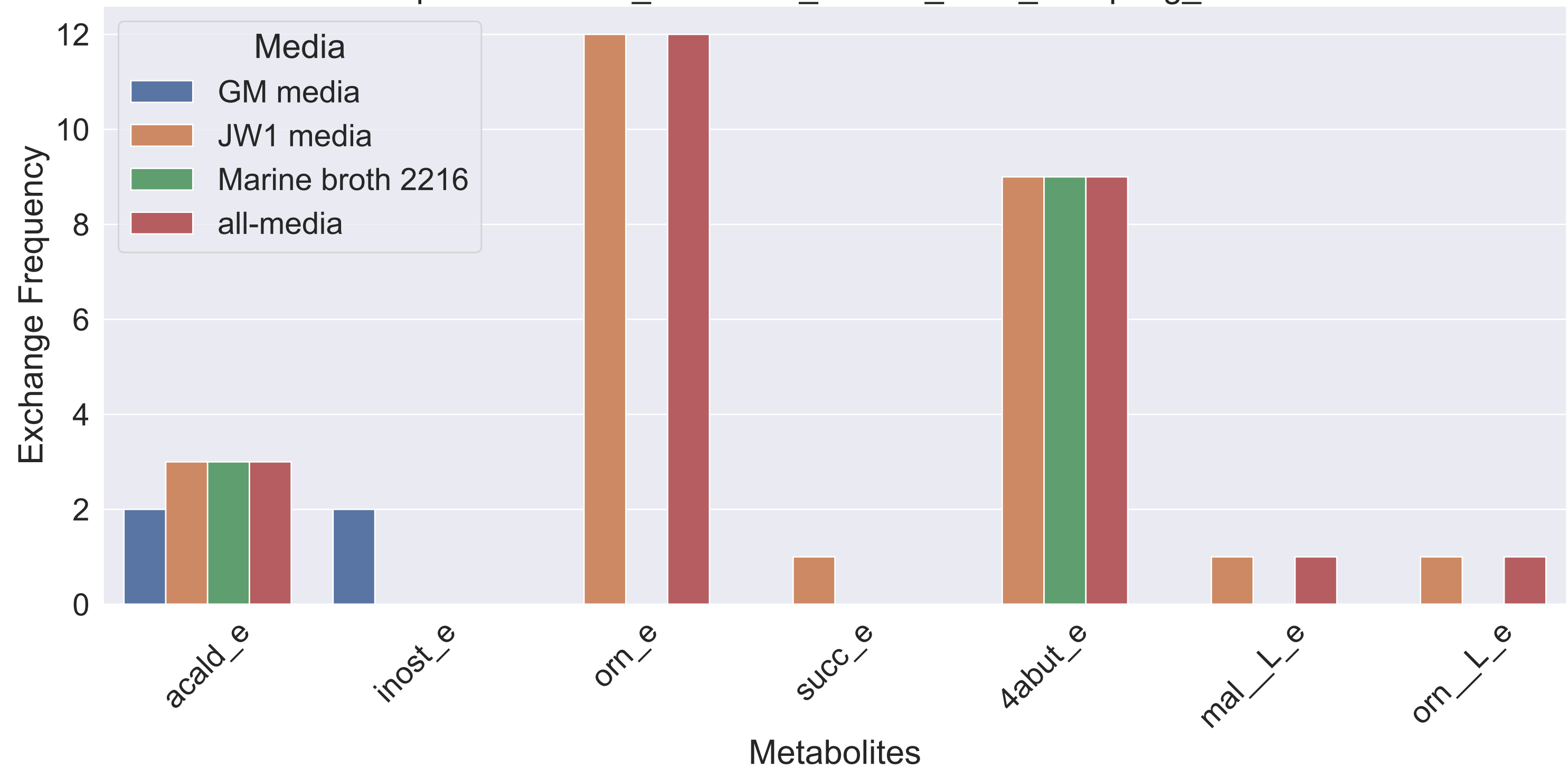

Supplement: Supplementary file 6 — Supplementary File S5 [file 43705_2023_242_MOESM6_ESM.zip › Supplementary Figure S44 Gammaproteobacteria bacterium UWMA 0281 accepting metabolites.pdf]

# Gammaproteobacteria\_bacterium\_UWMA\_0282\_accepting\_metabolites

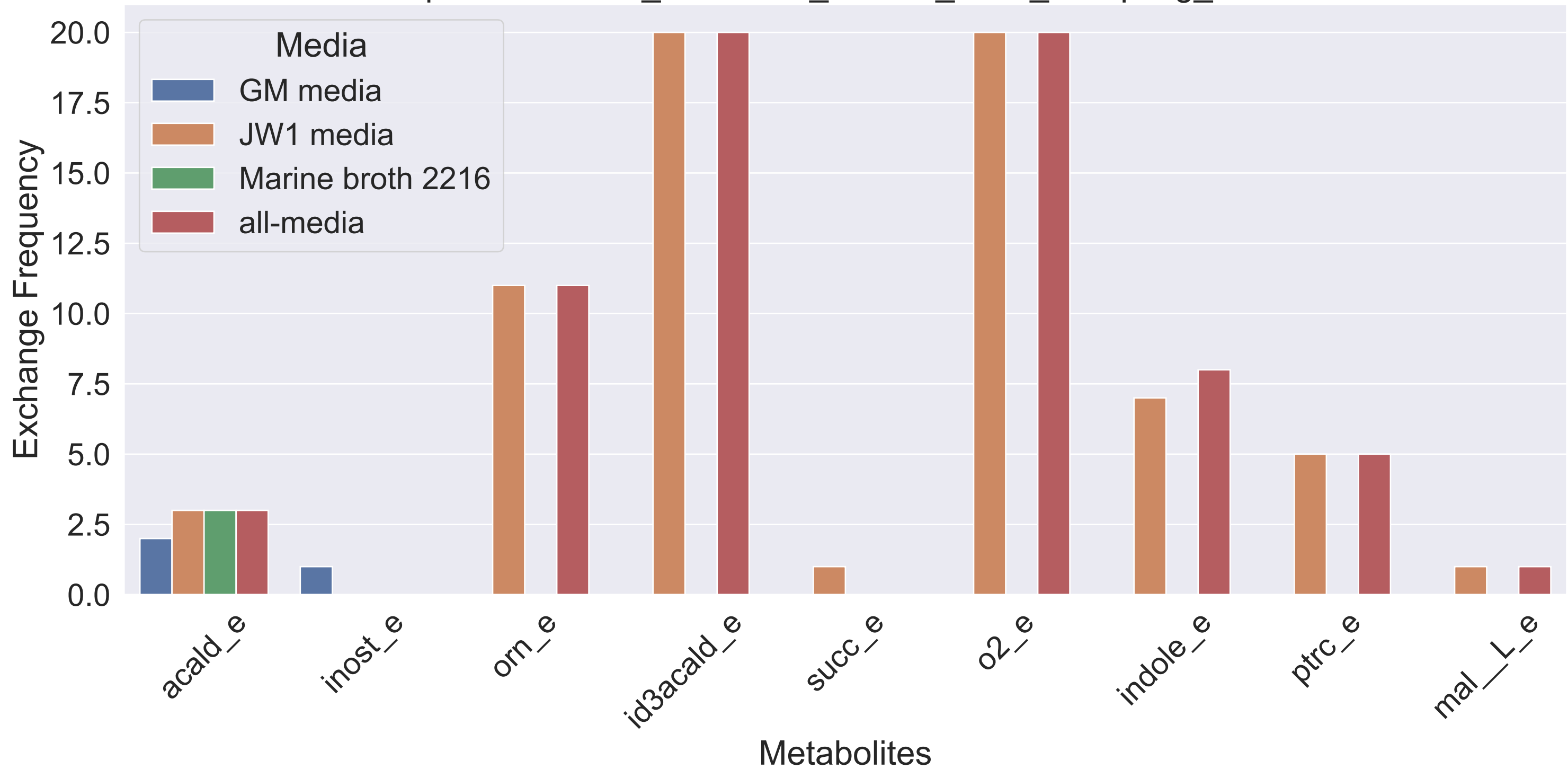

Supplement: Supplementary file 6 — Supplementary File S5 [file 43705_2023_242_MOESM6_ESM.zip › Supplementary Figure S45 Gammaproteobacteria bacterium UWMA 0282 accepting metabolites.pdf]

# Gammaproteobacteria\_bacterium\_UWMA\_0291\_accepting\_metabolites

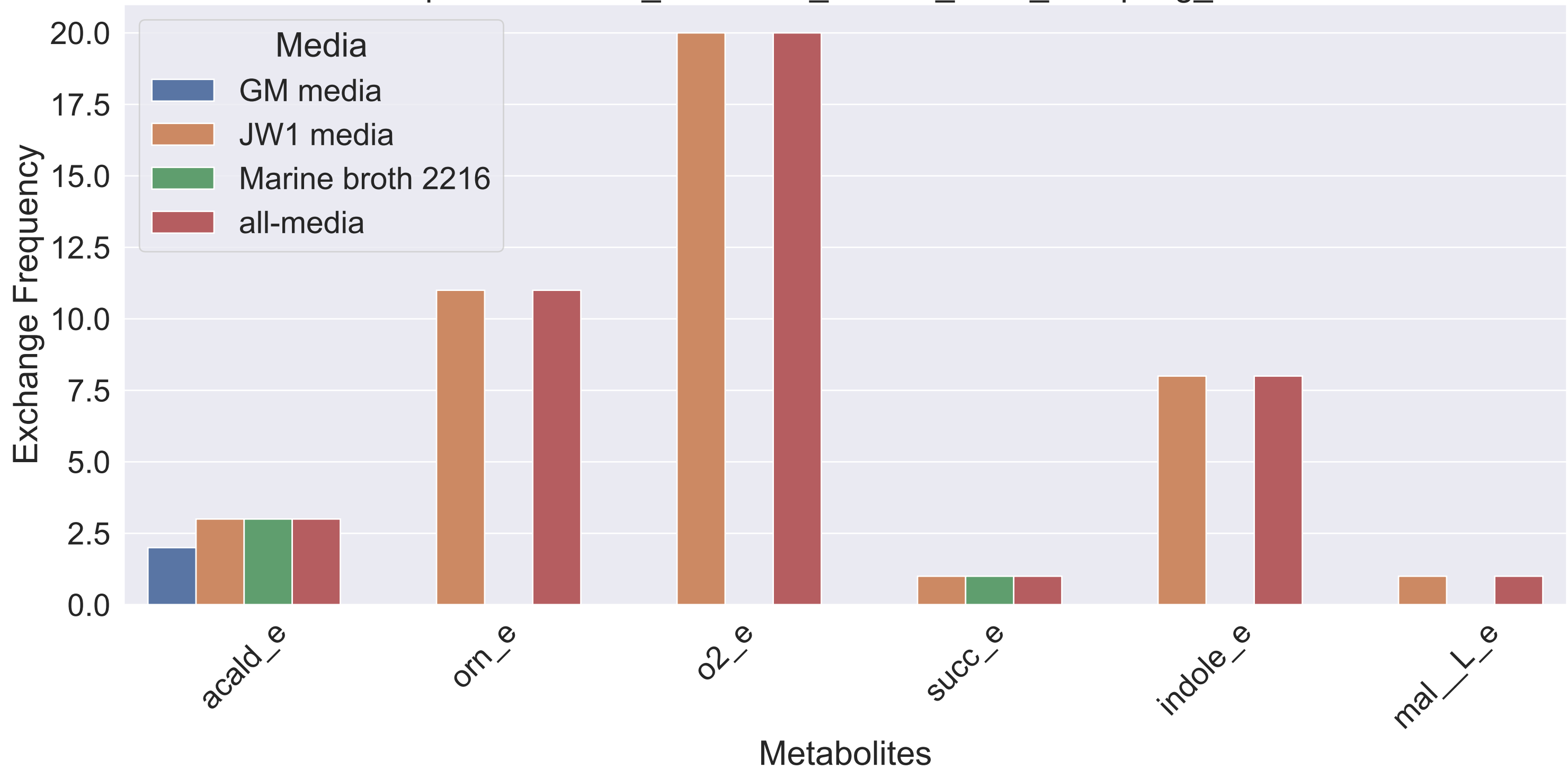

Supplement: Supplementary file 6 — Supplementary File S5 [file 43705_2023_242_MOESM6_ESM.zip › Supplementary Figure S46 Gammaproteobacteria bacterium UWMA 0291 accepting metabolites.pdf]

Gammaproteobacteria\_bacterium\_UWMA\_0299\_accepting\_metabolites

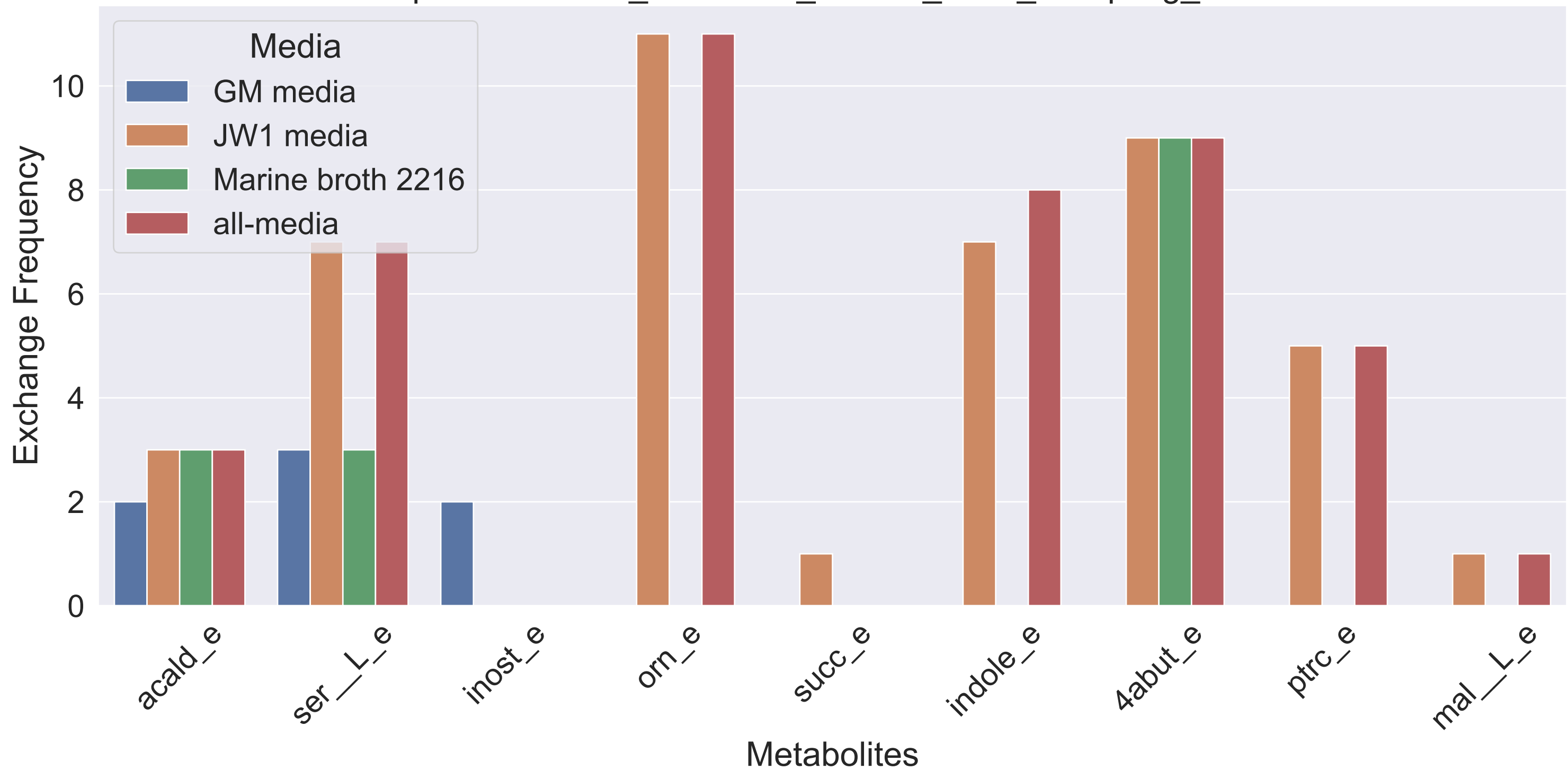

Supplement: Supplementary file 6 — Supplementary File S5 [file 43705_2023_242_MOESM6_ESM.zip › Supplementary Figure S47 Gammaproteobacteria bacterium UWMA 0299 accepting metabolites.pdf]

# Gammaproteobacteria\_bacterium\_UWMA\_0301\_accepting\_metabolites

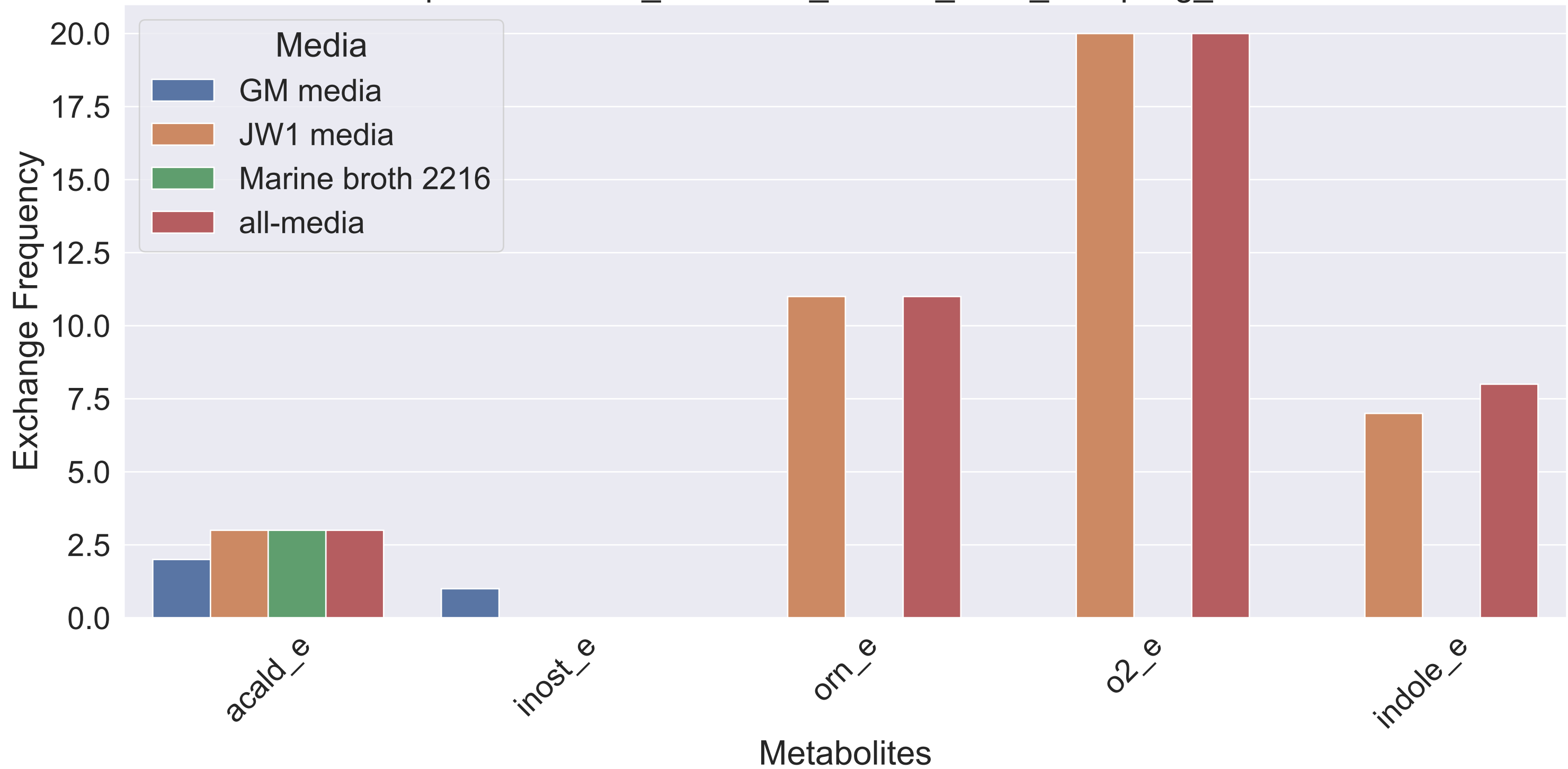

Supplement: Supplementary file 6 — Supplementary File S5 [file 43705_2023_242_MOESM6_ESM.zip › Supplementary Figure S48 Gammaproteobacteria bacterium UWMA 0301 accepting metabolites.pdf]

Gammaproteobacteria\_bacterium\_UWMA\_0302\_accepting\_metabolites

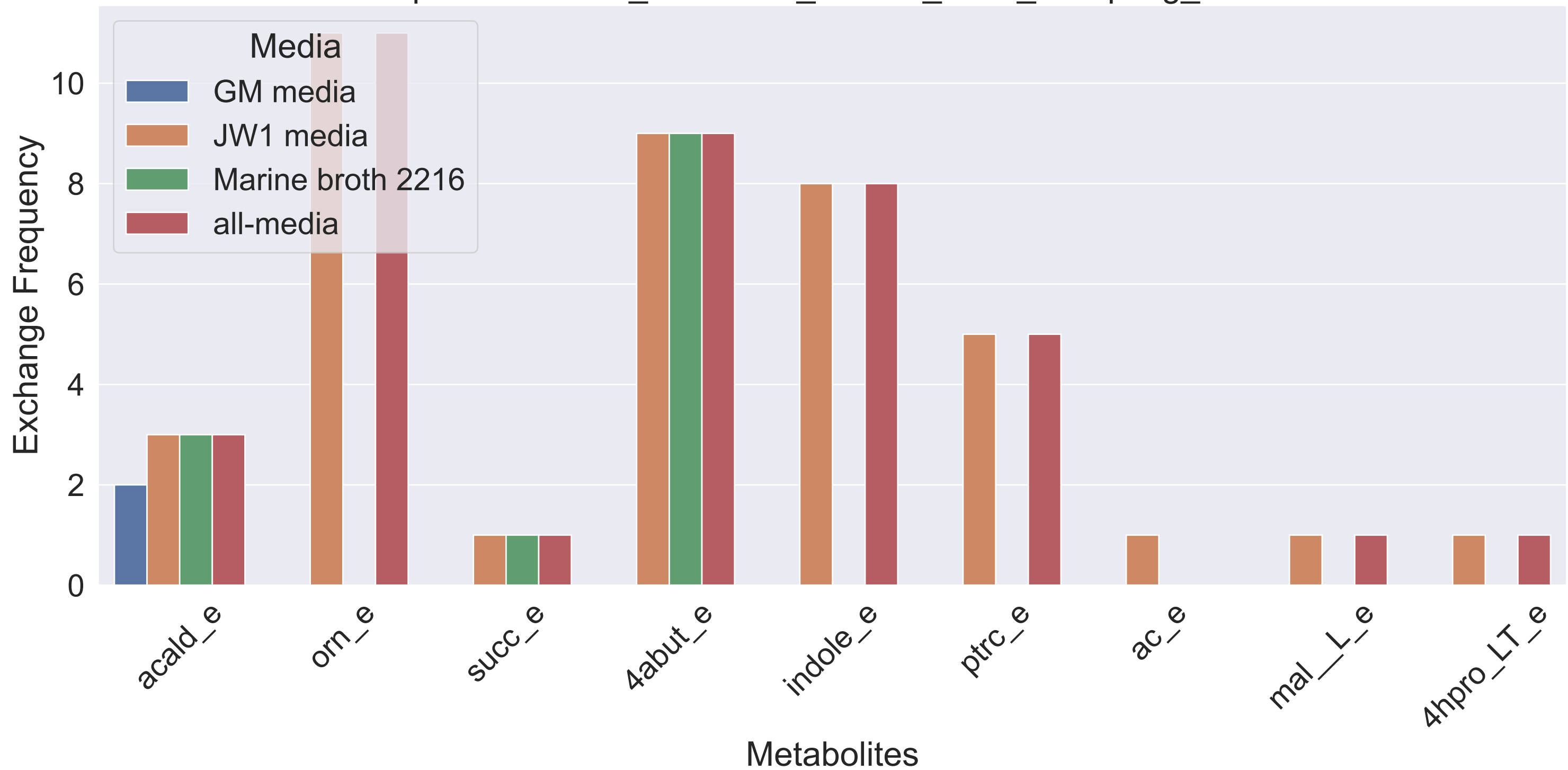

Supplement: Supplementary file 6 — Supplementary File S5 [file 43705_2023_242_MOESM6_ESM.zip › Supplementary Figure S49 Gammaproteobacteria bacterium UWMA 0302 accepting metabolites.pdf]

# Alphaproteobacteria\_bacterium\_UWMA\_0321\_accepting\_metabolites

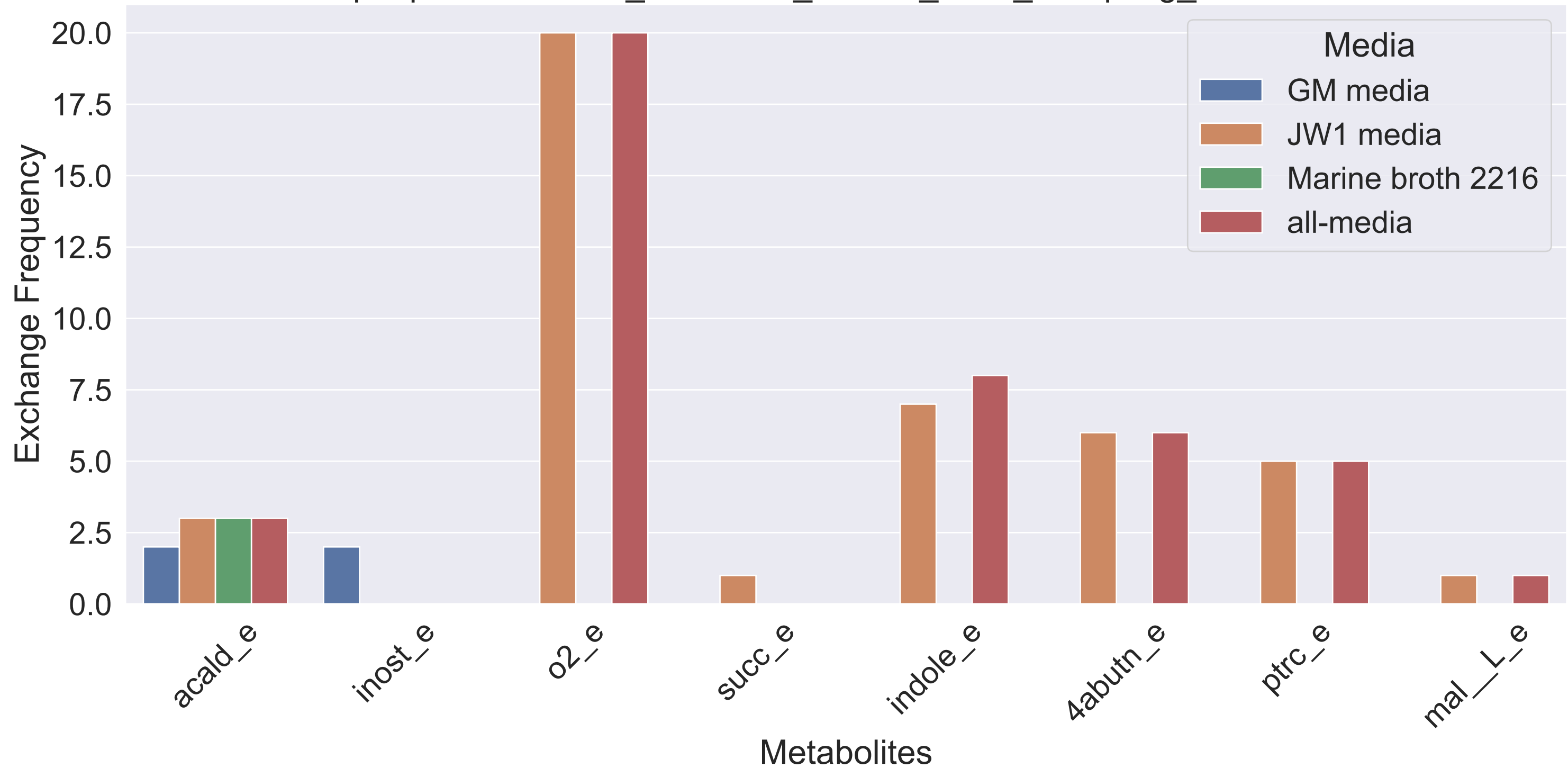

Supplement: Supplementary file 6 — Supplementary File S5 [file 43705_2023_242_MOESM6_ESM.zip › Supplementary Figure S5 Alphaproteobacteria bacterium UWMA 0321 accepting metabolites.pdf]

Gammaproteobacteria\_bacterium\_UWMA\_0343\_accepting\_metabolites

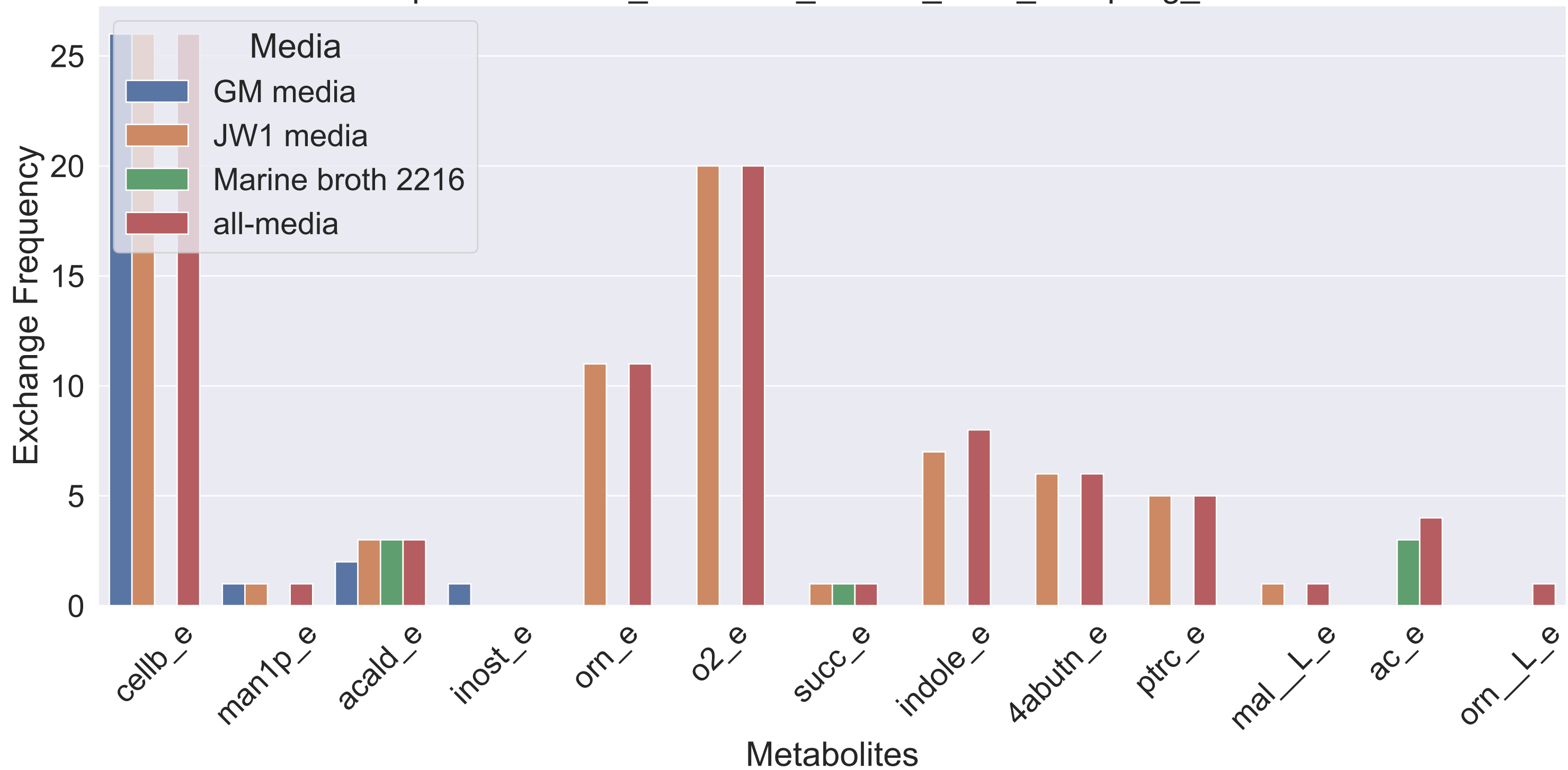

Supplement: Supplementary file 6 — Supplementary File S5 [file 43705_2023_242_MOESM6_ESM.zip › Supplementary Figure S50 Gammaproteobacteria bacterium UWMA 0343 accepting metabolites.pdf]

# Gemmatimonadetes\_bacterium\_UWMA\_0296\_accepting\_metabolites

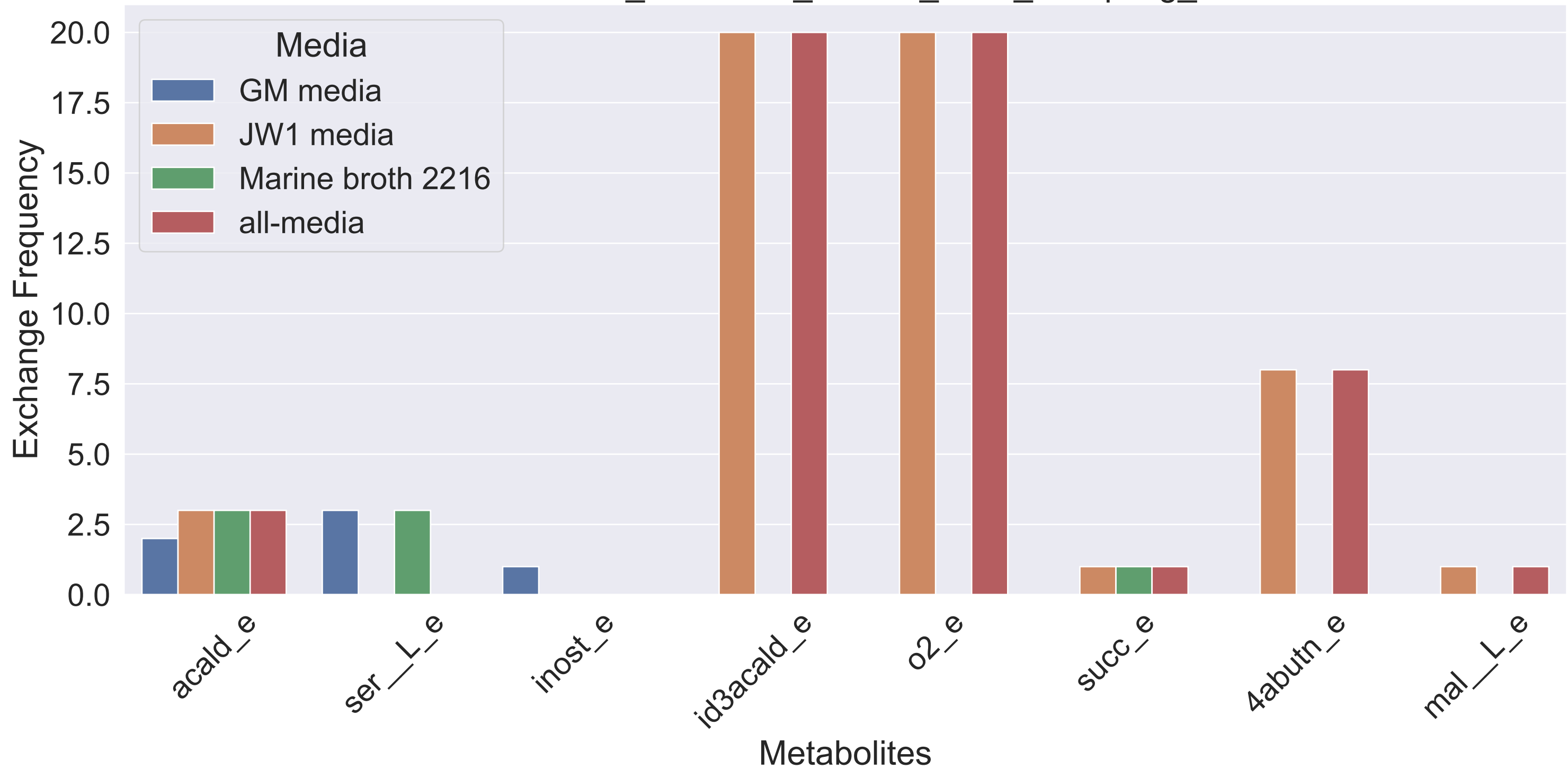

Supplement: Supplementary file 6 — Supplementary File S5 [file 43705_2023_242_MOESM6_ESM.zip › Supplementary Figure S51 Gemmatimonadetes bacterium UWMA 0296 accepting metabolites.pdf]

# Gemmatimonadetes\_bacterium\_UWMA\_0308\_accepting\_metabolites

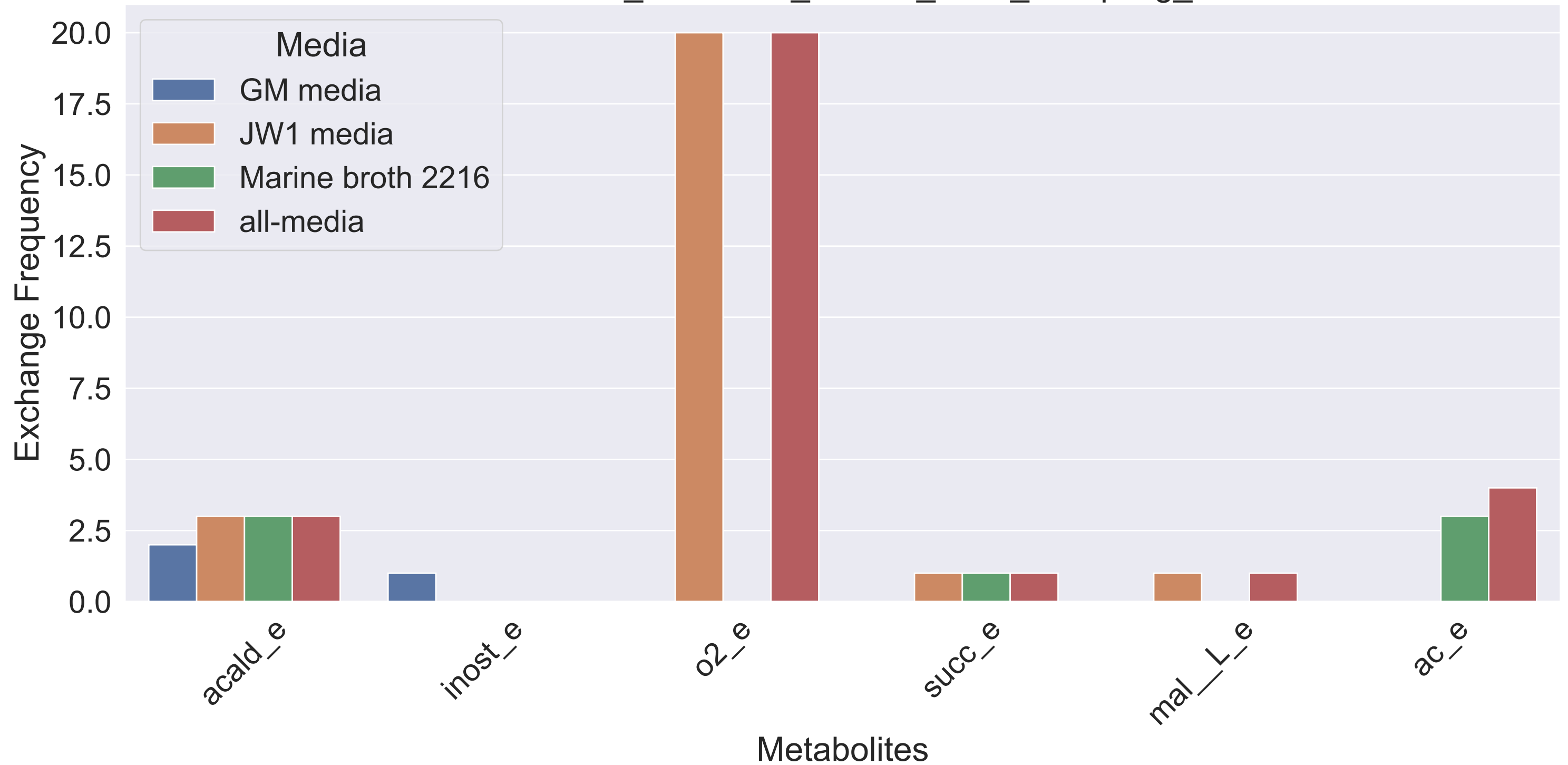

Supplement: Supplementary file 6 — Supplementary File S5 [file 43705_2023_242_MOESM6_ESM.zip › Supplementary Figure S52 Gemmatimonadetes bacterium UWMA 0308 accepting metabolites.pdf]

# Gemmatimonadetes\_bacterium\_UWMA\_0334\_accepting\_metabolites

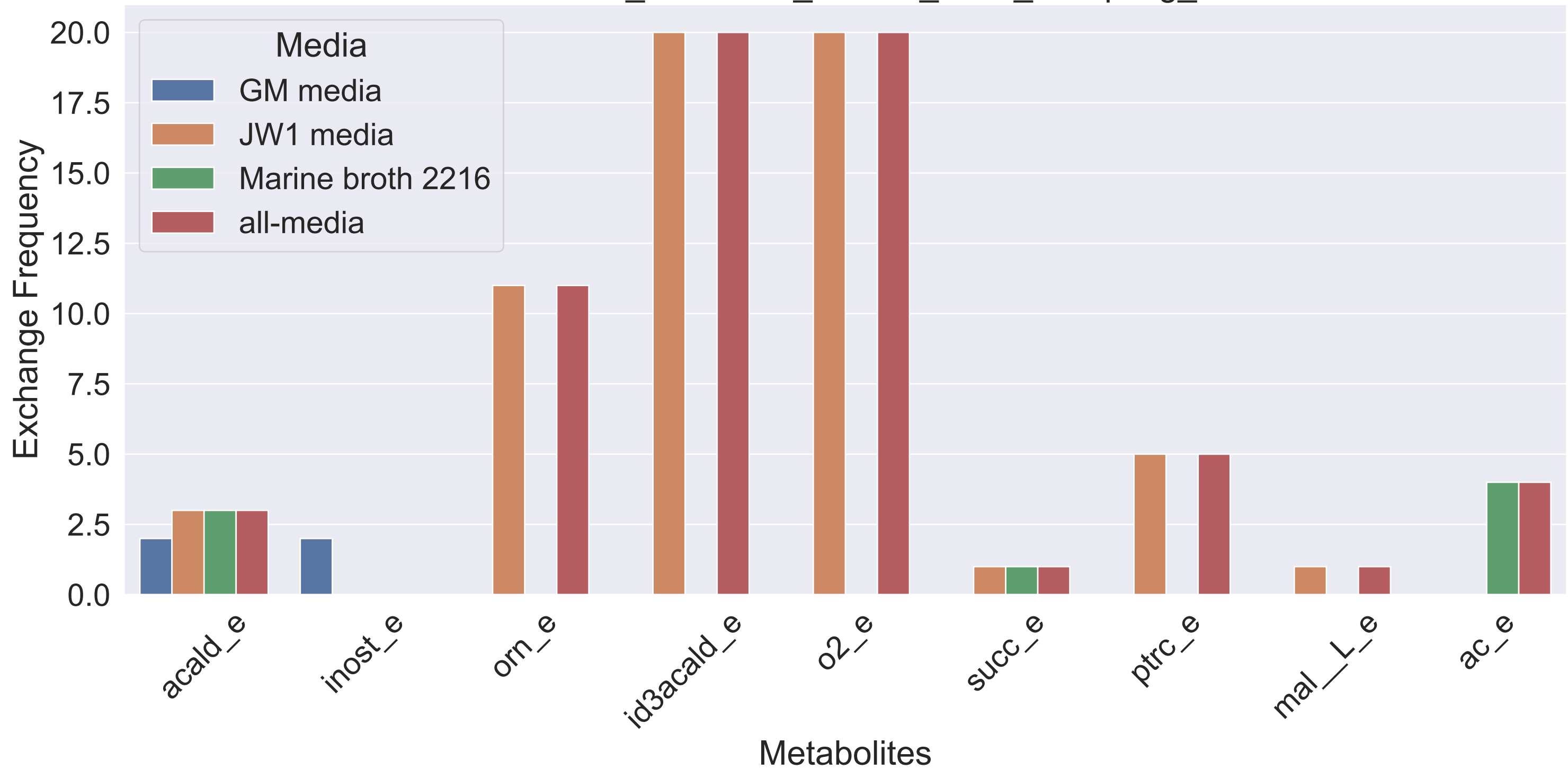

Supplement: Supplementary file 6 — Supplementary File S5 [file 43705_2023_242_MOESM6_ESM.zip › Supplementary Figure S53 Gemmatimonadetes bacterium UWMA 0334 accepting metabolites.pdf]

Gemmatimonadetes\_bacterium\_UWMA\_0339\_accepting\_metabolites

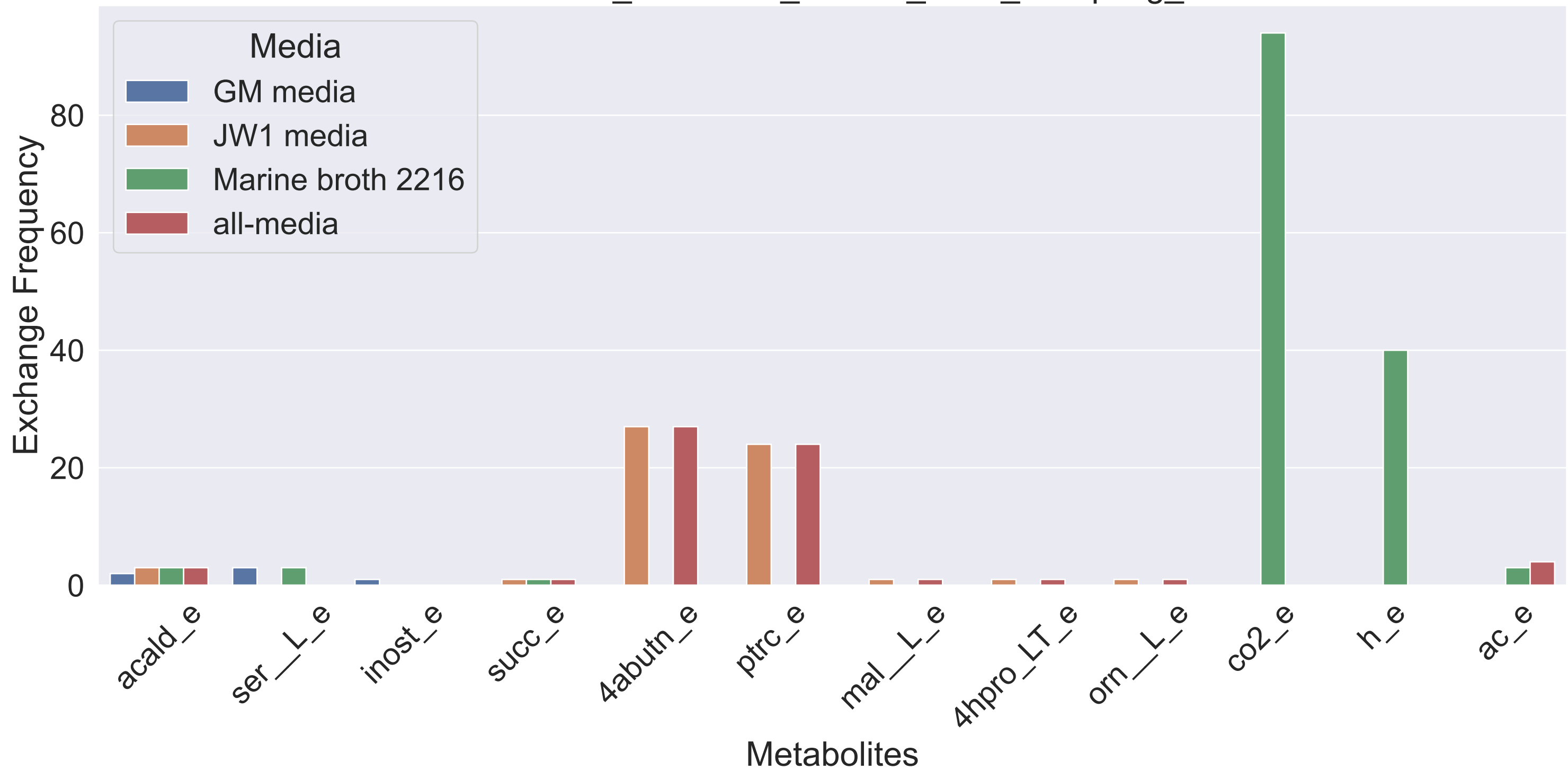

Supplement: Supplementary file 6 — Supplementary File S5 [file 43705_2023_242_MOESM6_ESM.zip › Supplementary Figure S54 Gemmatimonadetes bacterium UWMA 0339 accepting metabolites.pdf]

# Henriciella\_sp\_UWMA\_0297\_accepting\_metabolites

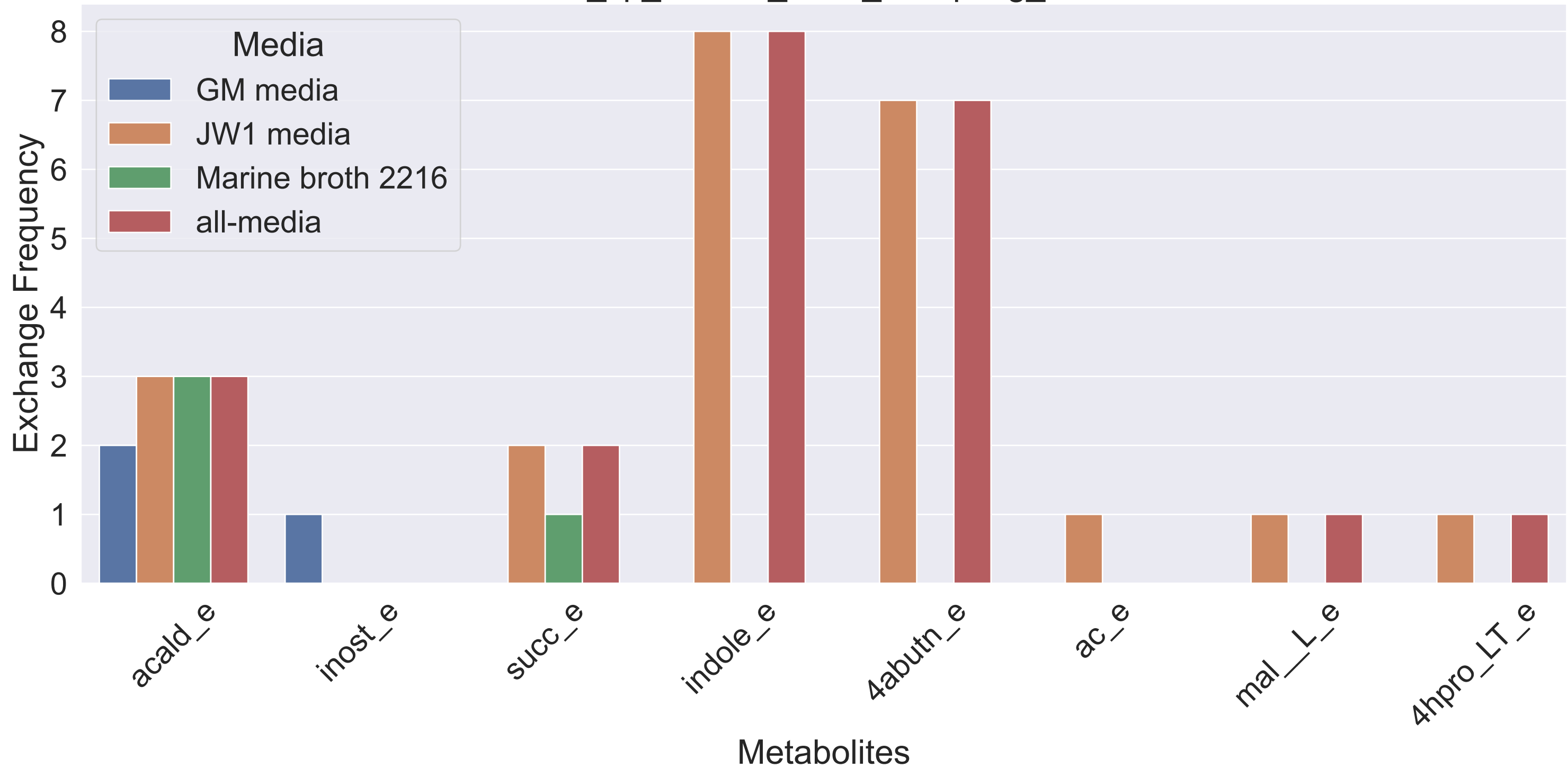

Supplement: Supplementary file 6 — Supplementary File S5 [file 43705_2023_242_MOESM6_ESM.zip › Supplementary Figure S55 Henriciella sp UWMA 0297 accepting metabolites.pdf]

Marine\_Group\_III\_euryarchaeote\_UWMA\_0284\_accepting\_metabolites

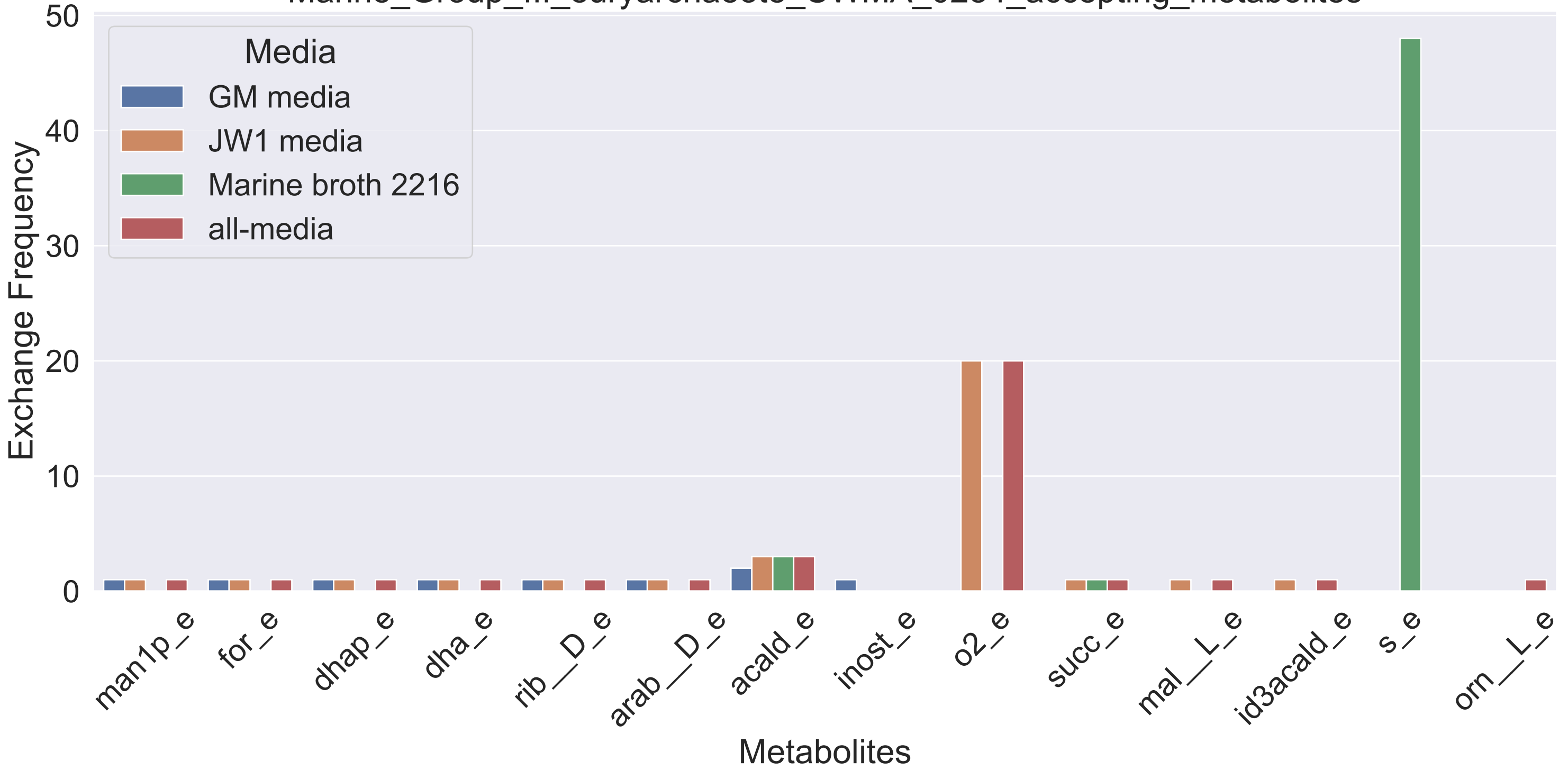

Supplement: Supplementary file 6 — Supplementary File S5 [file 43705_2023_242_MOESM6_ESM.zip › Supplementary Figure S56 Marine Group III euryarchaeote UWMA 0284 accepting metabolites.pdf]

Marine\_Group\_III\_euryarchaeote\_UWMA\_0340\_accepting\_metabolites

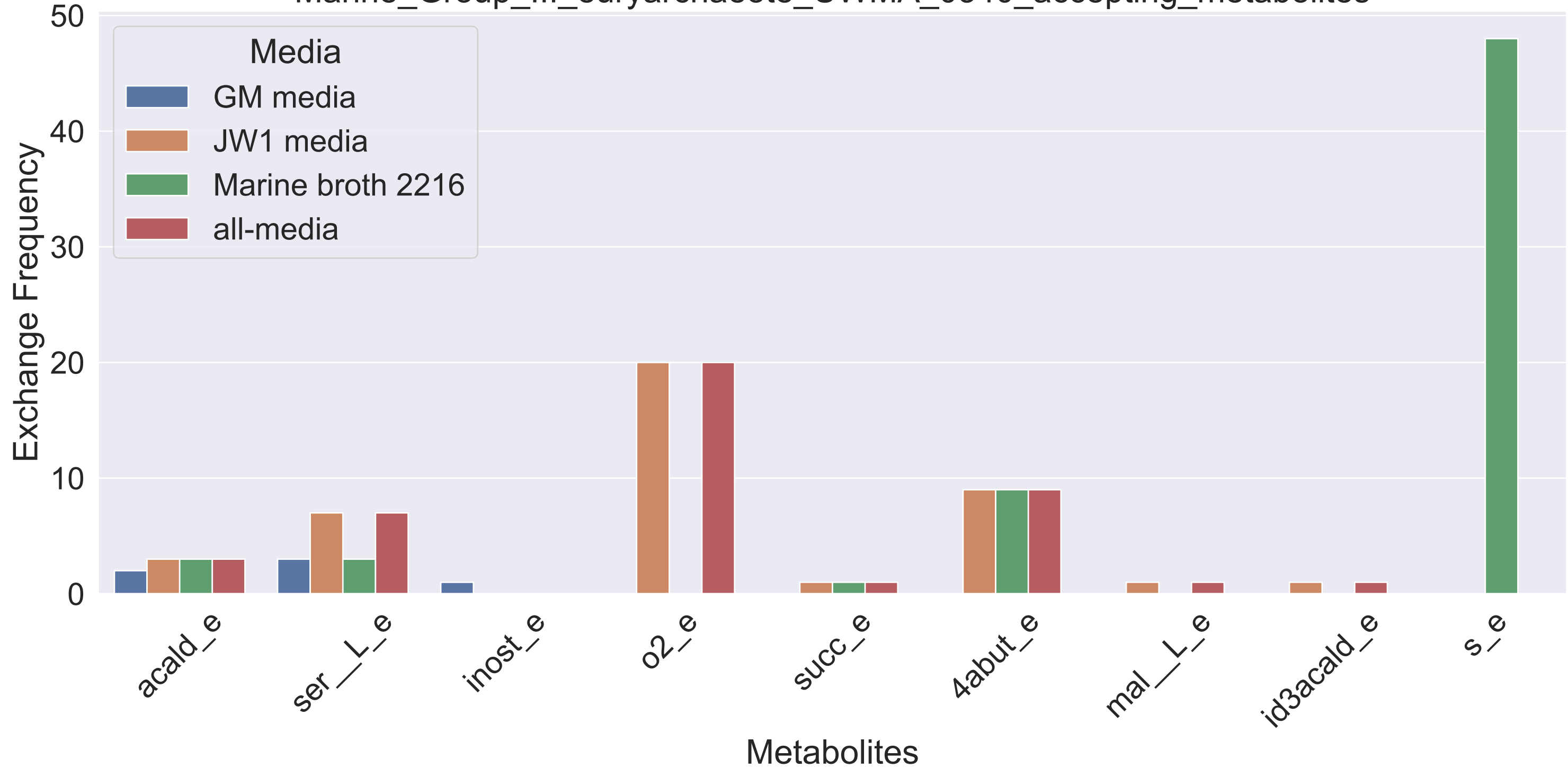

Supplement: Supplementary file 6 — Supplementary File S5 [file 43705_2023_242_MOESM6_ESM.zip › Supplementary Figure S57 Marine Group III euryarchaeote UWMA 0340 accepting metabolites.pdf]

Marine\_Group\_II\_euryarchaeote\_UWMA\_0266\_accepting\_metabolites

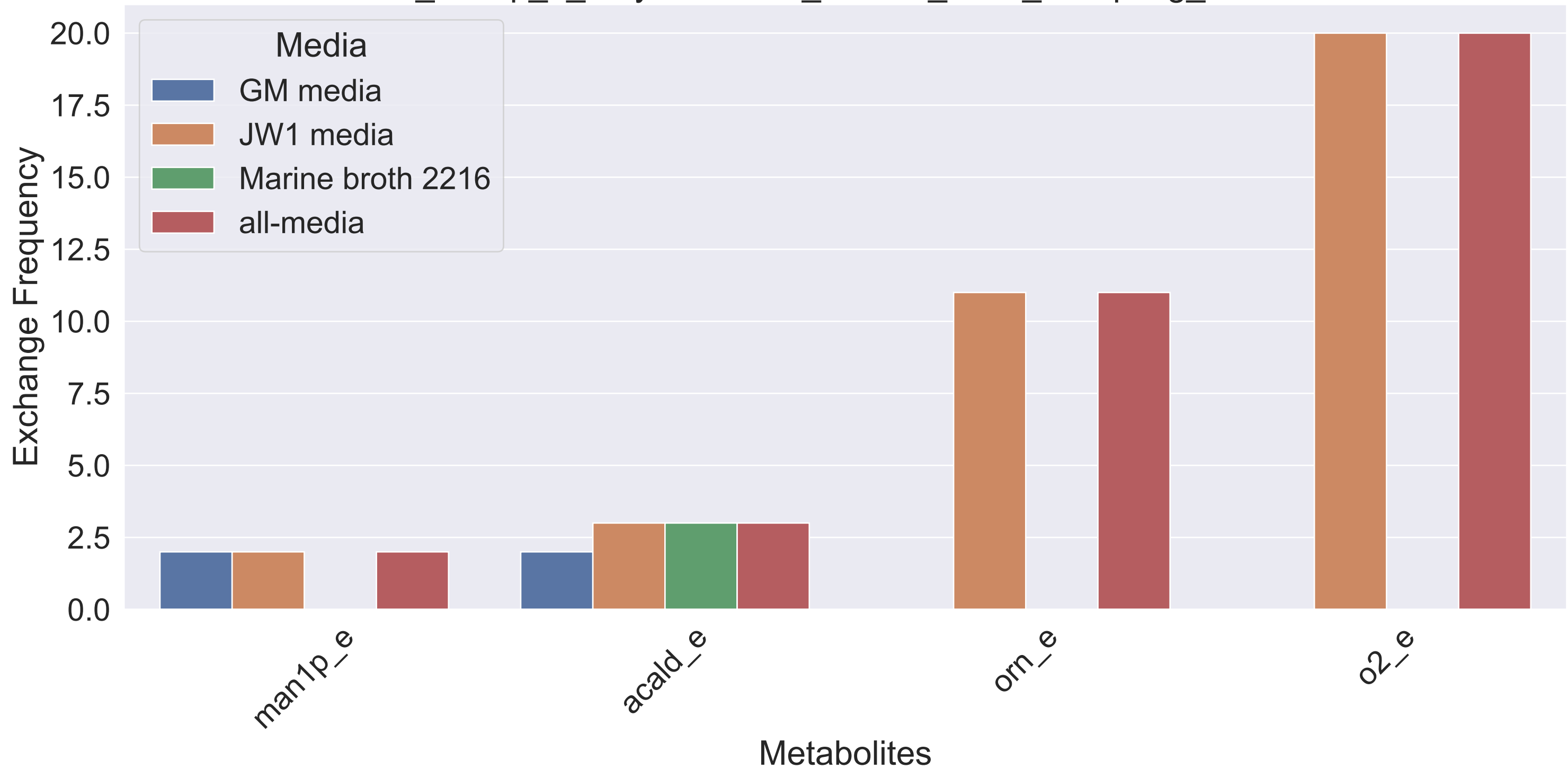

Supplement: Supplementary file 6 — Supplementary File S5 [file 43705_2023_242_MOESM6_ESM.zip › Supplementary Figure S58 Marine Group II euryarchaeote UWMA 0266 accepting metabolites.pdf]

Marine\_Group\_II\_euryarchaeote\_UWMA\_0275\_accepting\_metabolites

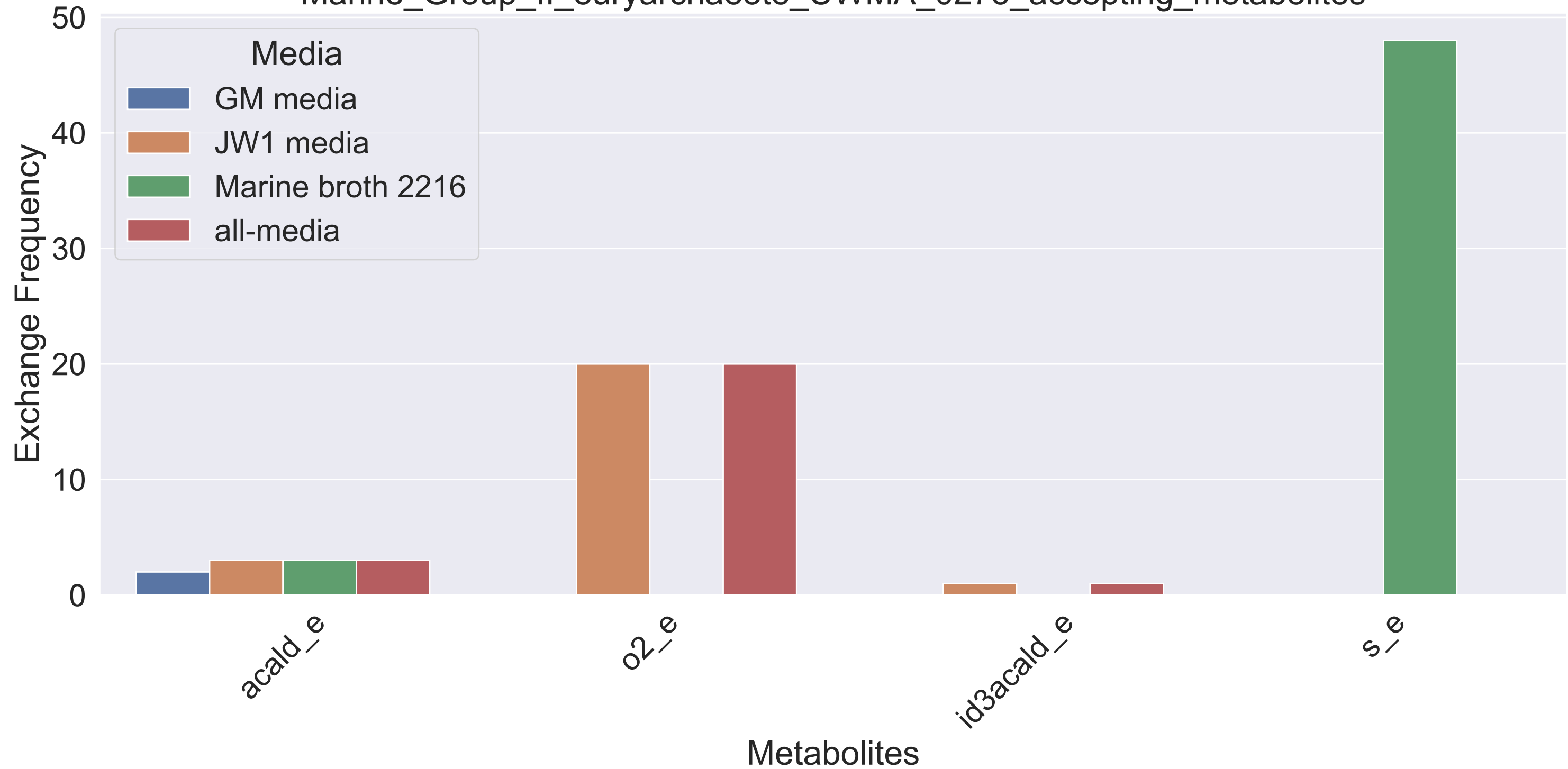

Supplement: Supplementary file 6 — Supplementary File S5 [file 43705_2023_242_MOESM6_ESM.zip › Supplementary Figure S59 Marine Group II euryarchaeote UWMA 0275 accepting metabolites.pdf]

# Bacteroidetes\_bacterium\_UWMA\_0293\_accepting\_metabolites

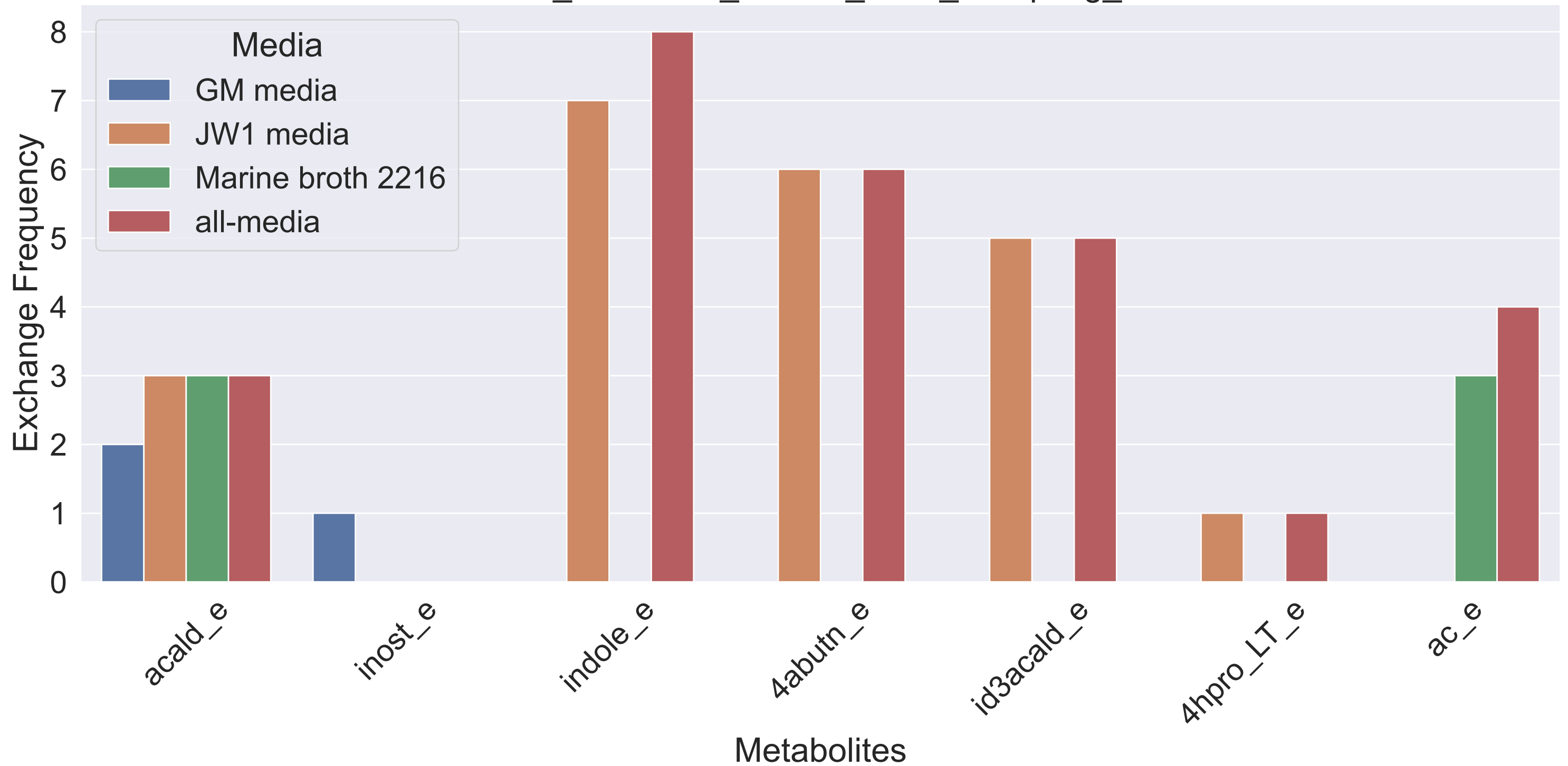

Supplement: Supplementary file 6 — Supplementary File S5 [file 43705_2023_242_MOESM6_ESM.zip › Supplementary Figure S6 Bacteroidetes bacterium UWMA 0293 accepting metabolites.pdf]

Marine\_Group\_II\_euryarchaeote\_UWMA\_0279\_accepting\_metabolites

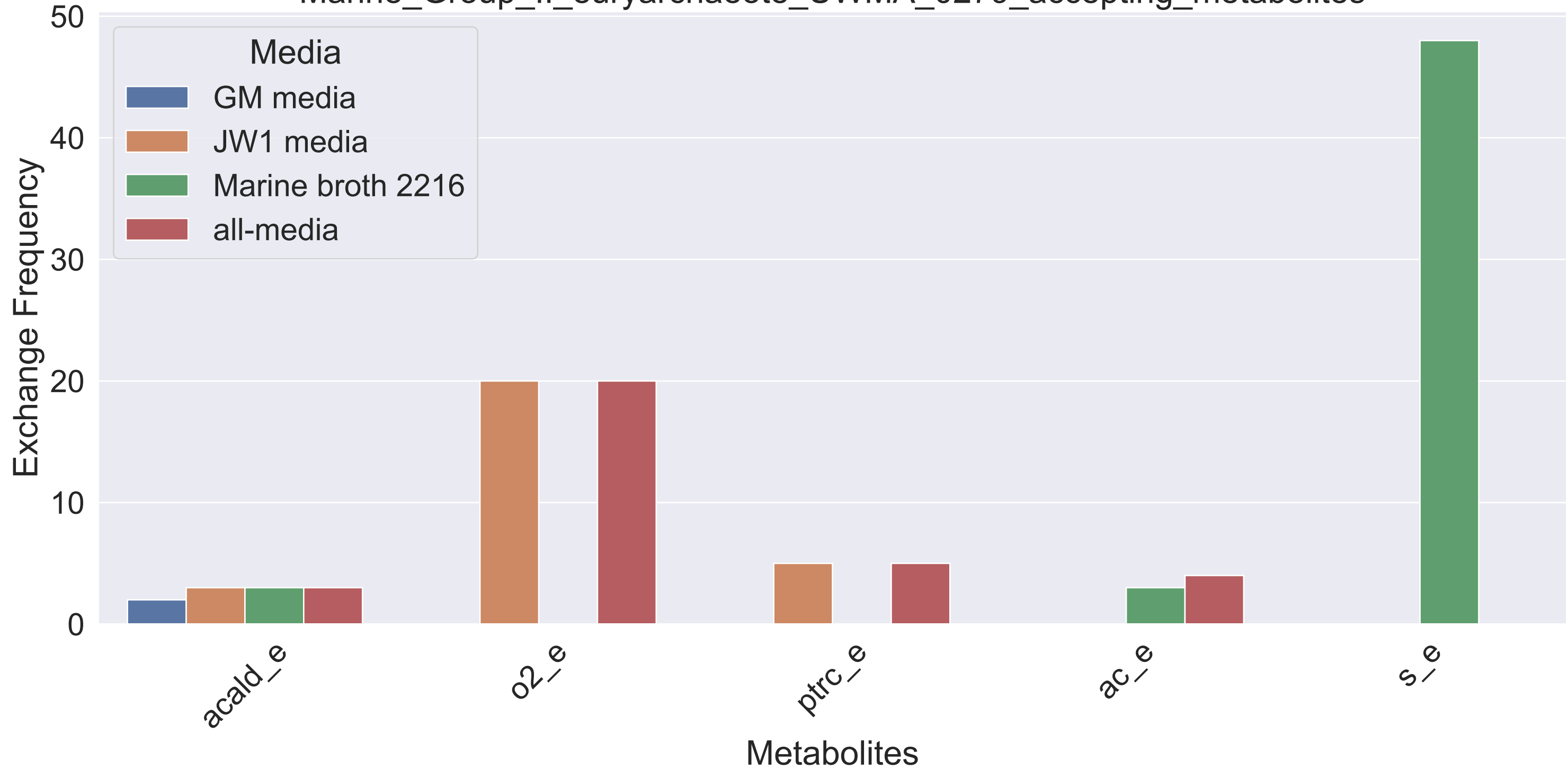

Supplement: Supplementary file 6 — Supplementary File S5 [file 43705_2023_242_MOESM6_ESM.zip › Supplementary Figure S60 Marine Group II euryarchaeote UWMA 0279 accepting metabolites.pdf]

Marine\_Group\_II\_euryarchaeote\_UWMA\_0283\_accepting\_metabolites

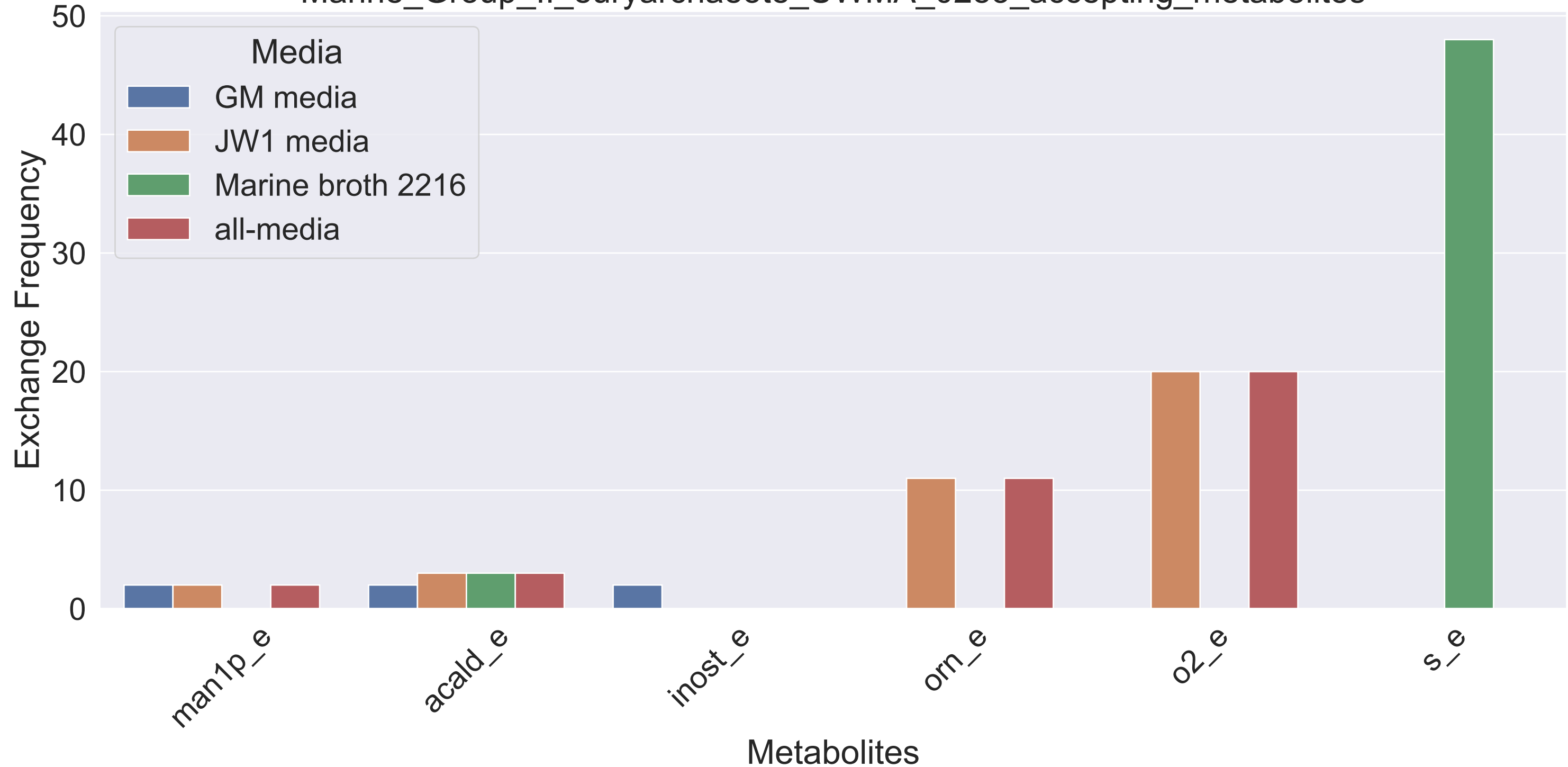

Supplement: Supplementary file 6 — Supplementary File S5 [file 43705_2023_242_MOESM6_ESM.zip › Supplementary Figure S61 Marine Group II euryarchaeote UWMA 0283 accepting metabolites.pdf]

Marine\_Group\_II\_euryarchaeote\_UWMA\_0323\_accepting\_metabolites

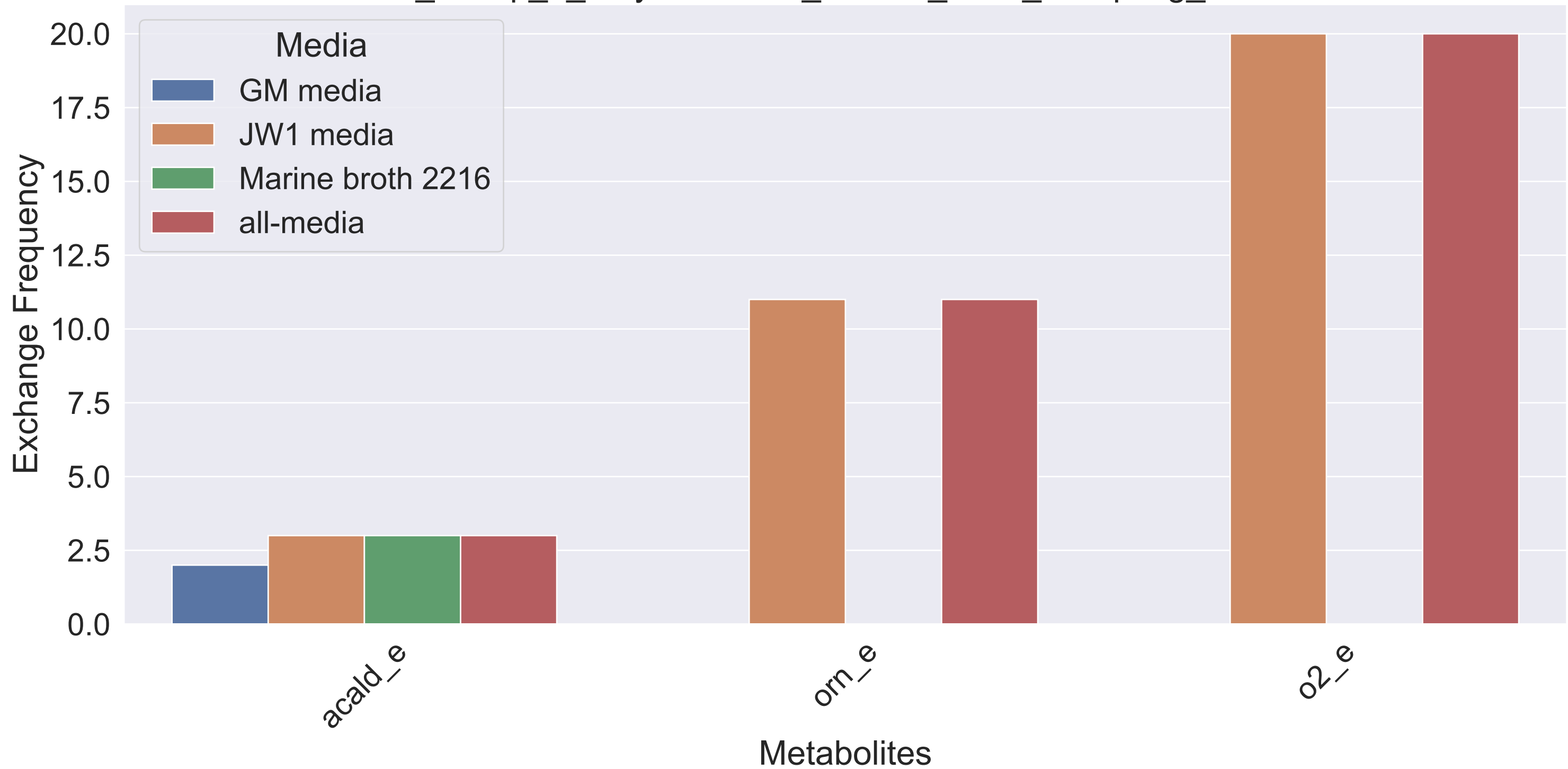

Supplement: Supplementary file 6 — Supplementary File S5 [file 43705_2023_242_MOESM6_ESM.zip › Supplementary Figure S62 Marine Group II euryarchaeote UWMA 0323 accepting metabolites.pdf]

Marine\_Group\_II\_euryarchaeote\_UWMA\_0328\_accepting\_metabolites

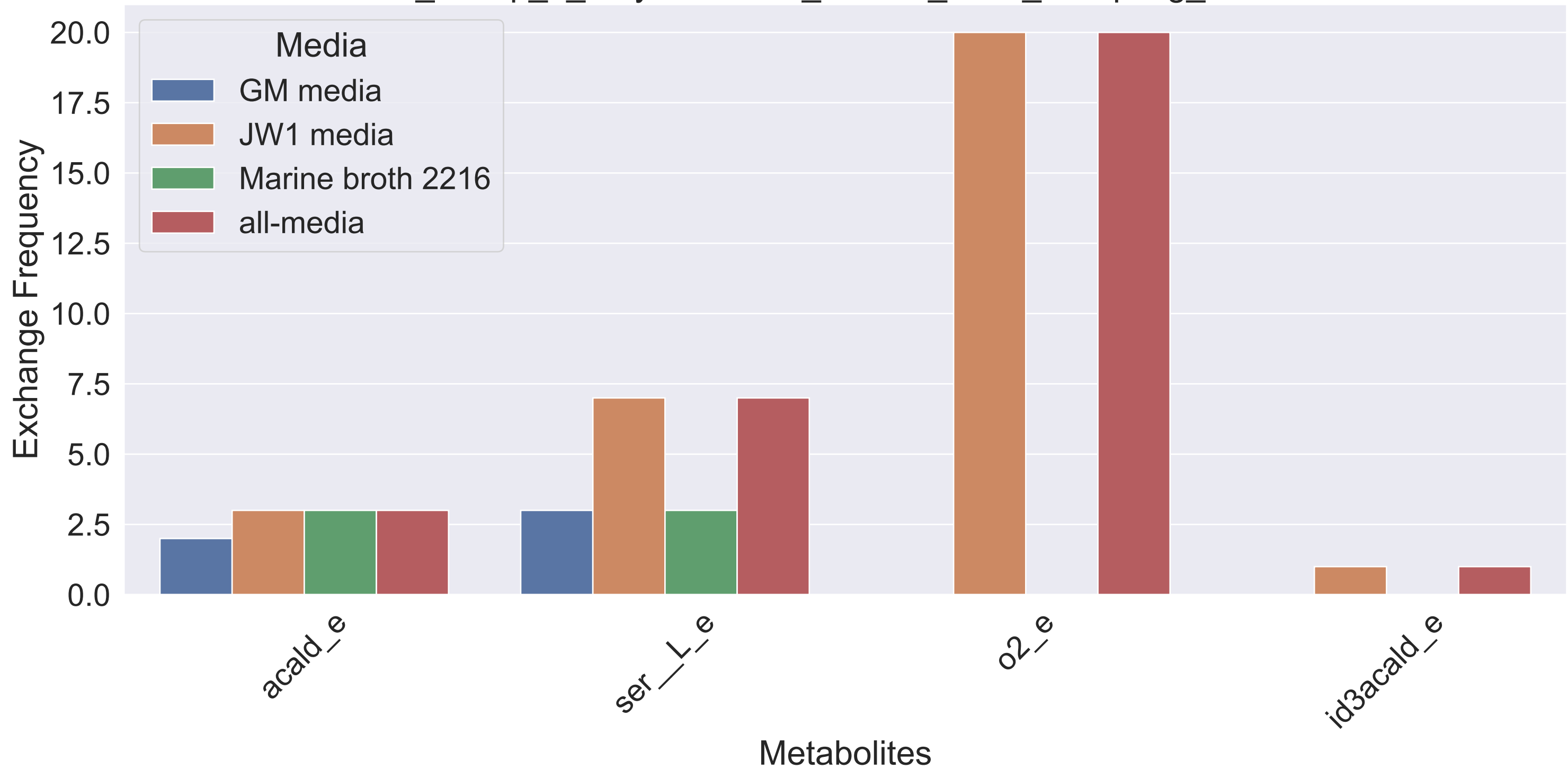

Supplement: Supplementary file 6 — Supplementary File S5 [file 43705_2023_242_MOESM6_ESM.zip › Supplementary Figure S63 Marine Group II euryarchaeote UWMA 0328 accepting metabolites.pdf]

Marine\_Group\_II\_euryarchaeote\_UWMA\_0344\_accepting\_metabolites

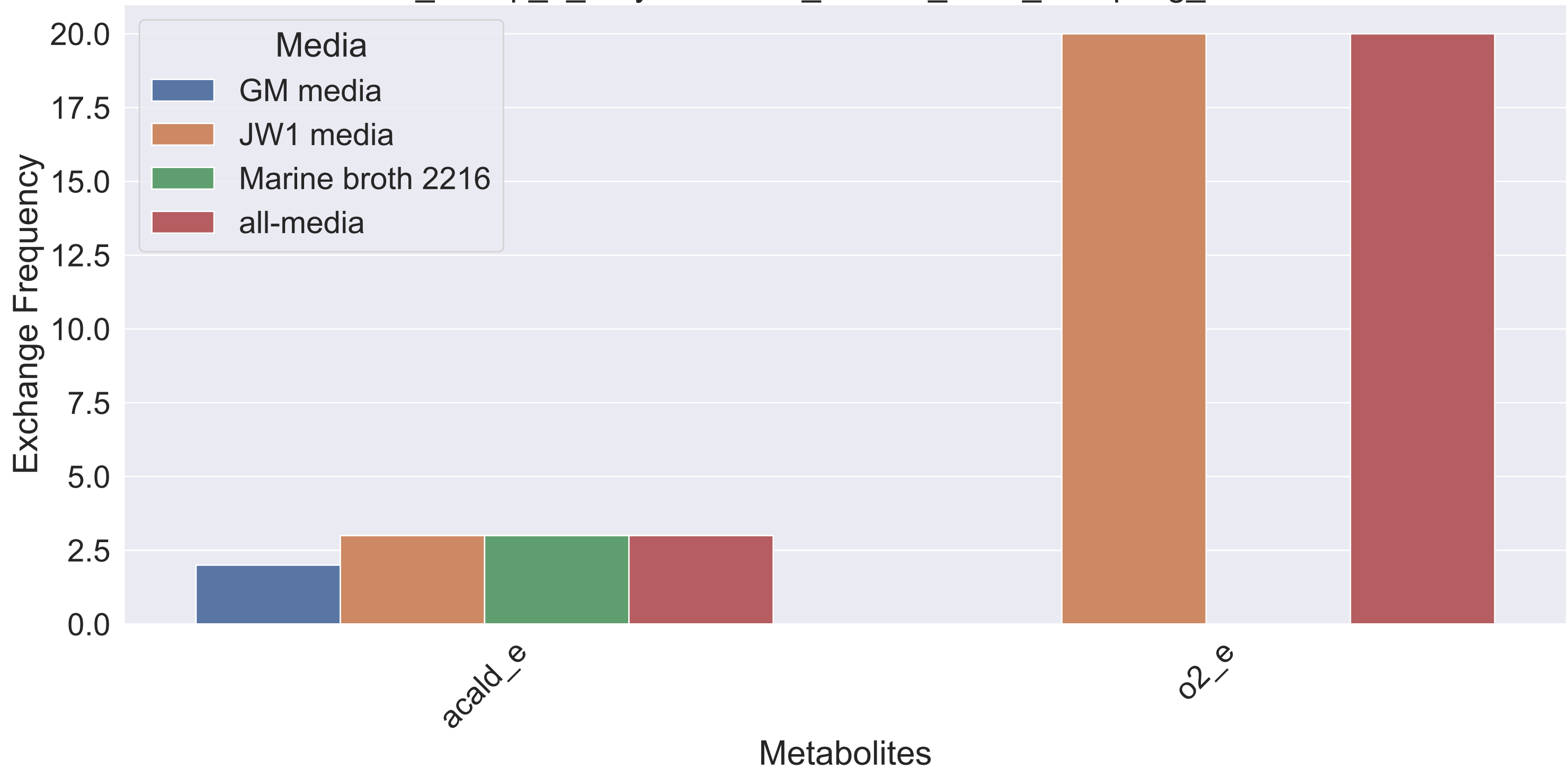

Supplement: Supplementary file 6 — Supplementary File S5 [file 43705_2023_242_MOESM6_ESM.zip › Supplementary Figure S64 Marine Group II euryarchaeote UWMA 0344 accepting metabolites.pdf]

Marine\_Group\_II\_euryarchaeote\_UWMA\_0350\_accepting\_metabolites

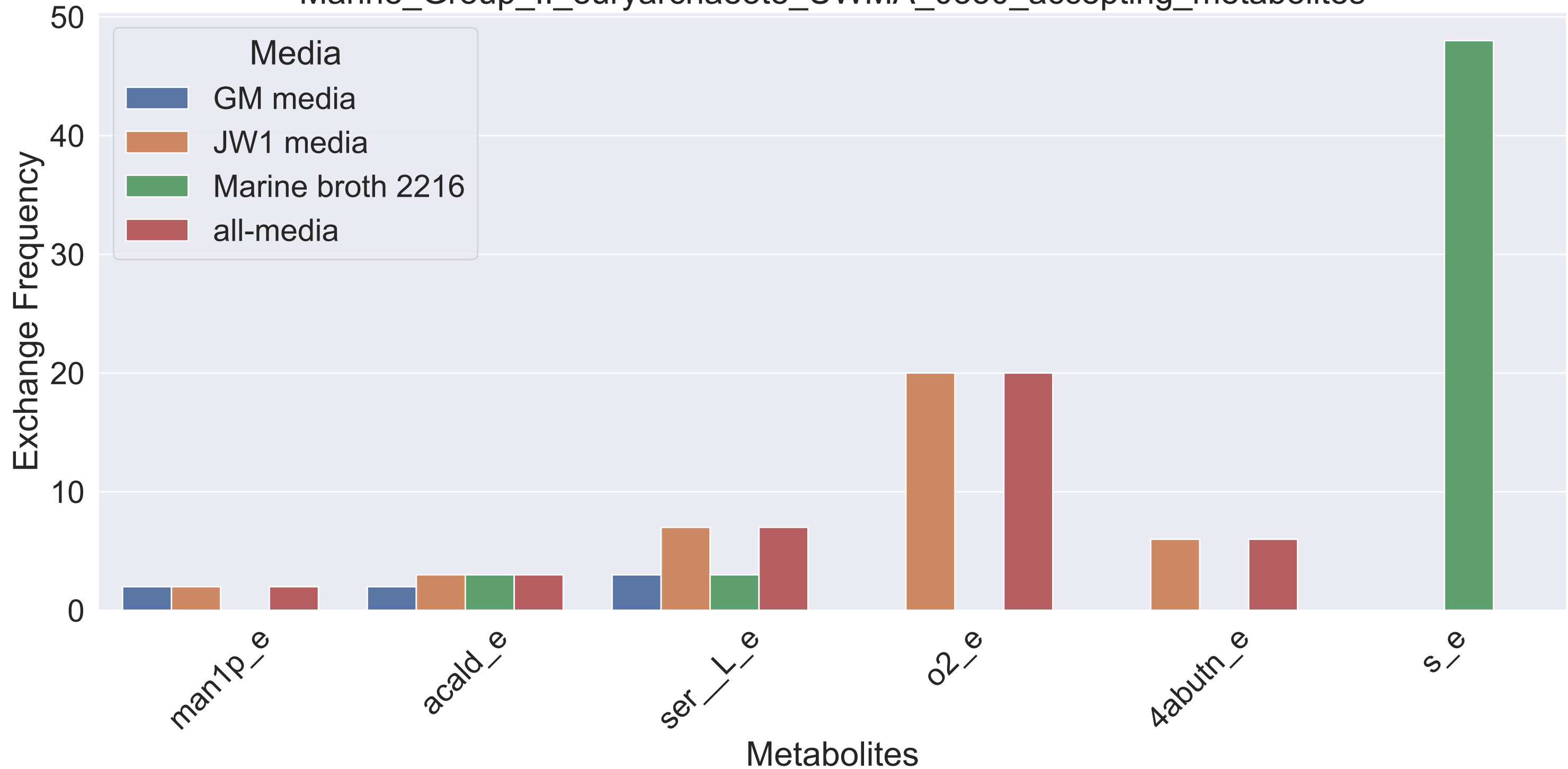

Supplement: Supplementary file 6 — Supplementary File S5 [file 43705_2023_242_MOESM6_ESM.zip › Supplementary Figure S65 Marine Group II euryarchaeote UWMA 0350 accepting metabolites.pdf]

# Marine\_Group\_II\_euryarchaeote\_UWMA\_0352\_accepting\_metabolites

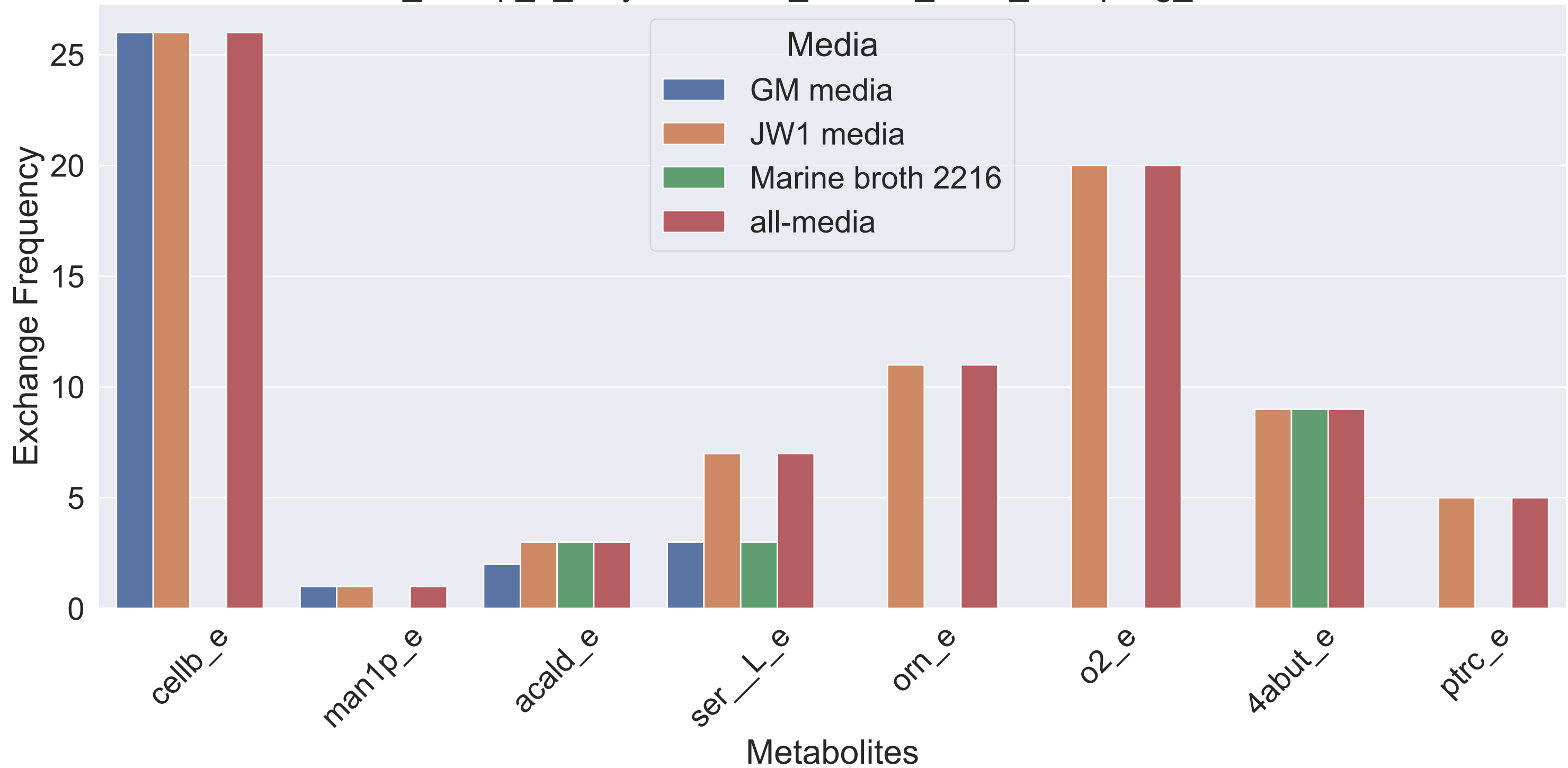

Supplement: Supplementary file 6 — Supplementary File S5 [file 43705_2023_242_MOESM6_ESM.zip › Supplementary Figure S66 Marine Group II euryarchaeote UWMA 0352 accepting metabolites.pdf]

Marine\_Group\_II\_euryarchaeote\_UWMA\_0357\_accepting\_metabolites

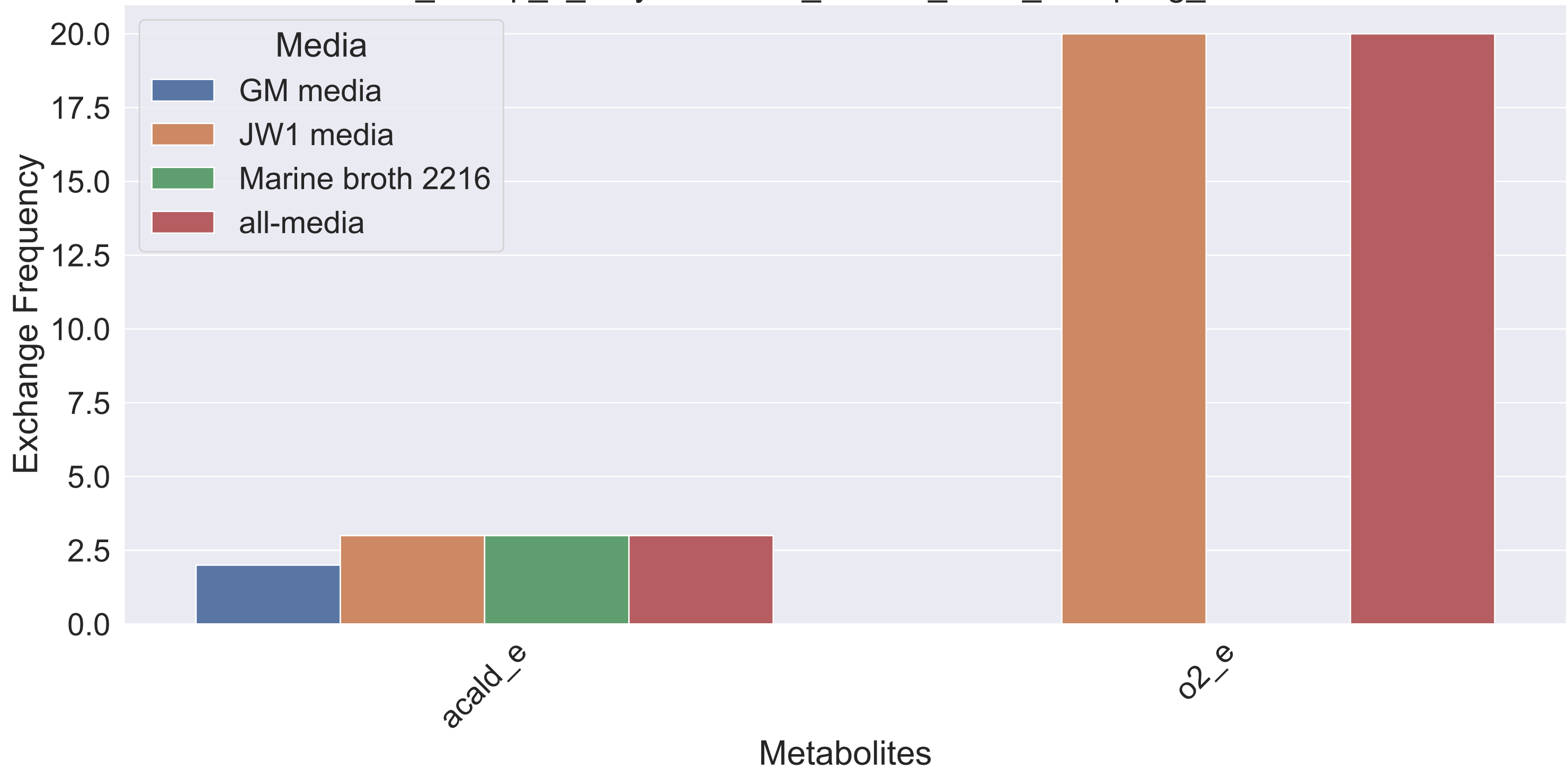

Supplement: Supplementary file 6 — Supplementary File S5 [file 43705_2023_242_MOESM6_ESM.zip › Supplementary Figure S67 Marine Group II euryarchaeote UWMA 0357 accepting metabolites.pdf]

# Methylococcaceae\_bacterium\_UWMA\_0325\_accepting\_metabolites

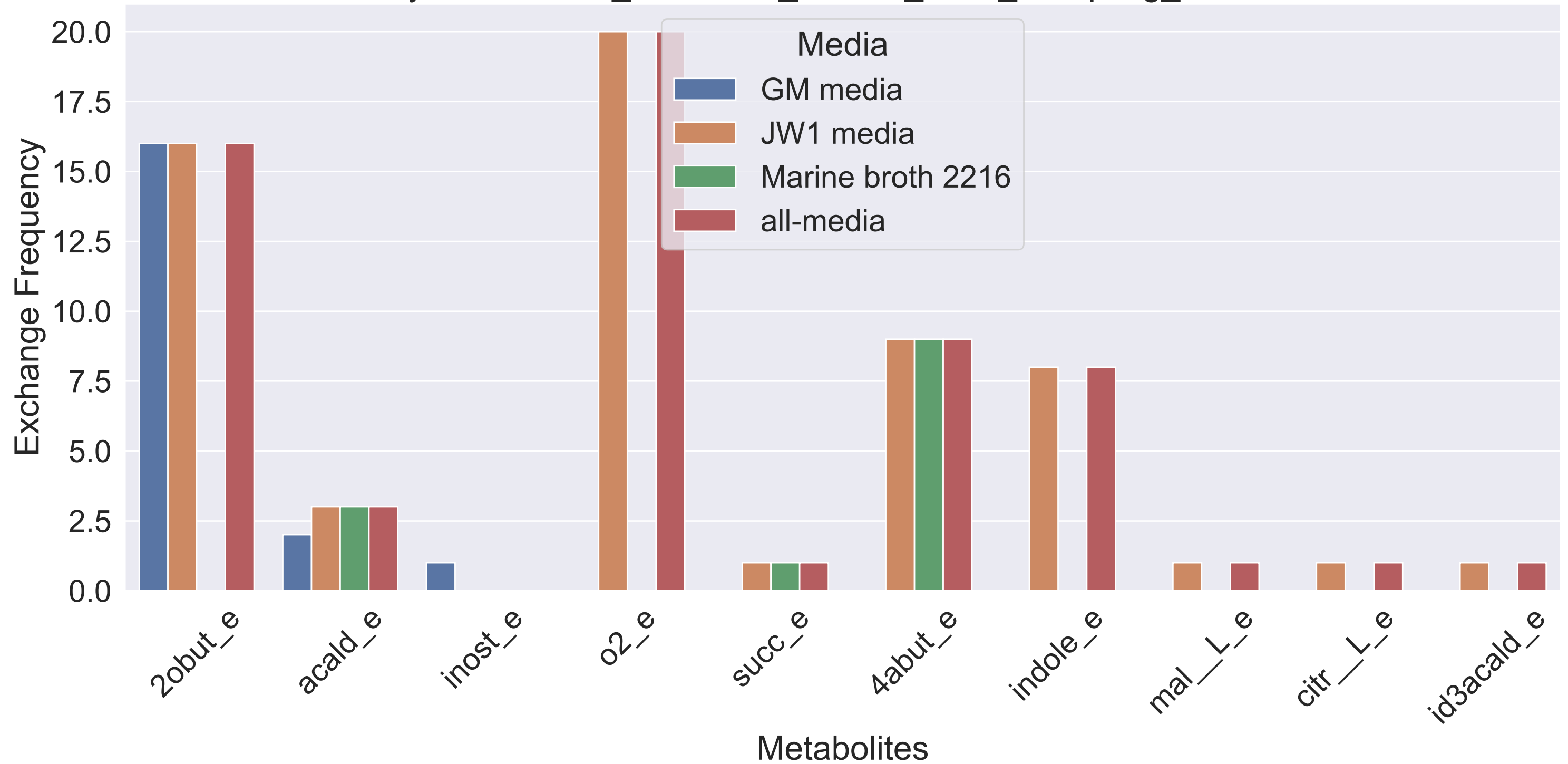

Supplement: Supplementary file 6 — Supplementary File S5 [file 43705_2023_242_MOESM6_ESM.zip › Supplementary Figure S68 Methylococcaceae bacterium UWMA 0325 accepting metabolites.pdf]

Methylococcaceae\_bacterium\_UWMA\_0327\_accepting\_metabolites

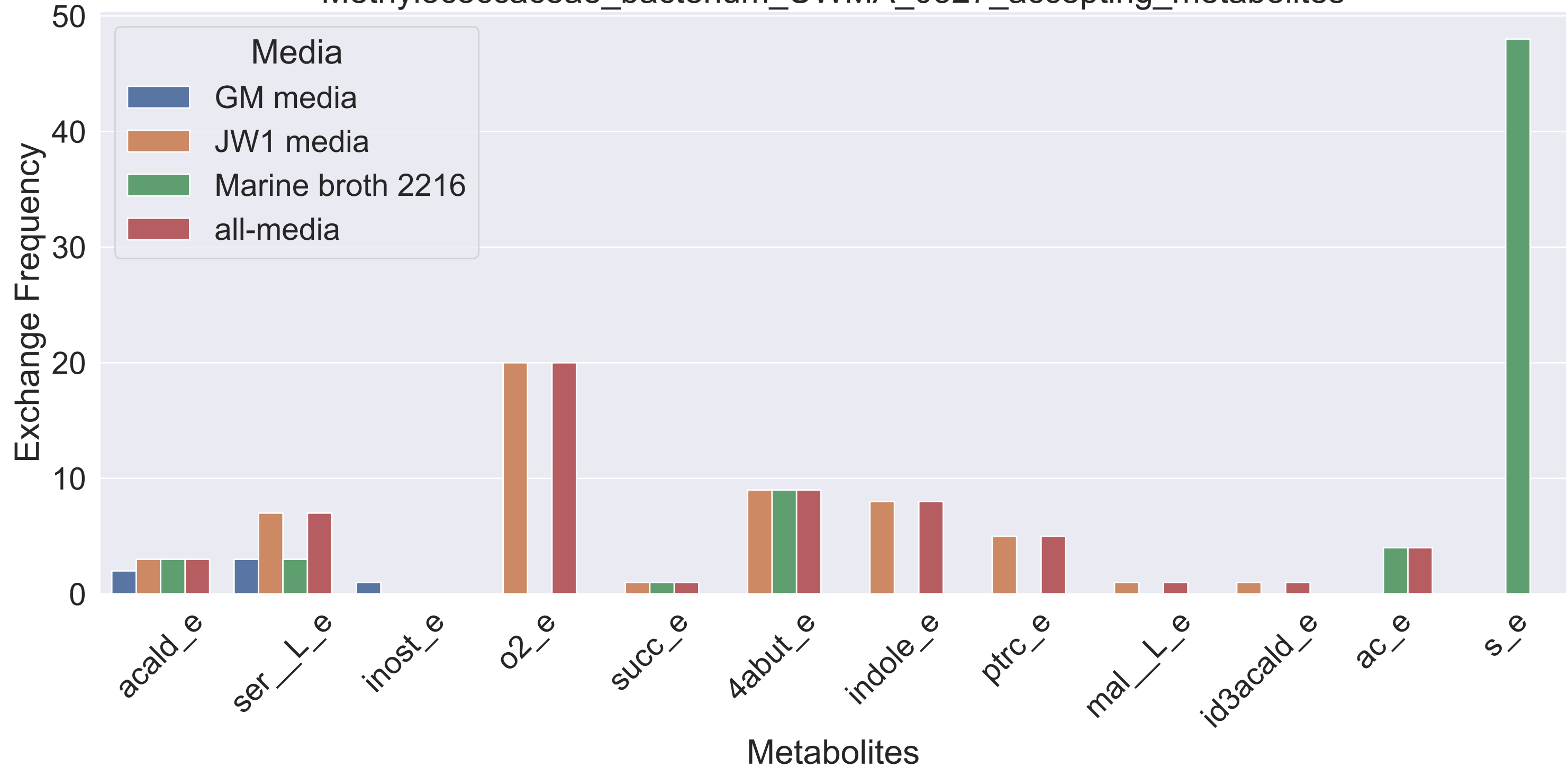

Supplement: Supplementary file 6 — Supplementary File S5 [file 43705_2023_242_MOESM6_ESM.zip › Supplementary Figure S69 Methylococcaceae bacterium UWMA 0327 accepting metabolites.pdf]

Bacteroidetes\_bacterium\_UWMA\_0358\_accepting\_metabolites

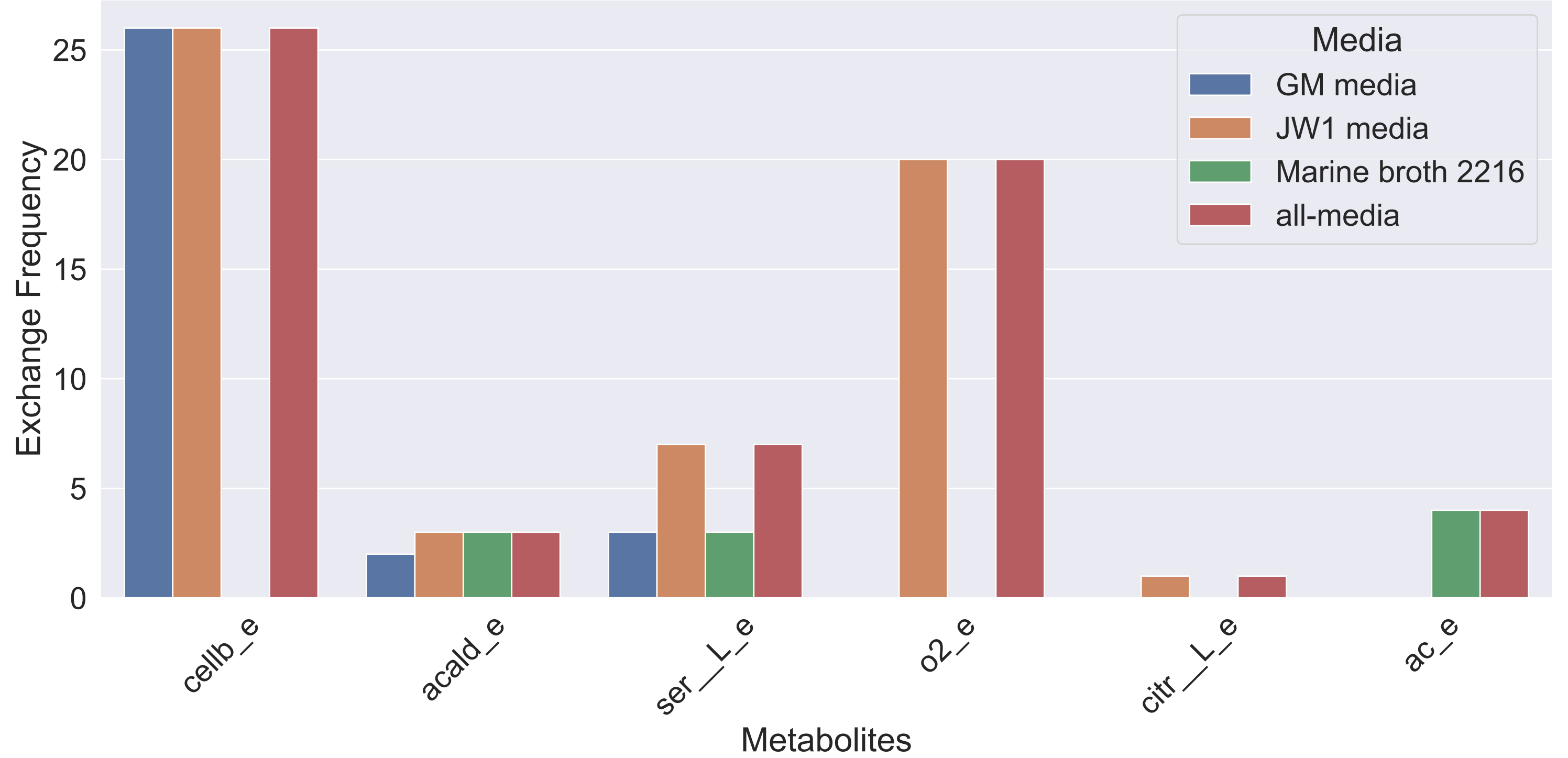

Supplement: Supplementary file 6 — Supplementary File S5 [file 43705_2023_242_MOESM6_ESM.zip › Supplementary Figure S7 Bacteroidetes bacterium UWMA 0358 accepting metabolites.pdf]

# Methylococcaceae\_bacterium\_UWMA\_0348\_accepting\_metabolites

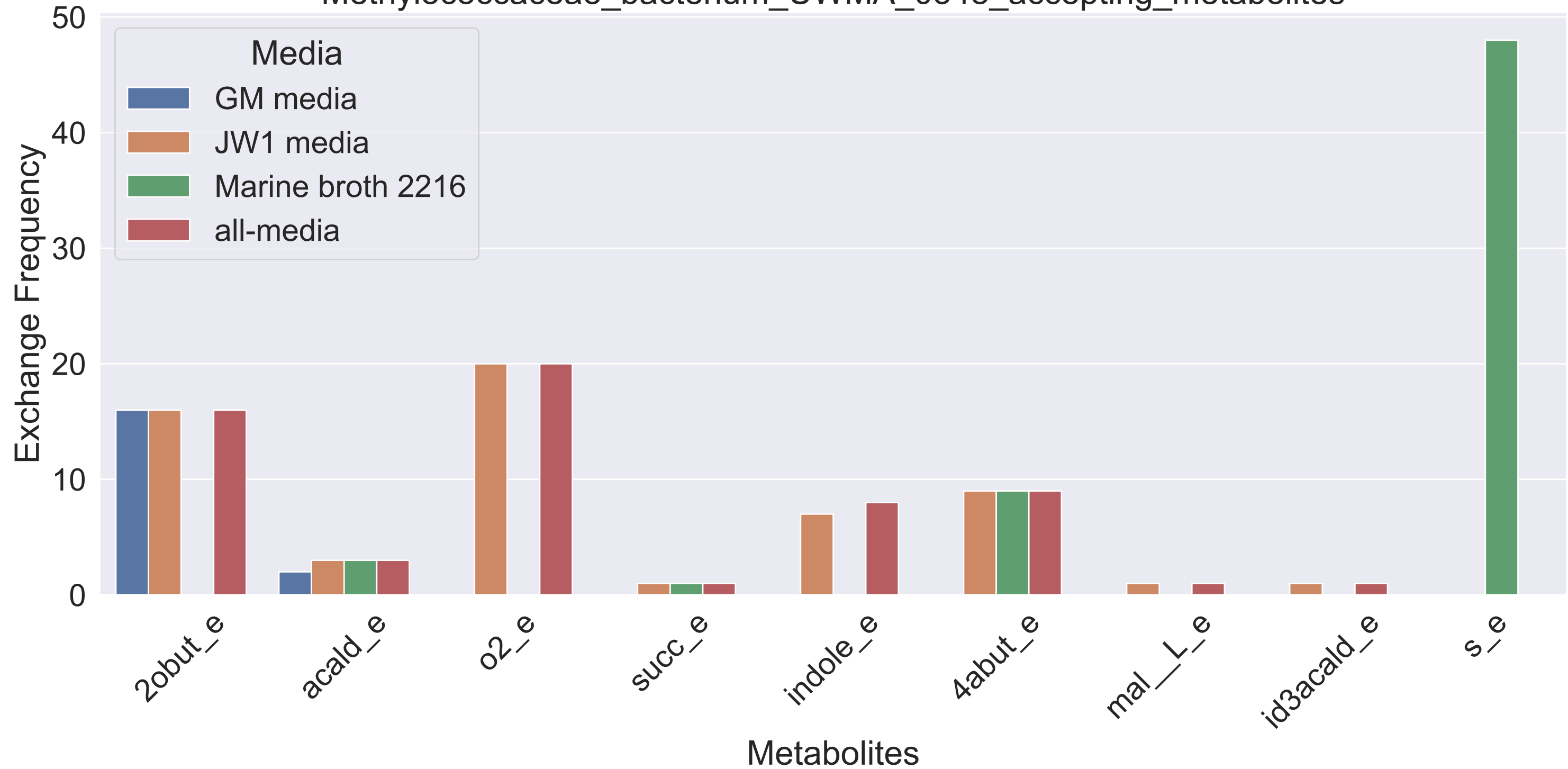

Supplement: Supplementary file 6 — Supplementary File S5 [file 43705_2023_242_MOESM6_ESM.zip › Supplementary Figure S70 Methylococcaceae bacterium UWMA 0348 accepting metabolites.pdf]

# Methylophilaceae\_bacterium\_UWMA\_0326\_accepting\_metabolites

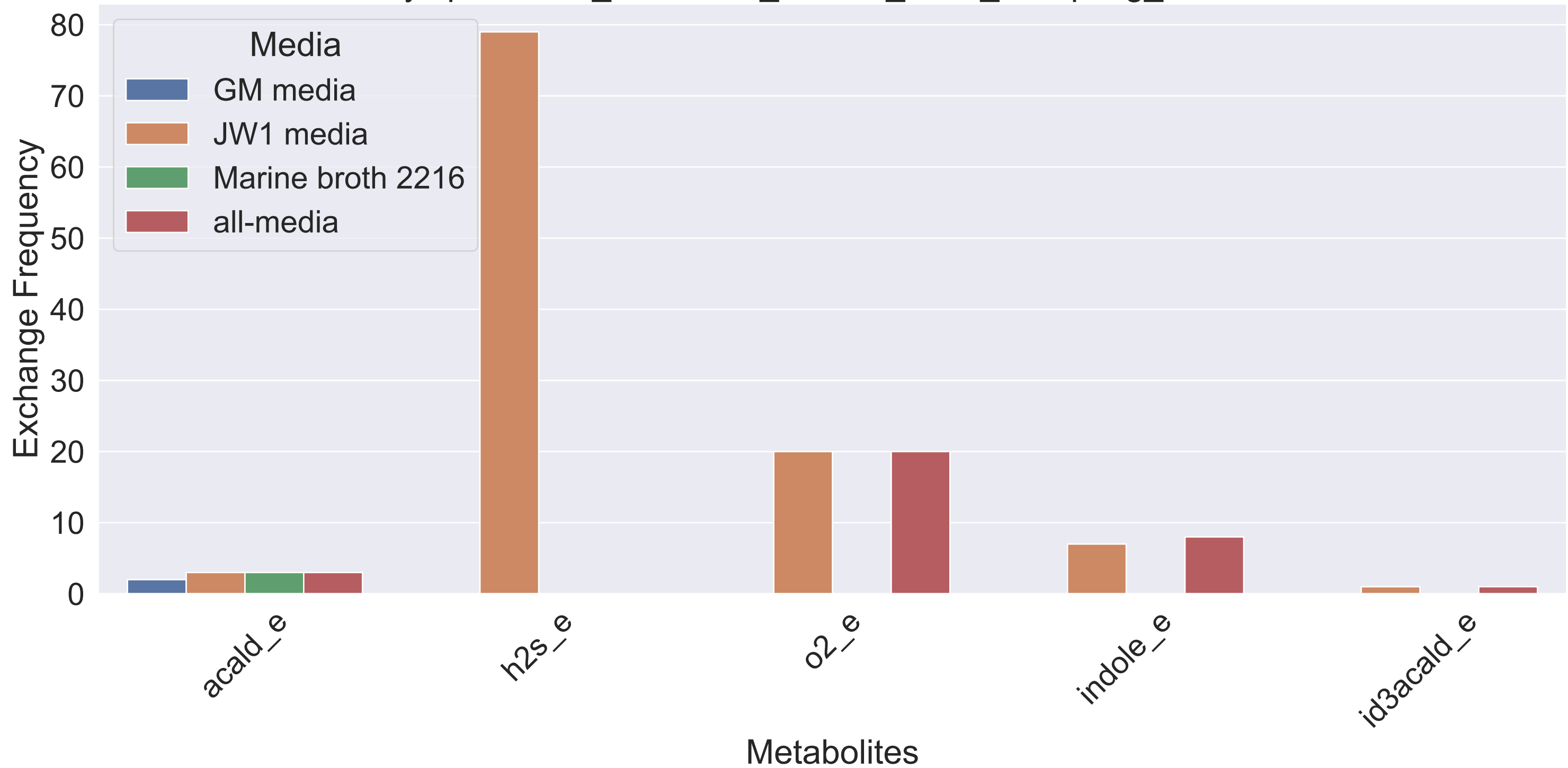

Supplement: Supplementary file 6 — Supplementary File S5 [file 43705_2023_242_MOESM6_ESM.zip › Supplementary Figure S71 Methylophilaceae bacterium UWMA 0326 accepting metabolites.pdf]

# Methyloprofundus\_sp\_UWMA\_0312\_accepting\_metabolites

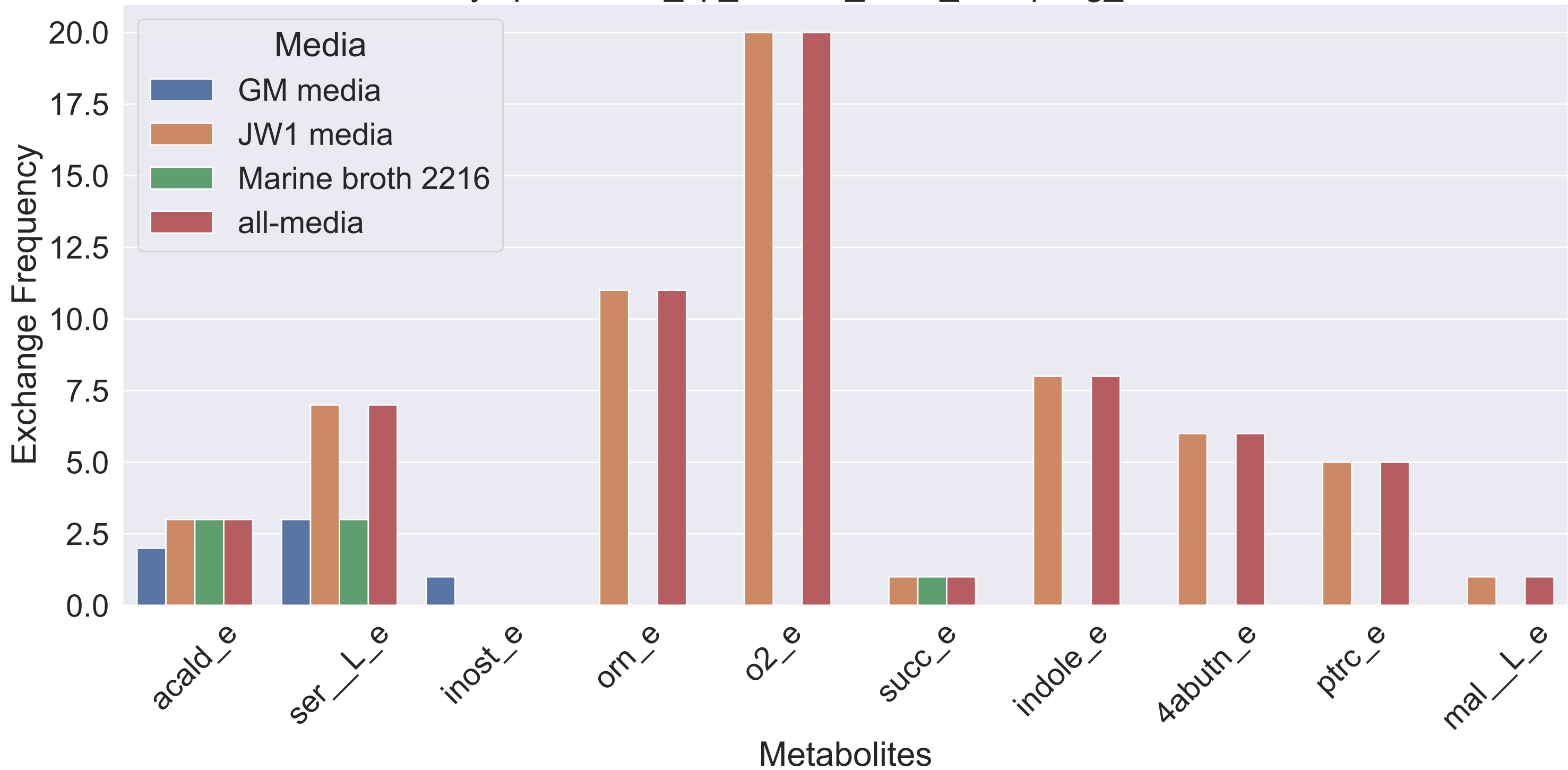

Supplement: Supplementary file 6 — Supplementary File S5 [file 43705_2023_242_MOESM6_ESM.zip › Supplementary Figure S72 Methyloprofundus sp UWMA 0312 accepting metabolites.pdf]

Micavibrio\_sp\_UWMA\_0310\_accepting\_metabolites

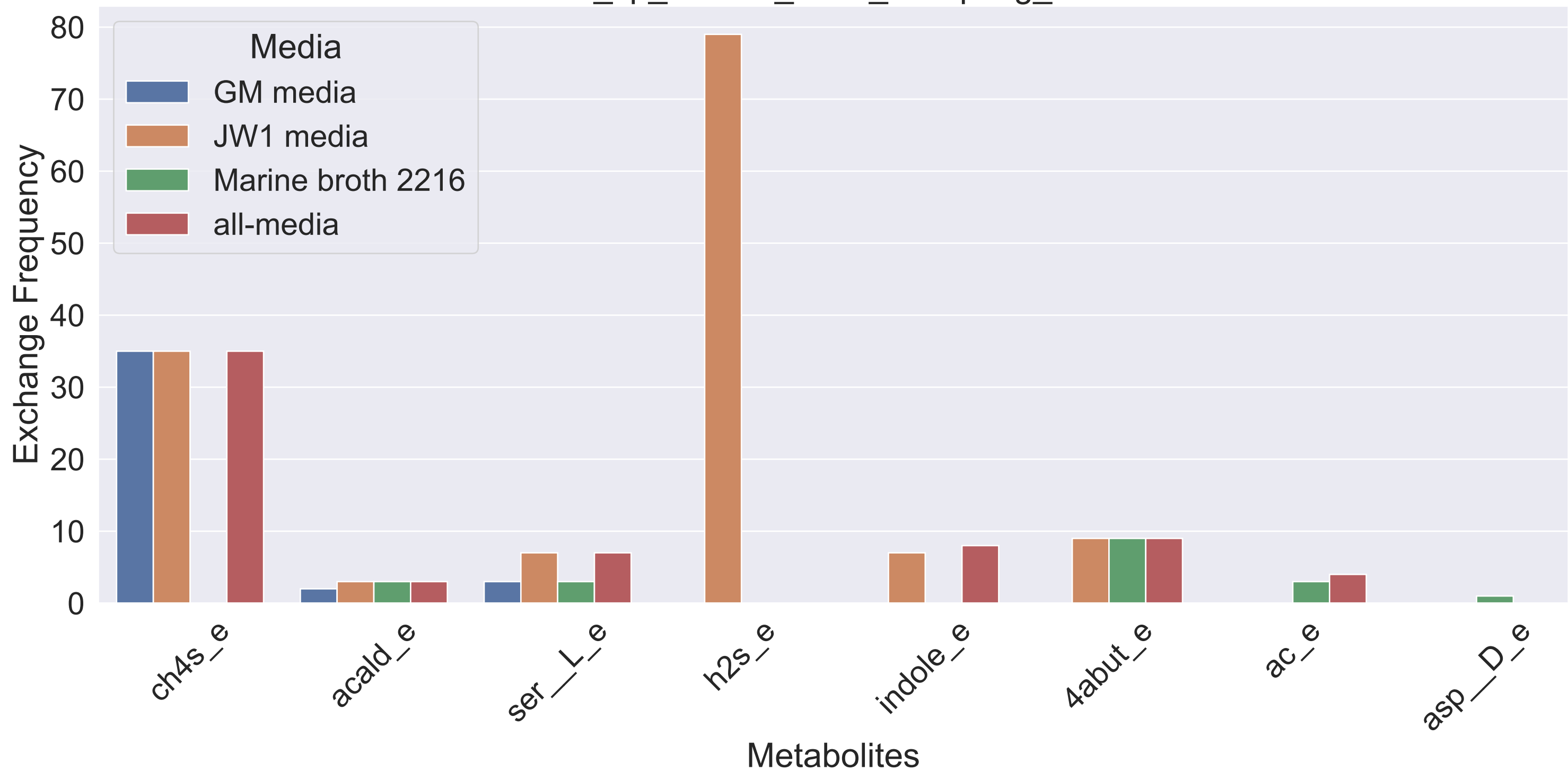

Supplement: Supplementary file 6 — Supplementary File S5 [file 43705_2023_242_MOESM6_ESM.zip › Supplementary Figure S73 Micavibrio sp UWMA 0310 accepting metabolites.pdf]

# Microbacterium\_sp\_UWMA\_0270\_accepting\_metabolites

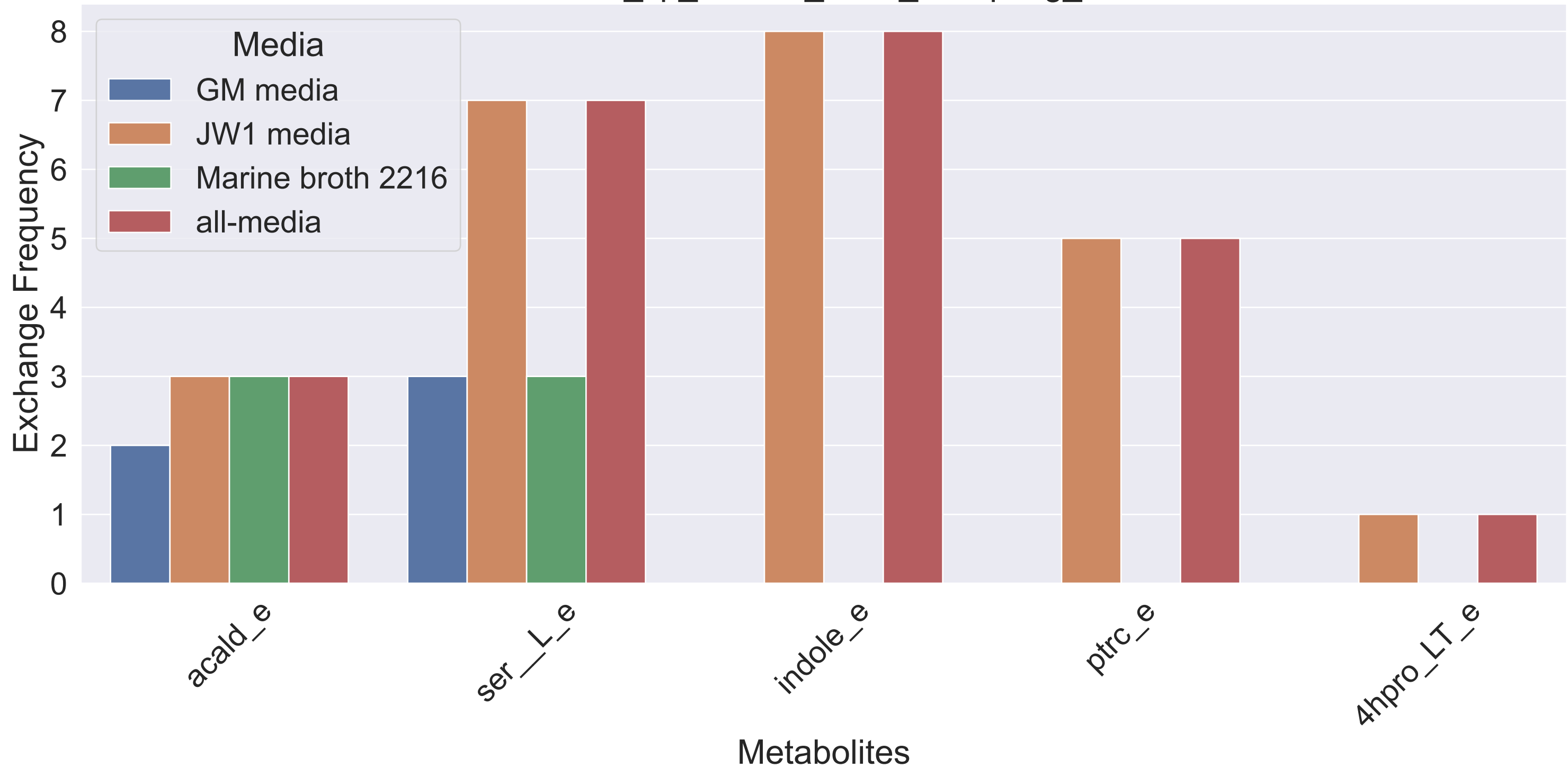

Supplement: Supplementary file 6 — Supplementary File S5 [file 43705_2023_242_MOESM6_ESM.zip › Supplementary Figure S74 Microbacterium sp UWMA 0270 accepting metabolites.pdf]

Myxococcales\_bacterium\_UWMA\_0315\_accepting\_metabolites

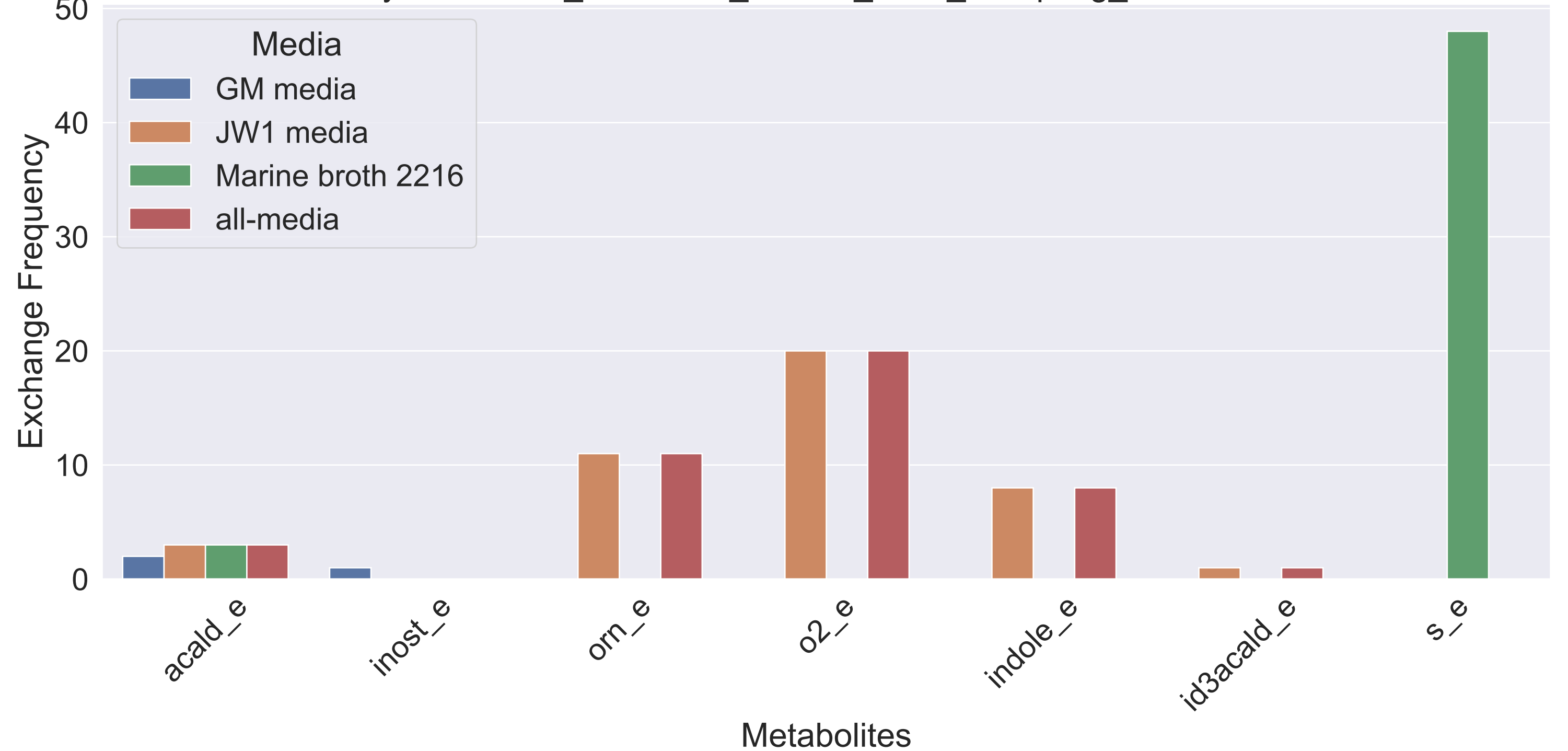

Supplement: Supplementary file 6 — Supplementary File S5 [file 43705_2023_242_MOESM6_ESM.zip › Supplementary Figure S75 Myxococcales bacterium UWMA 0315 accepting metabolites.pdf]

# Myxococcales\_bacterium\_UWMA\_0324\_accepting\_metabolites

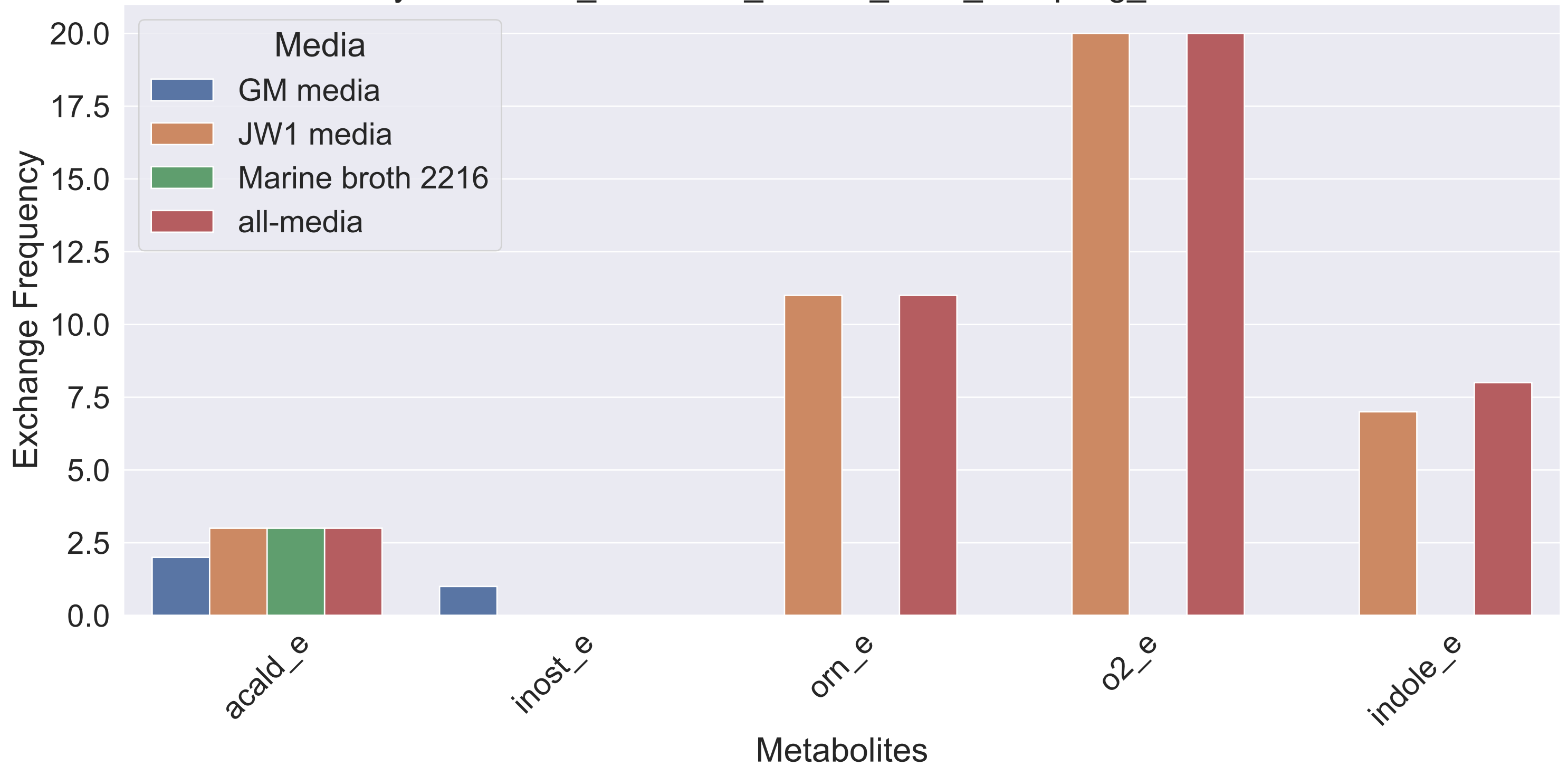

Supplement: Supplementary file 6 — Supplementary File S5 [file 43705_2023_242_MOESM6_ESM.zip › Supplementary Figure S76 Myxococcales bacterium UWMA 0324 accepting metabolites.pdf]

# Myxococcales\_bacterium\_UWMA\_0330\_accepting\_metabolites

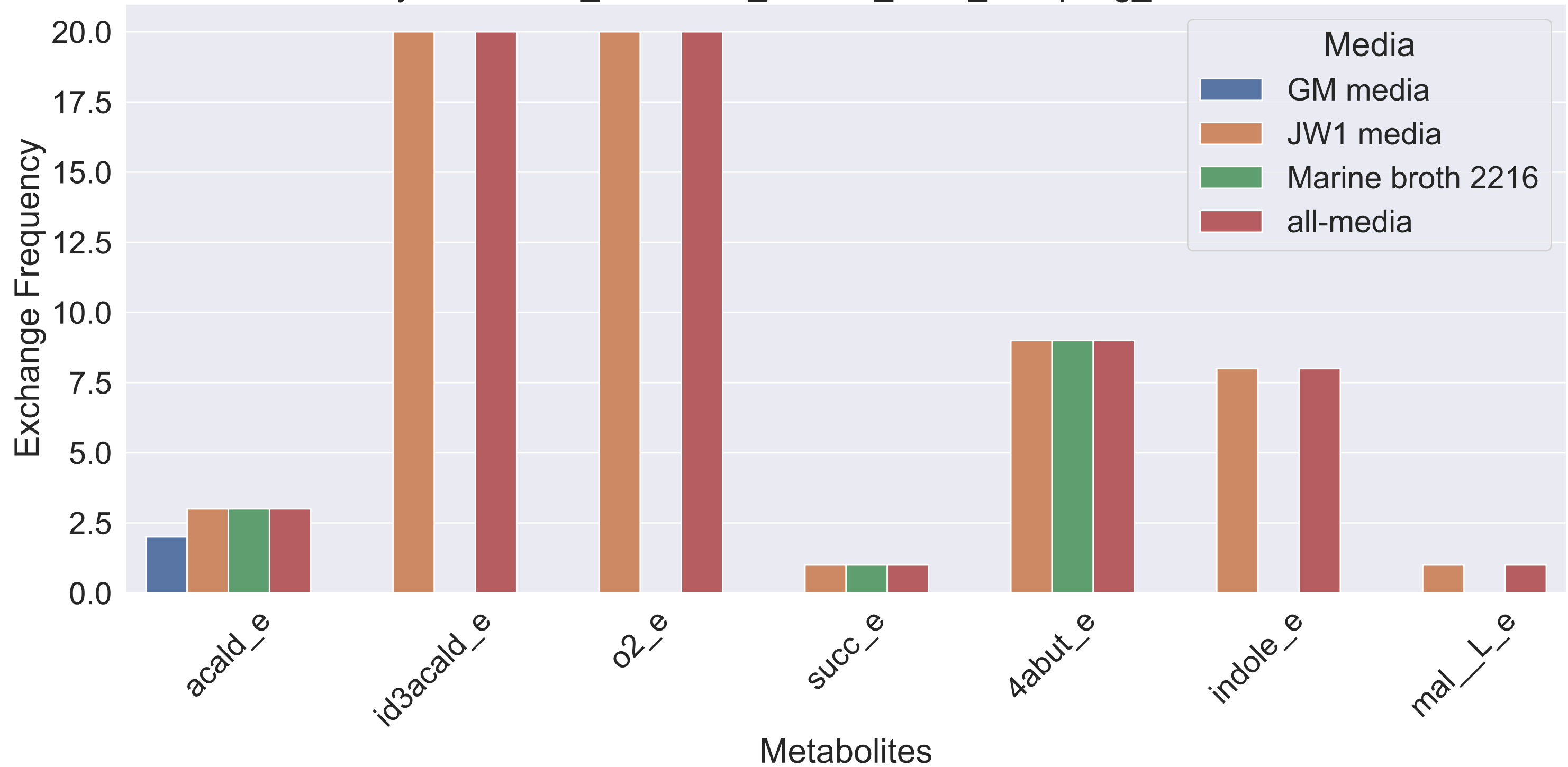

Supplement: Supplementary file 6 — Supplementary File S5 [file 43705_2023_242_MOESM6_ESM.zip › Supplementary Figure S77 Myxococcales bacterium UWMA 0330 accepting metabolites.pdf]

Myxococcales\_bacterium\_UWMA\_0336\_accepting\_metabolites

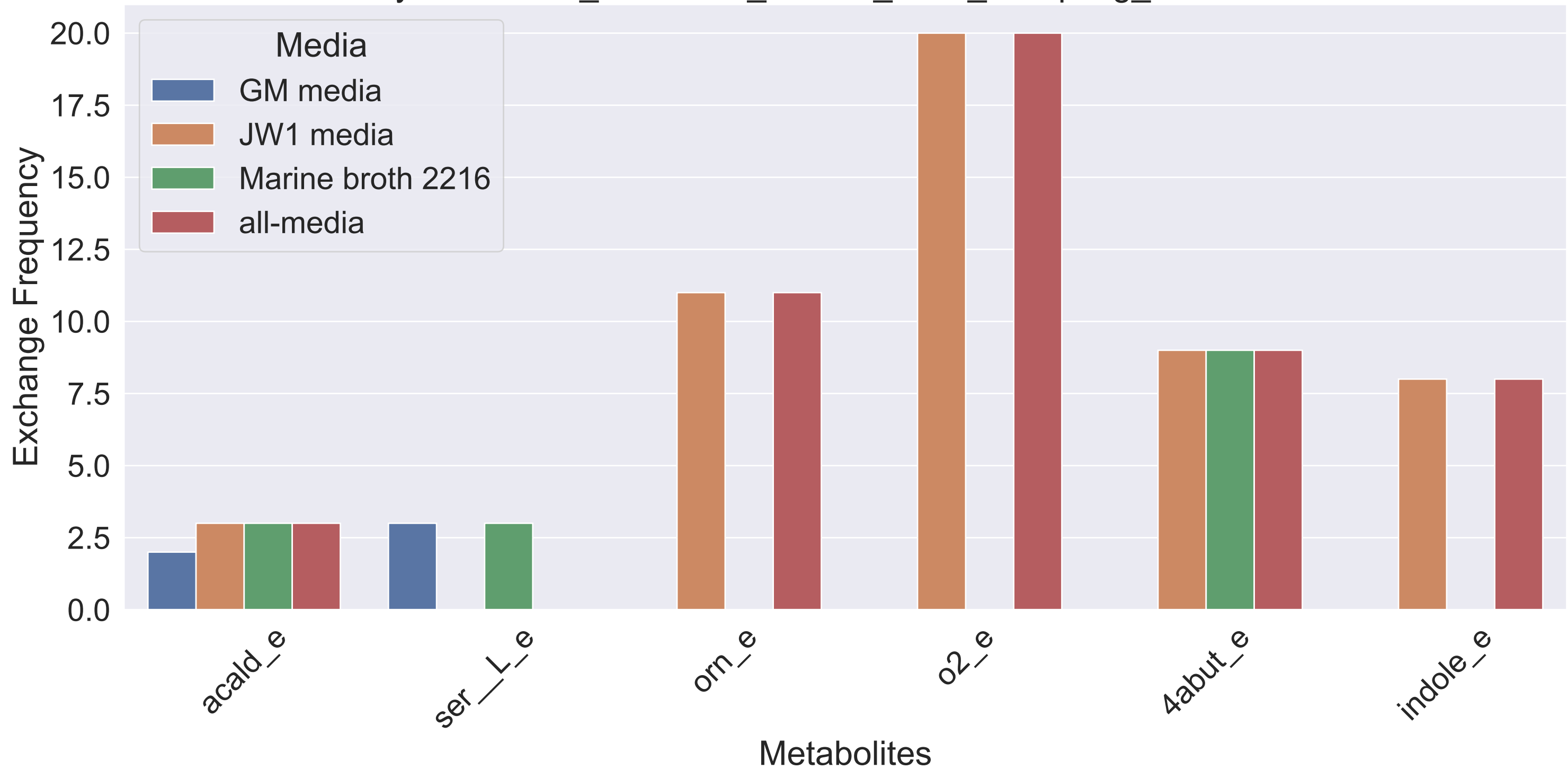

Supplement: Supplementary file 6 — Supplementary File S5 [file 43705_2023_242_MOESM6_ESM.zip › Supplementary Figure S78 Myxococcales bacterium UWMA 0336 accepting metabolites.pdf]

# Nitrosopumilus\_sp\_UWMA\_0263\_accepting\_metabolites

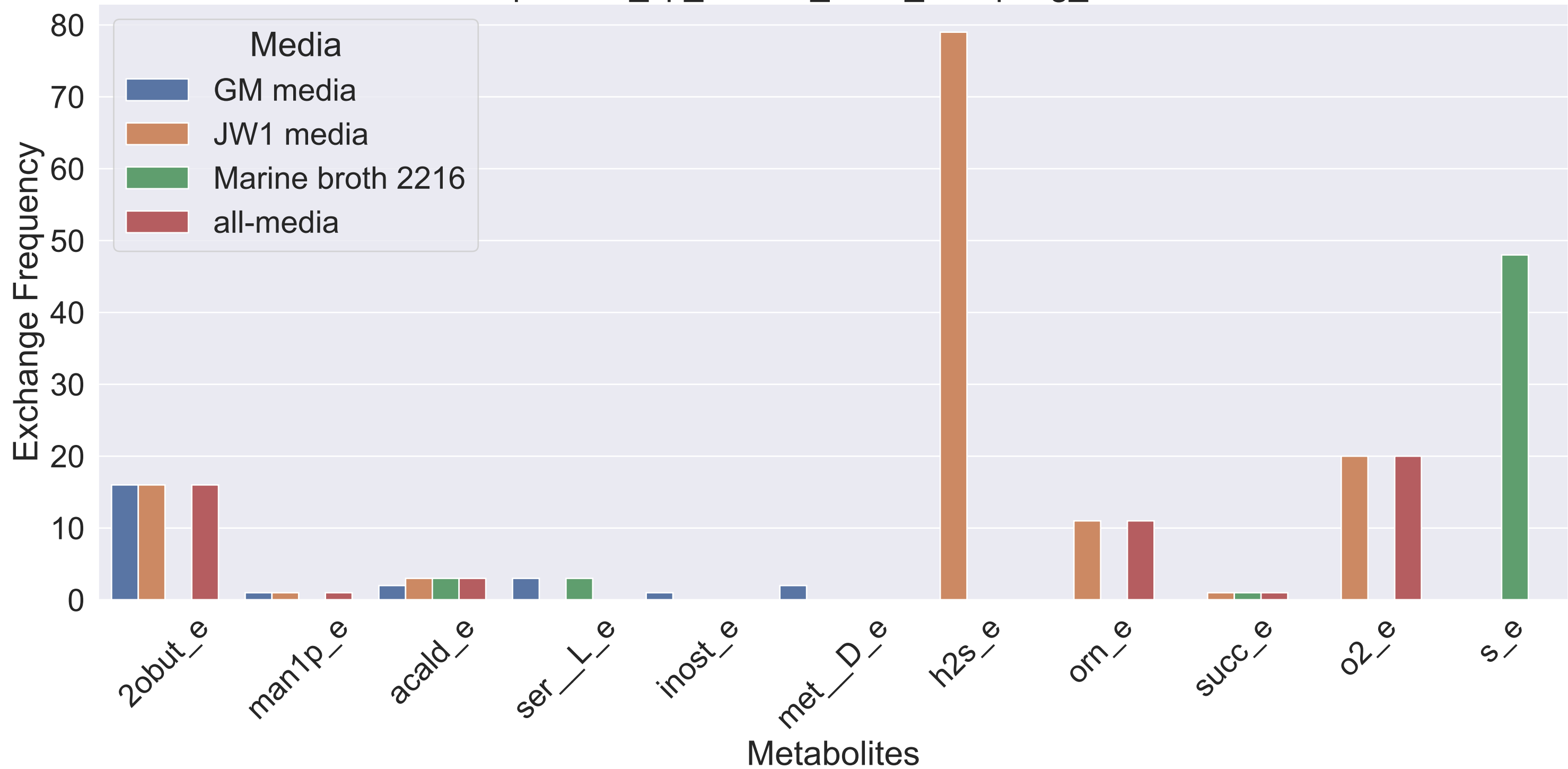

Supplement: Supplementary file 6 — Supplementary File S5 [file 43705_2023_242_MOESM6_ESM.zip › Supplementary Figure S79 Nitrosopumilus sp UWMA 0263 accepting metabolites.pdf]

candidate\_division\_UBP10\_bacterium\_UWMA\_0304\_accepting\_metabolites

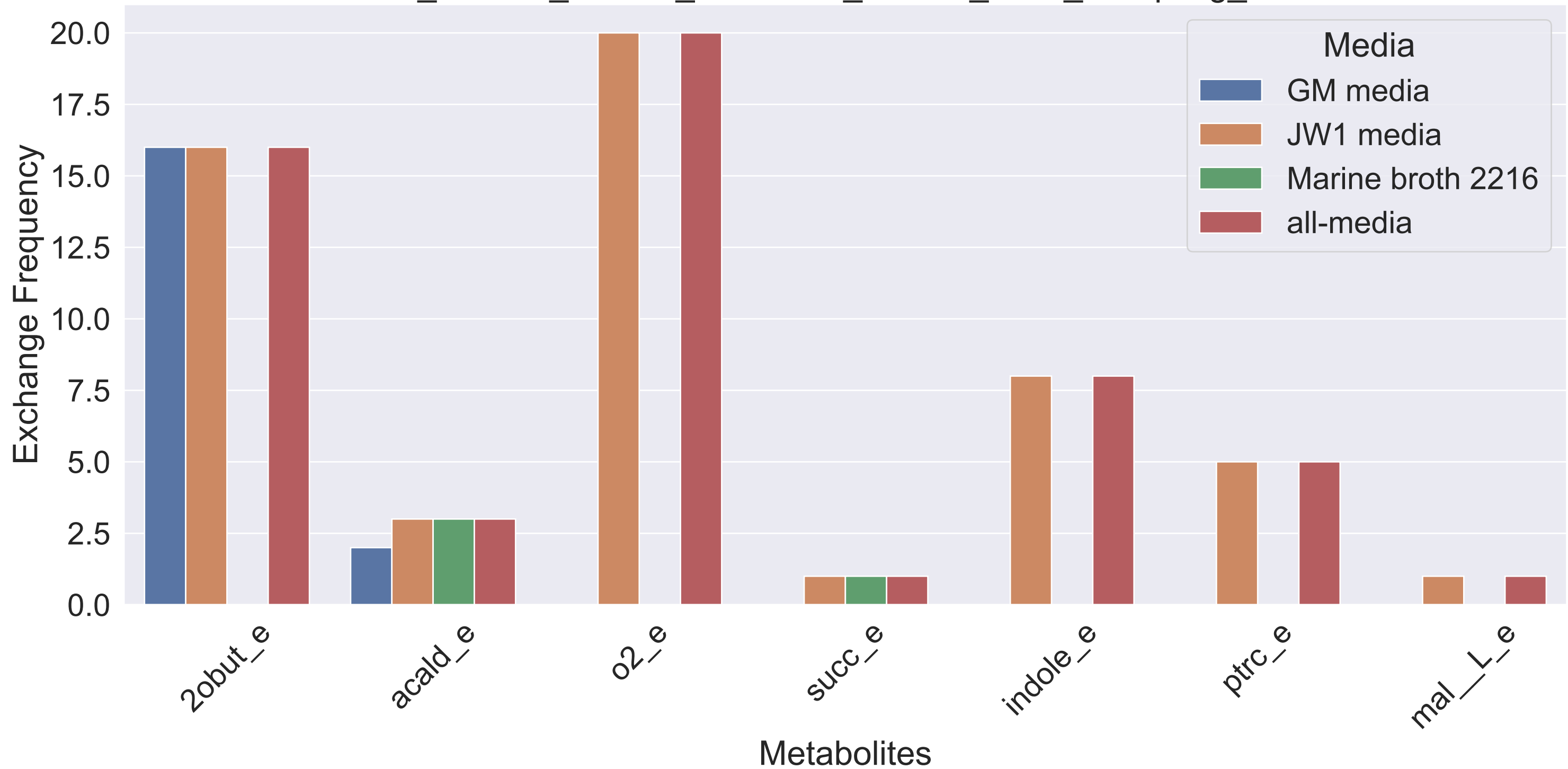

Supplement: Supplementary file 6 — Supplementary File S5 [file 43705_2023_242_MOESM6_ESM.zip › Supplementary Figure S8 candidate division UBP10 bacterium UWMA 0304 accepting metabolites.pdf]

Nitrospinaceae\_bacterium\_UWMA\_0262\_accepting\_metabolites

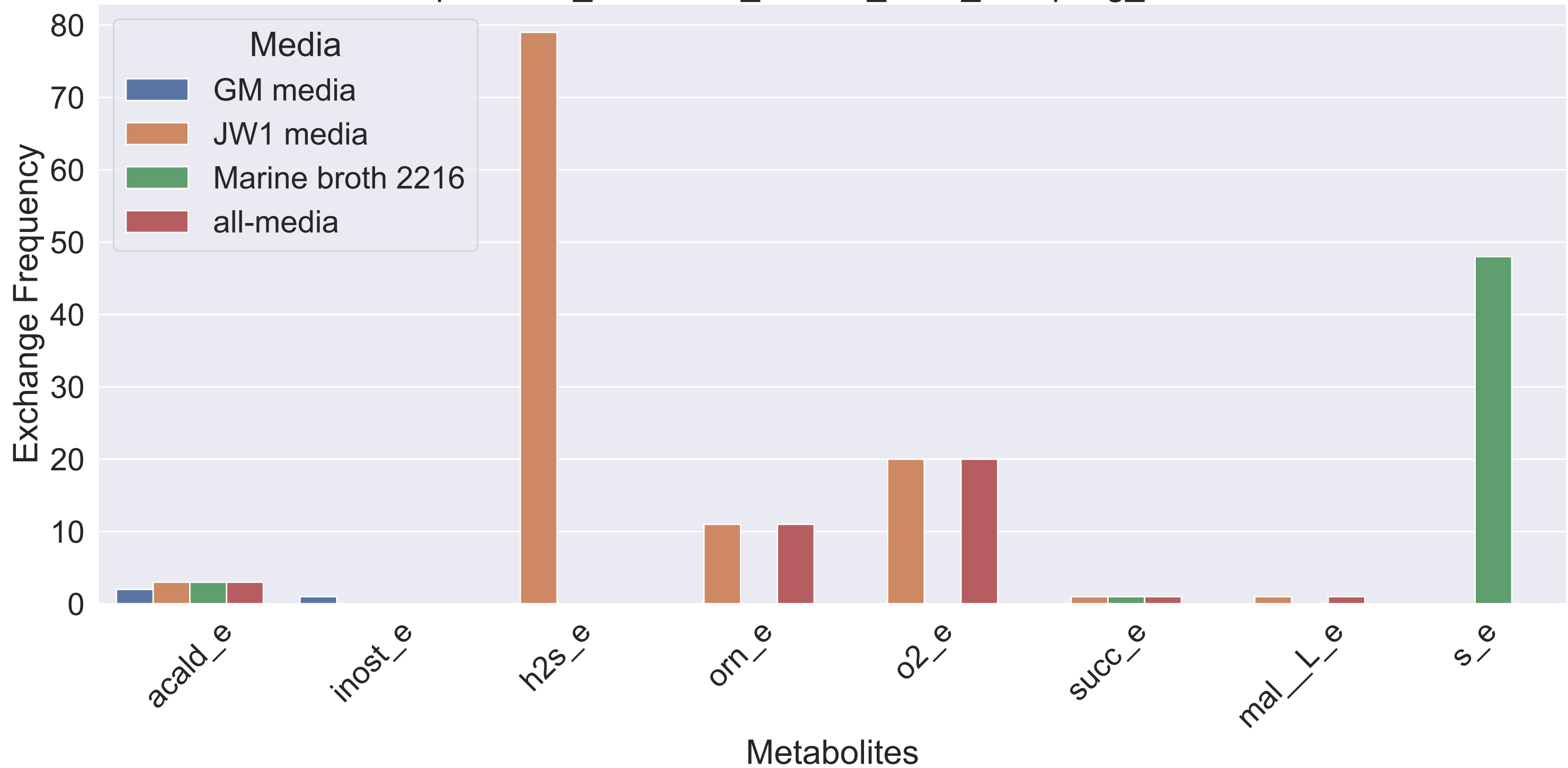

Supplement: Supplementary file 6 — Supplementary File S5 [file 43705_2023_242_MOESM6_ESM.zip › Supplementary Figure S80 Nitrospinaceae bacterium UWMA 0262 accepting metabolites.pdf]

Nitrospinaceae\_bacterium\_UWMA\_0271\_accepting\_metabolites

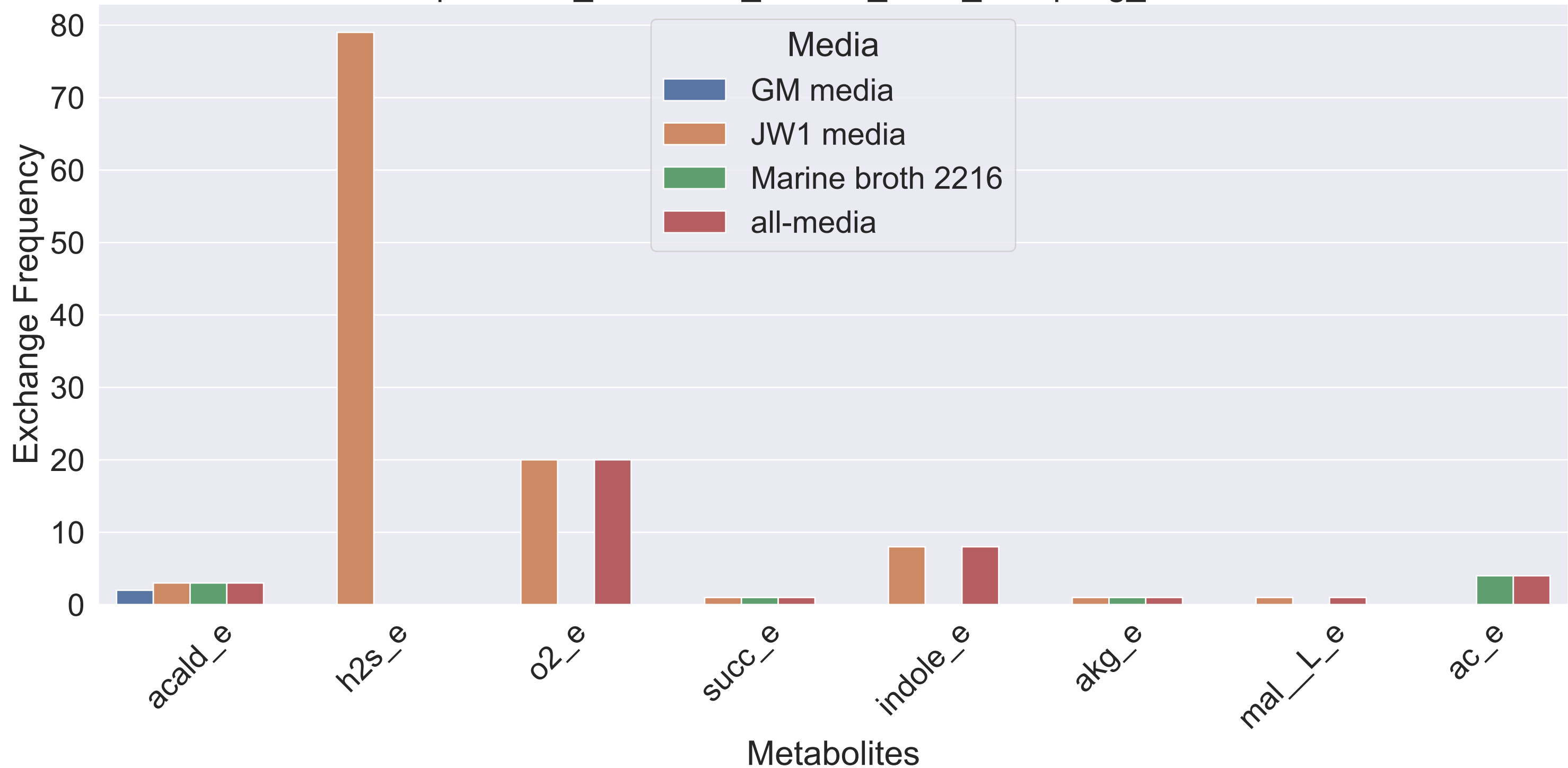

Supplement: Supplementary file 6 — Supplementary File S5 [file 43705_2023_242_MOESM6_ESM.zip › Supplementary Figure S81 Nitrospinaceae bacterium UWMA 0271 accepting metabolites.pdf]

Nitrospinaceae\_bacterium\_UWMA\_0356\_accepting\_metabolites

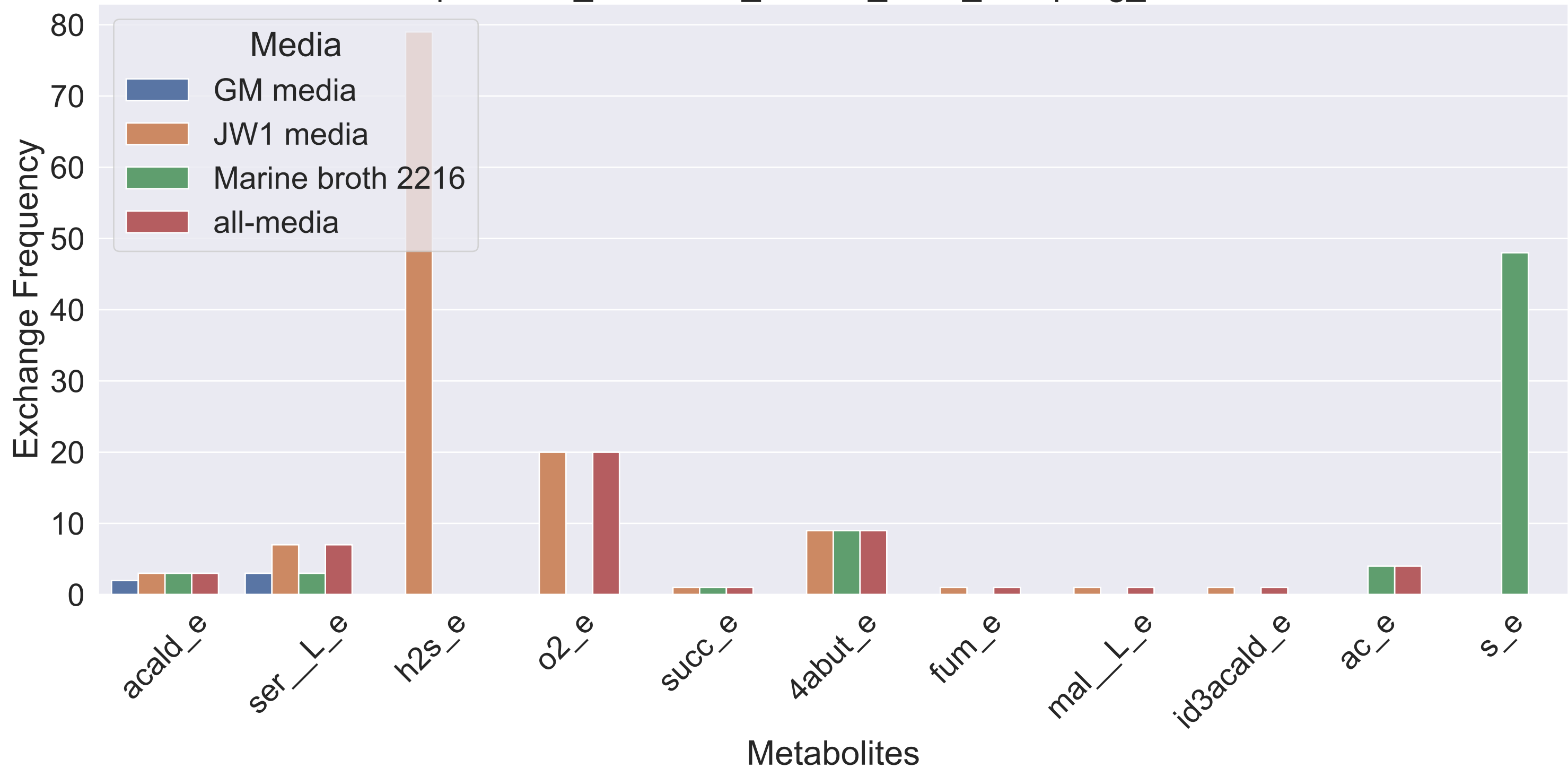

Supplement: Supplementary file 6 — Supplementary File S5 [file 43705_2023_242_MOESM6_ESM.zip › Supplementary Figure S82 Nitrospinaceae bacterium UWMA 0356 accepting metabolites.pdf]

# Nitrospira\_sp\_UWMA\_0268\_accepting\_metabolites

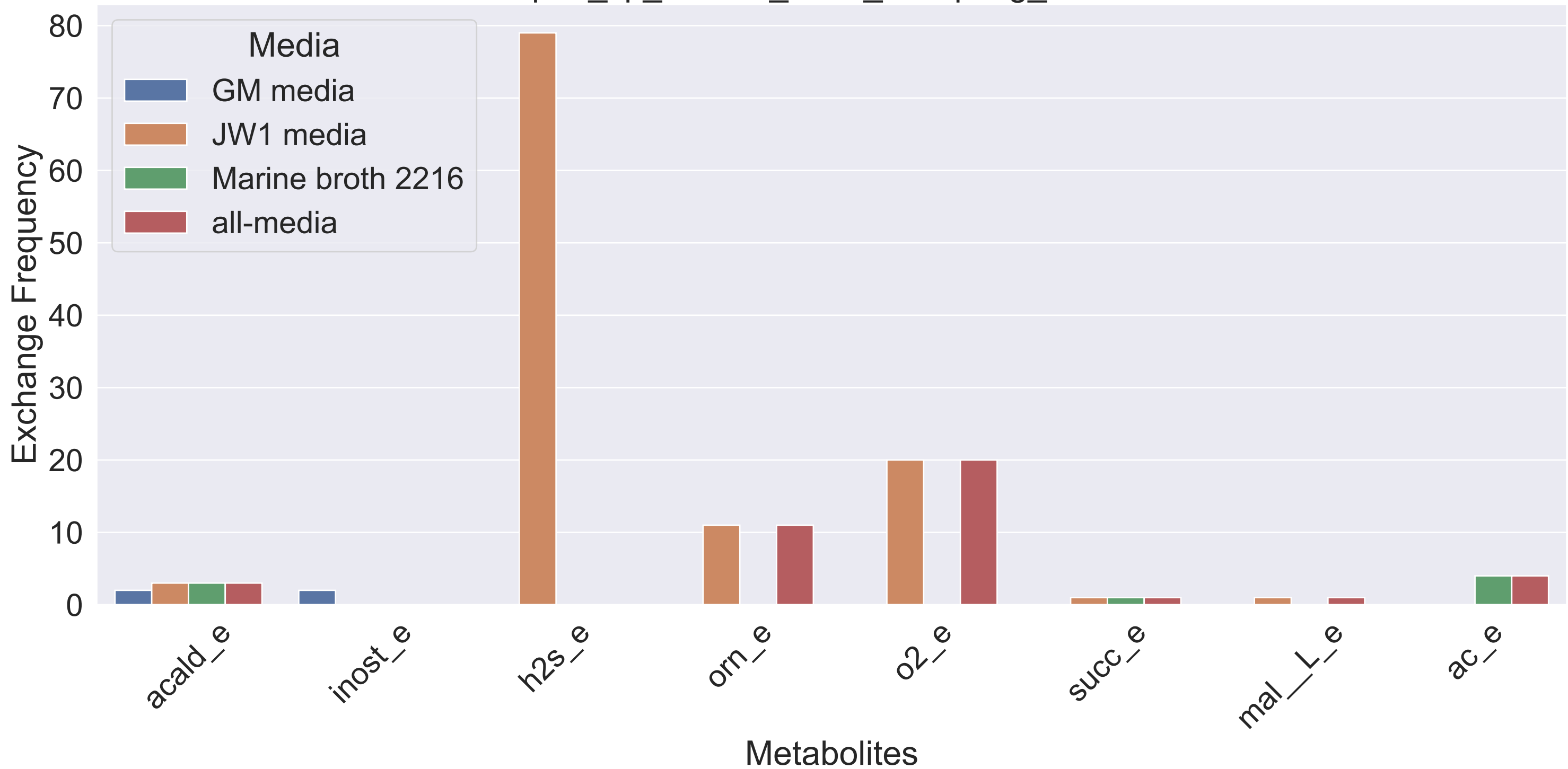

Supplement: Supplementary file 6 — Supplementary File S5 [file 43705_2023_242_MOESM6_ESM.zip › Supplementary Figure S83 Nitrospira sp UWMA 0268 accepting metabolites.pdf]

# Oceanospirillaceae\_bacterium\_UWMA\_0280\_accepting\_metabolites

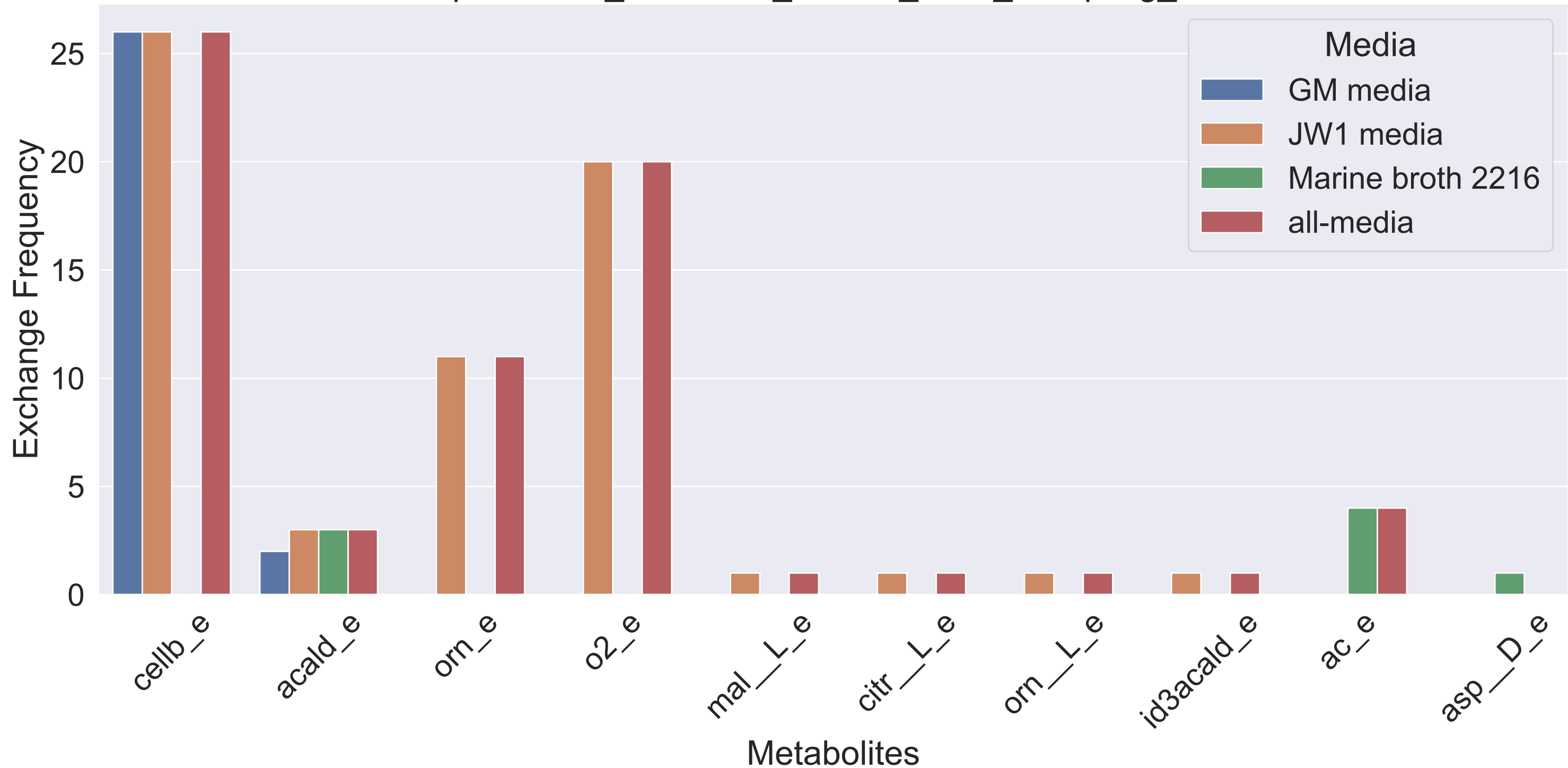

Supplement: Supplementary file 6 — Supplementary File S5 [file 43705_2023_242_MOESM6_ESM.zip › Supplementary Figure S84 Oceanospirillaceae bacterium UWMA 0280 accepting metabolites.pdf]

Planctomycetaceae\_bacterium\_UWMA\_0346\_accepting\_metabolites

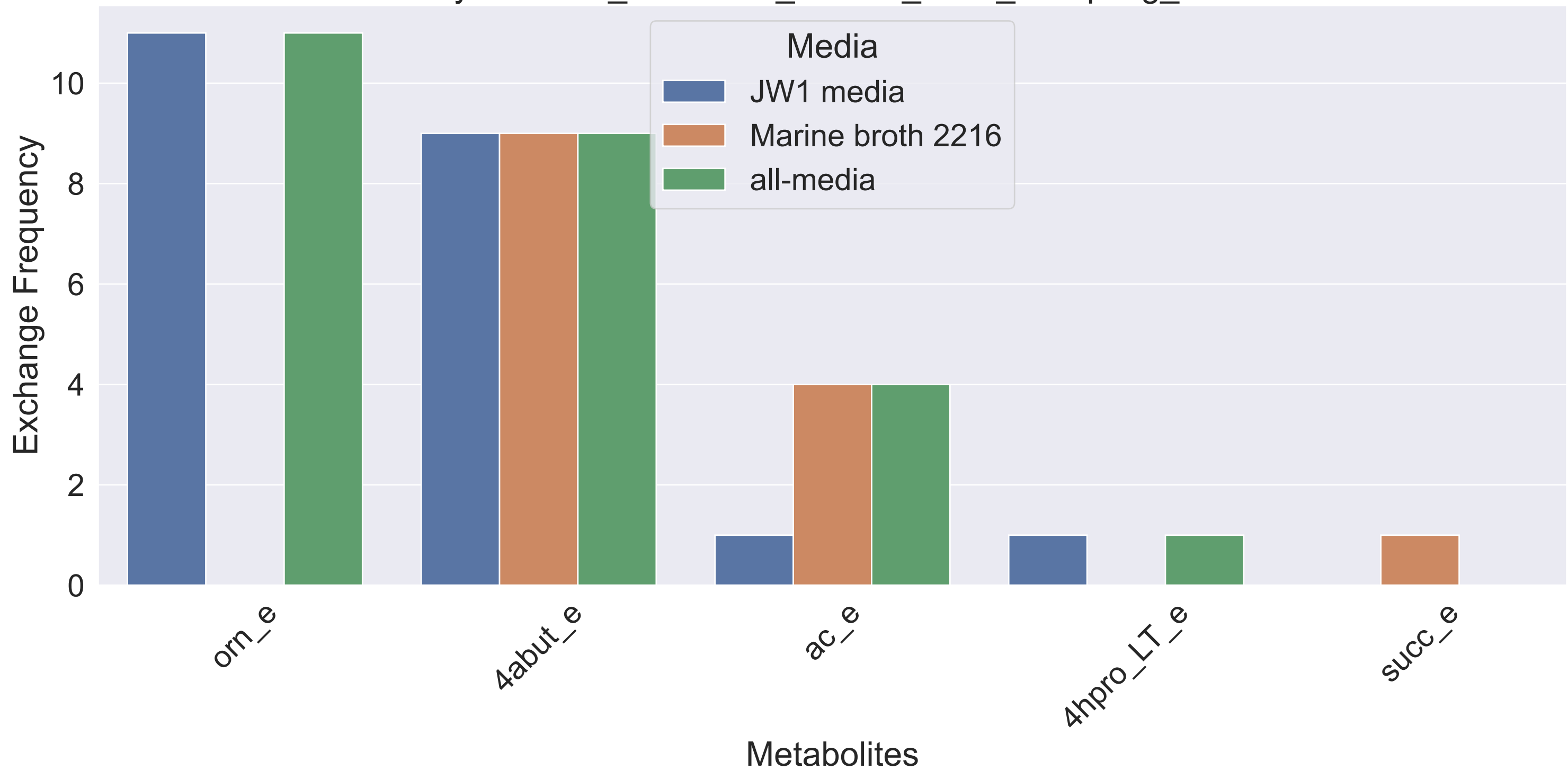

Supplement: Supplementary file 6 — Supplementary File S5 [file 43705_2023_242_MOESM6_ESM.zip › Supplementary Figure S85 Planctomycetaceae bacterium UWMA 0346 accepting metabolites.pdf]

Planctomycetes\_bacterium\_UWMA\_0265\_accepting\_metabolites

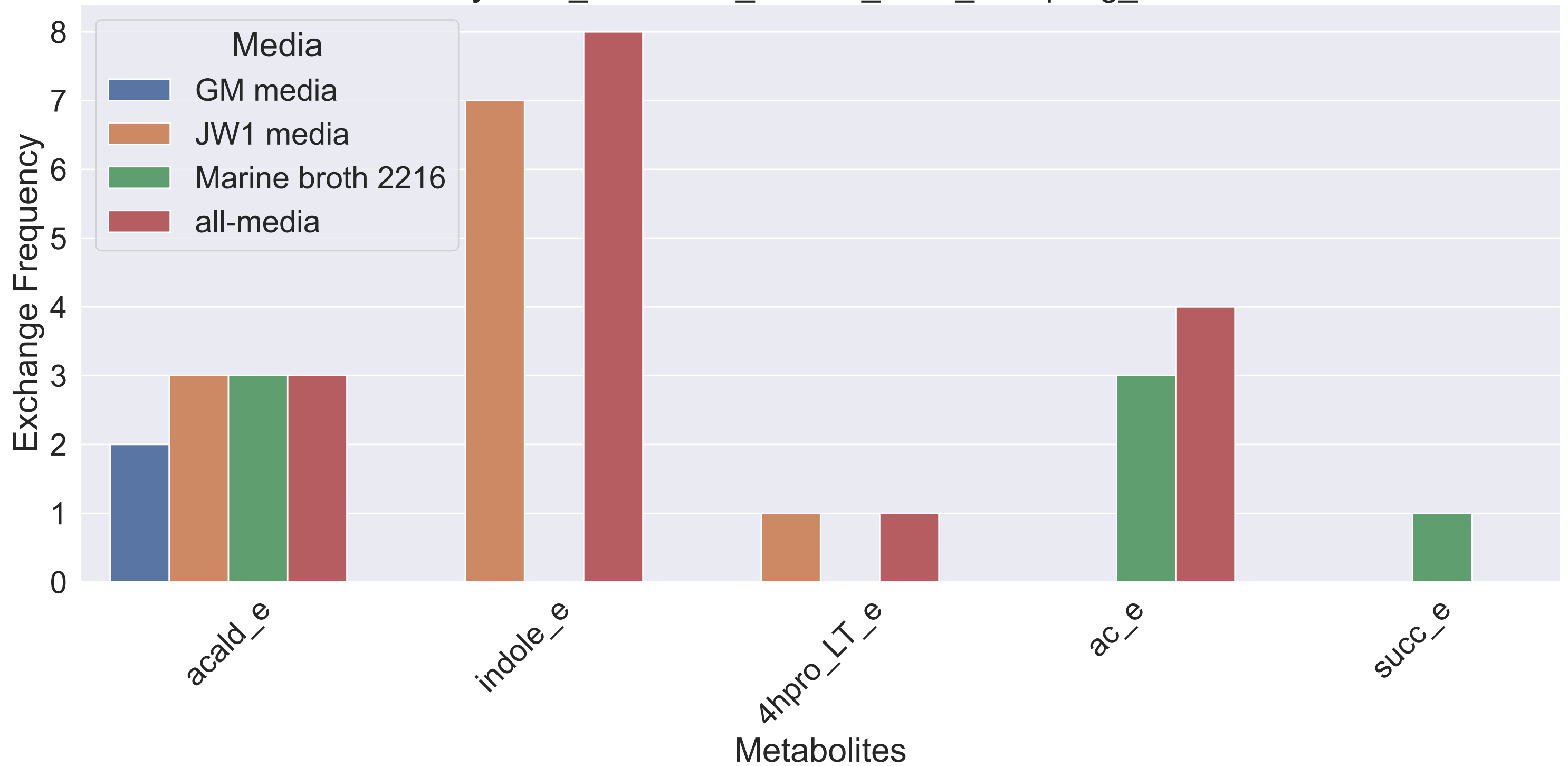

Supplement: Supplementary file 6 — Supplementary File S5 [file 43705_2023_242_MOESM6_ESM.zip › Supplementary Figure S86 Planctomycetes bacterium UWMA 0265 accepting metabolites.pdf]

# Planctomycetes\_bacterium\_UWMA\_0276\_accepting\_metabolites

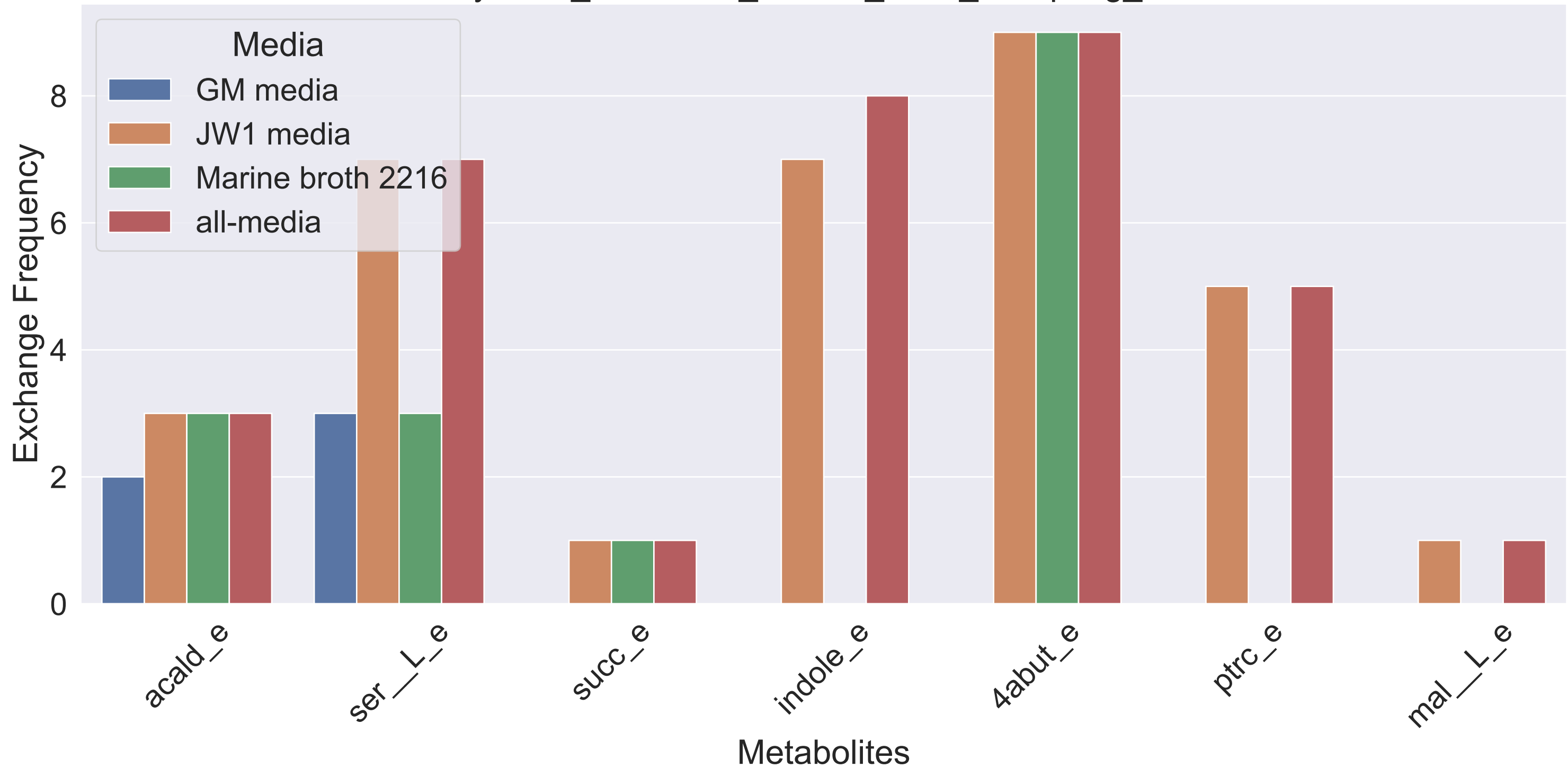

Supplement: Supplementary file 6 — Supplementary File S5 [file 43705_2023_242_MOESM6_ESM.zip › Supplementary Figure S87 Planctomycetes bacterium UWMA 0276 accepting metabolites.pdf]

# Planctomycetes\_bacterium\_UWMA\_0294\_accepting\_metabolites

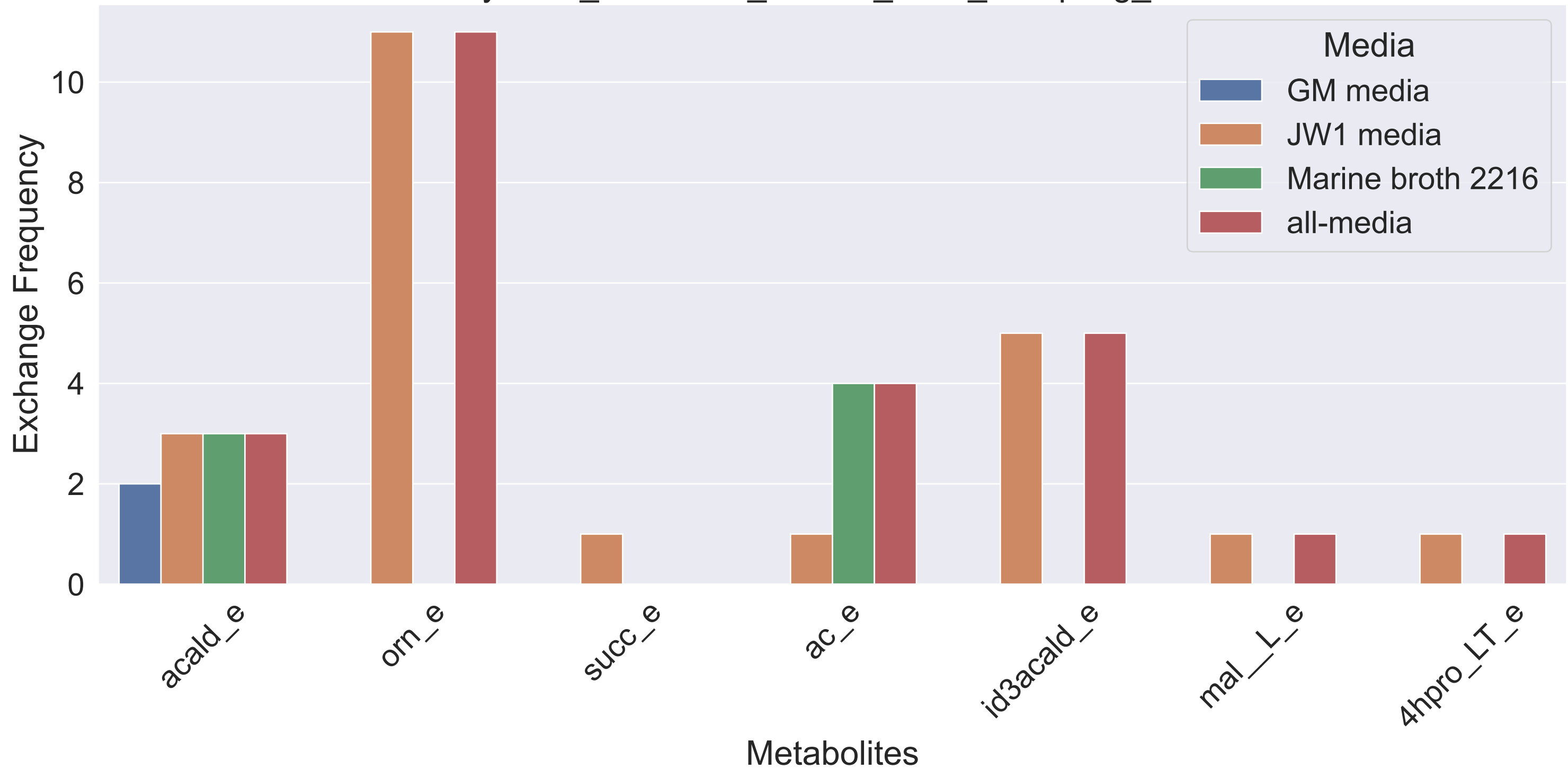

Supplement: Supplementary file 6 — Supplementary File S5 [file 43705_2023_242_MOESM6_ESM.zip › Supplementary Figure S88 Planctomycetes bacterium UWMA 0294 accepting metabolites.pdf]

Planctomycetes\_bacterium\_UWMA\_0329\_accepting\_metabolites

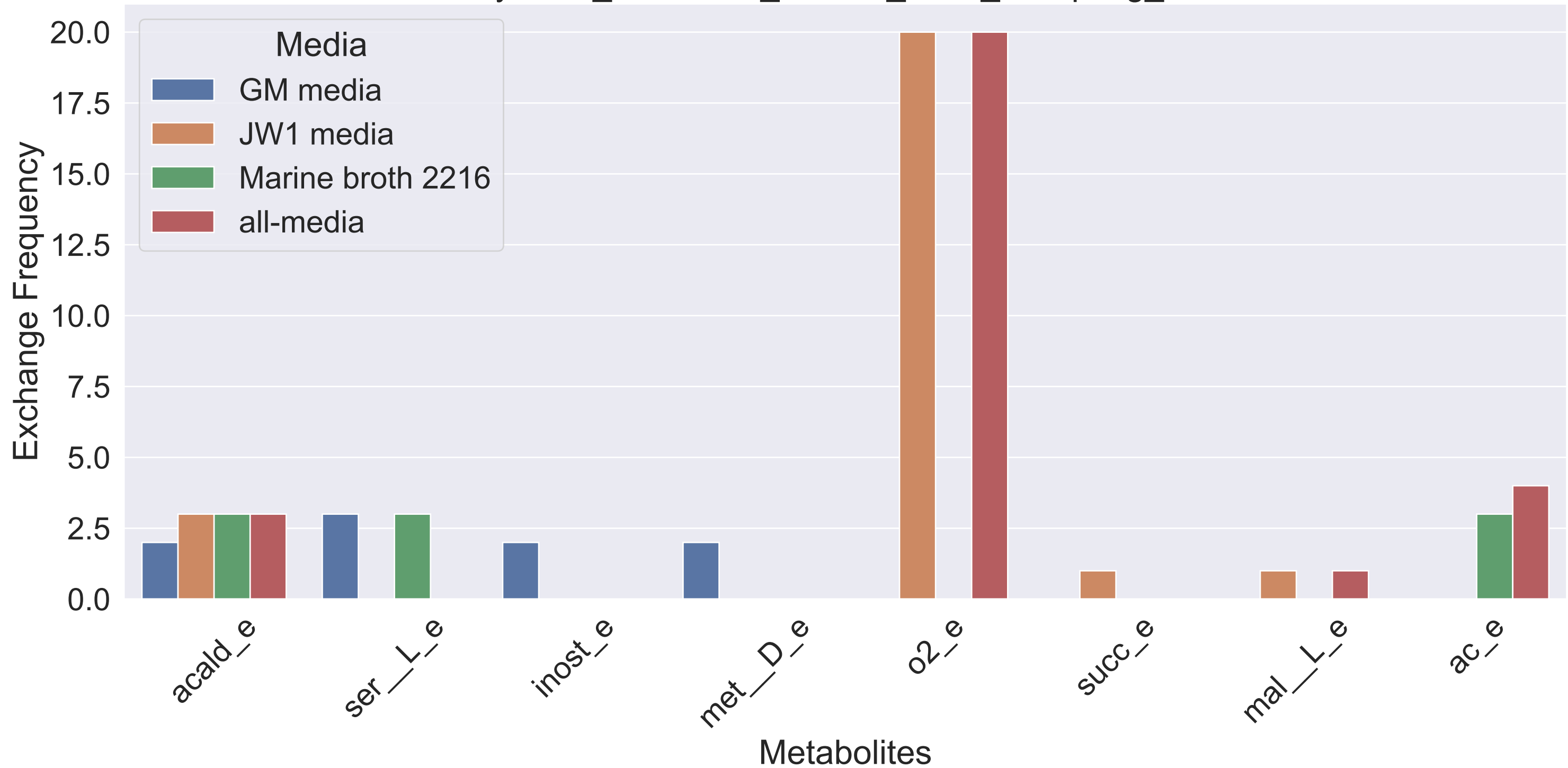

Supplement: Supplementary file 6 — Supplementary File S5 [file 43705_2023_242_MOESM6_ESM.zip › Supplementary Figure S89 Planctomycetes bacterium UWMA 0329 accepting metabolites.pdf]

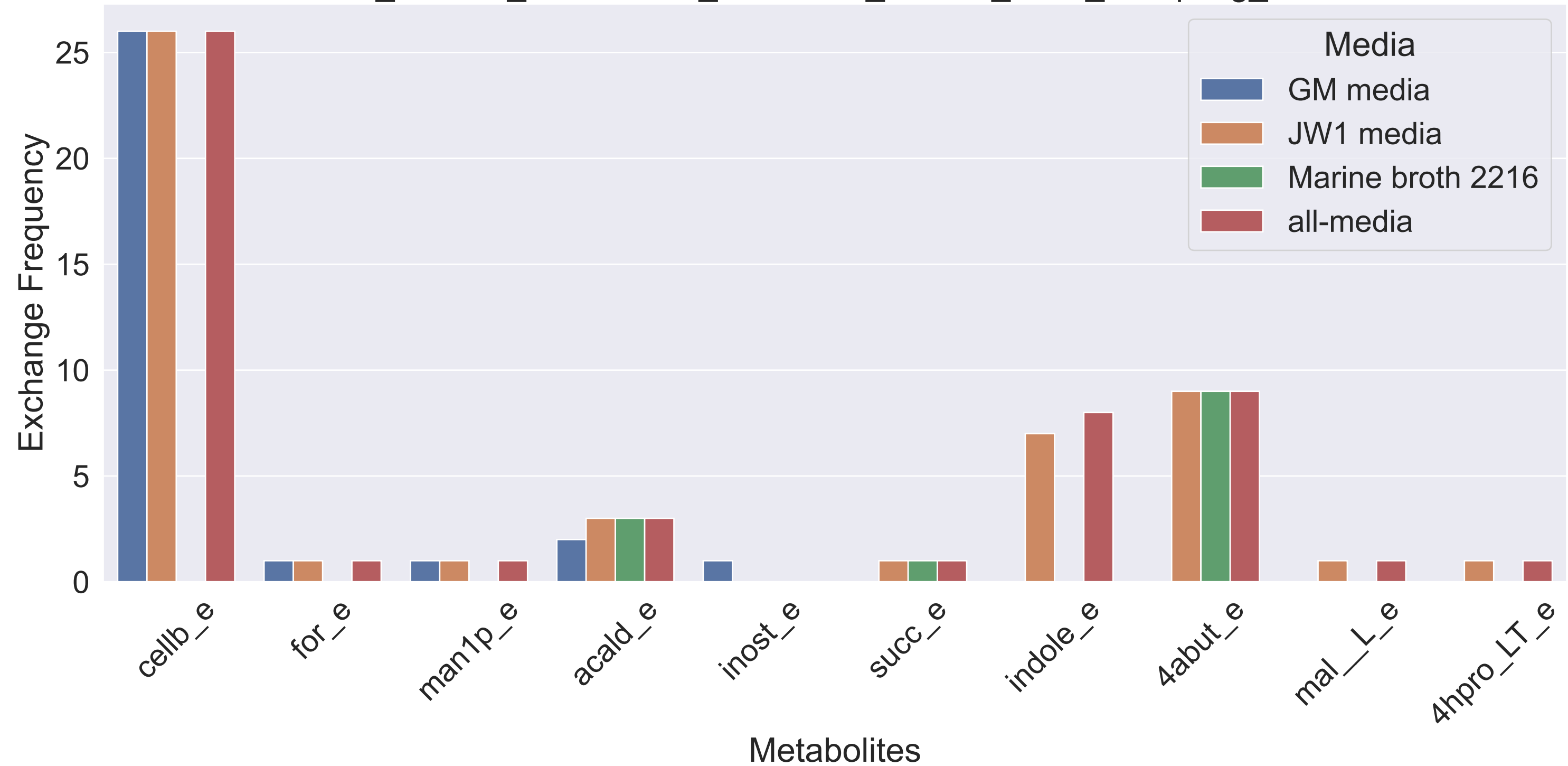

Supplement: Supplementary file 6 — Supplementary File S5 [file 43705_2023_242_MOESM6_ESM.zip › Supplementary Figure S9 candidate division Zixibacteria bacterium UWMA 0288 accepting metabolites.pdf]

Planctomycetes\_bacterium\_UWMA\_0345\_accepting\_metabolites

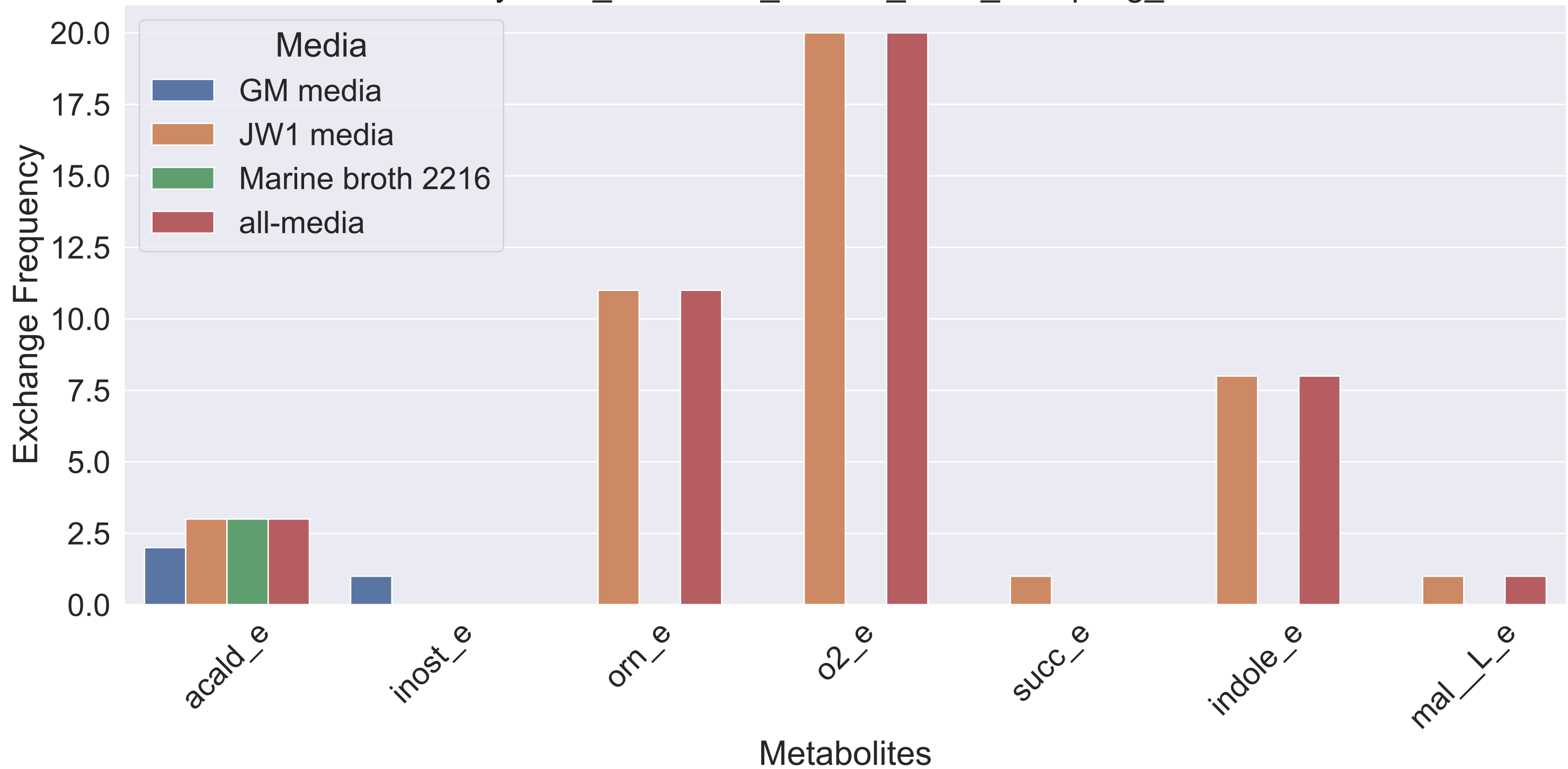

Supplement: Supplementary file 6 — Supplementary File S5 [file 43705_2023_242_MOESM6_ESM.zip › Supplementary Figure S90 Planctomycetes bacterium UWMA 0345 accepting metabolites.pdf]

# Porticoccaceae\_bacterium\_UWMA\_0313\_accepting\_metabolites

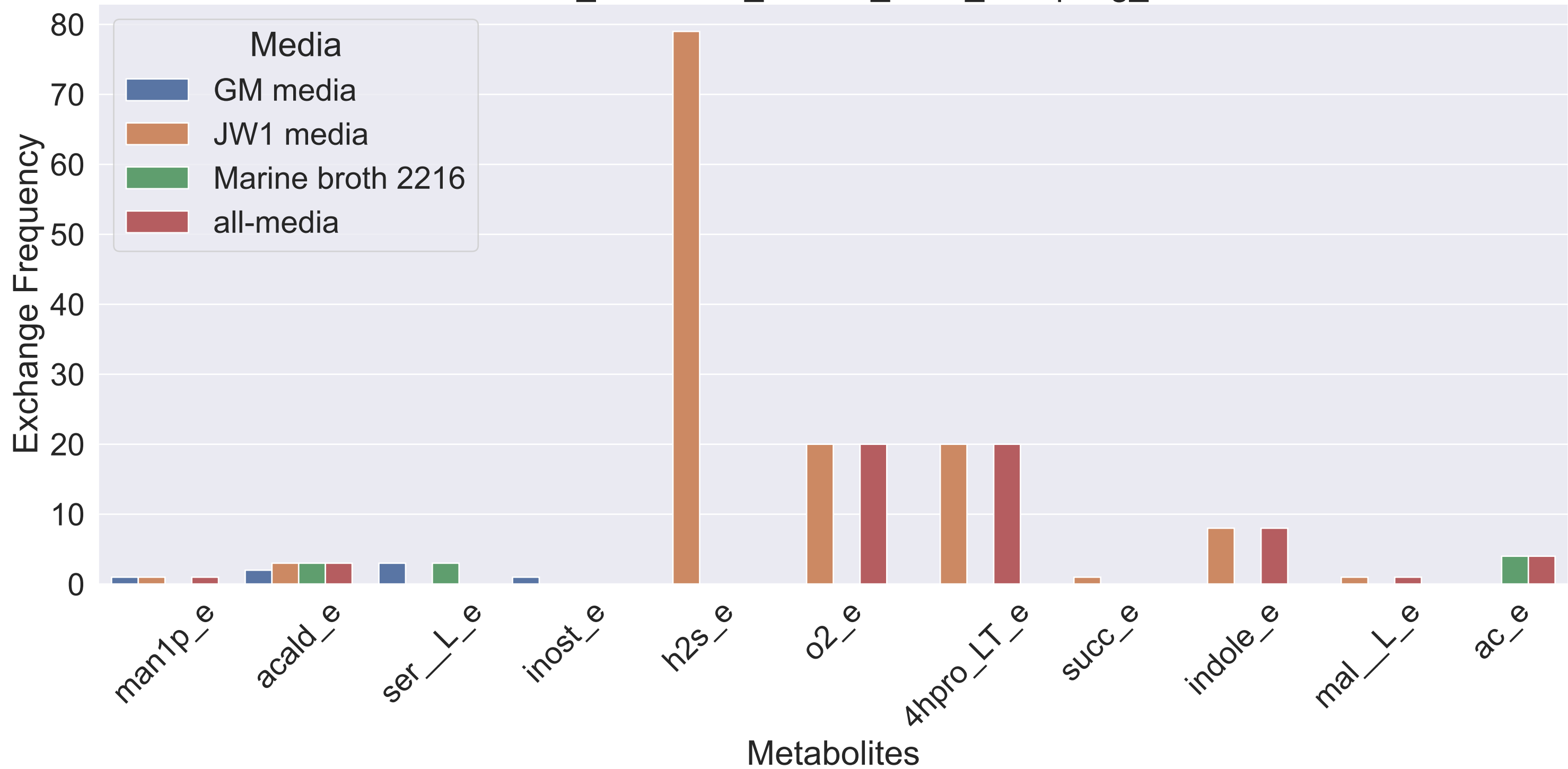

Supplement: Supplementary file 6 — Supplementary File S5 [file 43705_2023_242_MOESM6_ESM.zip › Supplementary Figure S91 Porticoccaceae bacterium UWMA 0313 accepting metabolites.pdf]

## Rhodospirillales\_bacterium\_UWMA\_0295\_accepting\_metabolites

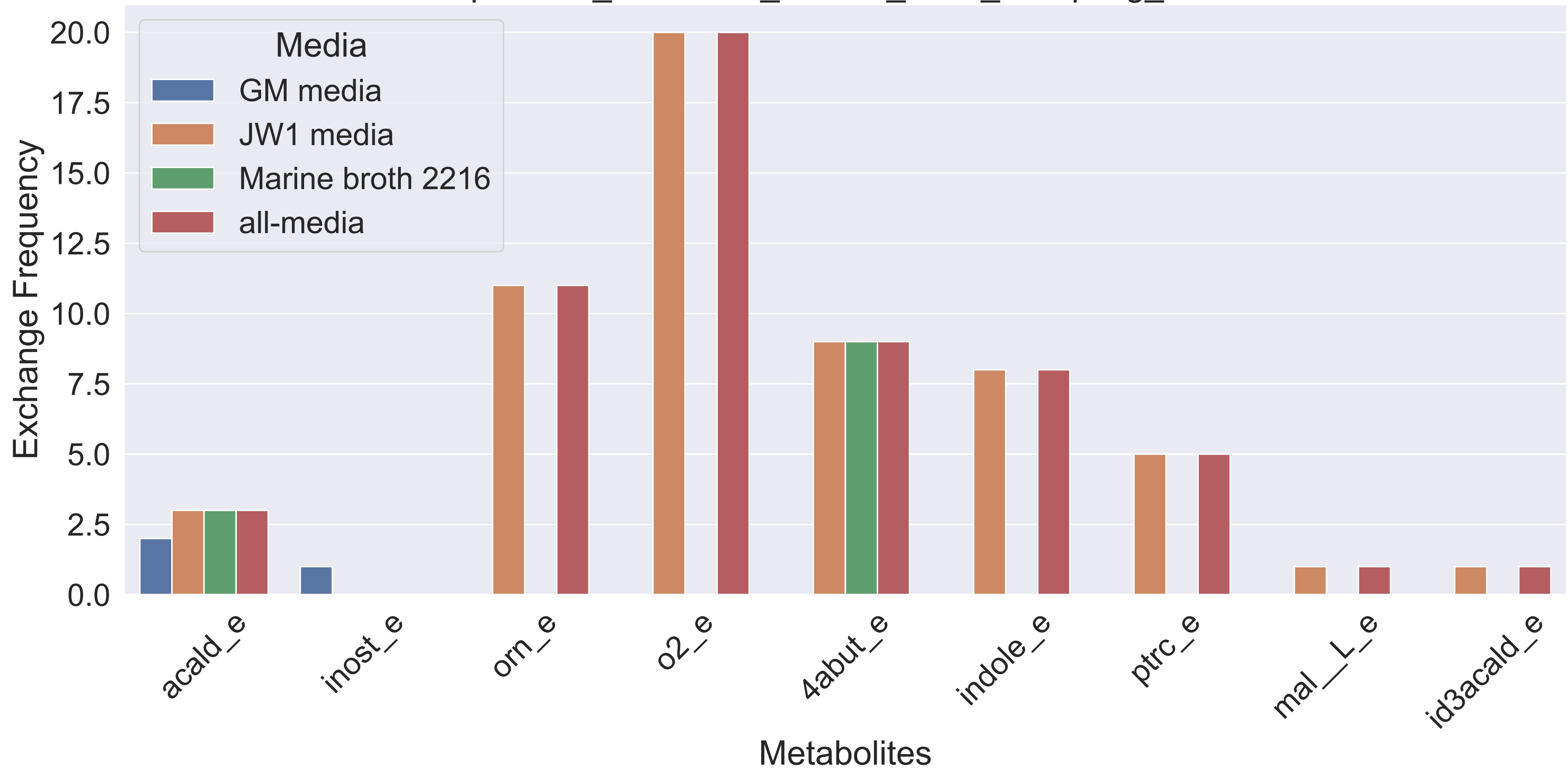

Supplement: Supplementary file 6 — Supplementary File S5 [file 43705_2023_242_MOESM6_ESM.zip › Supplementary Figure S92 Rhodospirillales bacterium UWMA 0295 accepting metabolites.pdf]

# Sneathiellales\_bacterium\_UWMA\_0353\_accepting\_metabolites

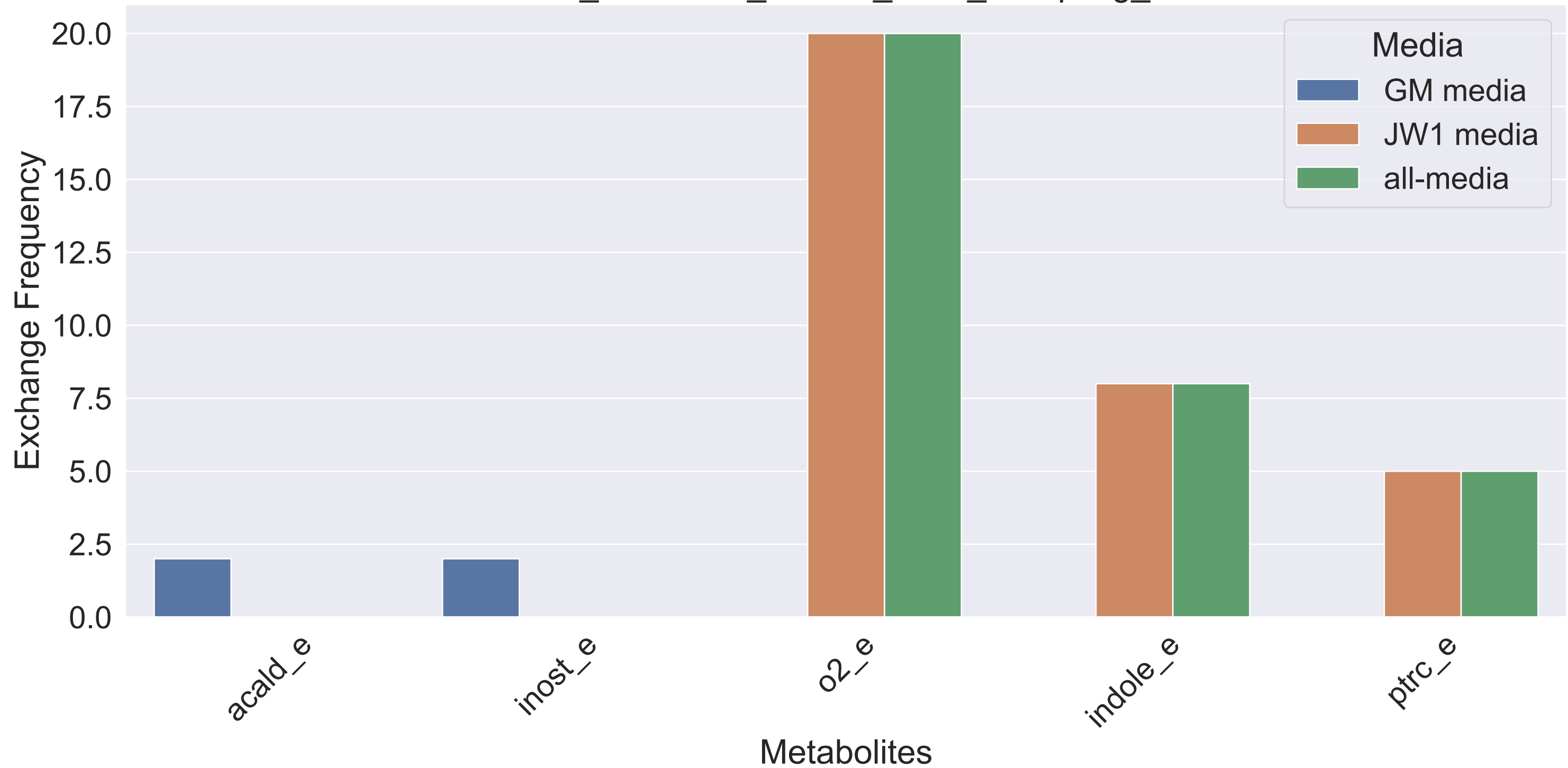

Supplement: Supplementary file 6 — Supplementary File S5 [file 43705_2023_242_MOESM6_ESM.zip › Supplementary Figure S93 Sneathiellales bacterium UWMA 0353 accepting metabolites.pdf]

# Sulfitobacter\_sp\_UWMA\_0305\_accepting\_metabolites

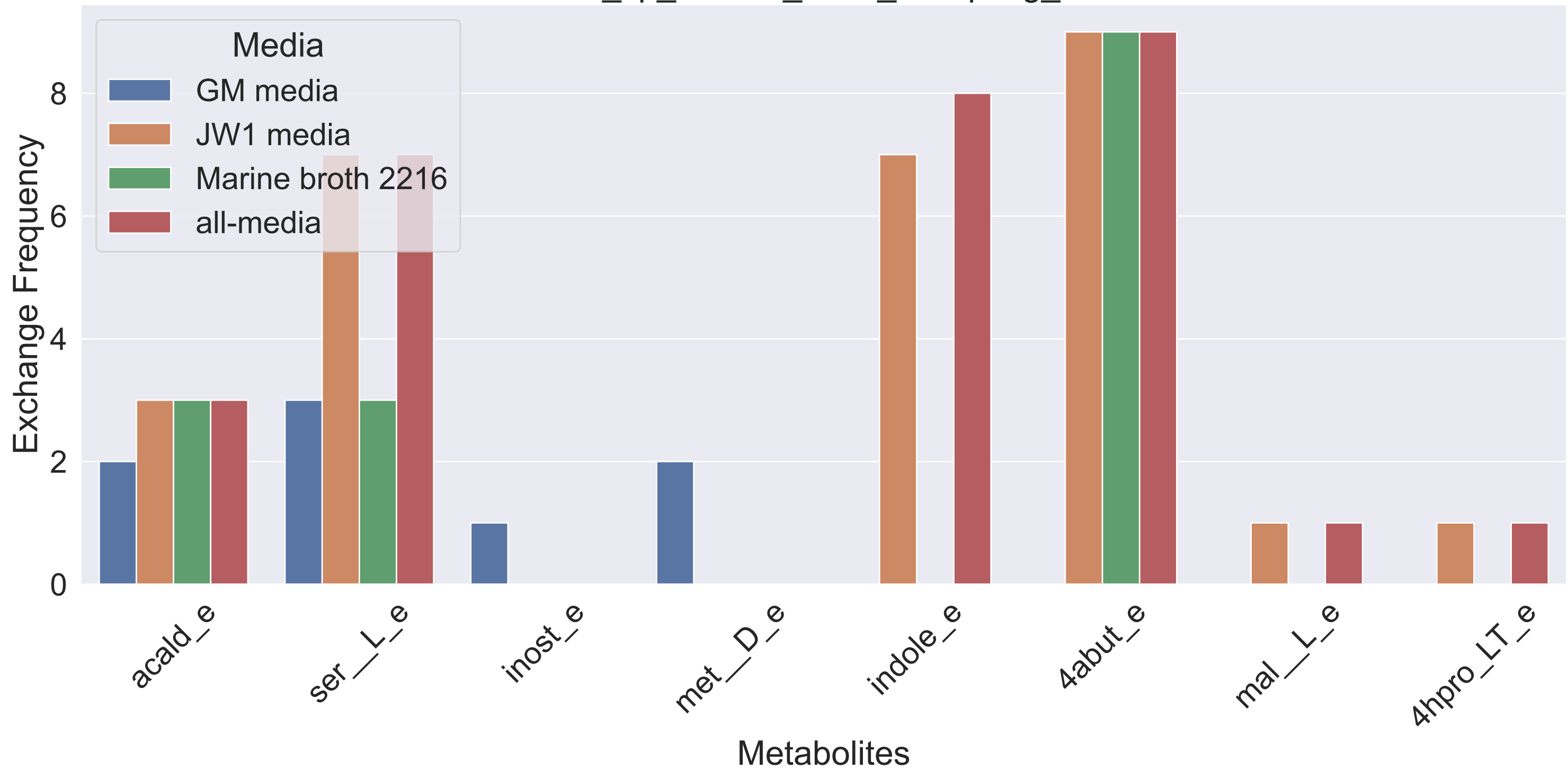

Supplement: Supplementary file 6 — Supplementary File S5 [file 43705_2023_242_MOESM6_ESM.zip › Supplementary Figure S94 Sulfitobacter sp UWMA 0305 accepting metabolites.pdf]

# Thiotrichaceae\_bacterium\_UWMA\_0311\_accepting\_metabolites

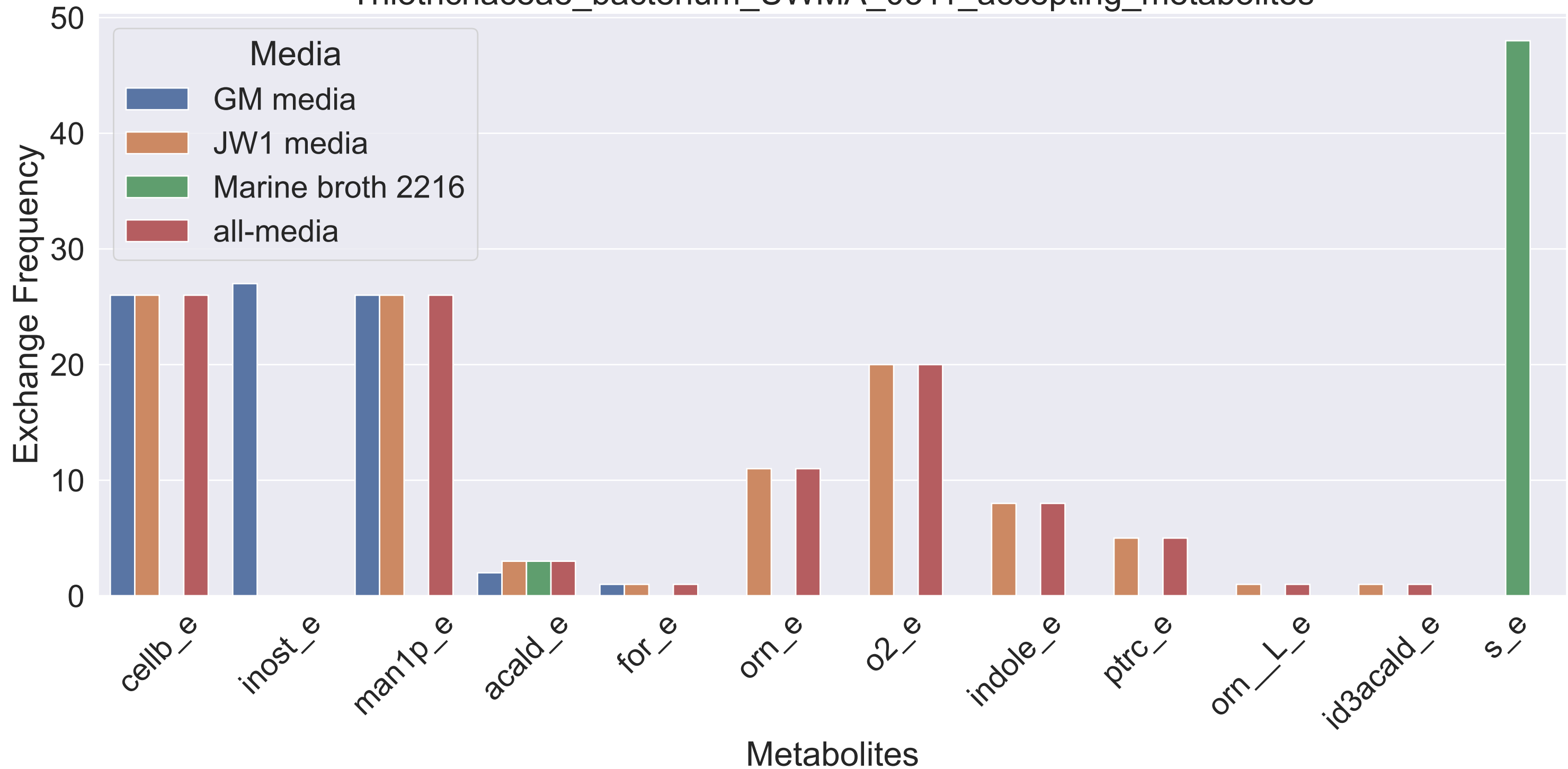

Supplement: Supplementary file 6 — Supplementary File S5 [file 43705_2023_242_MOESM6_ESM.zip › Supplementary Figure S95 Thiotrichaceae bacterium UWMA 0311 accepting metabolites.pdf]

# Verrucomicrobiales\_bacterium\_UWMA\_0292\_accepting\_metabolites

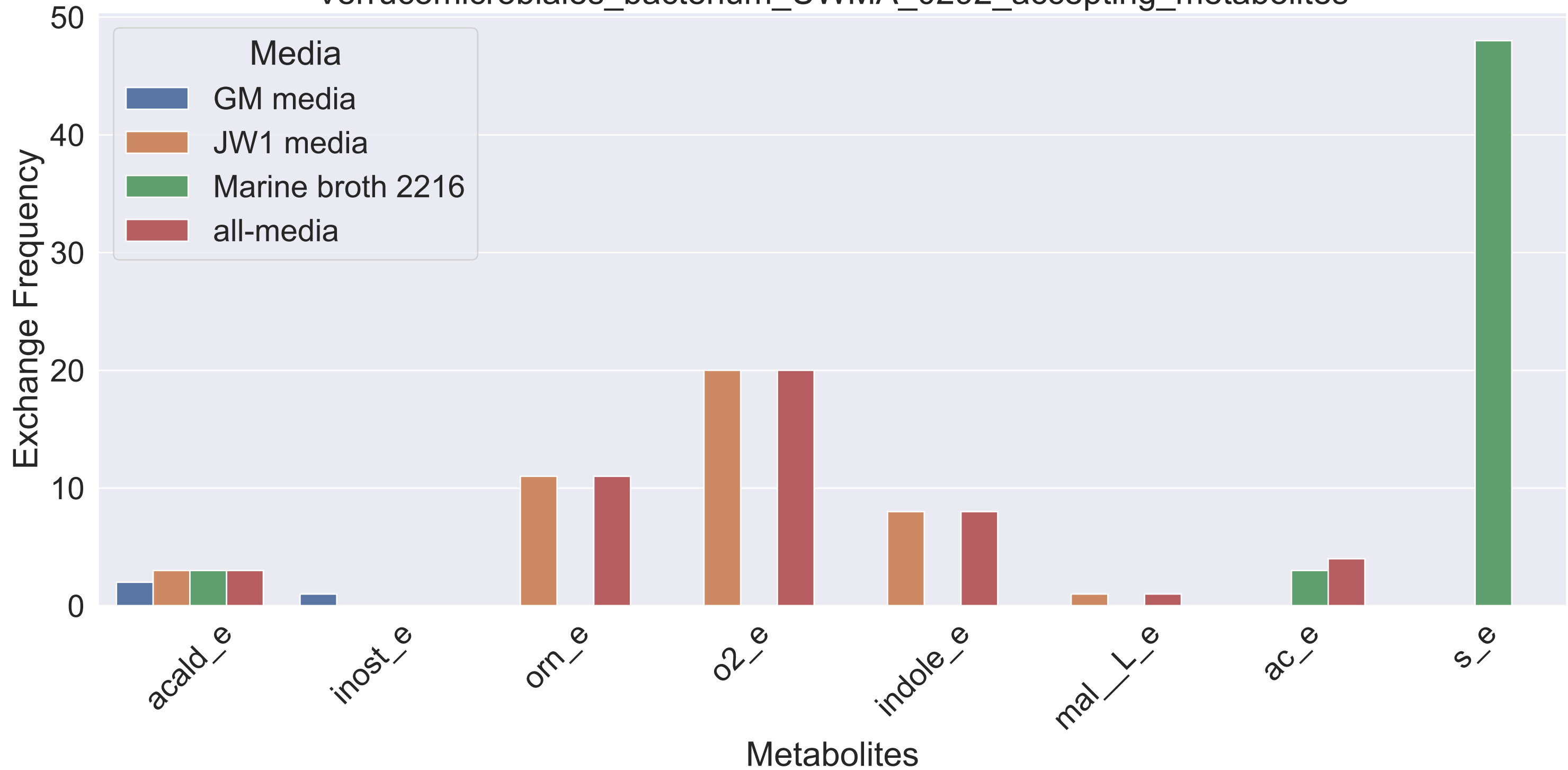

Supplement: Supplementary file 6 — Supplementary File S5 [file 43705_2023_242_MOESM6_ESM.zip › Supplementary Figure S96 Verrucomicrobiales bacterium UWMA 0292 accepting metabolites.pdf]

# Verrucomicrobiales\_bacterium\_UWMA\_0320\_accepting\_metabolites

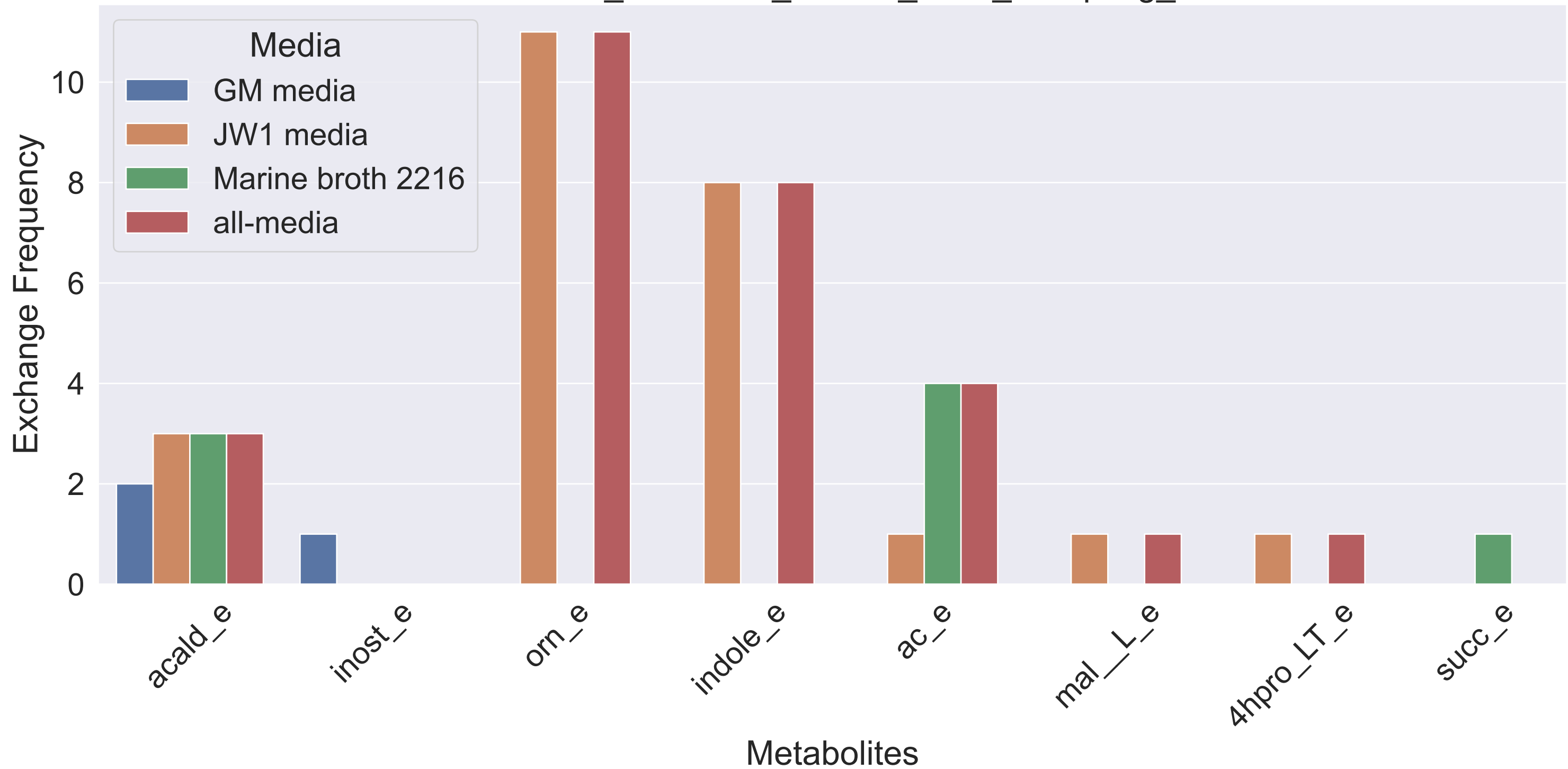

Supplement: Supplementary file 6 — Supplementary File S5 [file 43705_2023_242_MOESM6_ESM.zip › Supplementary Figure S97 Verrucomicrobiales bacterium UWMA 0320 accepting metabolites.pdf]

# Verrucomicrobiales\_bacterium\_UWMA\_0332\_accepting\_metabolites

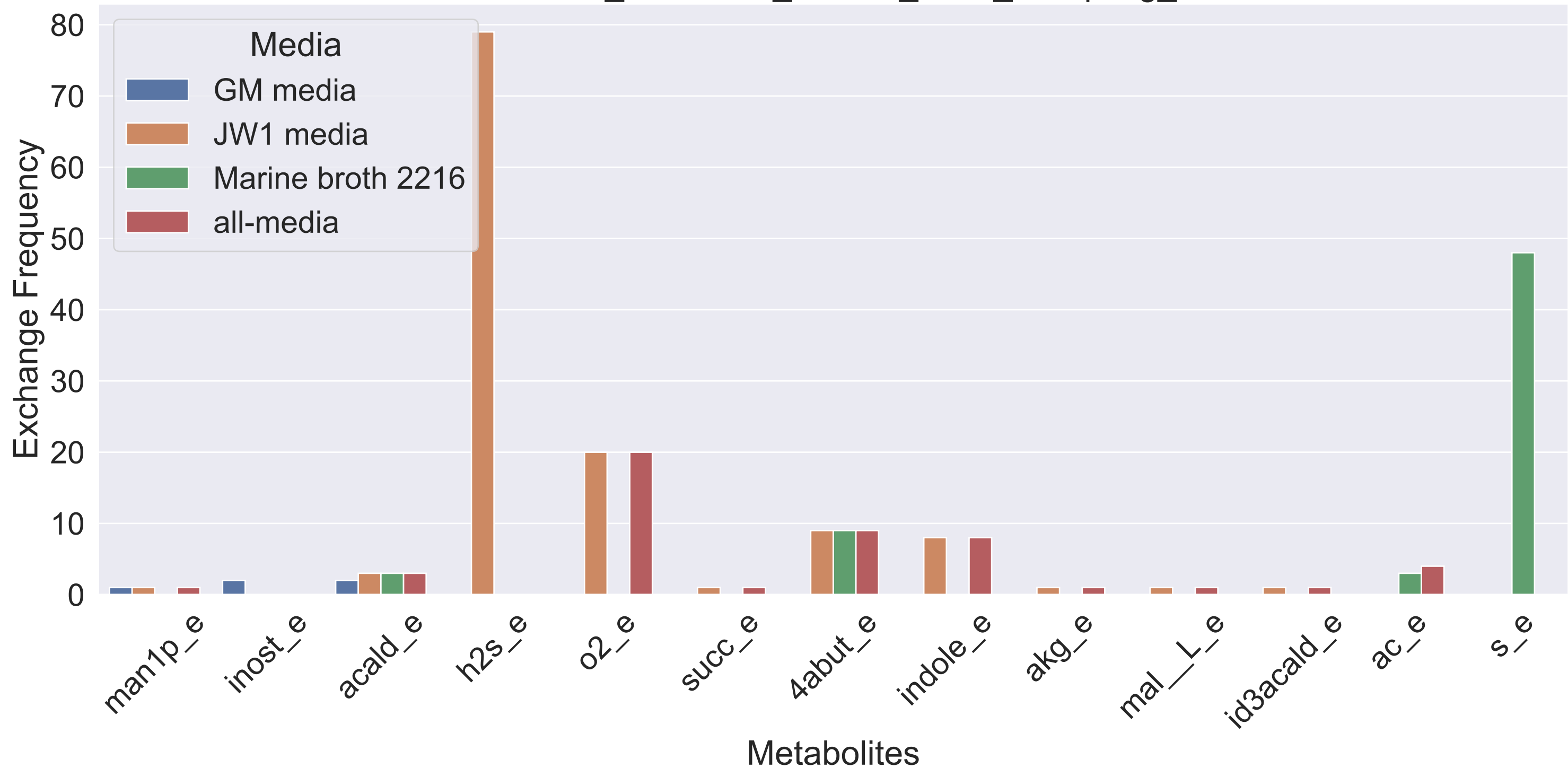

Supplement: Supplementary file 6 — Supplementary File S5 [file 43705_2023_242_MOESM6_ESM.zip › Supplementary Figure S98 Verrucomicrobiales bacterium UWMA 0332 accepting metabolites.pdf]

# Acidimicrobiia\_bacterium\_UWMA\_0303\_donating\_metabolites

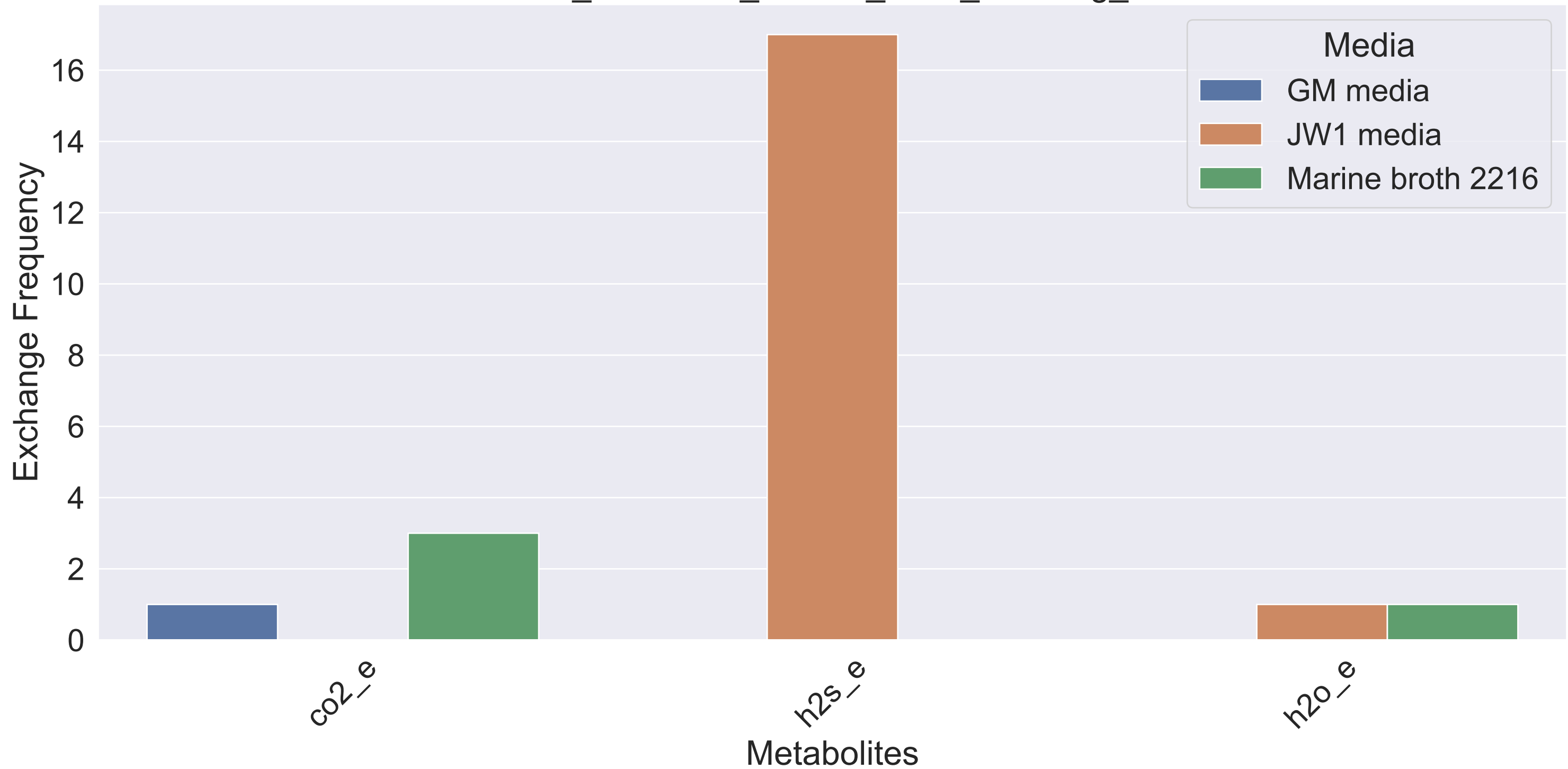

Supplement: Supplementary file 7 — Supplementary File S6 [file 43705_2023_242_MOESM7_ESM.zip › Supplementary Figure S100 Acidimicrobiia bacterium UWMA 0303 donating metabolites.pdf]

# Acidimicrobiia\_bacterium\_UWMA\_0319\_donating\_metabolites

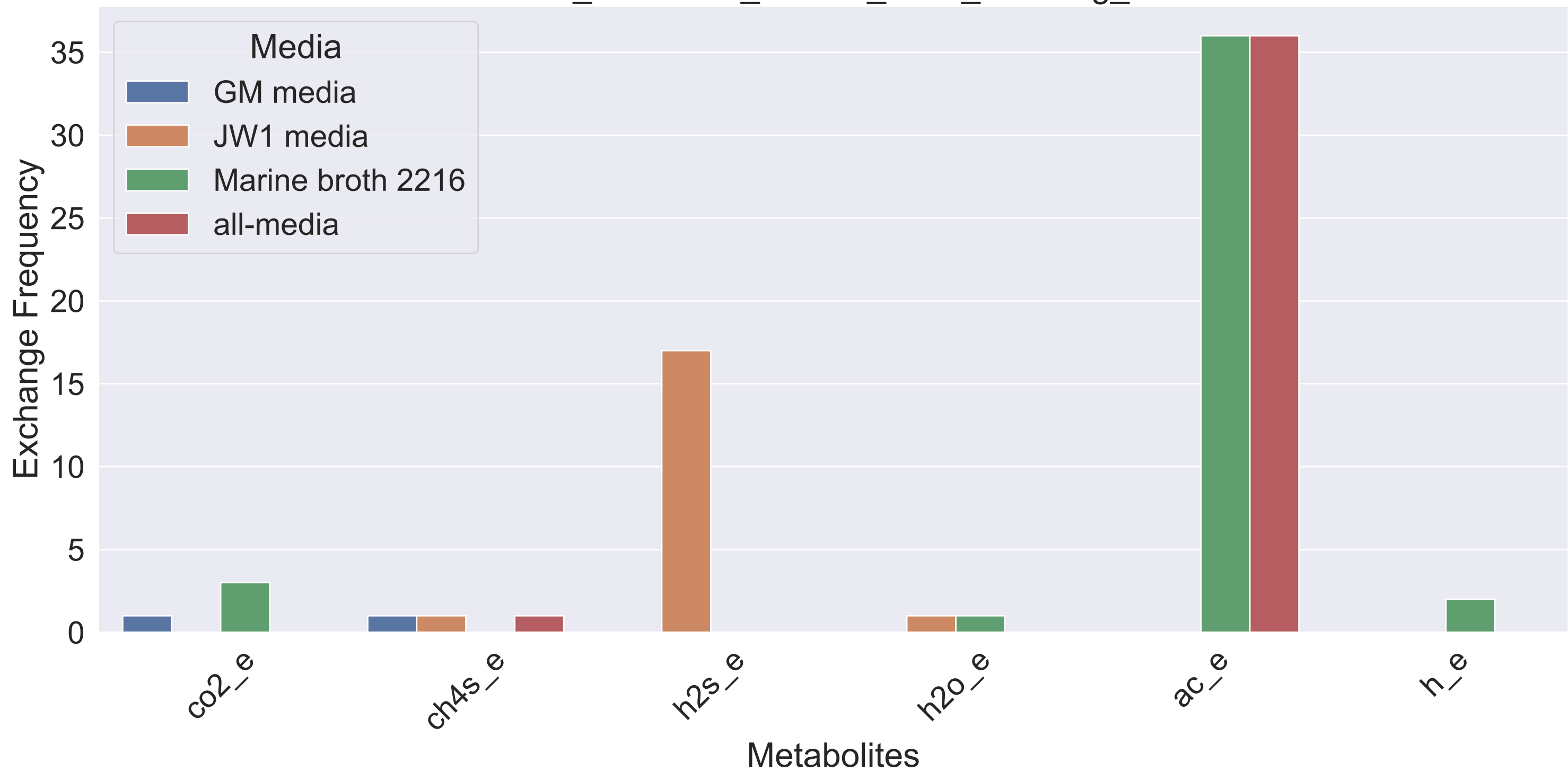

Supplement: Supplementary file 7 — Supplementary File S6 [file 43705_2023_242_MOESM7_ESM.zip › Supplementary Figure S101 Acidimicrobiia bacterium UWMA 0319 donating metabolites.pdf]
