# Supplementary figures and images for: Metagenome-based metabolic modelling predicts unique microbial interactions in deep-sea hydrothermal plume microbiomes (part 2 of 2)
Source: ISME Commun. 2023 Apr 29;3:42. doi: 10.1038/s43705-023-00242-8 (PMC10148797; doi:10.1038/s43705-023-00242-8)

# Acidobacteria\_bacterium\_UWMA\_0355\_donating\_metabolites

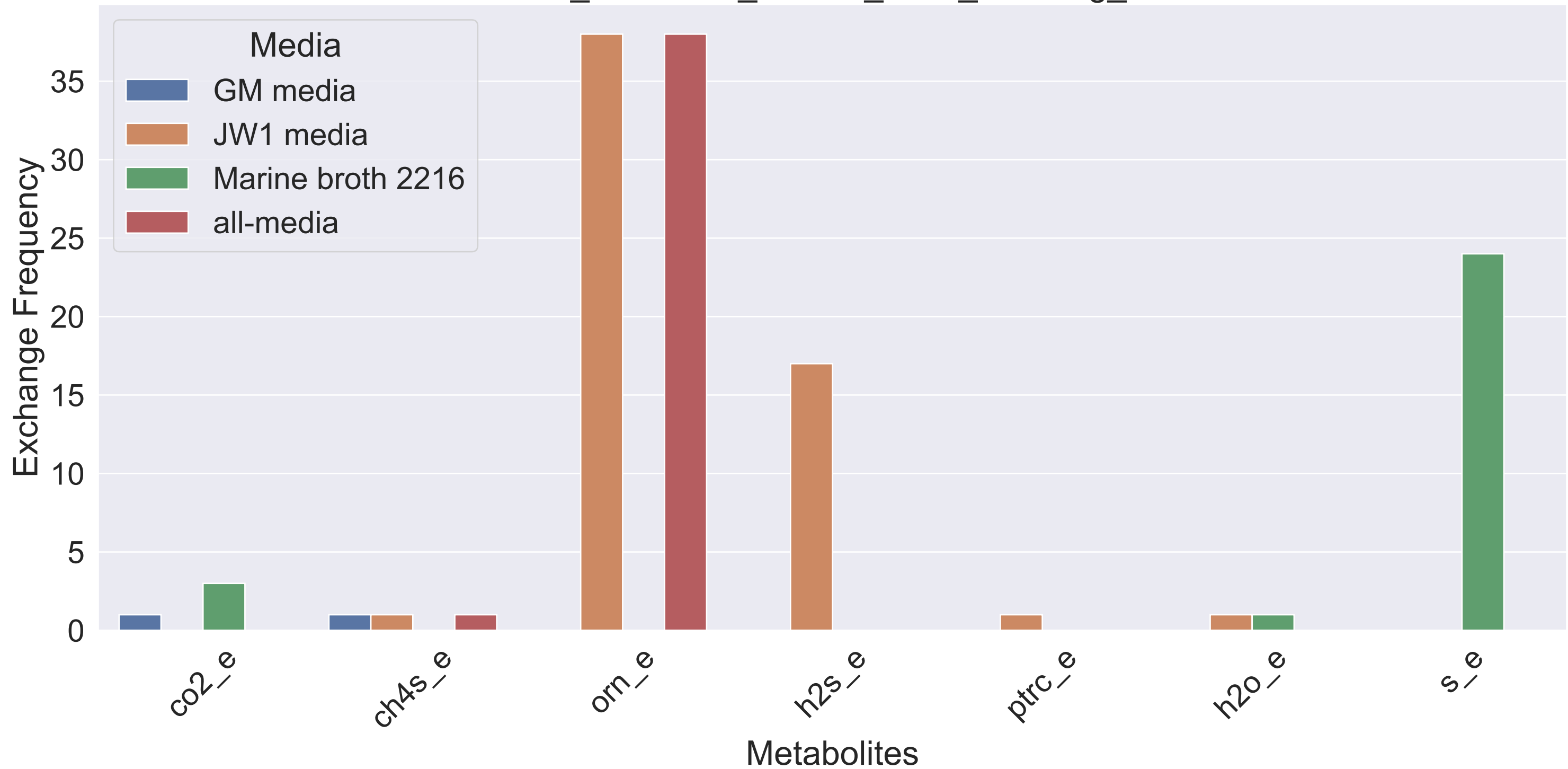

Supplement: Supplementary file 7 — Supplementary File S6 [file 43705_2023_242_MOESM7_ESM.zip › Supplementary Figure S102 Acidobacteria bacterium UWMA 0355 donating metabolites.pdf]

Alphaproteobacteria\_bacterium\_UWMA\_0321\_donating\_metabolites

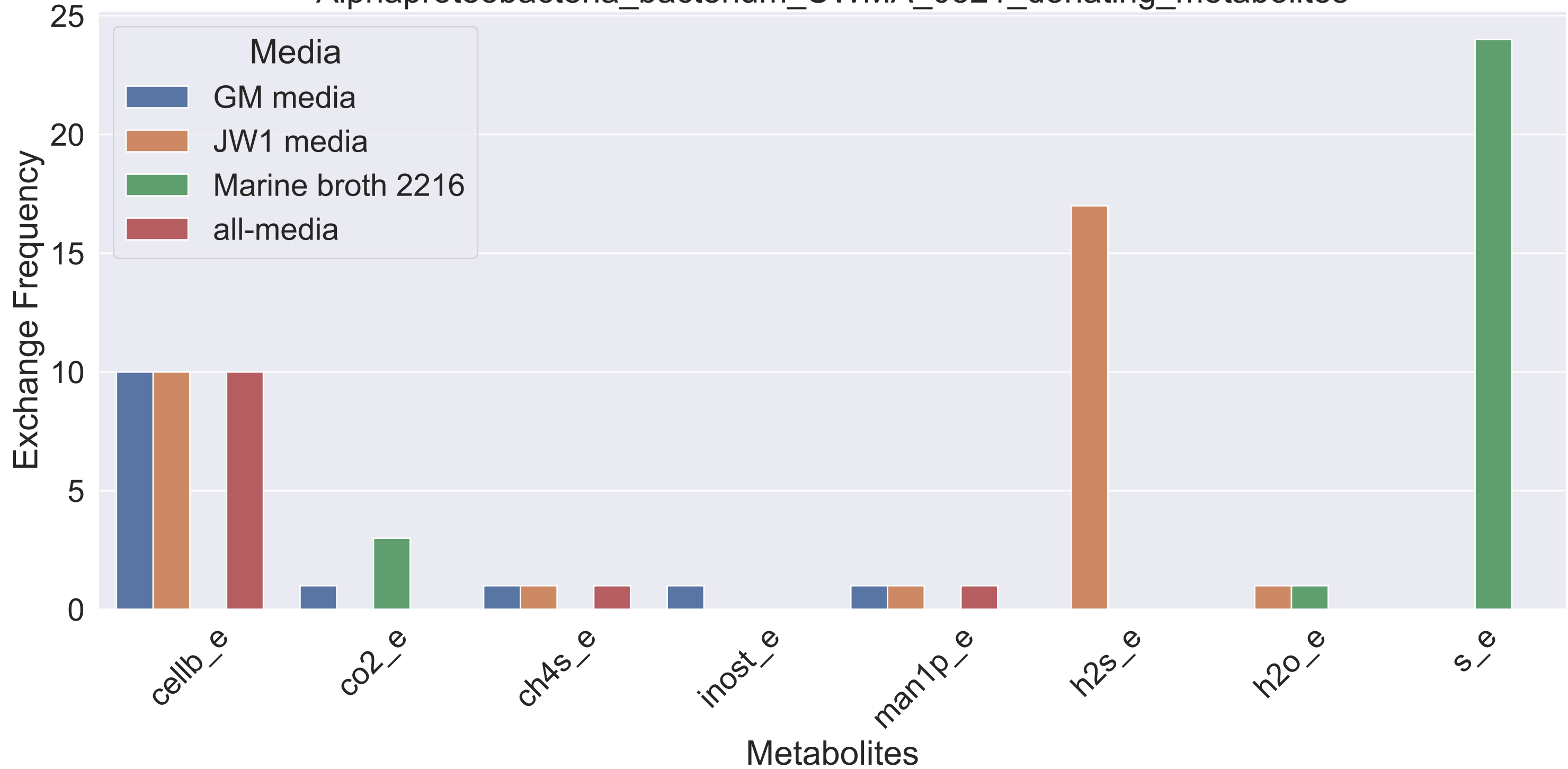

Supplement: Supplementary file 7 — Supplementary File S6 [file 43705_2023_242_MOESM7_ESM.zip › Supplementary Figure S103 Alphaproteobacteria bacterium UWMA 0321 donating metabolites.pdf]

Bacteroidetes\_bacterium\_UWMA\_0293\_donating\_metabolites

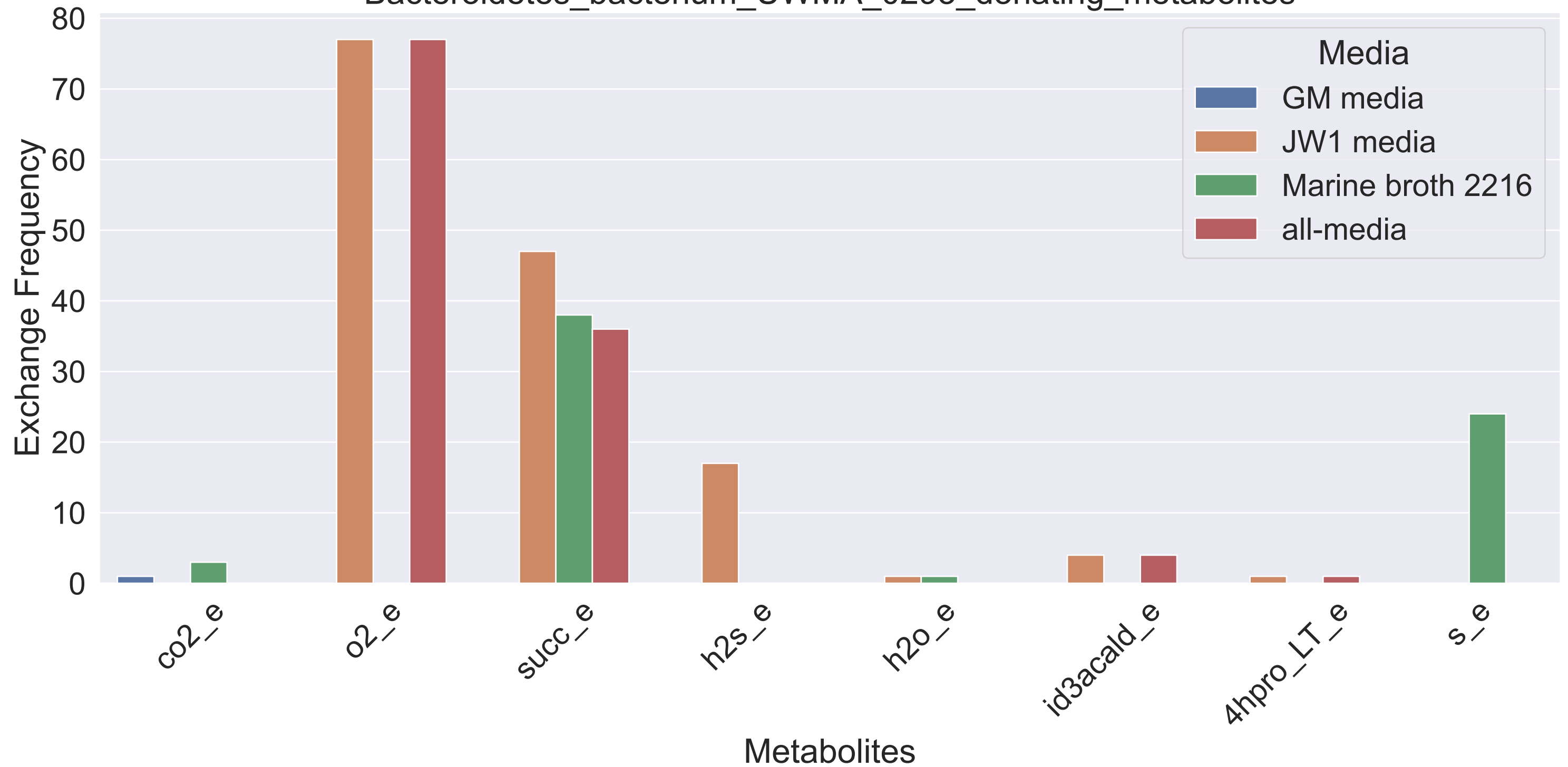

Supplement: Supplementary file 7 — Supplementary File S6 [file 43705_2023_242_MOESM7_ESM.zip › Supplementary Figure S104 Bacteroidetes bacterium UWMA 0293 donating metabolites.pdf]

# Bacteroidetes\_bacterium\_UWMA\_0358\_donating\_metabolites

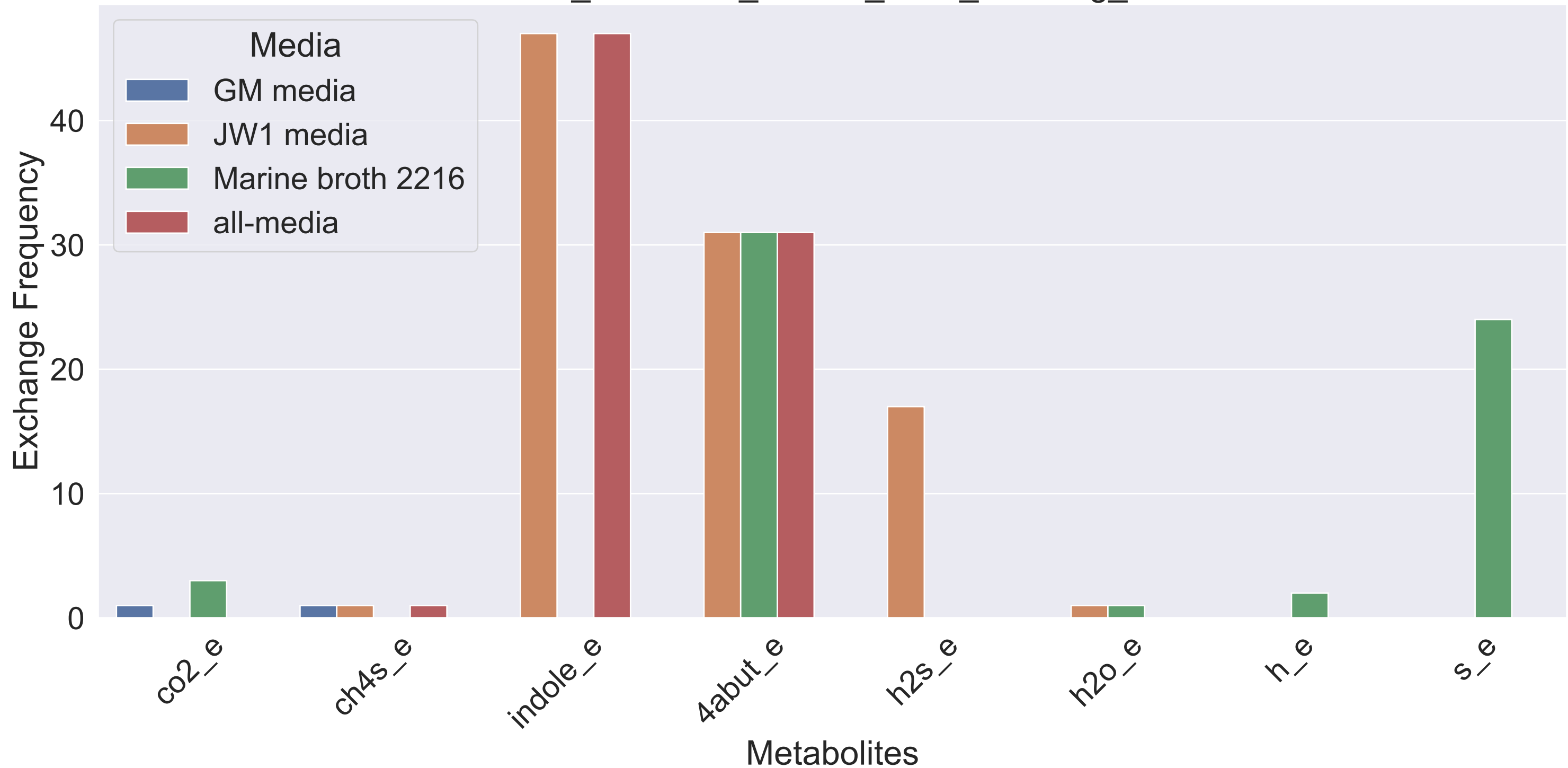

Supplement: Supplementary file 7 — Supplementary File S6 [file 43705_2023_242_MOESM7_ESM.zip › Supplementary Figure S105 Bacteroidetes bacterium UWMA 0358 donating metabolites.pdf]

# candidate\_division\_UBP10\_bacterium\_UWMA\_0304\_donating\_metabolites

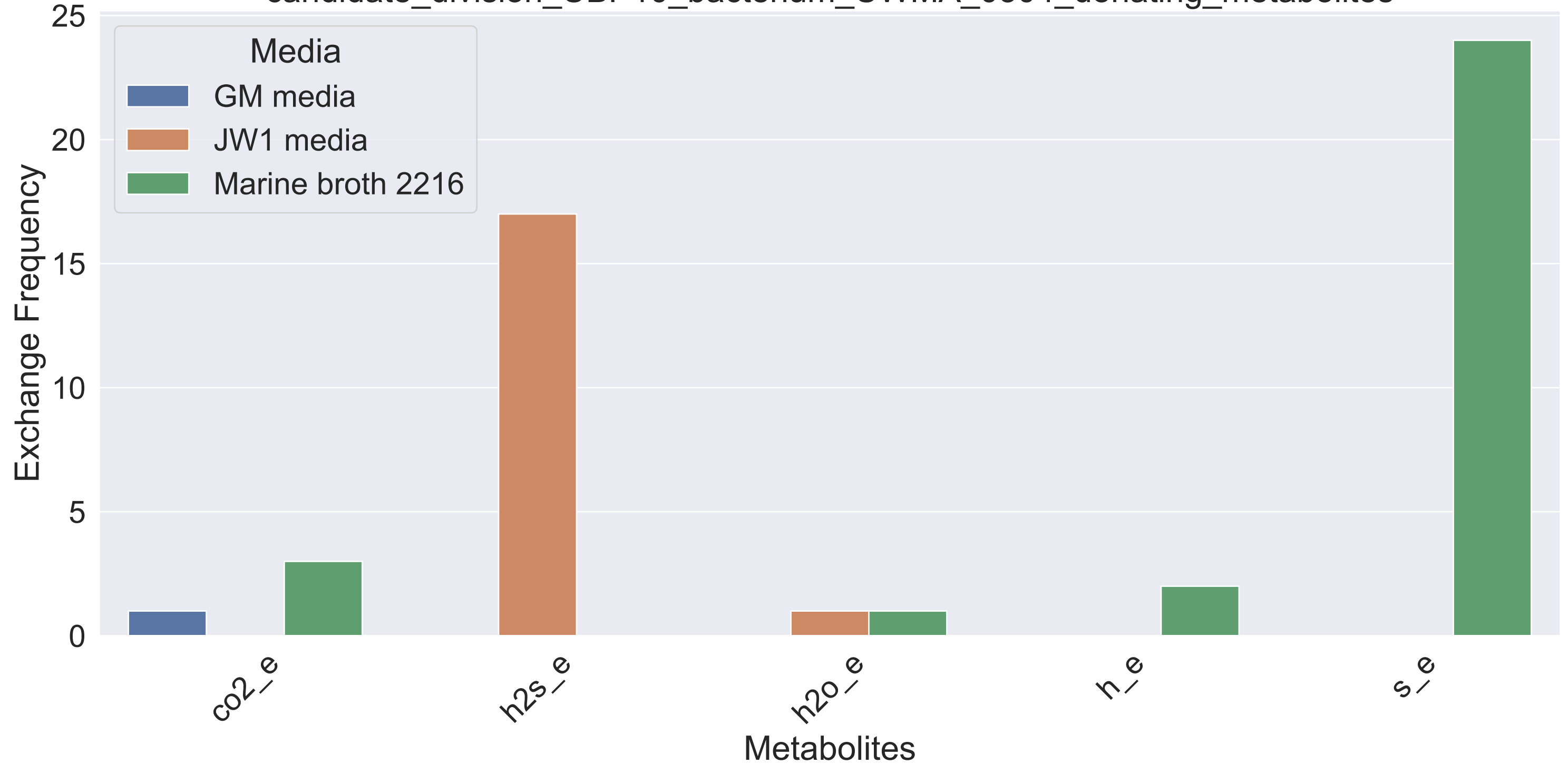

Supplement: Supplementary file 7 — Supplementary File S6 [file 43705_2023_242_MOESM7_ESM.zip › Supplementary Figure S106 candidate division UBP10 bacterium UWMA 0304 donating metabolites.pdf]

# candidate\_division\_Zixibacteria\_bacterium\_UWMA\_0288\_donating\_metabolites

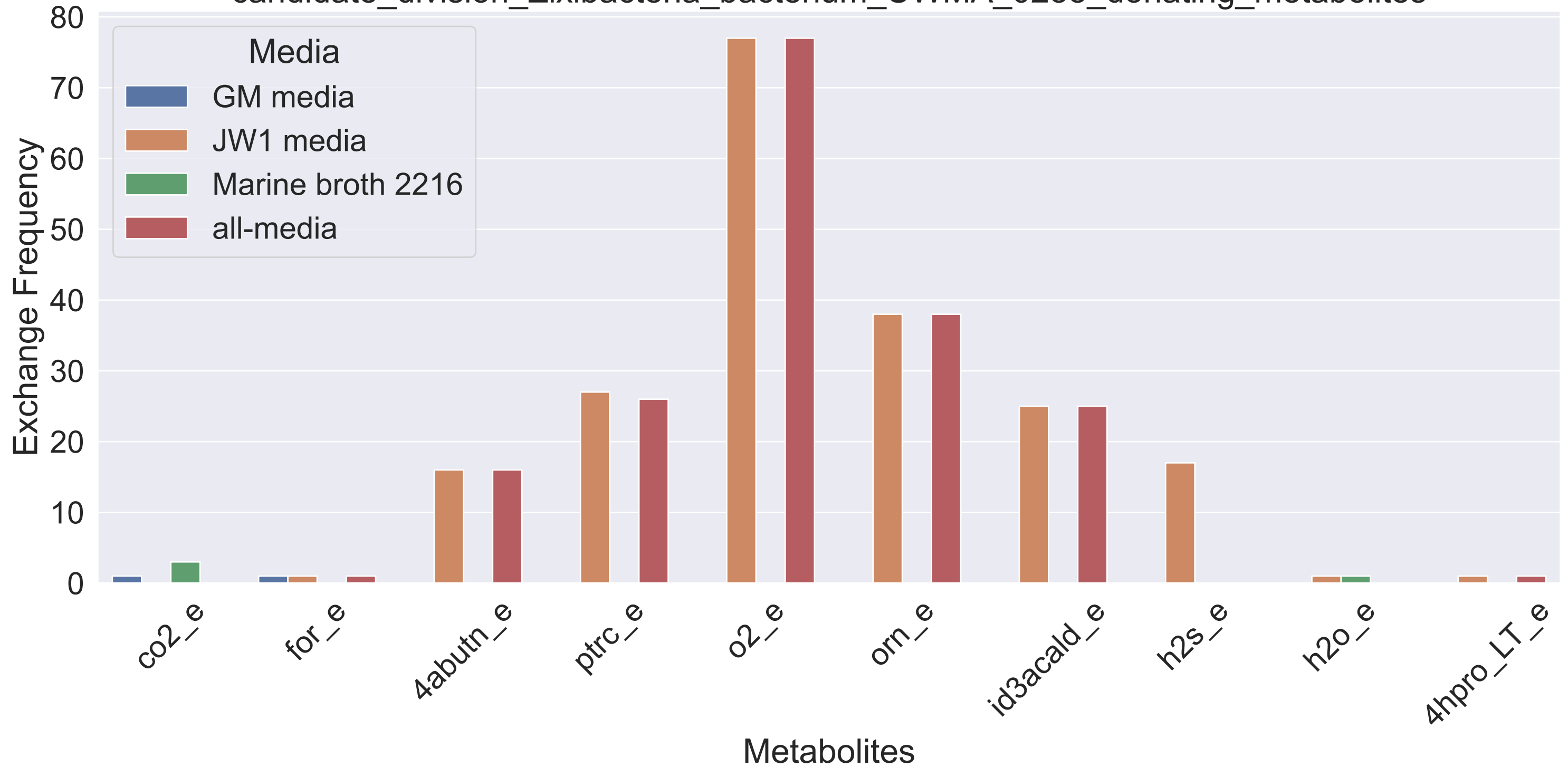

Supplement: Supplementary file 7 — Supplementary File S6 [file 43705_2023_242_MOESM7_ESM.zip › Supplementary Figure S107 candidate division Zixibacteria bacterium UWMA 0288 donating metabolites.pdf]

# Candidatus\_Handelsmanbacteria\_bacterium\_UWMA\_0286\_donating\_metabolites

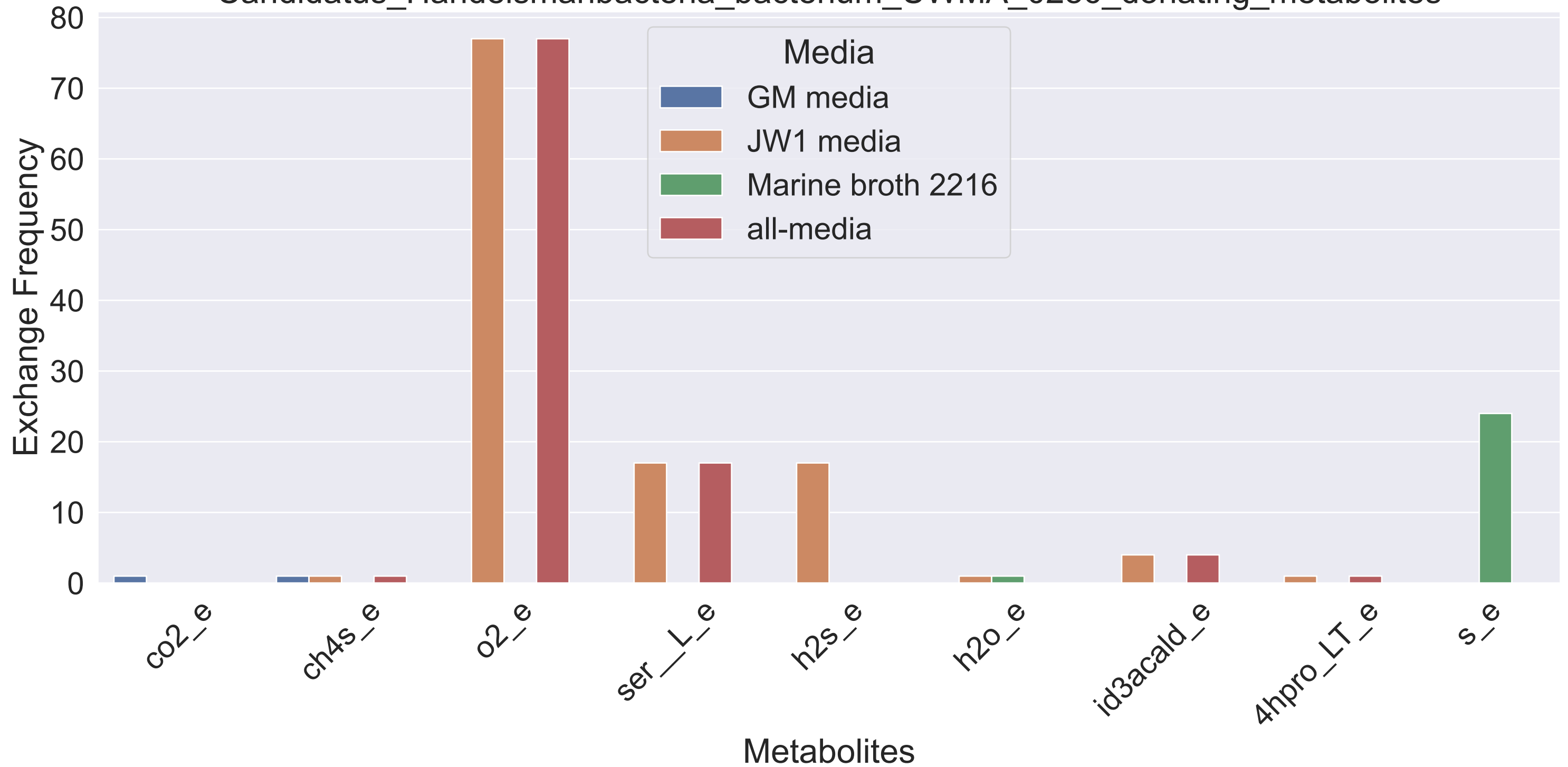

Supplement: Supplementary file 7 — Supplementary File S6 [file 43705_2023_242_MOESM7_ESM.zip › Supplementary Figure S108 Candidatus Handelsmanbacteria bacterium UWMA 0286 donating metabolites.pdf]

# Candidatus\_Handelsmanbacteria\_bacterium\_UWMA\_0300\_donating\_metabolites

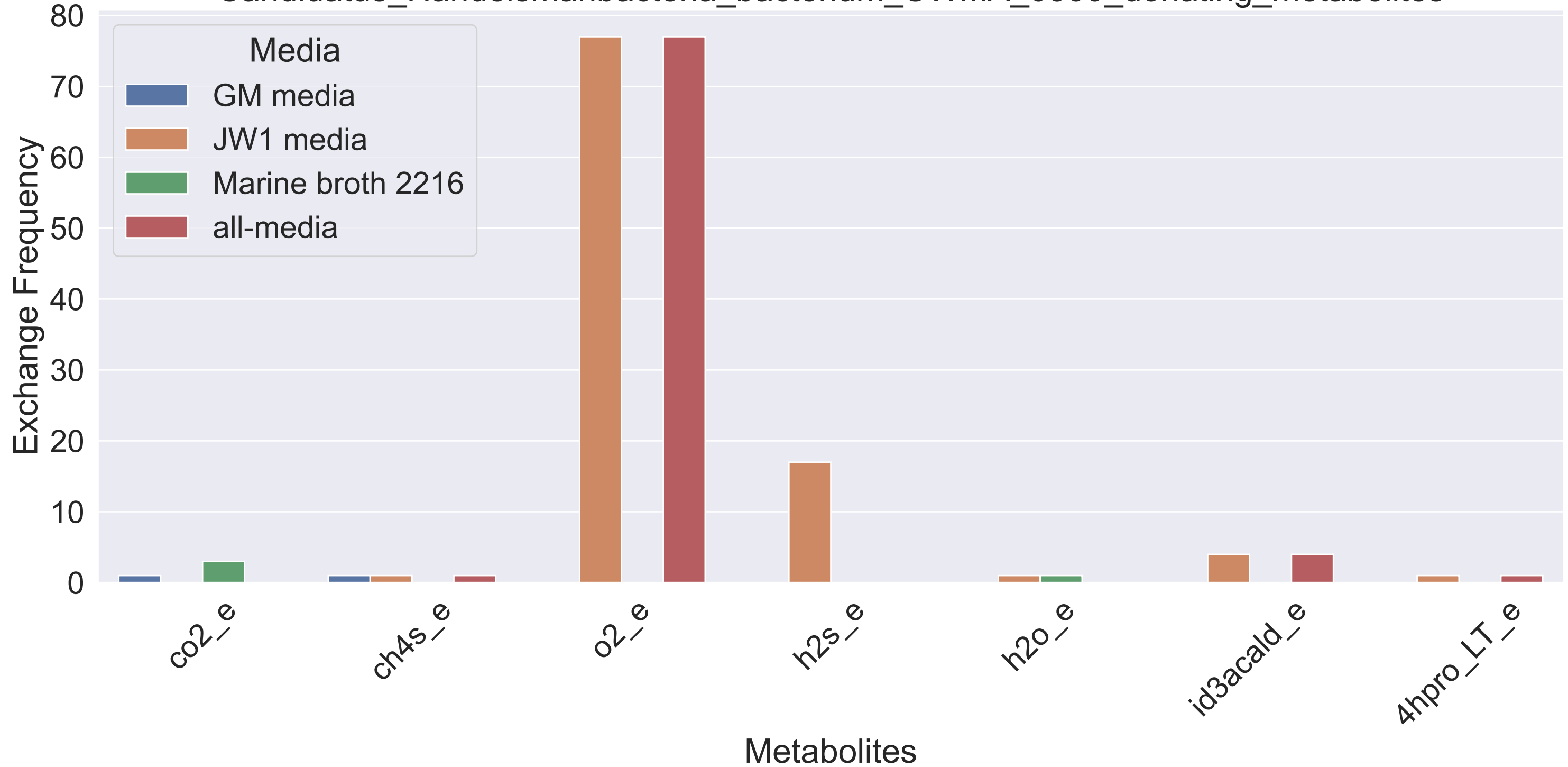

Supplement: Supplementary file 7 — Supplementary File S6 [file 43705_2023_242_MOESM7_ESM.zip › Supplementary Figure S109 Candidatus Handelsmanbacteria bacterium UWMA 0300 donating metabolites.pdf]

# Candidatus\_Lambdaproteobacteria\_bacterium\_UWMA\_0298\_donating\_metabolites

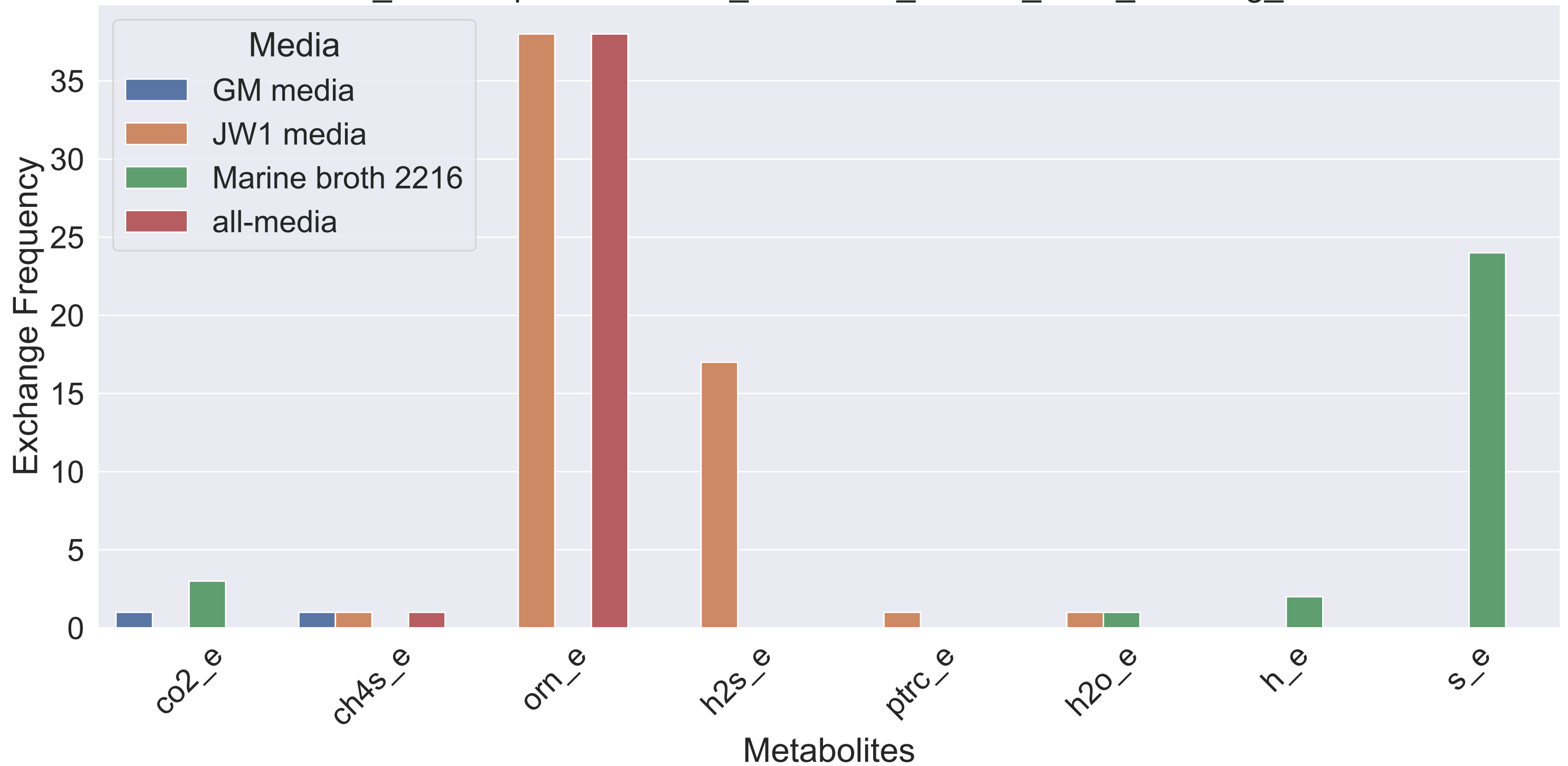

Supplement: Supplementary file 7 — Supplementary File S6 [file 43705_2023_242_MOESM7_ESM.zip › Supplementary Figure S110 Candidatus Lambdaproteobacteria bacterium UWMA 0298 donating metabolites.pdf]

# Candidatus\_Lambdaproteobacteria\_bacterium\_UWMA\_0318\_donating\_metabolites

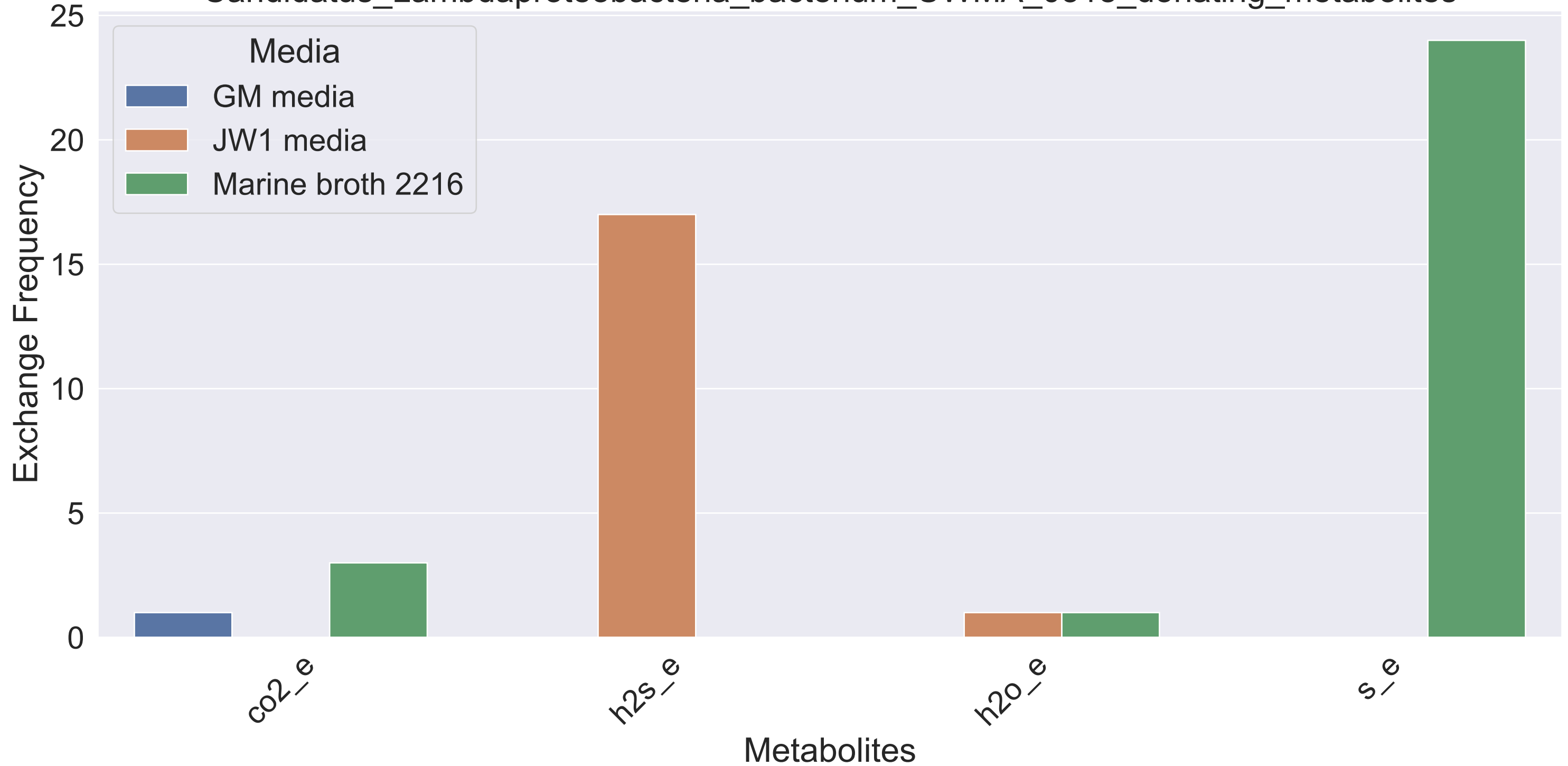

Supplement: Supplementary file 7 — Supplementary File S6 [file 43705_2023_242_MOESM7_ESM.zip › Supplementary Figure S111 Candidatus Lambdaproteobacteria bacterium UWMA 0318 donating metabolites.pdf]

Candidatus\_Marinimicrobia\_bacterium\_UWMA\_0285\_donating\_metabolites

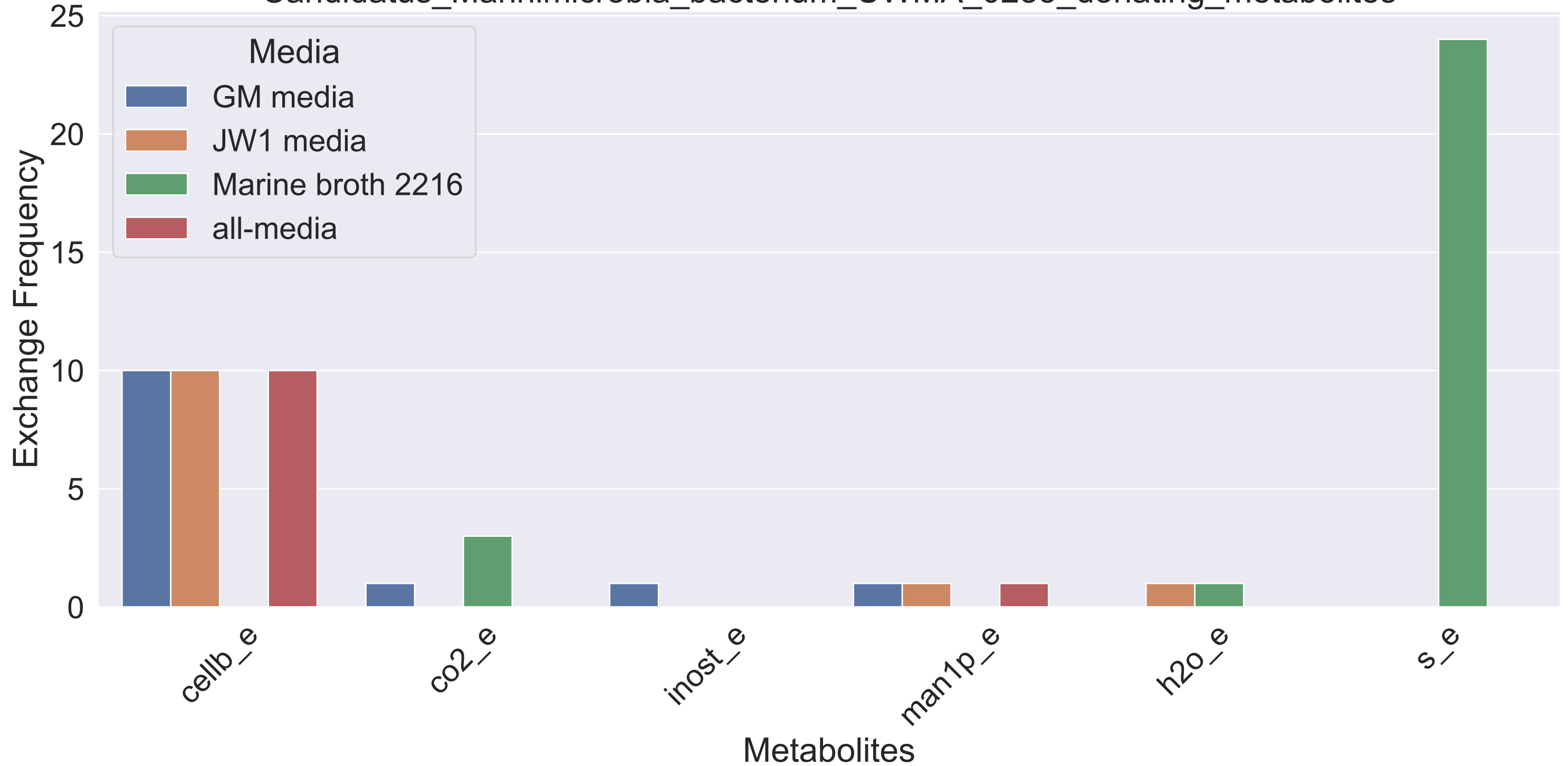

Supplement: Supplementary file 7 — Supplementary File S6 [file 43705_2023_242_MOESM7_ESM.zip › Supplementary Figure S112 Candidatus Marinimicrobia bacterium UWMA 0285 donating metabolites.pdf]

# Candidatus\_Marinimicrobia\_bacterium\_UWMA\_0306\_donating\_metabolites

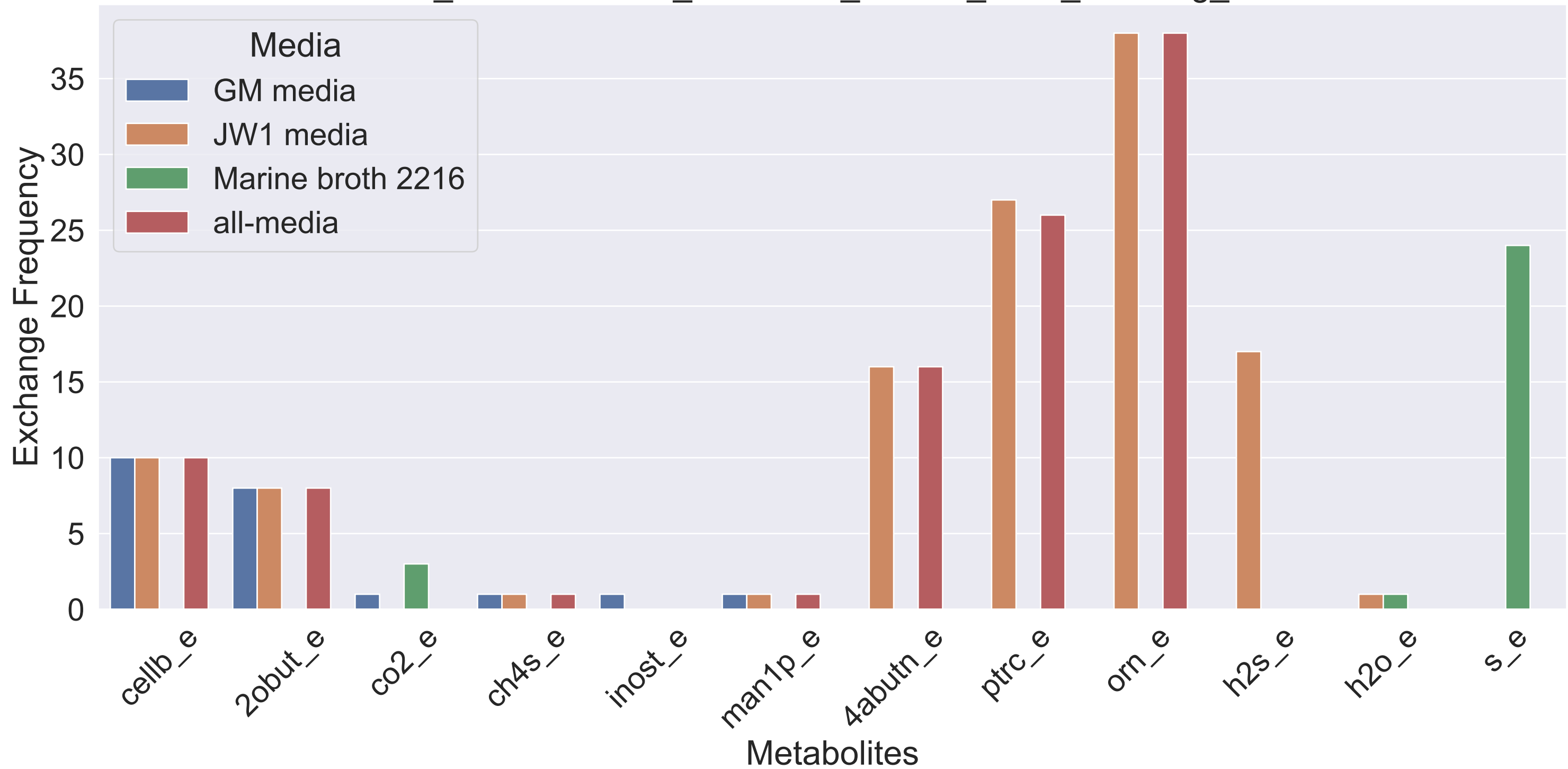

Supplement: Supplementary file 7 — Supplementary File S6 [file 43705_2023_242_MOESM7_ESM.zip › Supplementary Figure S113 Candidatus Marinimicrobia bacterium UWMA 0306 donating metabolites.pdf]

# Candidatus\_Marinimicrobia\_bacterium\_UWMA\_0309\_donating\_metabolites

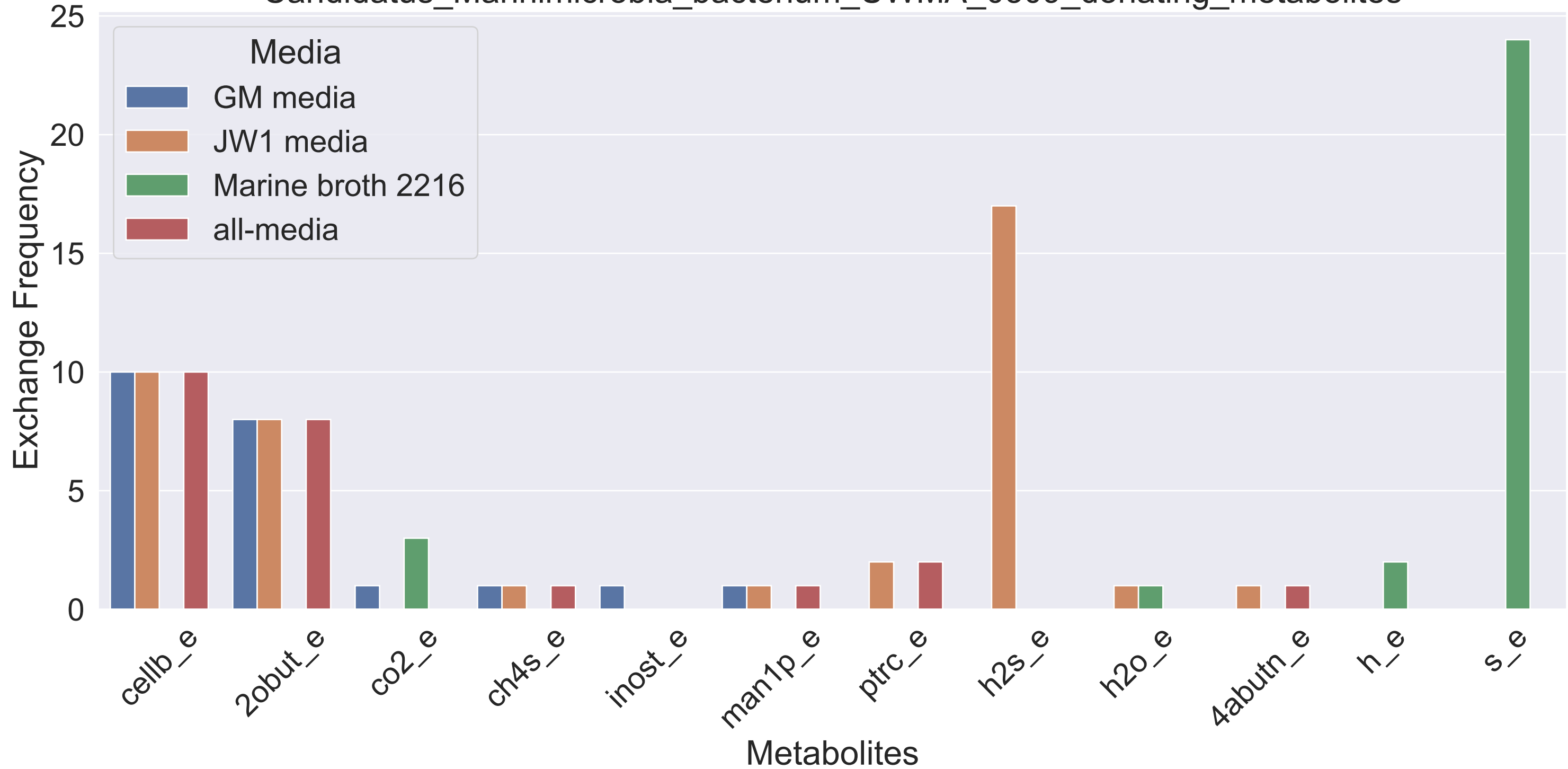

Supplement: Supplementary file 7 — Supplementary File S6 [file 43705_2023_242_MOESM7_ESM.zip › Supplementary Figure S114 Candidatus Marinimicrobia bacterium UWMA 0309 donating metabolites.pdf]

Candidatus\_Marinimicrobia\_bacterium\_UWMA\_0347\_donating\_metabolites

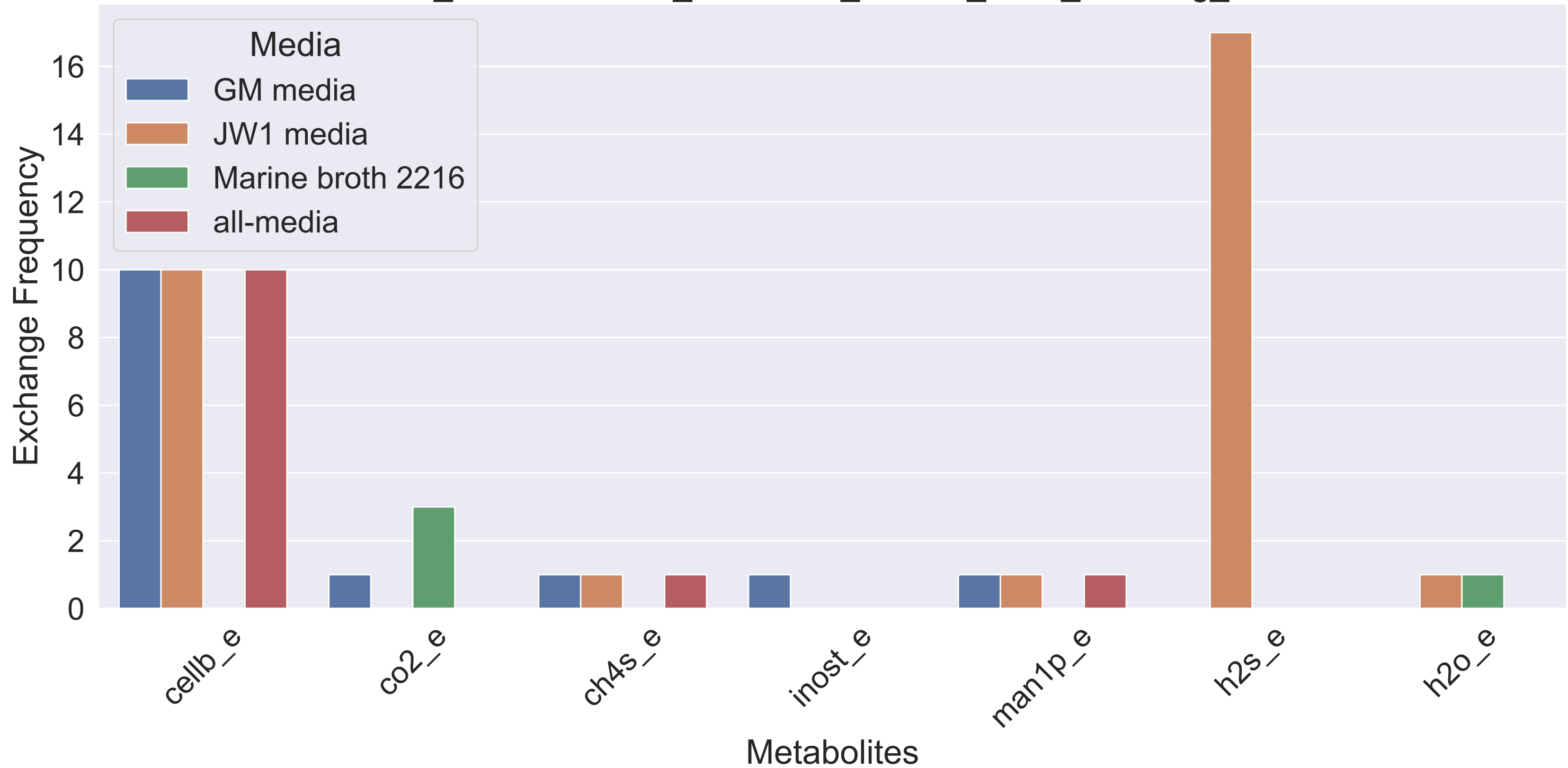

Supplement: Supplementary file 7 — Supplementary File S6 [file 43705_2023_242_MOESM7_ESM.zip › Supplementary Figure S115 Candidatus Marinimicrobia bacterium UWMA 0347 donating metabolites.pdf]

# Candidatus\_Nitrosopelagicus\_sp\_UWMA\_0359\_donating\_metabolites

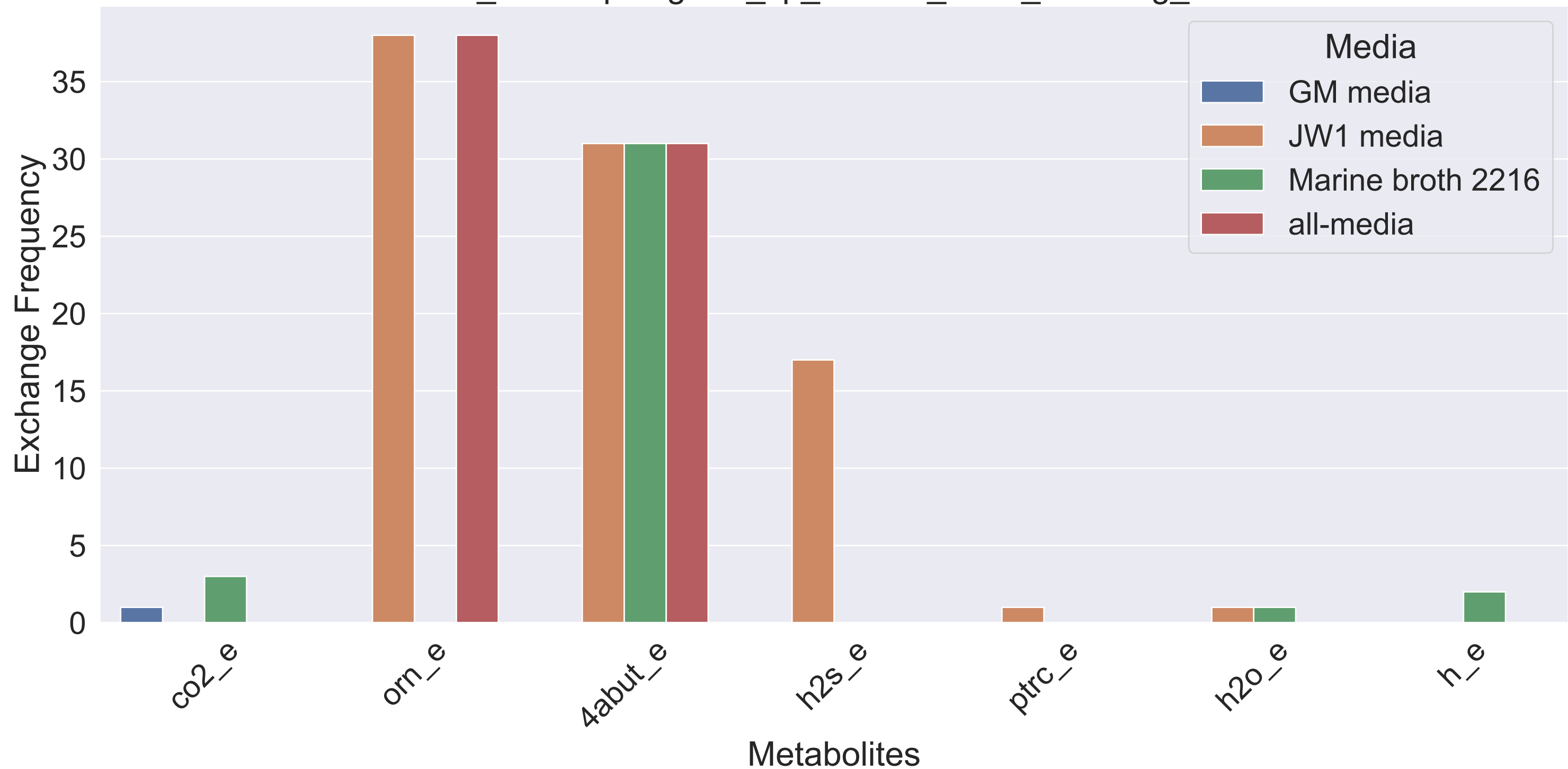

Supplement: Supplementary file 7 — Supplementary File S6 [file 43705_2023_242_MOESM7_ESM.zip › Supplementary Figure S116 Candidatus Nitrosopelagicus sp UWMA 0359 donating metabolites.pdf]

# Candidatus\_Pacearchaeota\_archaeon\_UWMA\_0287\_donating\_metabolites

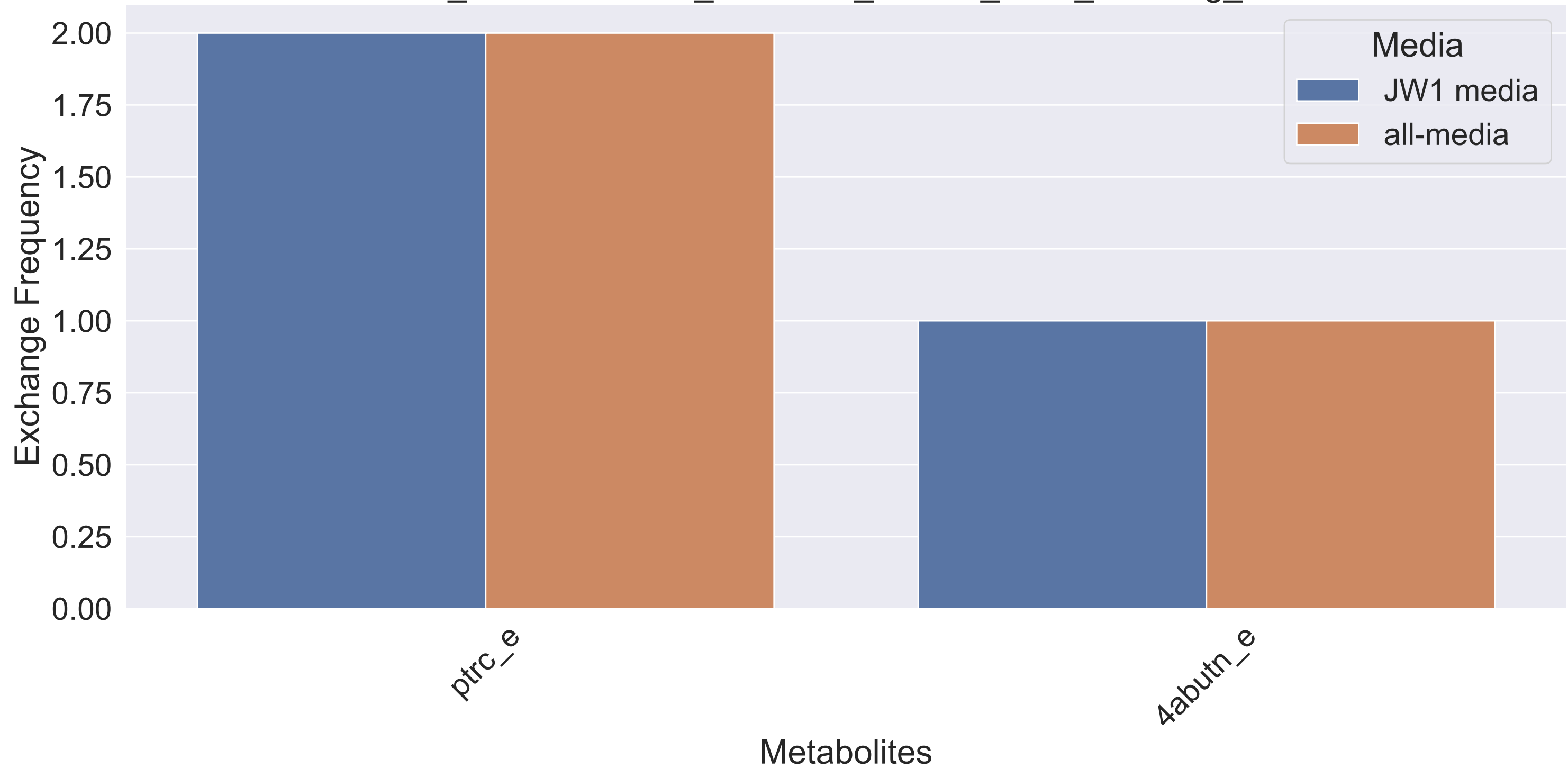

Supplement: Supplementary file 7 — Supplementary File S6 [file 43705_2023_242_MOESM7_ESM.zip › Supplementary Figure S117 Candidatus Pacearchaeota archaeon UWMA 0287 donating metabolites.pdf]

Candidatus\_Pelagibacter\_sp\_UWMA\_0307\_donating\_metabolites

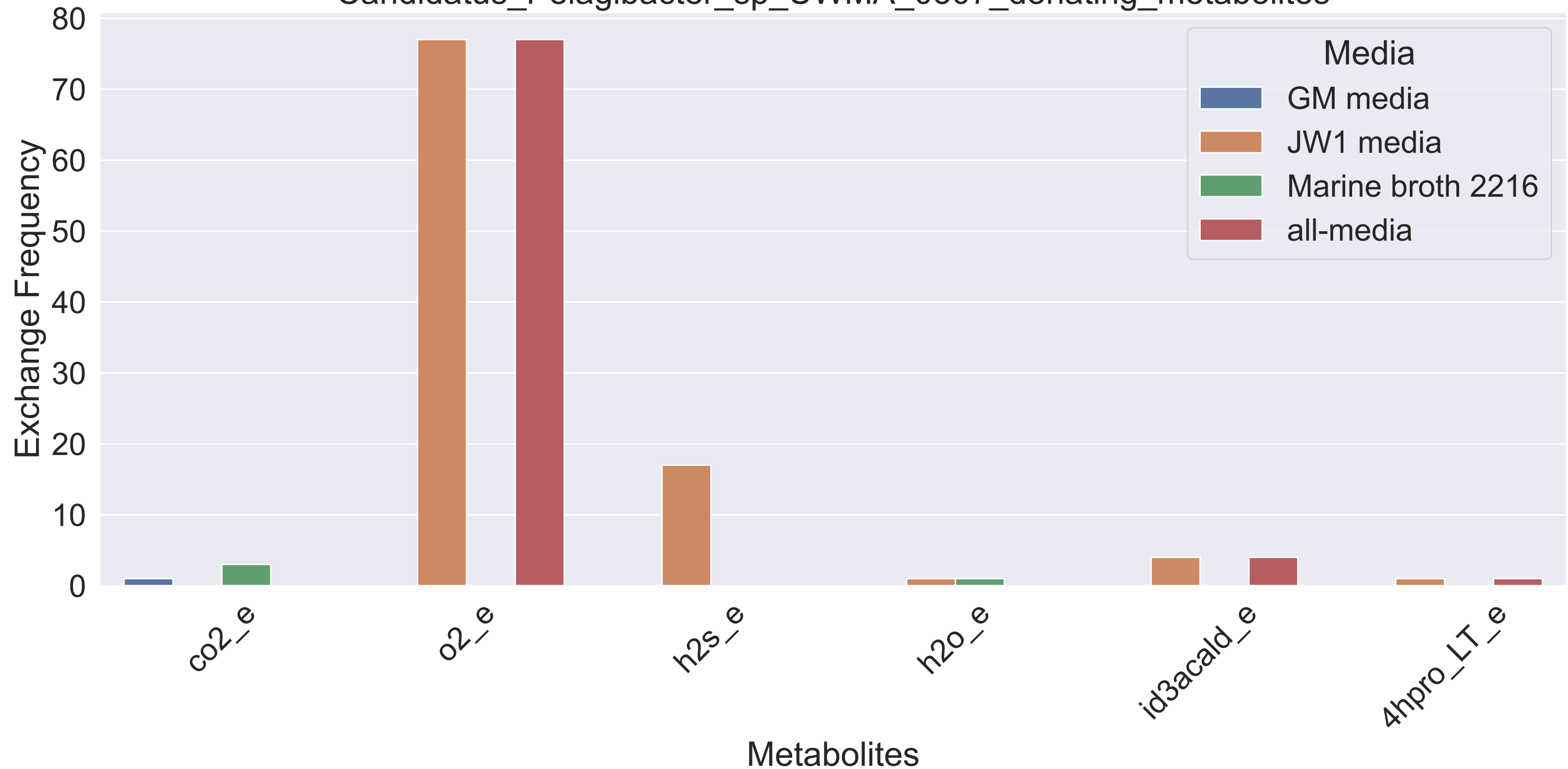

Supplement: Supplementary file 7 — Supplementary File S6 [file 43705_2023_242_MOESM7_ESM.zip › Supplementary Figure S118 Candidatus Pelagibacter sp UWMA 0307 donating metabolites.pdf]

Candidatus\_Thioglobus\_sp\_UWMA\_0259\_donating\_metabolites

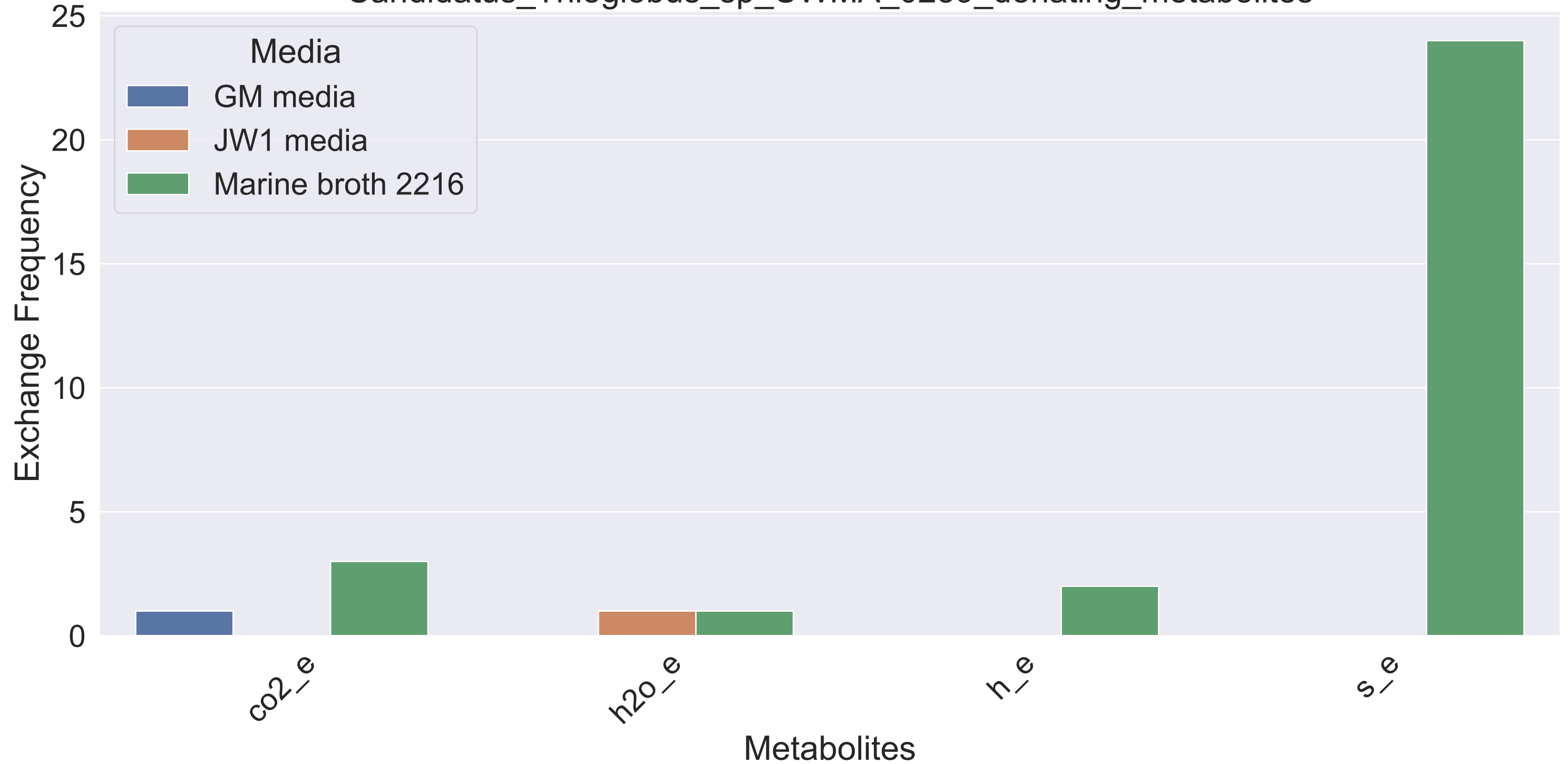

Supplement: Supplementary file 7 — Supplementary File S6 [file 43705_2023_242_MOESM7_ESM.zip › Supplementary Figure S119 Candidatus Thioglobus sp UWMA 0259 donating metabolites.pdf]

Candidatus\_Thioglobus\_sp\_UWMA\_0272\_donating\_metabolites

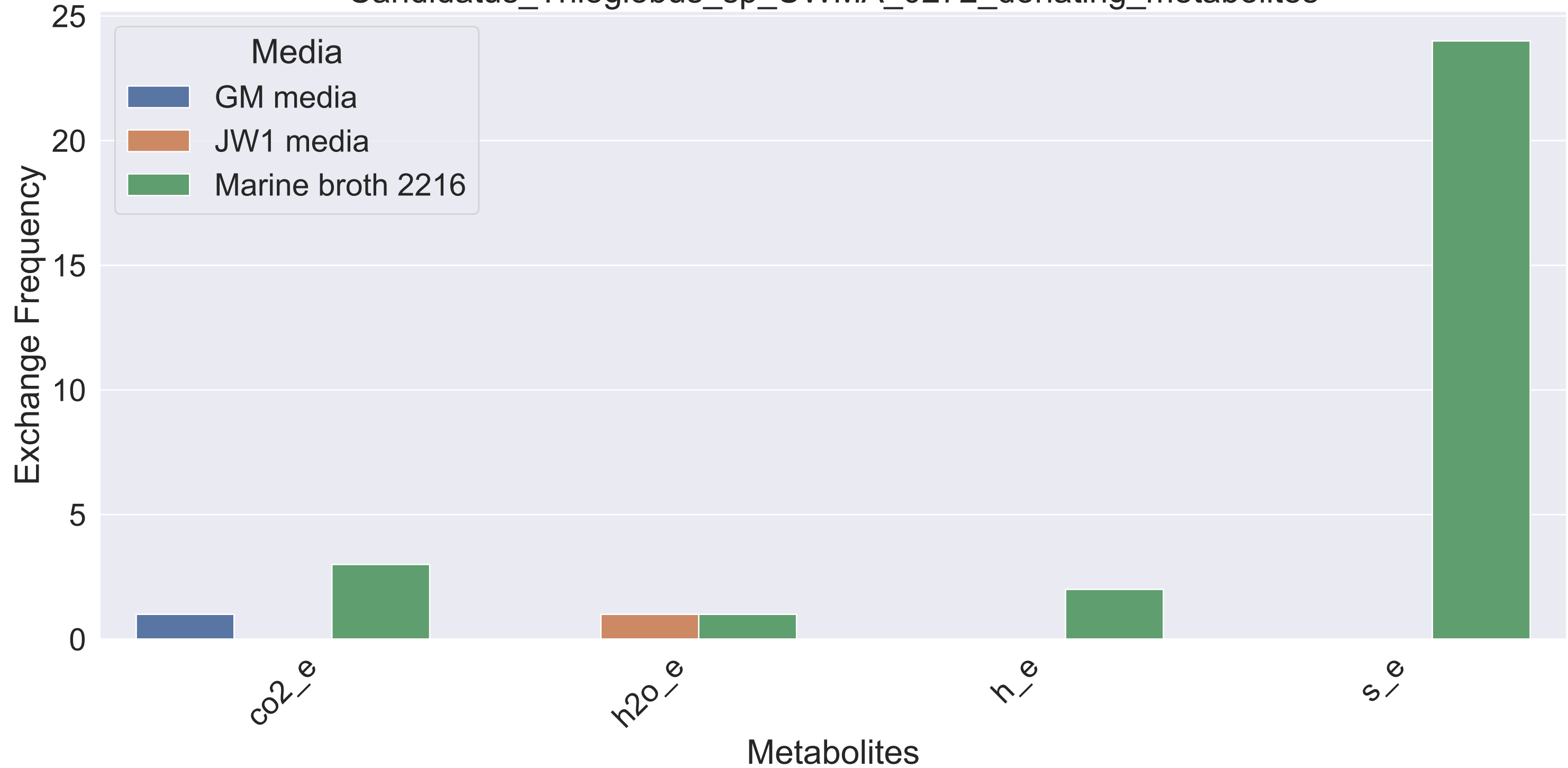

Supplement: Supplementary file 7 — Supplementary File S6 [file 43705_2023_242_MOESM7_ESM.zip › Supplementary Figure S120 Candidatus Thioglobus sp UWMA 0272 donating metabolites.pdf]

Candidatus\_Thioglobus\_sp\_UWMA\_0322\_donating\_metabolites

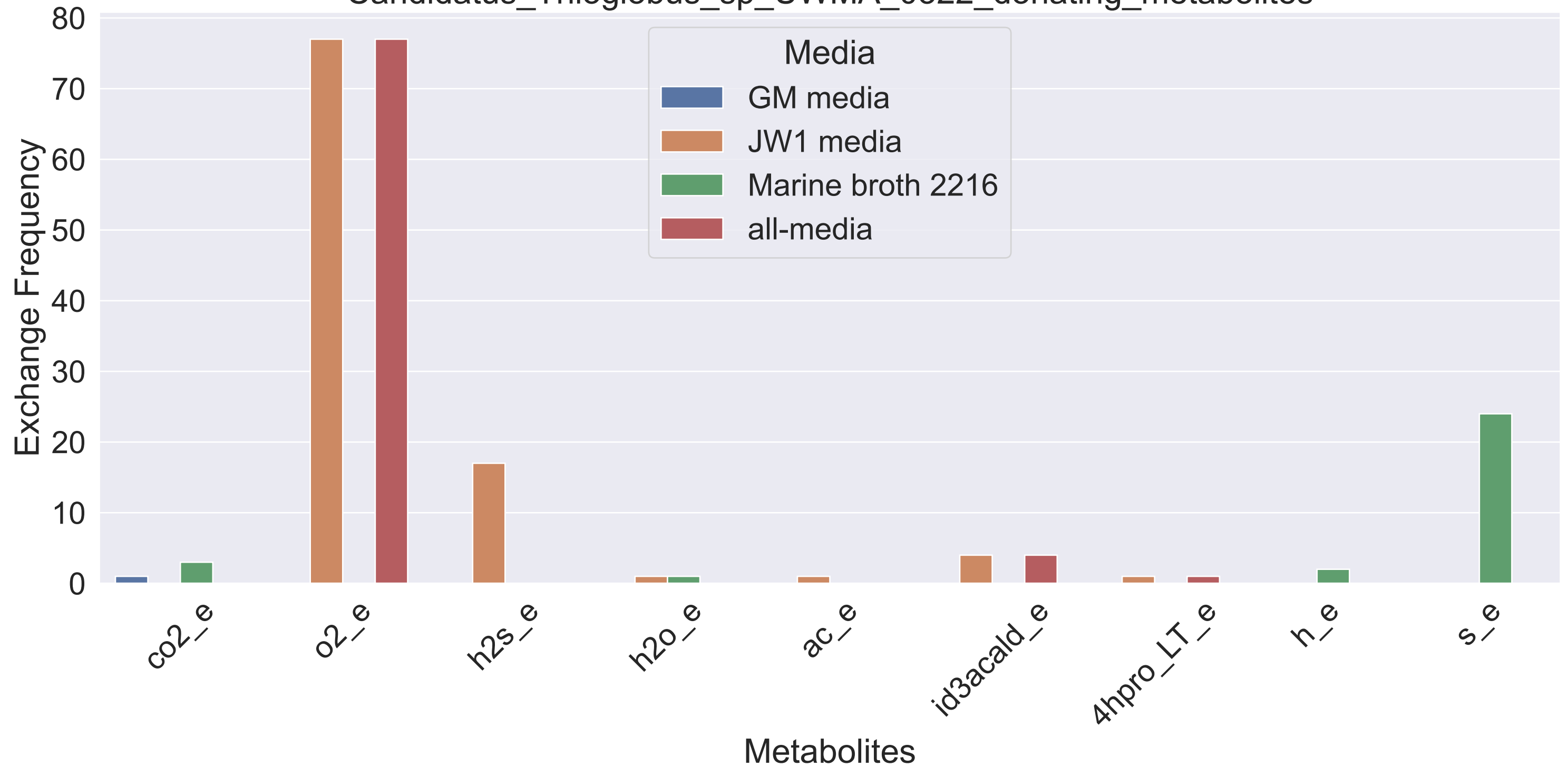

Supplement: Supplementary file 7 — Supplementary File S6 [file 43705_2023_242_MOESM7_ESM.zip › Supplementary Figure S121 Candidatus Thioglobus sp UWMA 0322 donating metabolites.pdf]

Candidatus\_Thioglobus\_sp\_UWMA\_0342\_donating\_metabolites

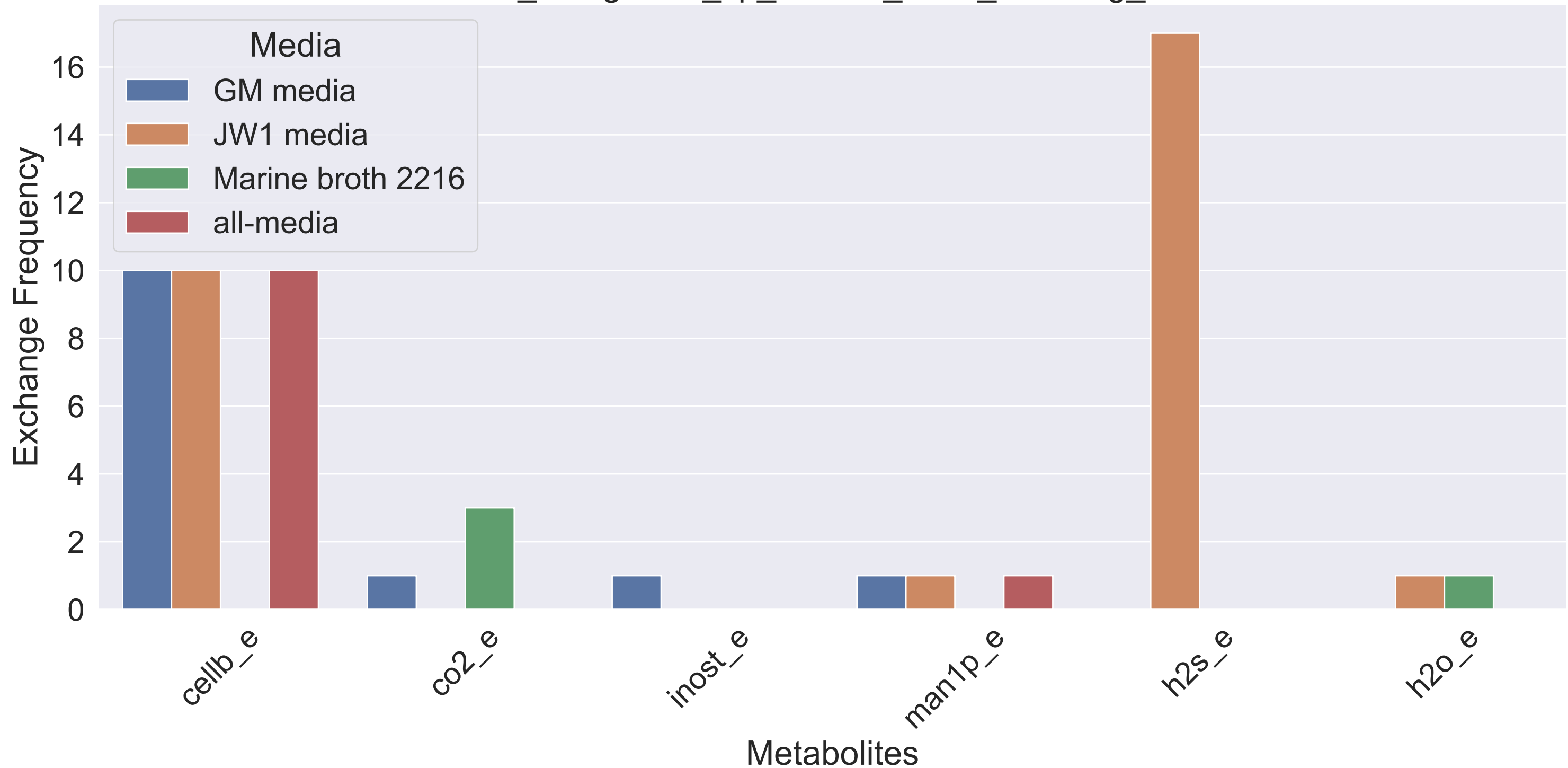

Supplement: Supplementary file 7 — Supplementary File S6 [file 43705_2023_242_MOESM7_ESM.zip › Supplementary Figure S122 Candidatus Thioglobus sp UWMA 0342 donating metabolites.pdf]

# Candidatus\_Thioglobus\_sp\_UWMA\_0360\_donating\_metabolites

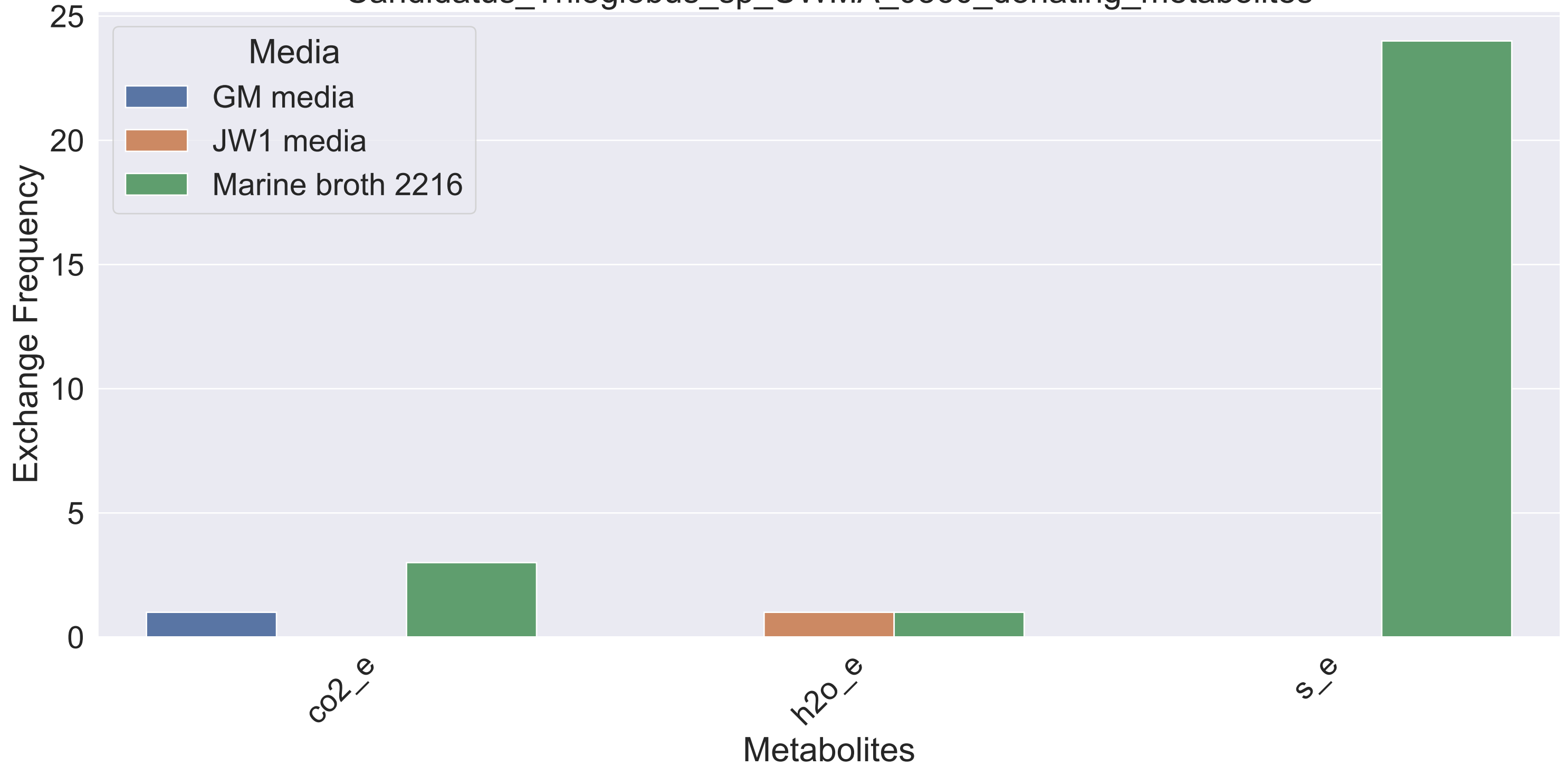

Supplement: Supplementary file 7 — Supplementary File S6 [file 43705_2023_242_MOESM7_ESM.zip › Supplementary Figure S123 Candidatus Thioglobus sp UWMA 0360 donating metabolites.pdf]

# Cycloclasticus\_sp\_UWMA\_0316\_donating\_metabolites

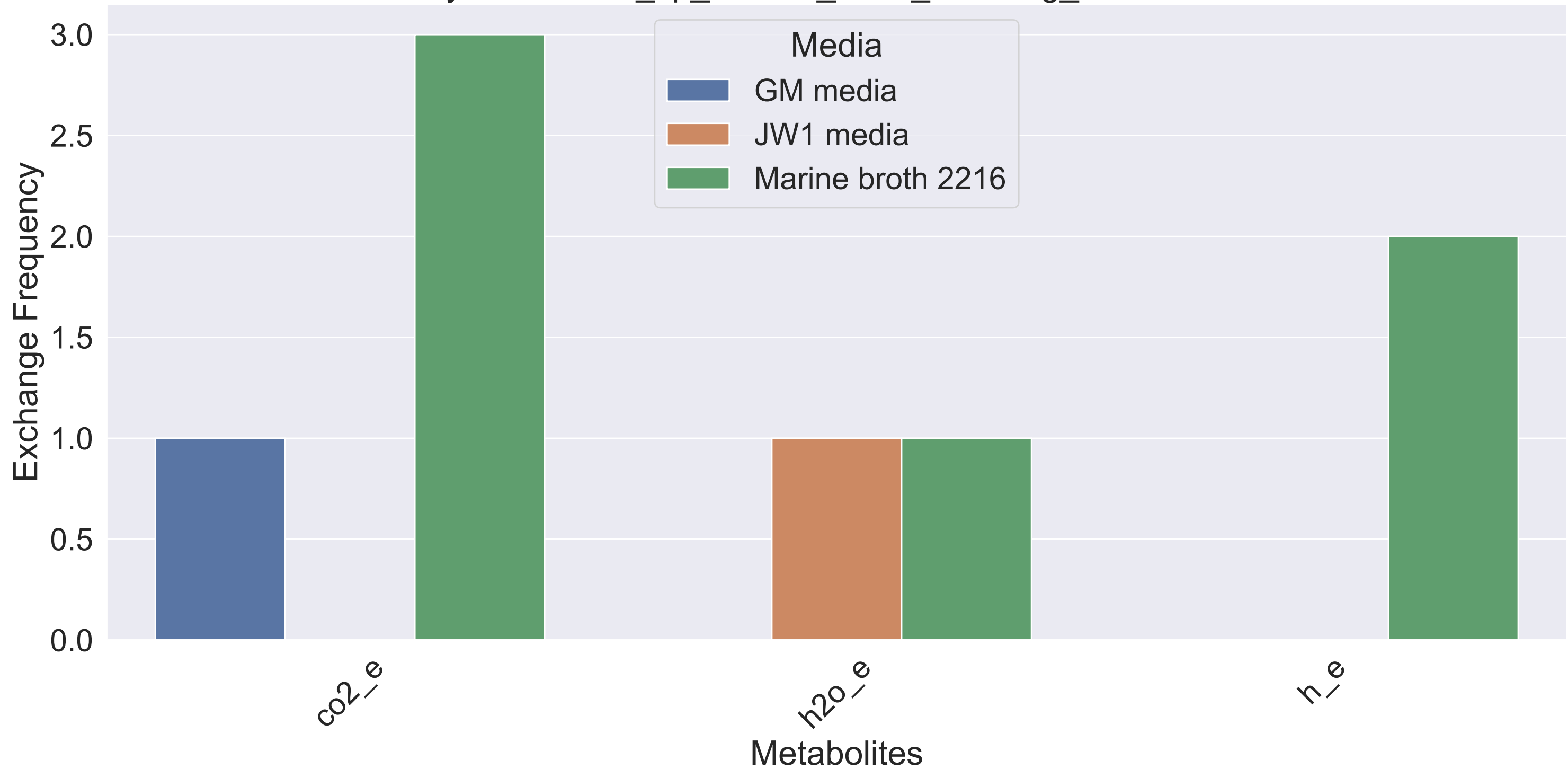

Supplement: Supplementary file 7 — Supplementary File S6 [file 43705_2023_242_MOESM7_ESM.zip › Supplementary Figure S124 Cycloclasticus sp UWMA 0316 donating metabolites.pdf]

# Cycloclasticus\_sp\_UWMA\_0335\_donating\_metabolites

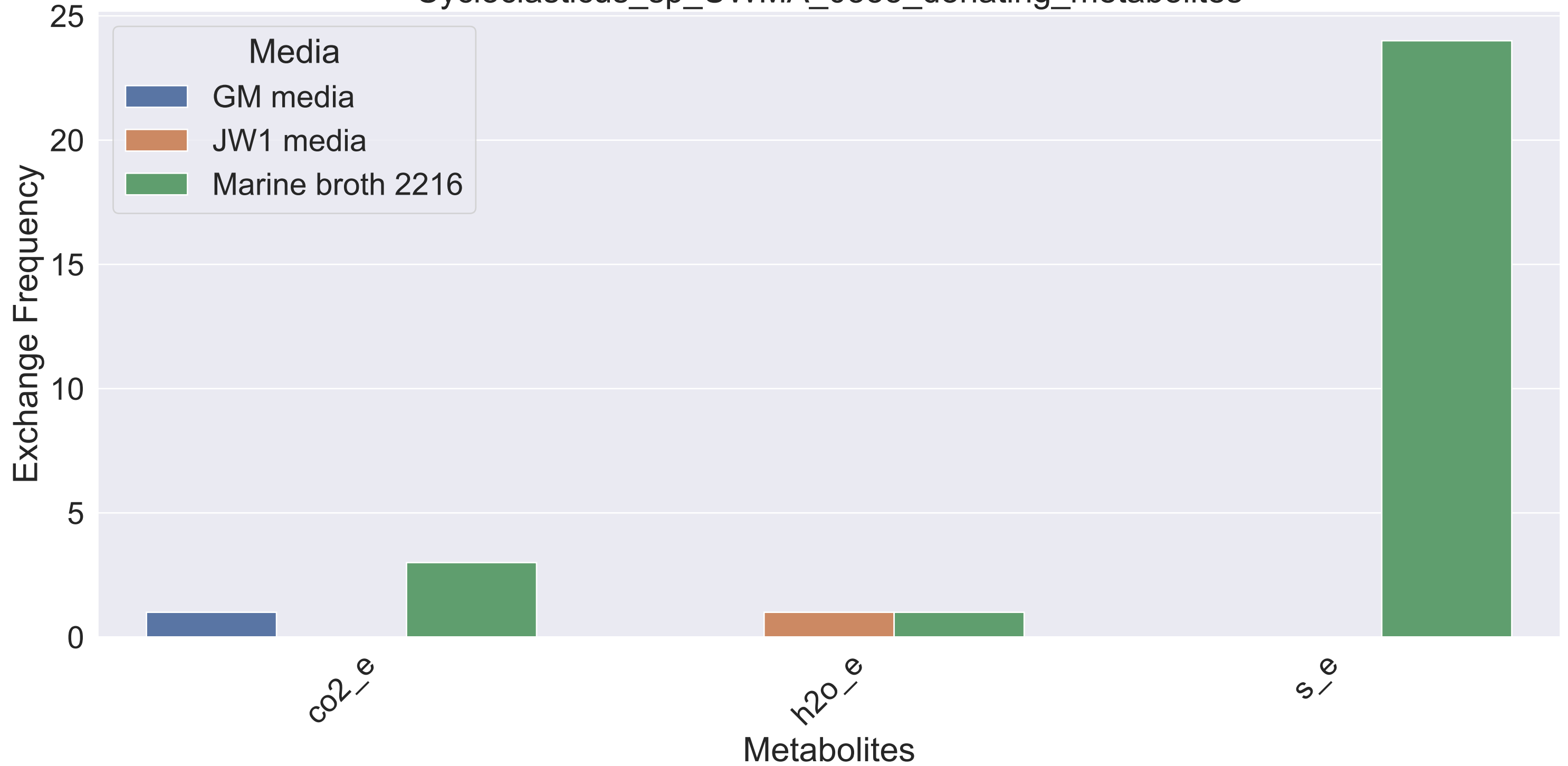

Supplement: Supplementary file 7 — Supplementary File S6 [file 43705_2023_242_MOESM7_ESM.zip › Supplementary Figure S125 Cycloclasticus sp UWMA 0335 donating metabolites.pdf]

# Cytophagales\_bacterium\_UWMA\_0338\_donating\_metabolites

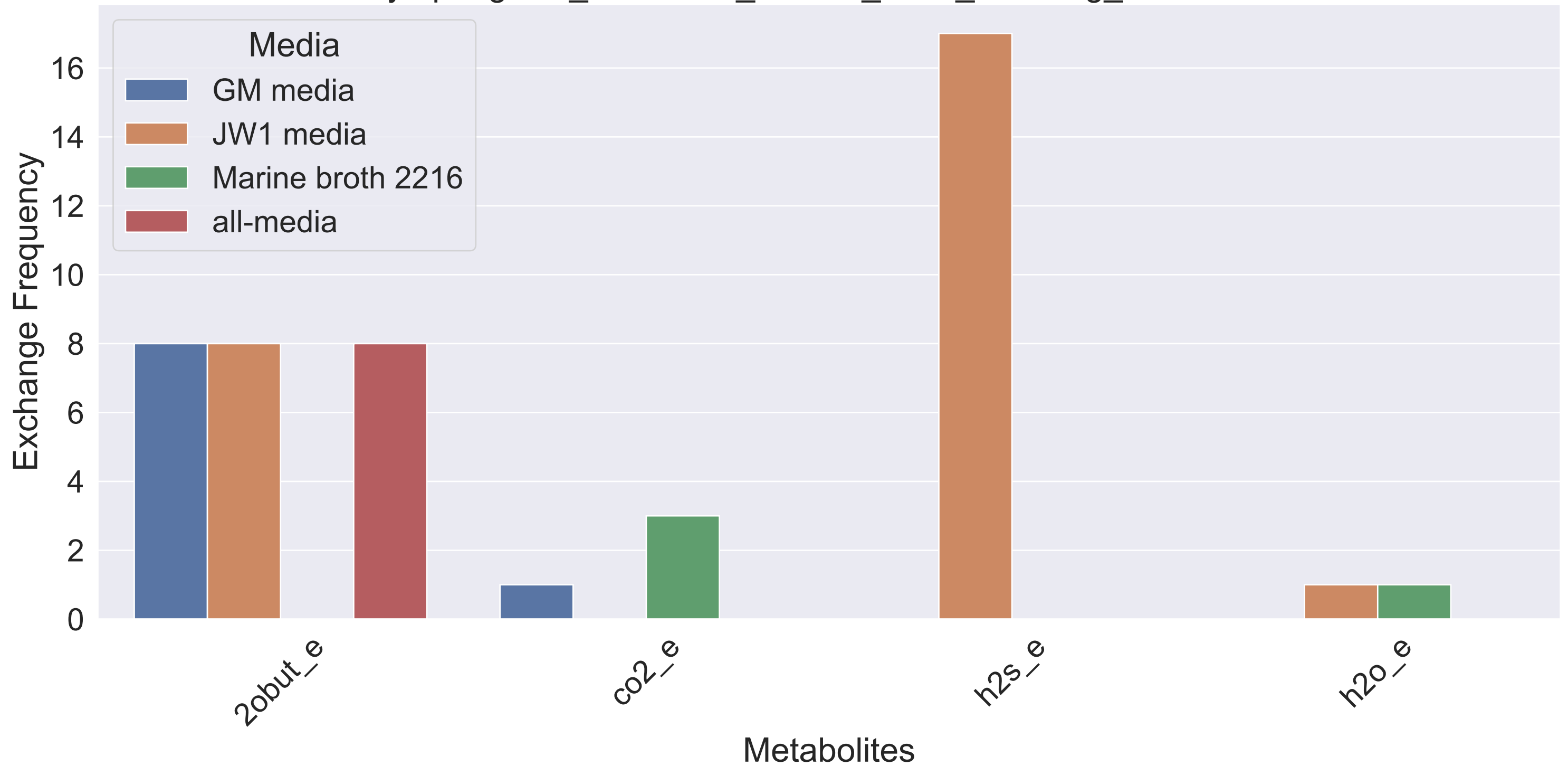

Supplement: Supplementary file 7 — Supplementary File S6 [file 43705_2023_242_MOESM7_ESM.zip › Supplementary Figure S126 Cytophagales bacterium UWMA 0338 donating metabolites.pdf]

Dehalococcoidia\_bacterium\_UWMA\_0267\_donating\_metabolites

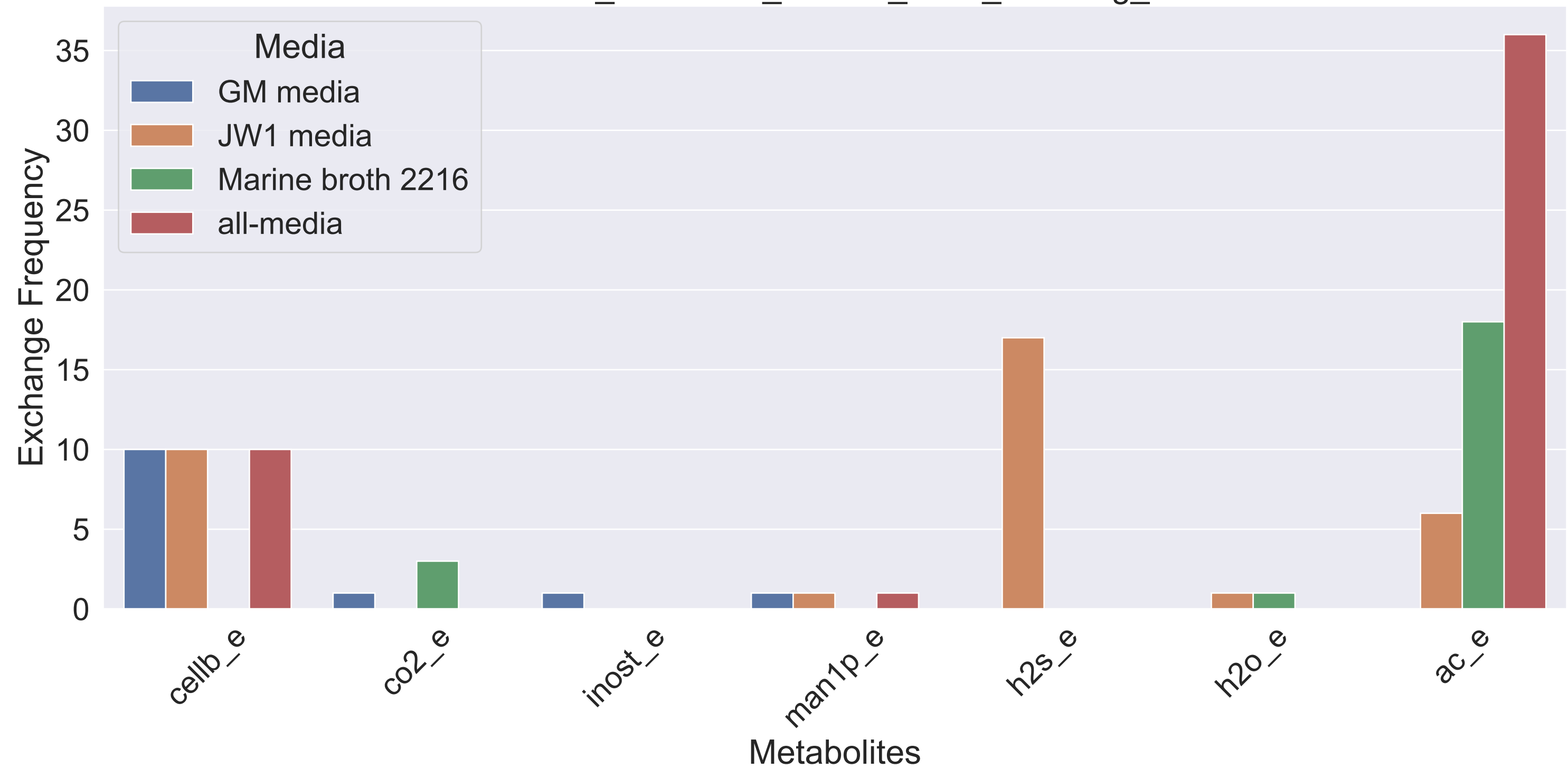

Supplement: Supplementary file 7 — Supplementary File S6 [file 43705_2023_242_MOESM7_ESM.zip › Supplementary Figure S127 Dehalococcoidia bacterium UWMA 0267 donating metabolites.pdf]

Dehalococcoidia\_bacterium\_UWMA\_0331\_donating\_metabolites

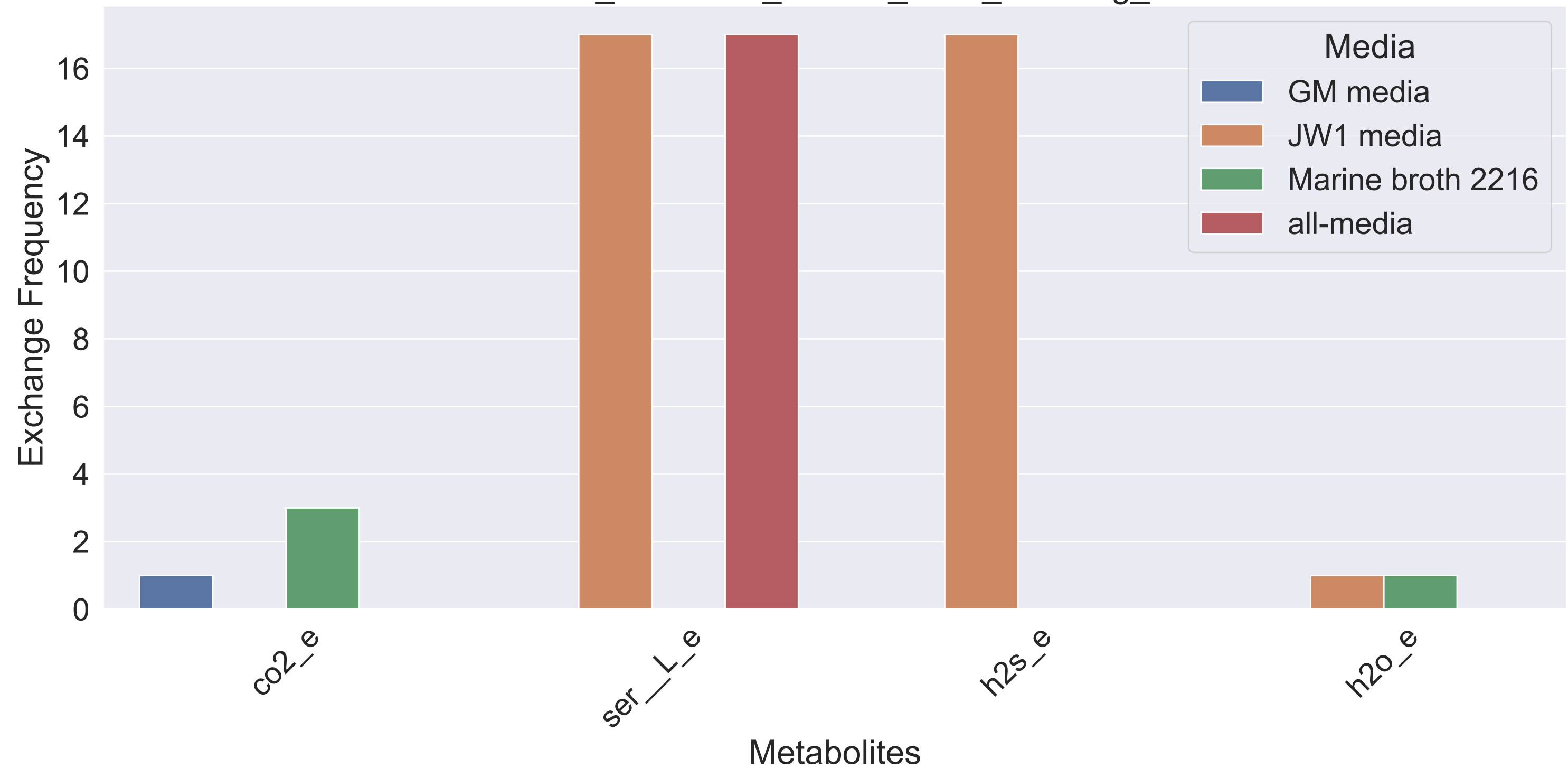

Supplement: Supplementary file 7 — Supplementary File S6 [file 43705_2023_242_MOESM7_ESM.zip › Supplementary Figure S128 Dehalococcoidia bacterium UWMA 0331 donating metabolites.pdf]

Dehalococcoidia\_bacterium\_UWMA\_0337\_donating\_metabolites

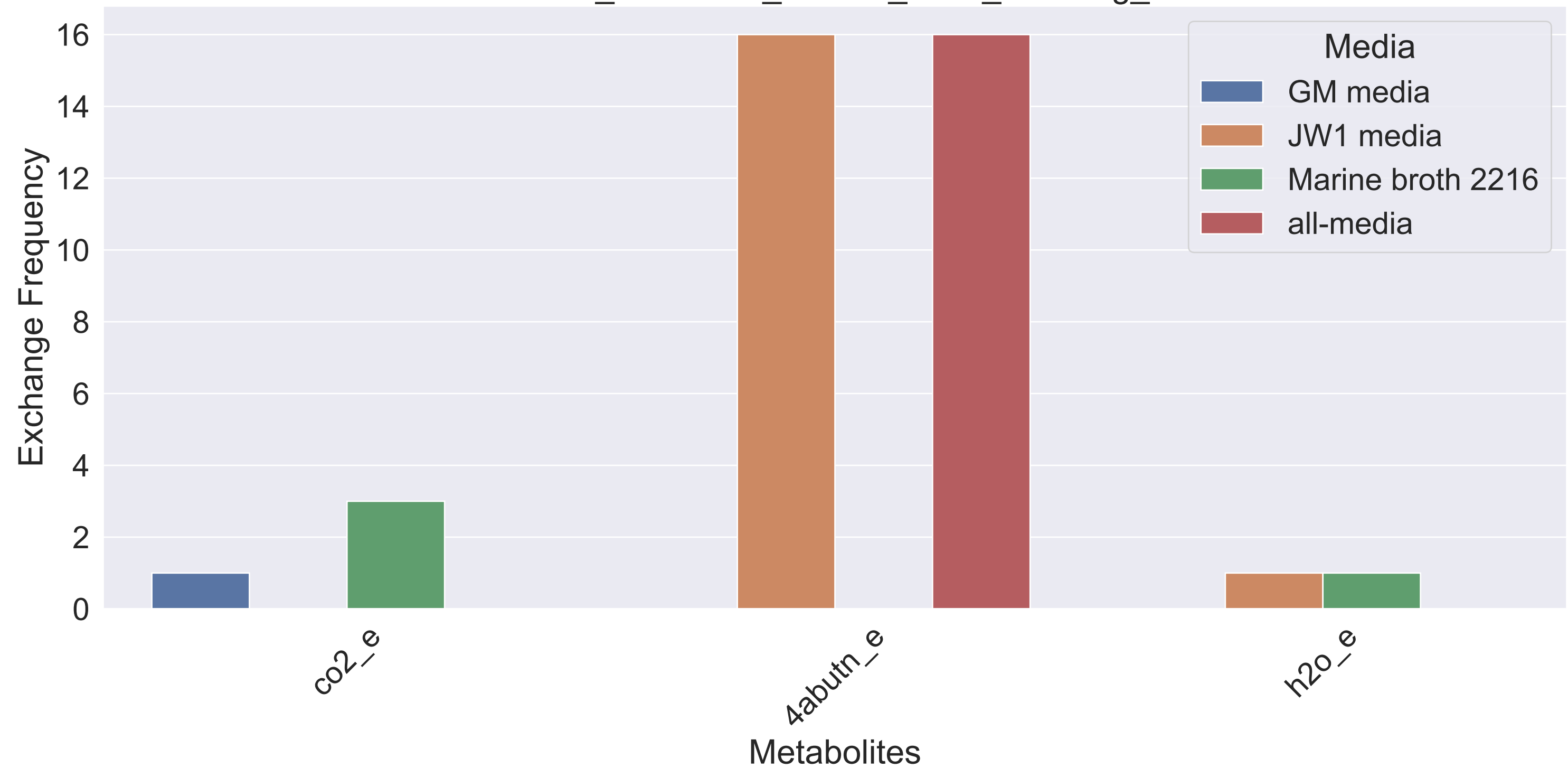

Supplement: Supplementary file 7 — Supplementary File S6 [file 43705_2023_242_MOESM7_ESM.zip › Supplementary Figure S129 Dehalococcoidia bacterium UWMA 0337 donating metabolites.pdf]

Dehalococcoidia\_bacterium\_UWMA\_0354\_donating\_metabolites

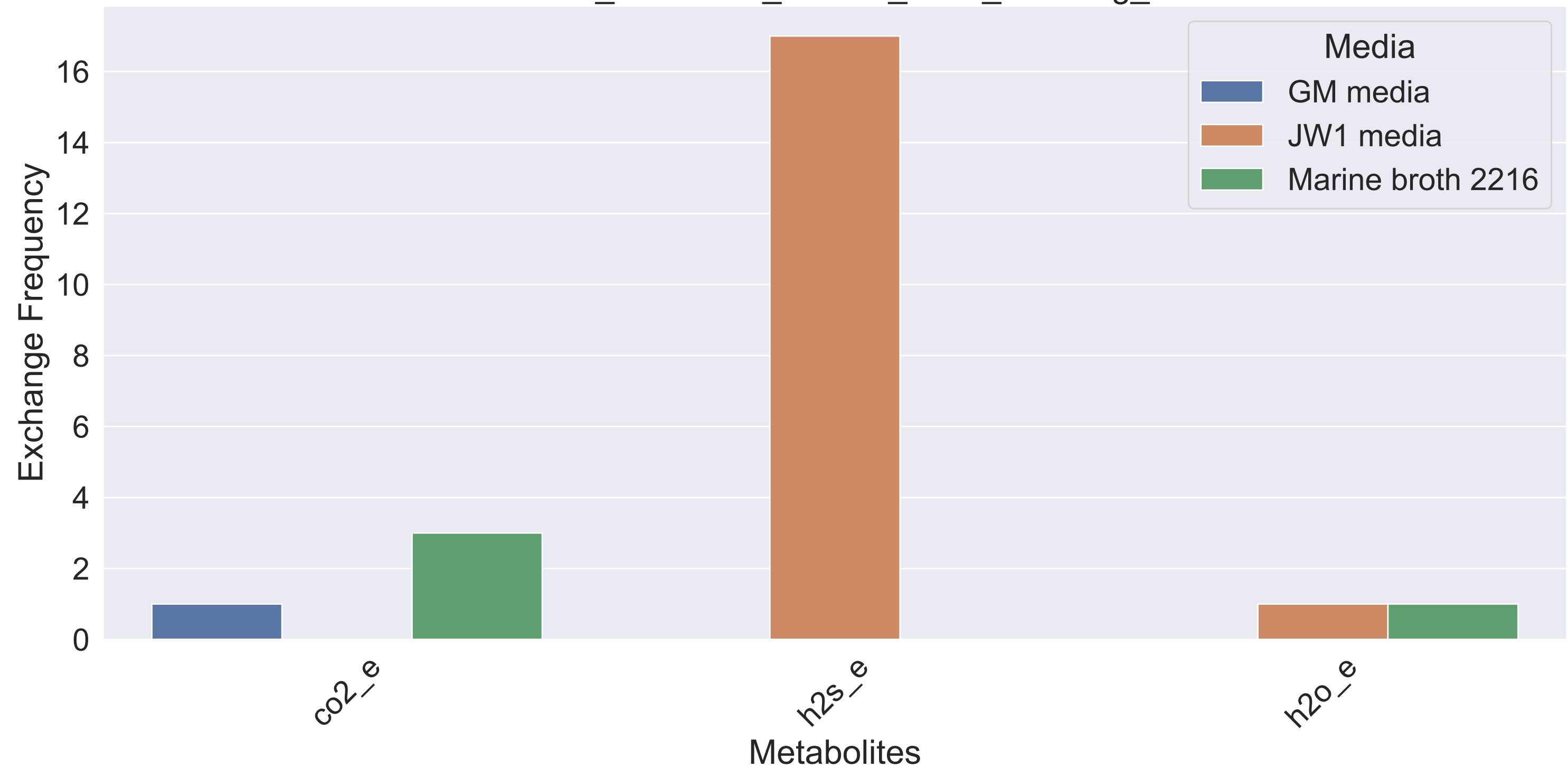

Supplement: Supplementary file 7 — Supplementary File S6 [file 43705_2023_242_MOESM7_ESM.zip › Supplementary Figure S130 Dehalococcoidia bacterium UWMA 0354 donating metabolites.pdf]

# Flavobacteriaceae\_bacterium\_UWMA\_0314\_donating\_metabolites

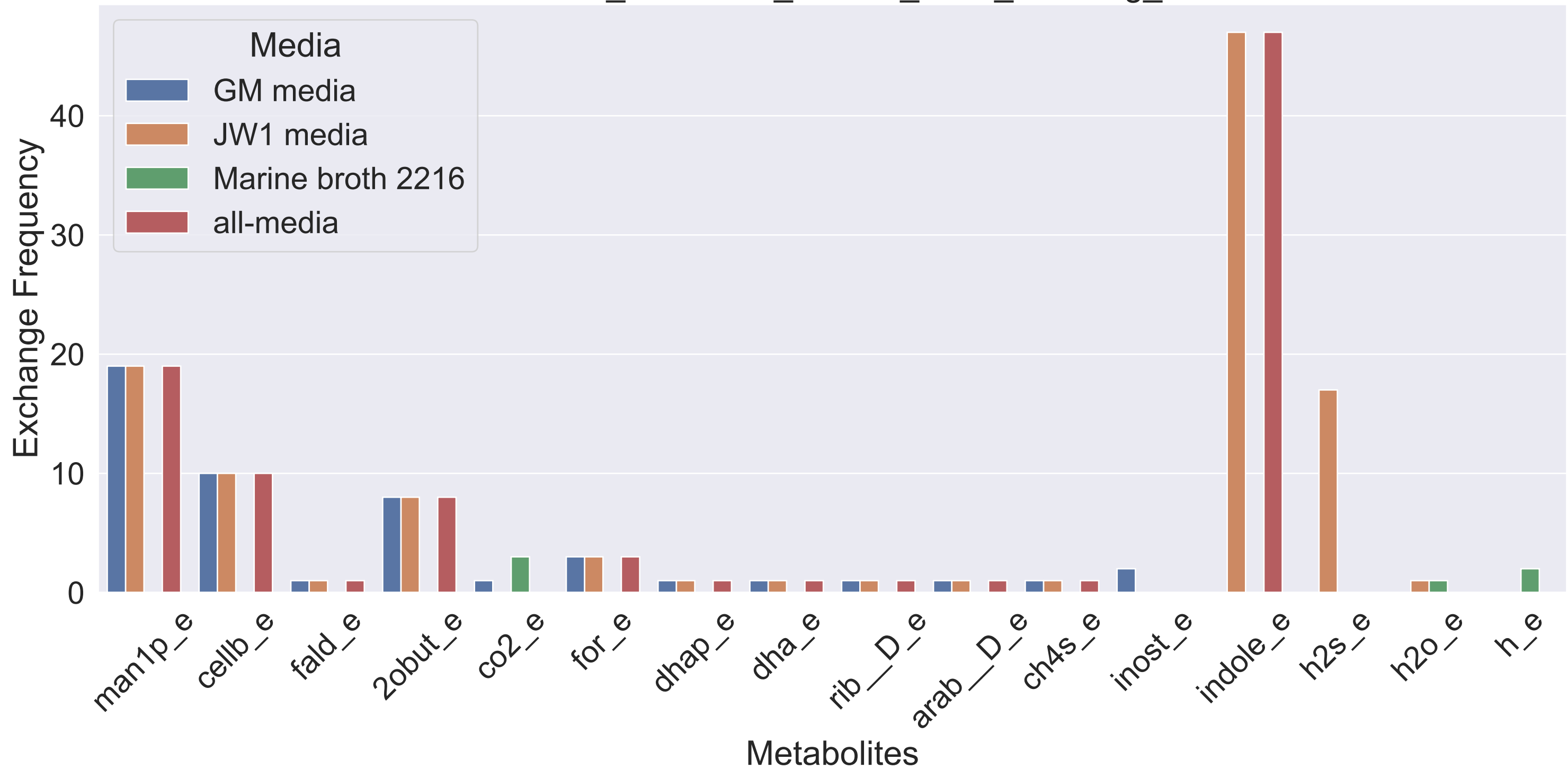

Supplement: Supplementary file 7 — Supplementary File S6 [file 43705_2023_242_MOESM7_ESM.zip › Supplementary Figure S131 Flavobacteriaceae bacterium UWMA 0314 donating metabolites.pdf]

# Flavobacteriaceae\_bacterium\_UWMA\_0351\_donating\_metabolites

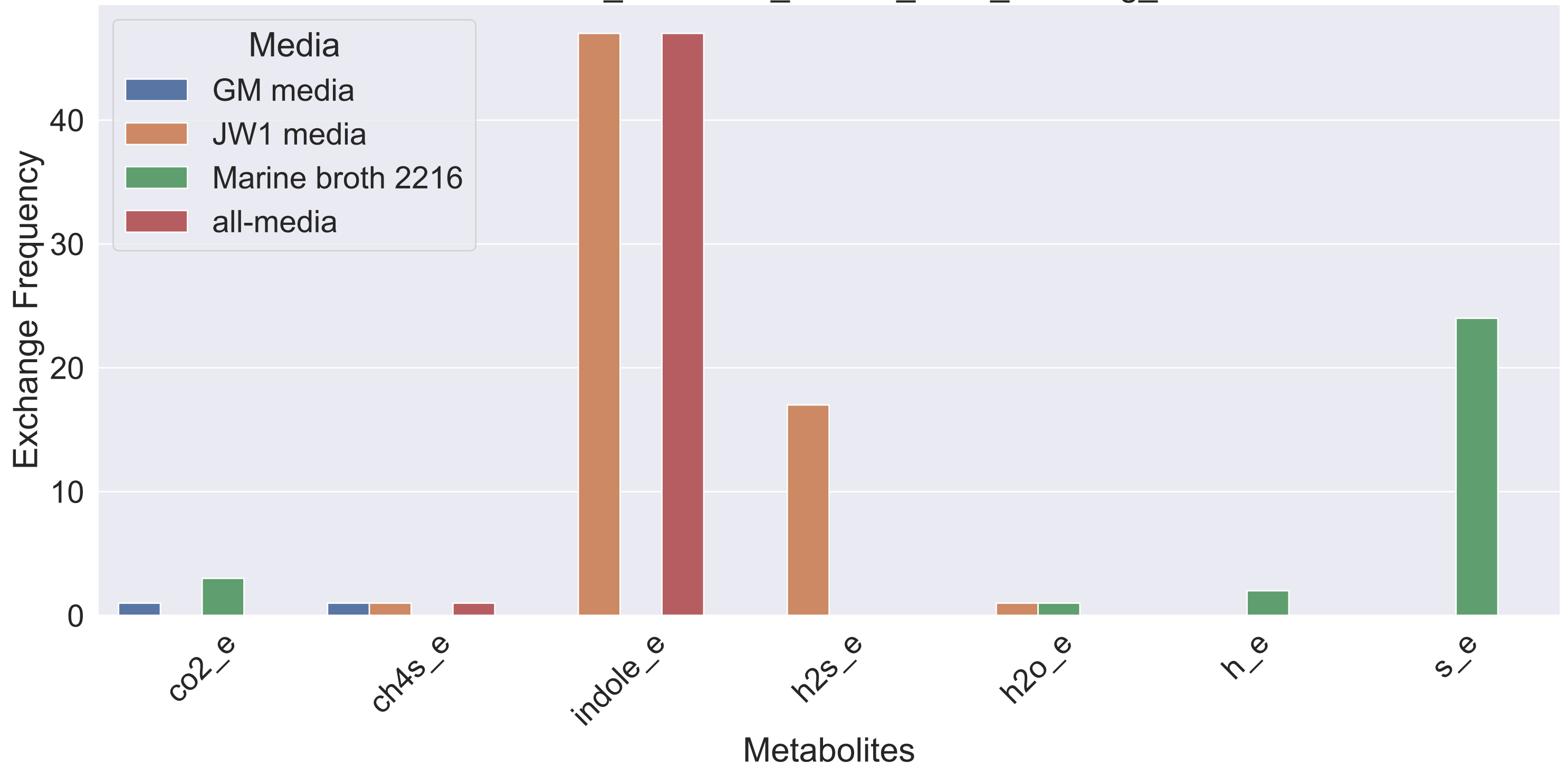

Supplement: Supplementary file 7 — Supplementary File S6 [file 43705_2023_242_MOESM7_ESM.zip › Supplementary Figure S132 Flavobacteriaceae bacterium UWMA 0351 donating metabolites.pdf]

# Flavobacteriales\_bacterium\_UWMA\_0274\_donating\_metabolites

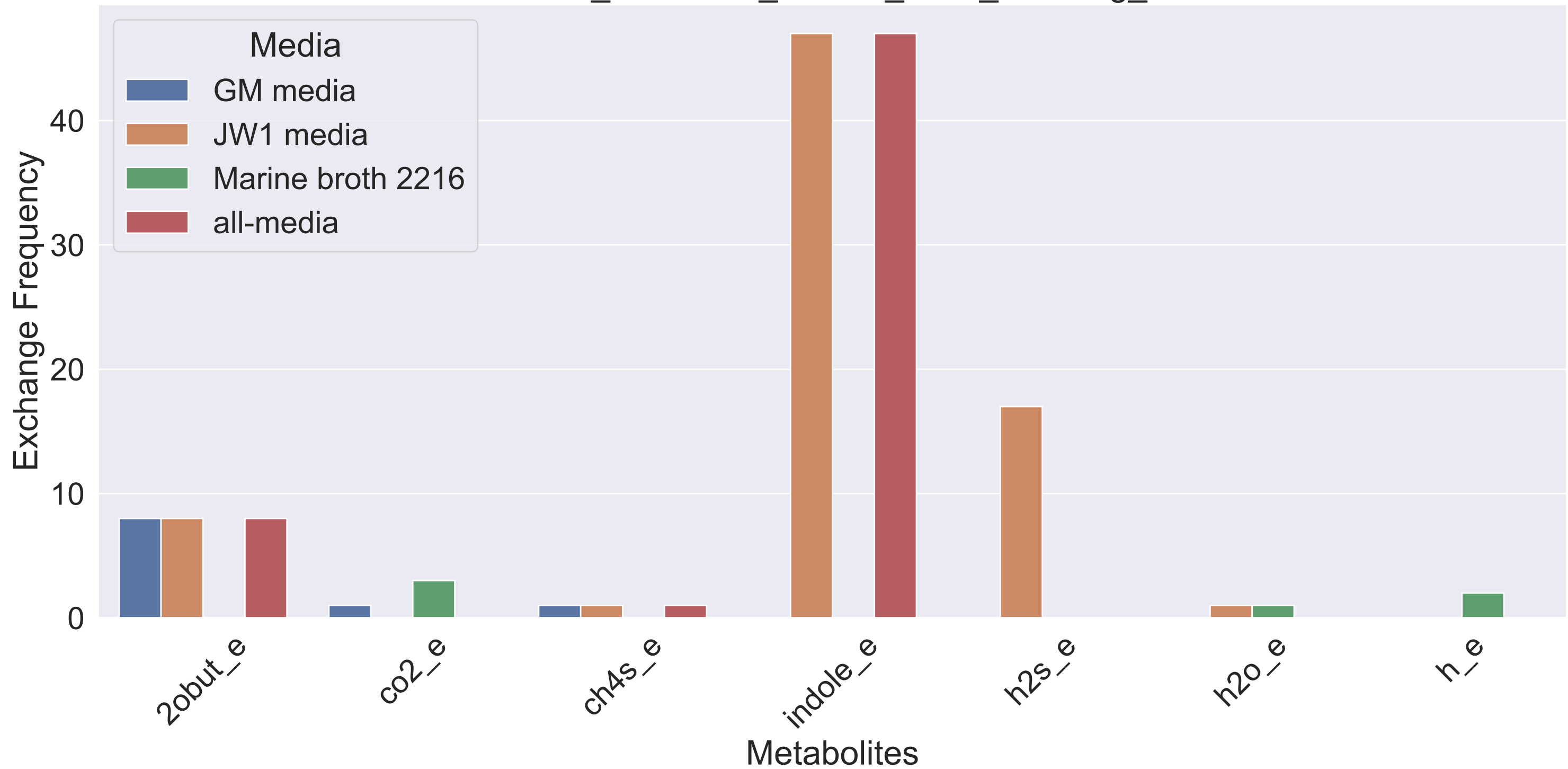

Supplement: Supplementary file 7 — Supplementary File S6 [file 43705_2023_242_MOESM7_ESM.zip › Supplementary Figure S133 Flavobacteriales bacterium UWMA 0274 donating metabolites.pdf]

# Flavobacteriales\_bacterium\_UWMA\_0277\_donating\_metabolites

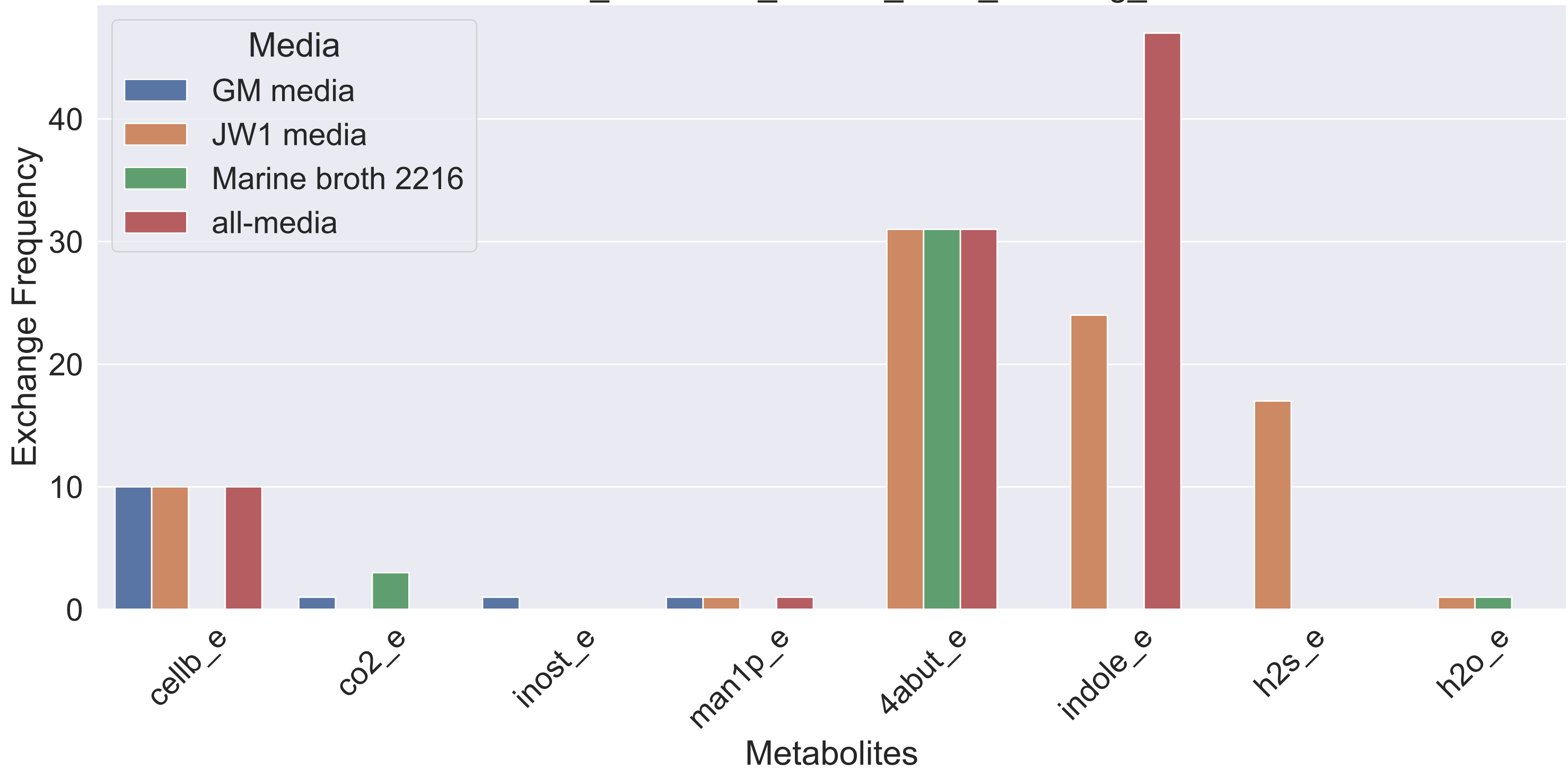

Supplement: Supplementary file 7 — Supplementary File S6 [file 43705_2023_242_MOESM7_ESM.zip › Supplementary Figure S134 Flavobacteriales bacterium UWMA 0277 donating metabolites.pdf]

# Flavobacteriales\_bacterium\_UWMA\_0289\_donating\_metabolites

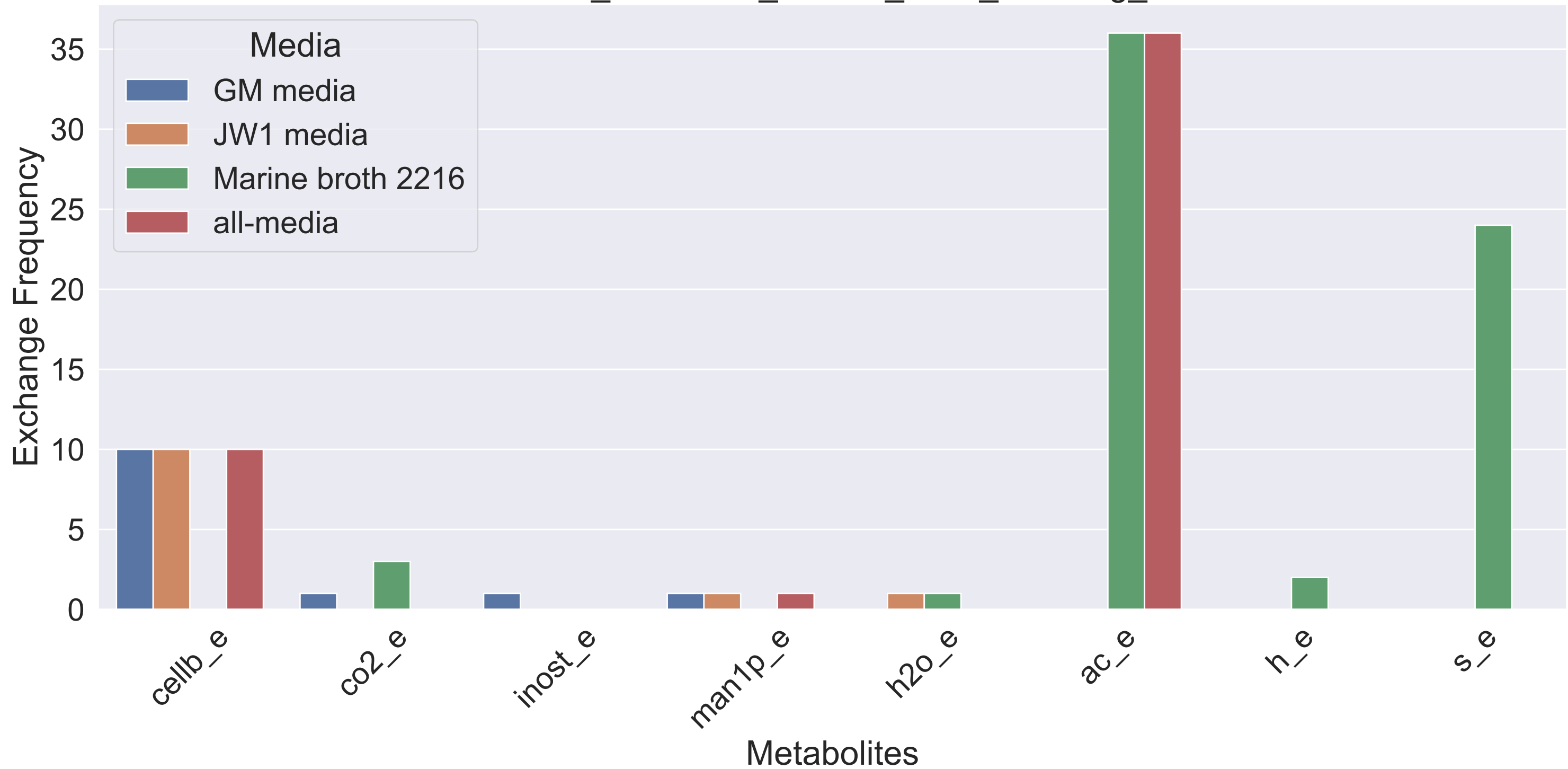

Supplement: Supplementary file 7 — Supplementary File S6 [file 43705_2023_242_MOESM7_ESM.zip › Supplementary Figure S135 Flavobacteriales bacterium UWMA 0289 donating metabolites.pdf]

# Fuerstia\_sp\_UWMA\_0333\_donating\_metabolites

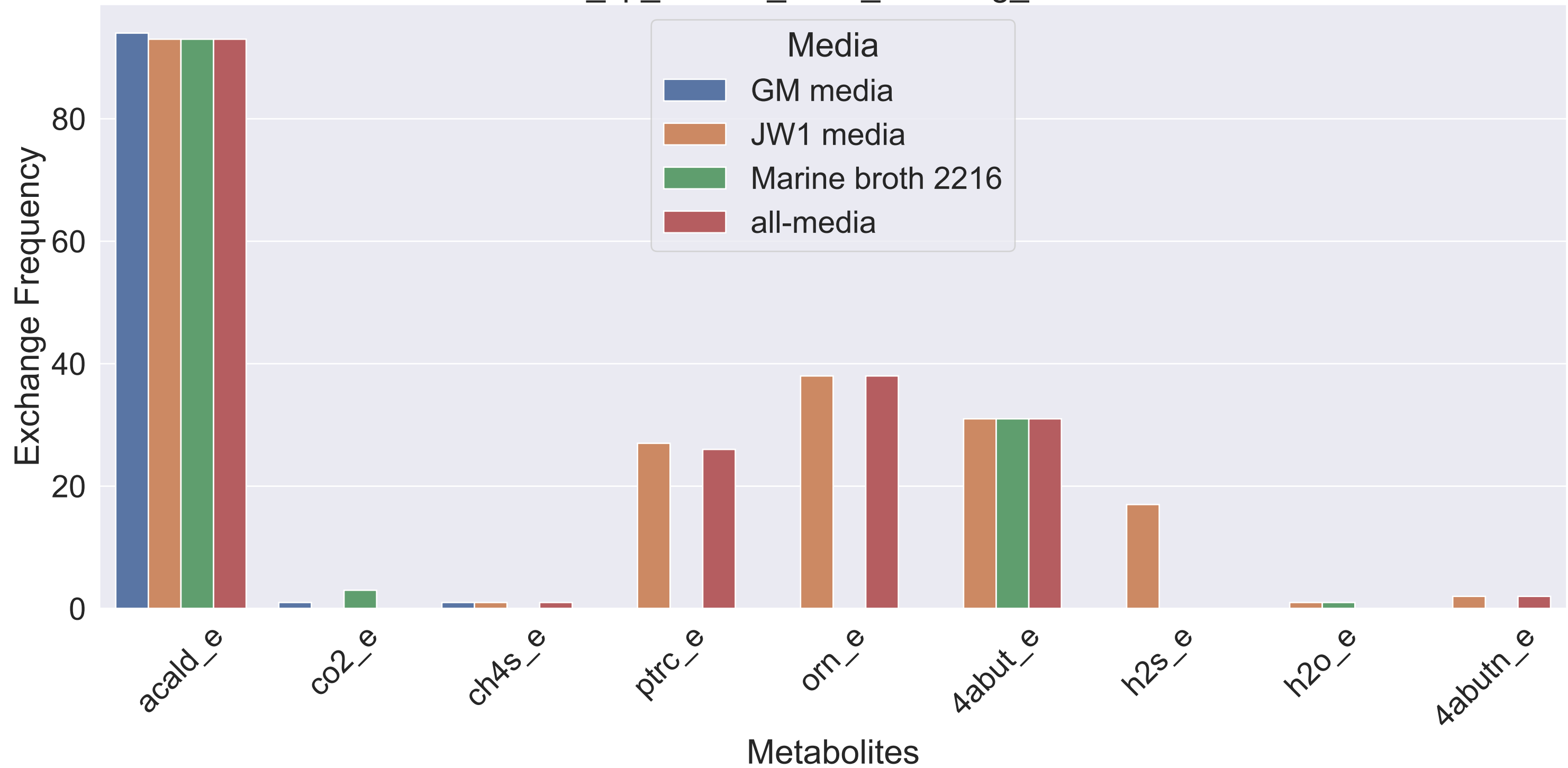

Supplement: Supplementary file 7 — Supplementary File S6 [file 43705_2023_242_MOESM7_ESM.zip › Supplementary Figure S136 Fuerstia sp UWMA 0333 donating metabolites.pdf]

Gammaproteobacteria\_bacterium\_UWMA\_0258\_donating\_metabolites

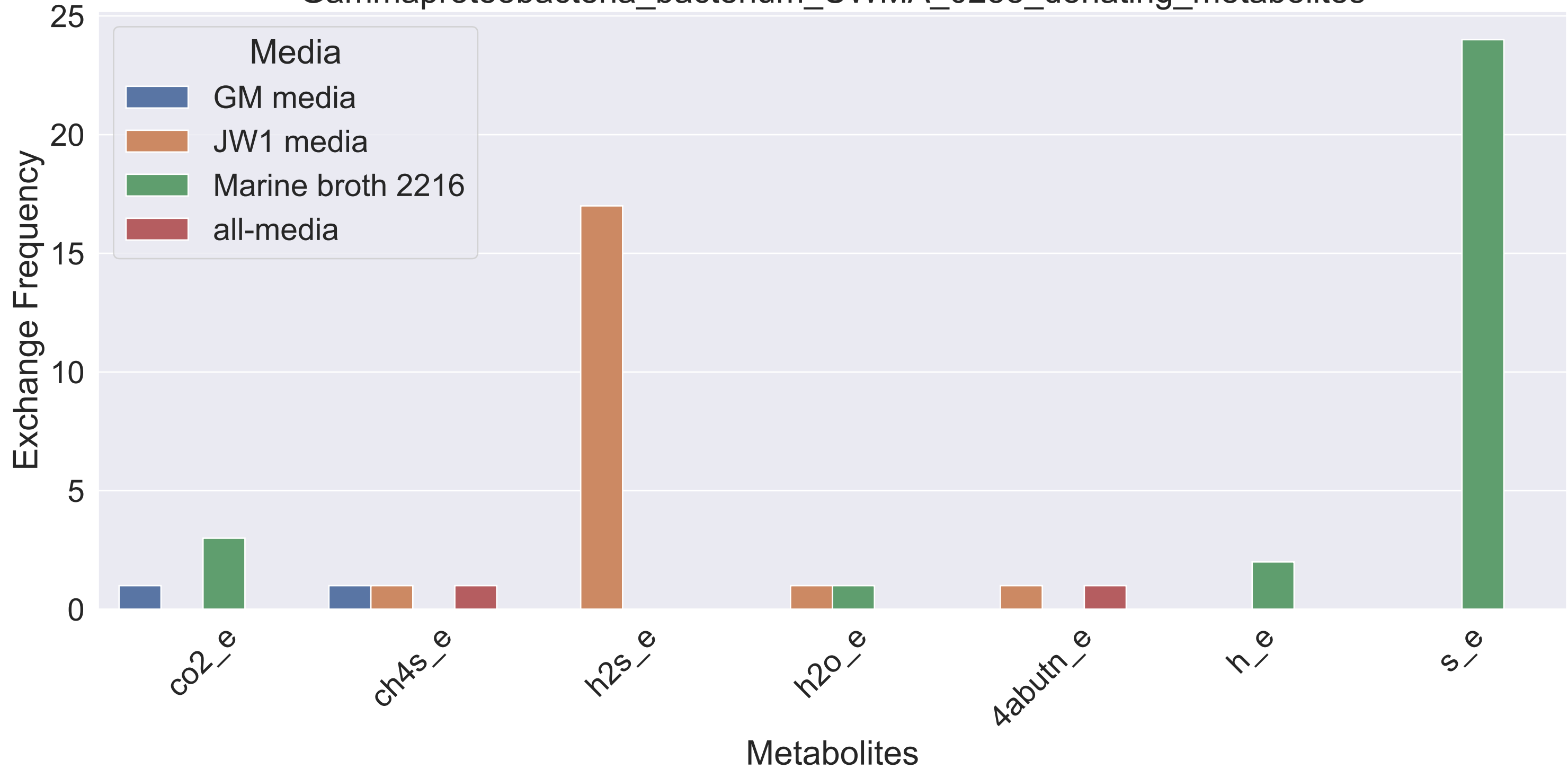

Supplement: Supplementary file 7 — Supplementary File S6 [file 43705_2023_242_MOESM7_ESM.zip › Supplementary Figure S137 Gammaproteobacteria bacterium UWMA 0258 donating metabolites.pdf]

# Gammaproteobacteria\_bacterium\_UWMA\_0260\_donating\_metabolites

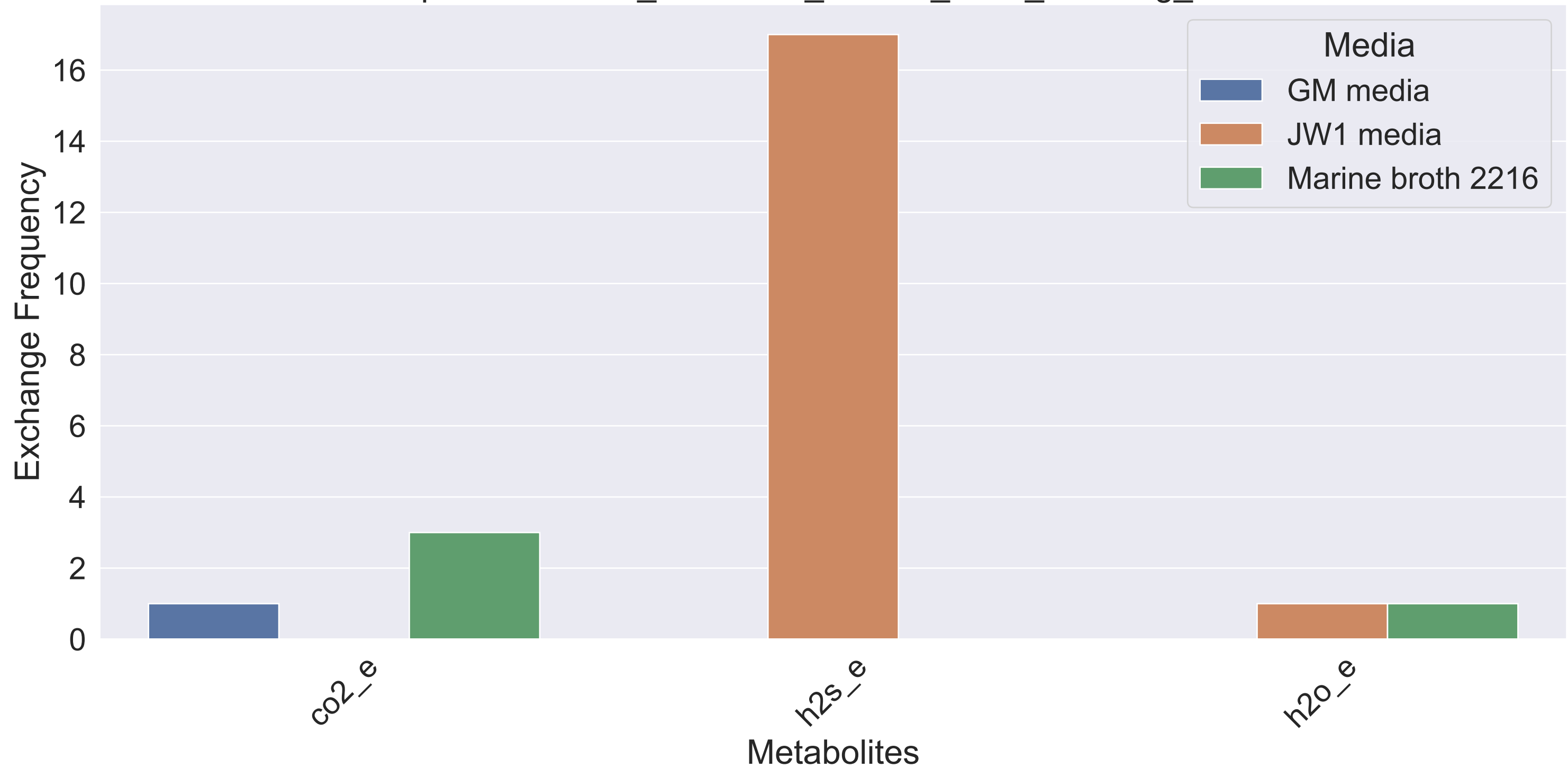

Supplement: Supplementary file 7 — Supplementary File S6 [file 43705_2023_242_MOESM7_ESM.zip › Supplementary Figure S138 Gammaproteobacteria bacterium UWMA 0260 donating metabolites.pdf]

Gammaproteobacteria\_bacterium\_UWMA\_0261\_donating\_metabolites

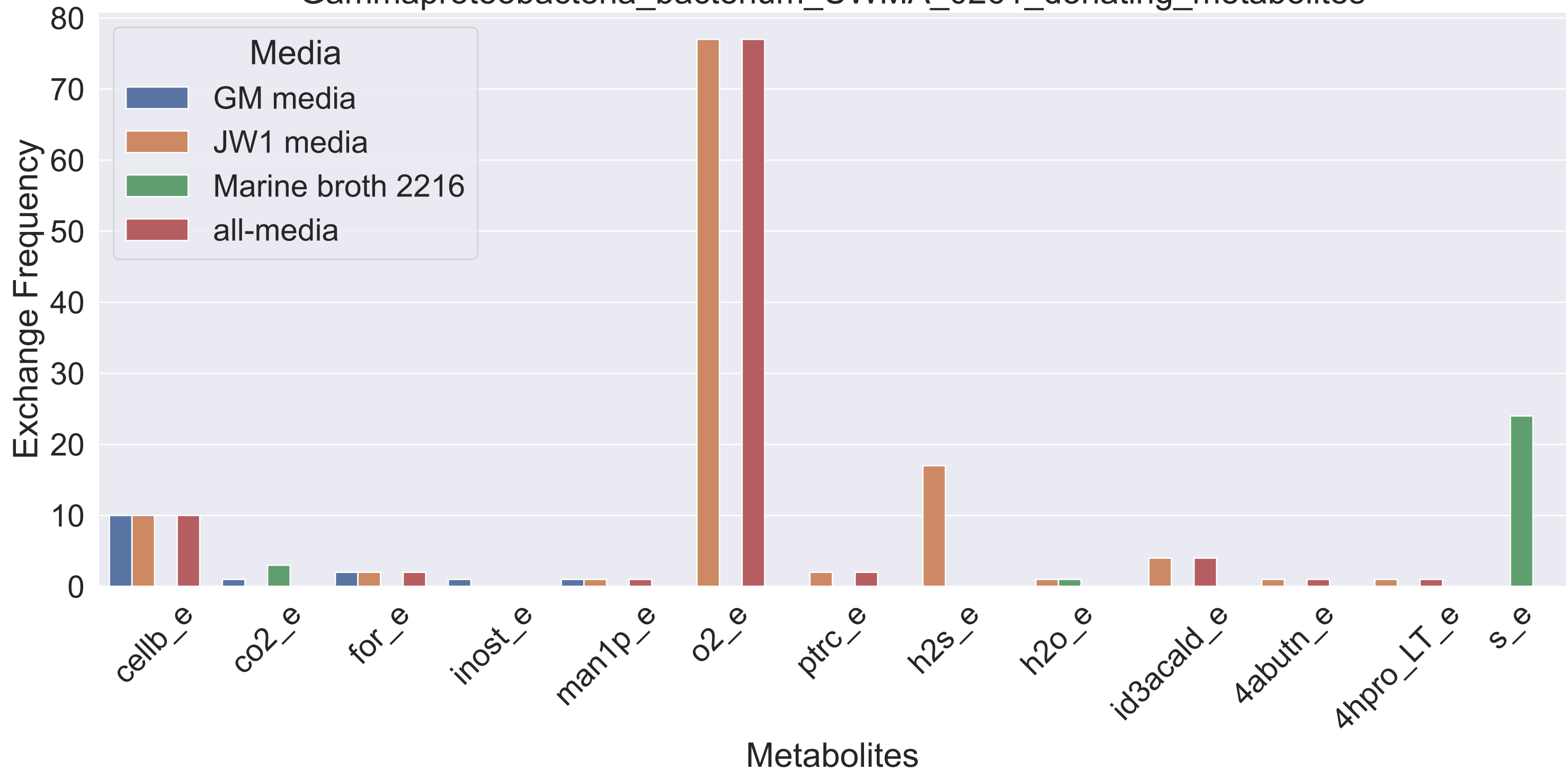

Supplement: Supplementary file 7 — Supplementary File S6 [file 43705_2023_242_MOESM7_ESM.zip › Supplementary Figure S139 Gammaproteobacteria bacterium UWMA 0261 donating metabolites.pdf]

# Gammaproteobacteria\_bacterium\_UWMA\_0273\_donating\_metabolites

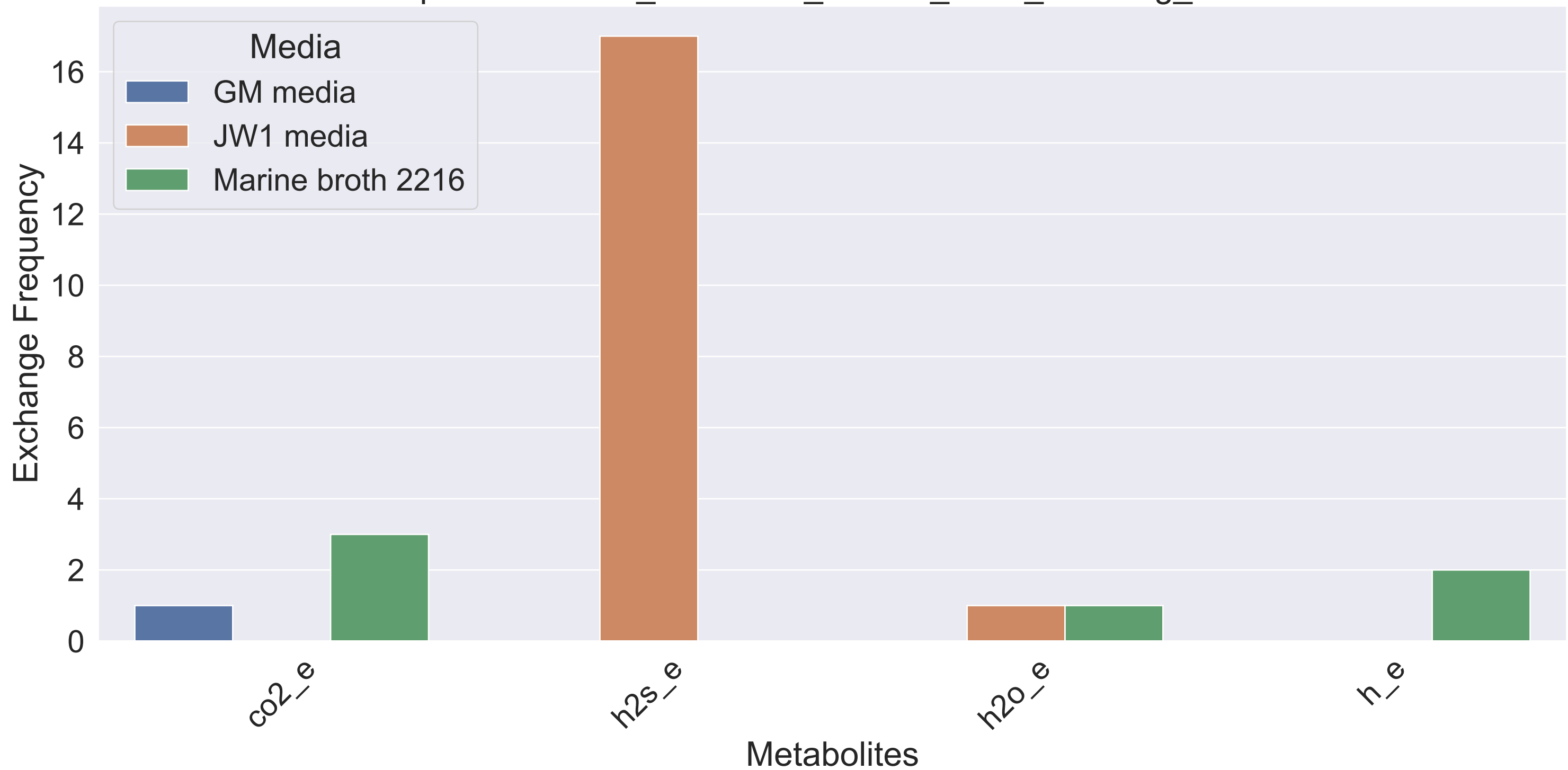

Supplement: Supplementary file 7 — Supplementary File S6 [file 43705_2023_242_MOESM7_ESM.zip › Supplementary Figure S140 Gammaproteobacteria bacterium UWMA 0273 donating metabolites.pdf]

Gammaproteobacteria\_bacterium\_UWMA\_0278\_donating\_metabolites

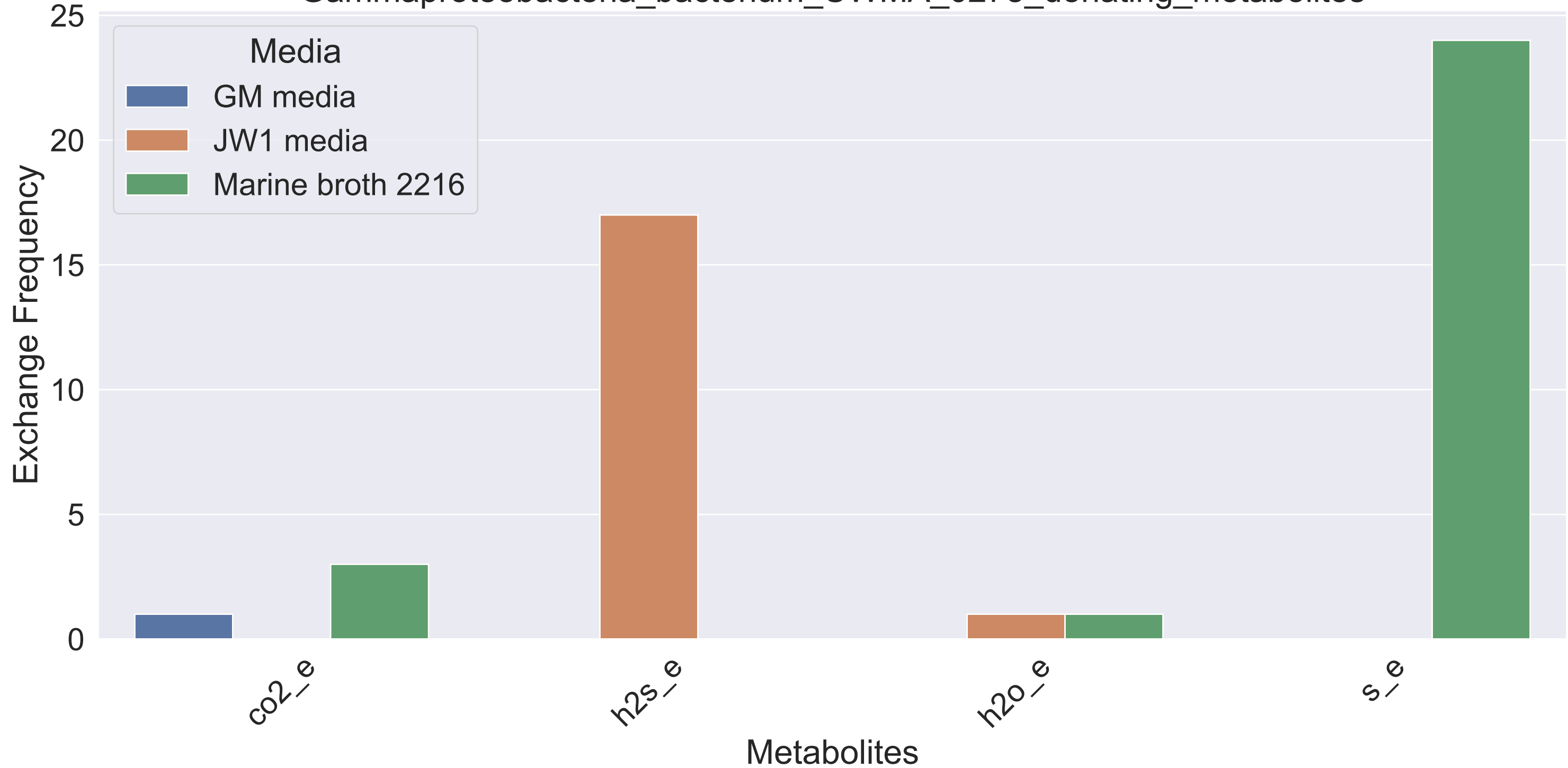

Supplement: Supplementary file 7 — Supplementary File S6 [file 43705_2023_242_MOESM7_ESM.zip › Supplementary Figure S141 Gammaproteobacteria bacterium UWMA 0278 donating metabolites.pdf]

# Gammaproteobacteria\_bacterium\_UWMA\_0281\_donating\_metabolites

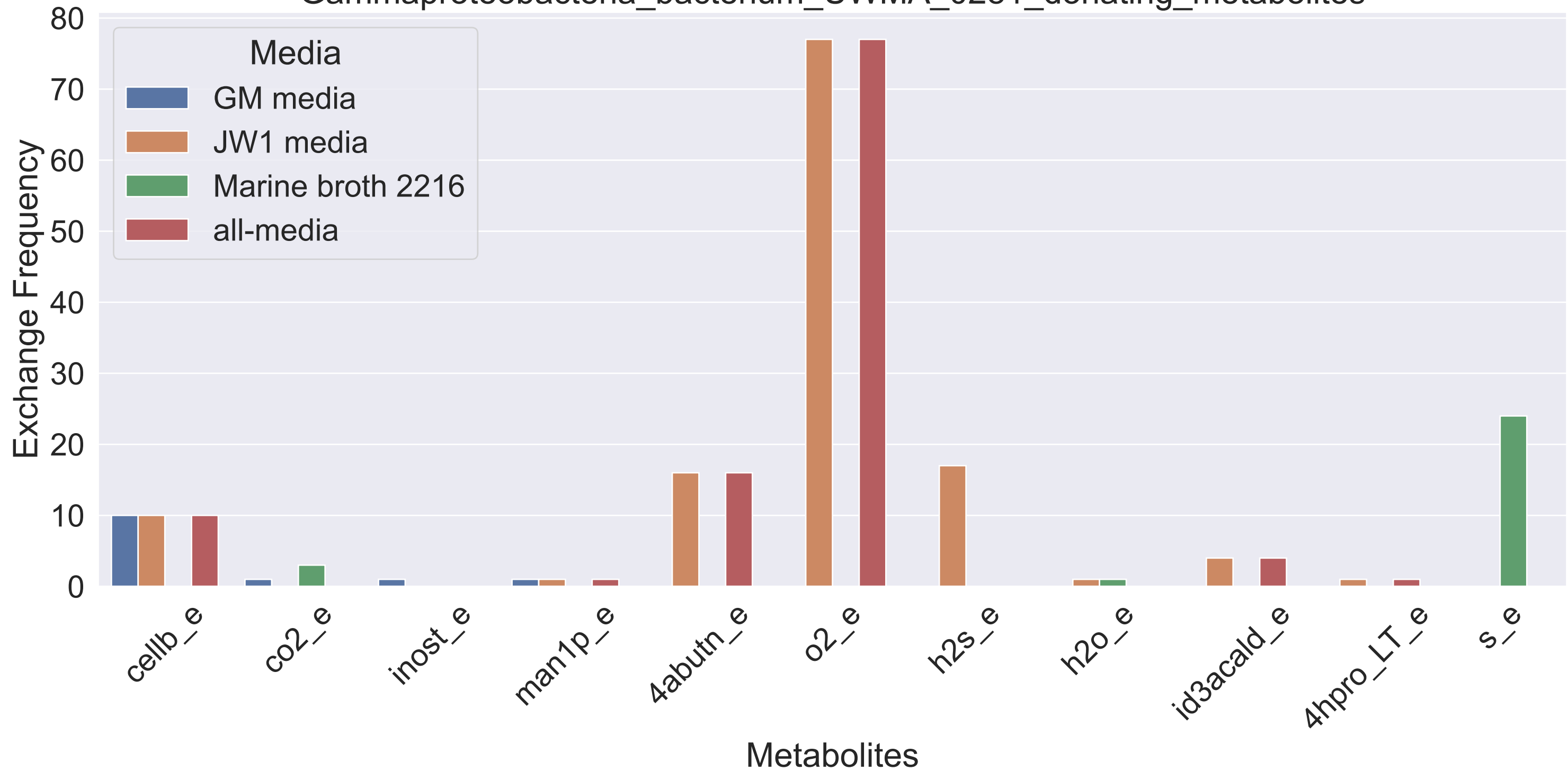

Supplement: Supplementary file 7 — Supplementary File S6 [file 43705_2023_242_MOESM7_ESM.zip › Supplementary Figure S142 Gammaproteobacteria bacterium UWMA 0281 donating metabolites.pdf]

# Gammaproteobacteria\_bacterium\_UWMA\_0282\_donating\_metabolites

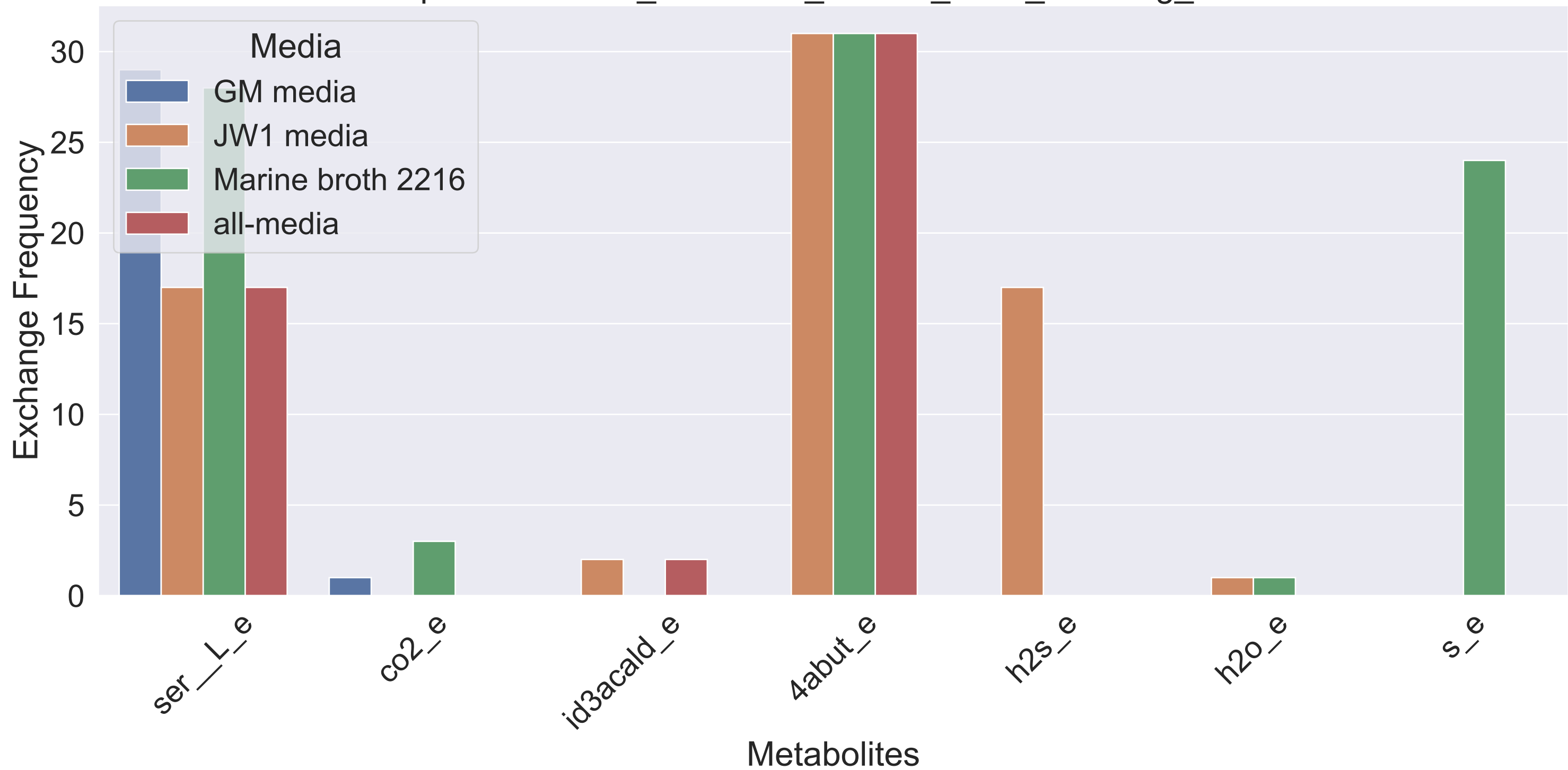

Supplement: Supplementary file 7 — Supplementary File S6 [file 43705_2023_242_MOESM7_ESM.zip › Supplementary Figure S143 Gammaproteobacteria bacterium UWMA 0282 donating metabolites.pdf]

# Gammaproteobacteria\_bacterium\_UWMA\_0291\_donating\_metabolites

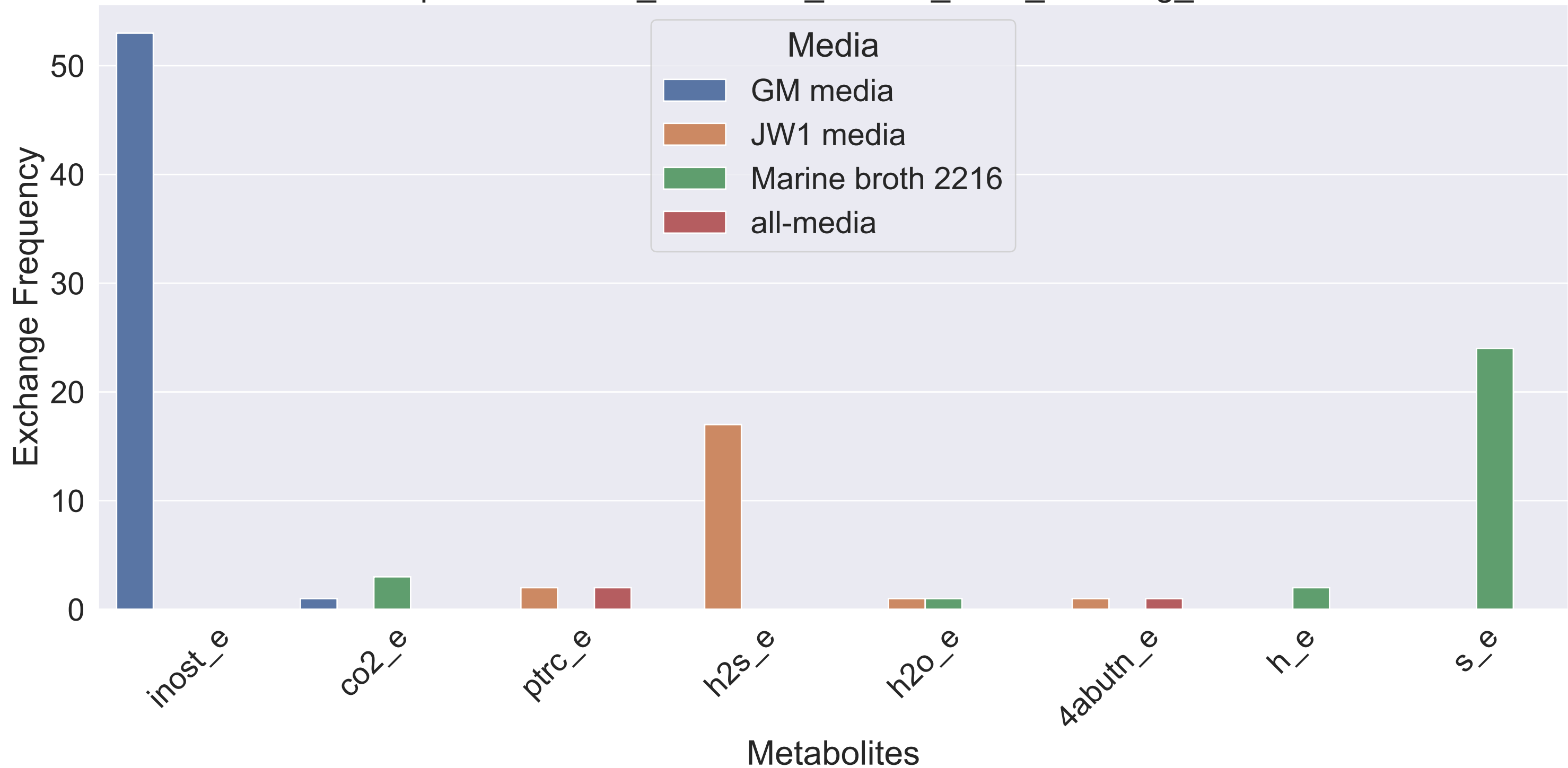

Supplement: Supplementary file 7 — Supplementary File S6 [file 43705_2023_242_MOESM7_ESM.zip › Supplementary Figure S144 Gammaproteobacteria bacterium UWMA 0291 donating metabolites.pdf]

Gammaproteobacteria\_bacterium\_UWMA\_0299\_donating\_metabolites

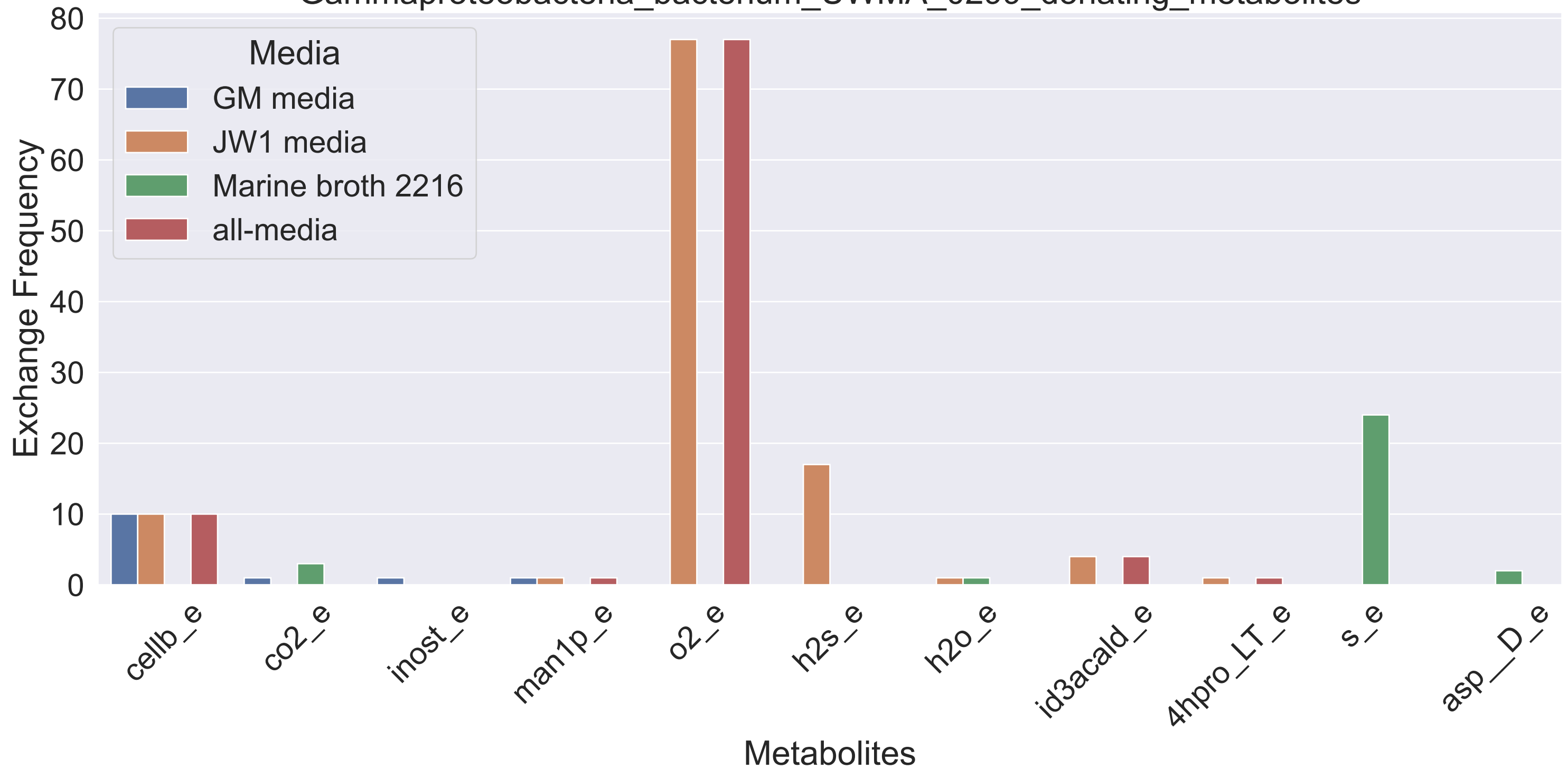

Supplement: Supplementary file 7 — Supplementary File S6 [file 43705_2023_242_MOESM7_ESM.zip › Supplementary Figure S145 Gammaproteobacteria bacterium UWMA 0299 donating metabolites.pdf]

# Gammaproteobacteria\_bacterium\_UWMA\_0301\_donating\_metabolites

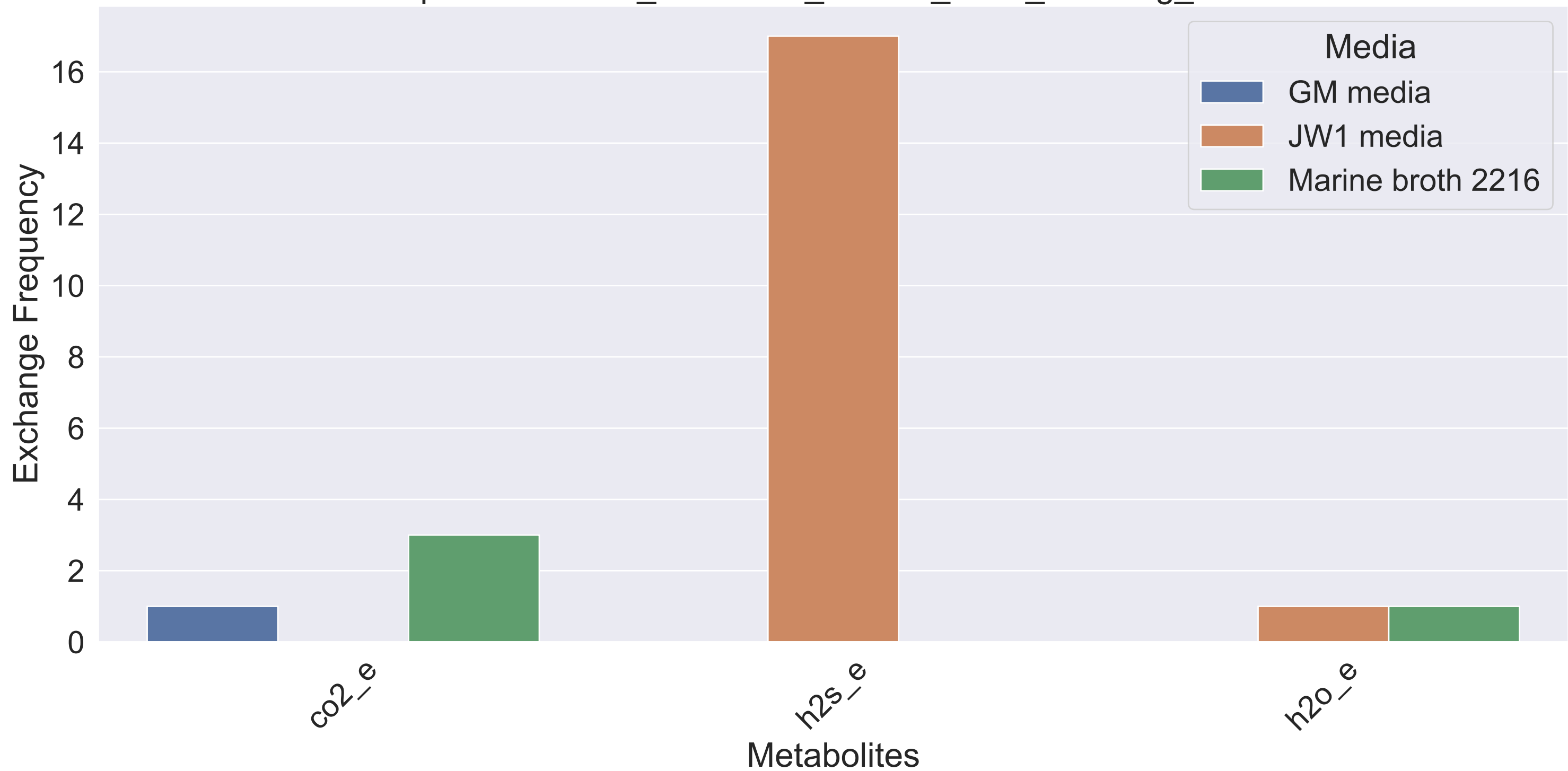

Supplement: Supplementary file 7 — Supplementary File S6 [file 43705_2023_242_MOESM7_ESM.zip › Supplementary Figure S146 Gammaproteobacteria bacterium UWMA 0301 donating metabolites.pdf]

Gammaproteobacteria\_bacterium\_UWMA\_0302\_donating\_metabolites

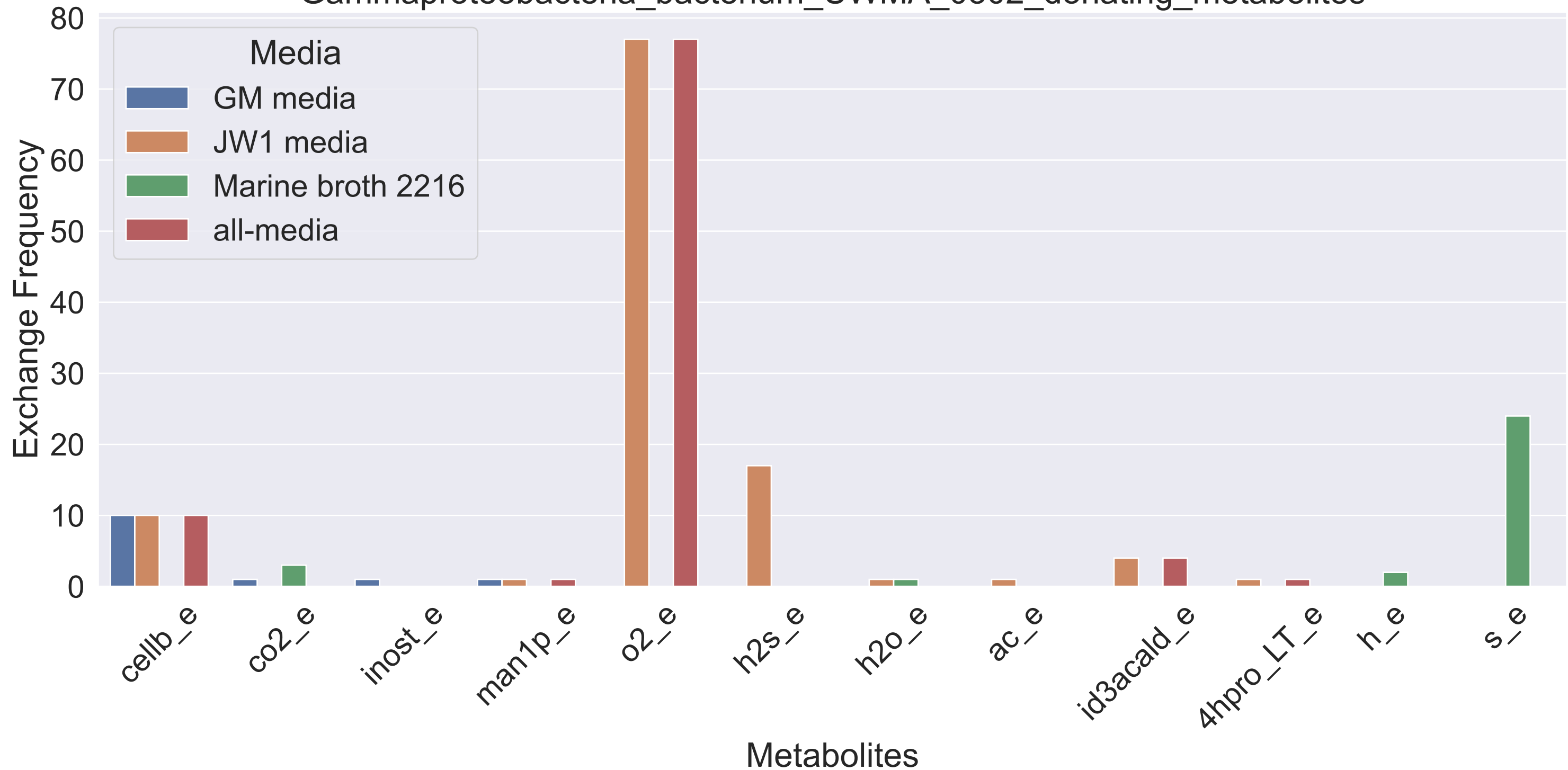

Supplement: Supplementary file 7 — Supplementary File S6 [file 43705_2023_242_MOESM7_ESM.zip › Supplementary Figure S147 Gammaproteobacteria bacterium UWMA 0302 donating metabolites.pdf]

# Gammaproteobacteria\_bacterium\_UWMA\_0343\_donating\_metabolites

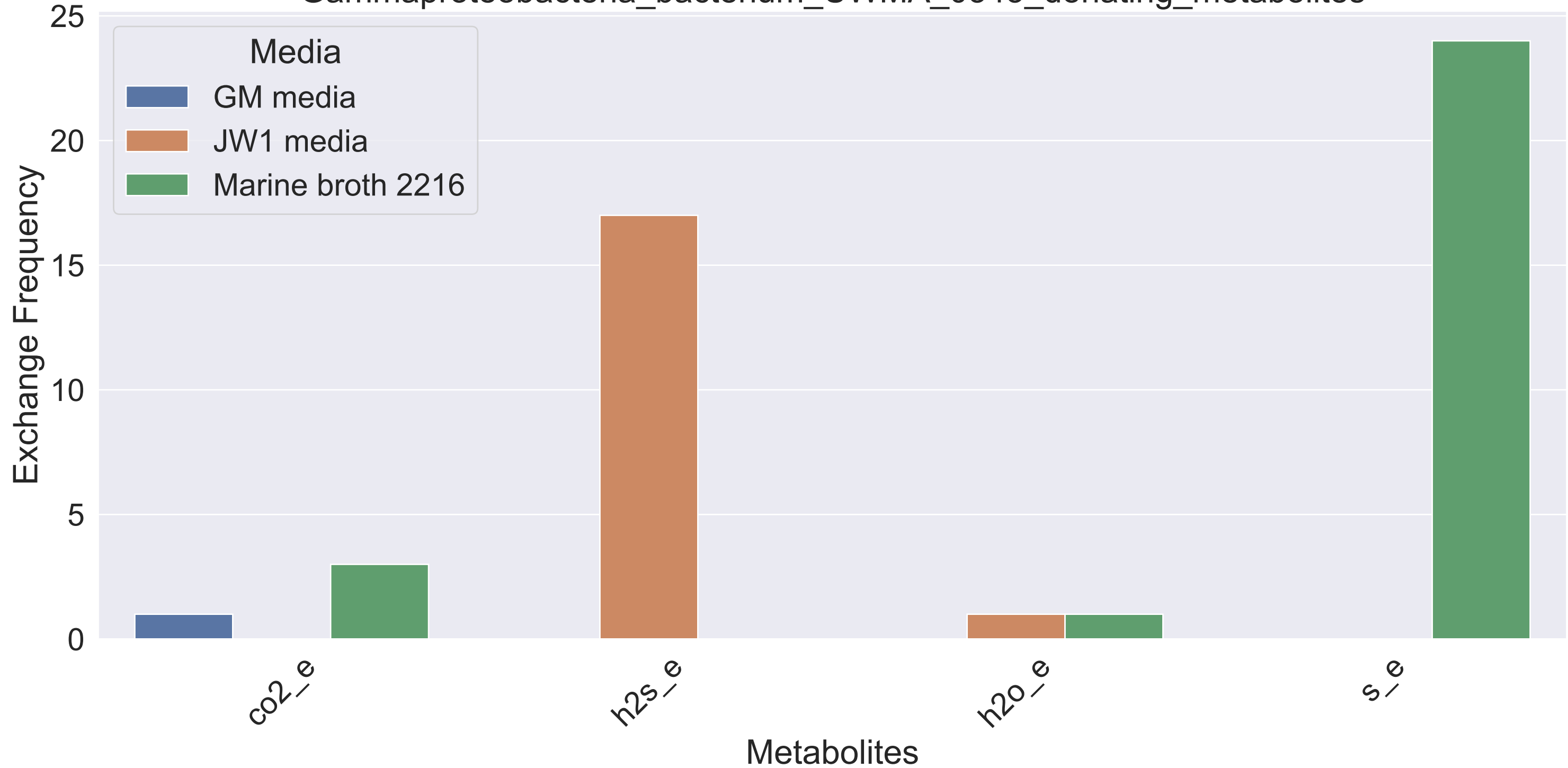

Supplement: Supplementary file 7 — Supplementary File S6 [file 43705_2023_242_MOESM7_ESM.zip › Supplementary Figure S148 Gammaproteobacteria bacterium UWMA 0343 donating metabolites.pdf]

# Gemmatimonadetes\_bacterium\_UWMA\_0296\_donating\_metabolites

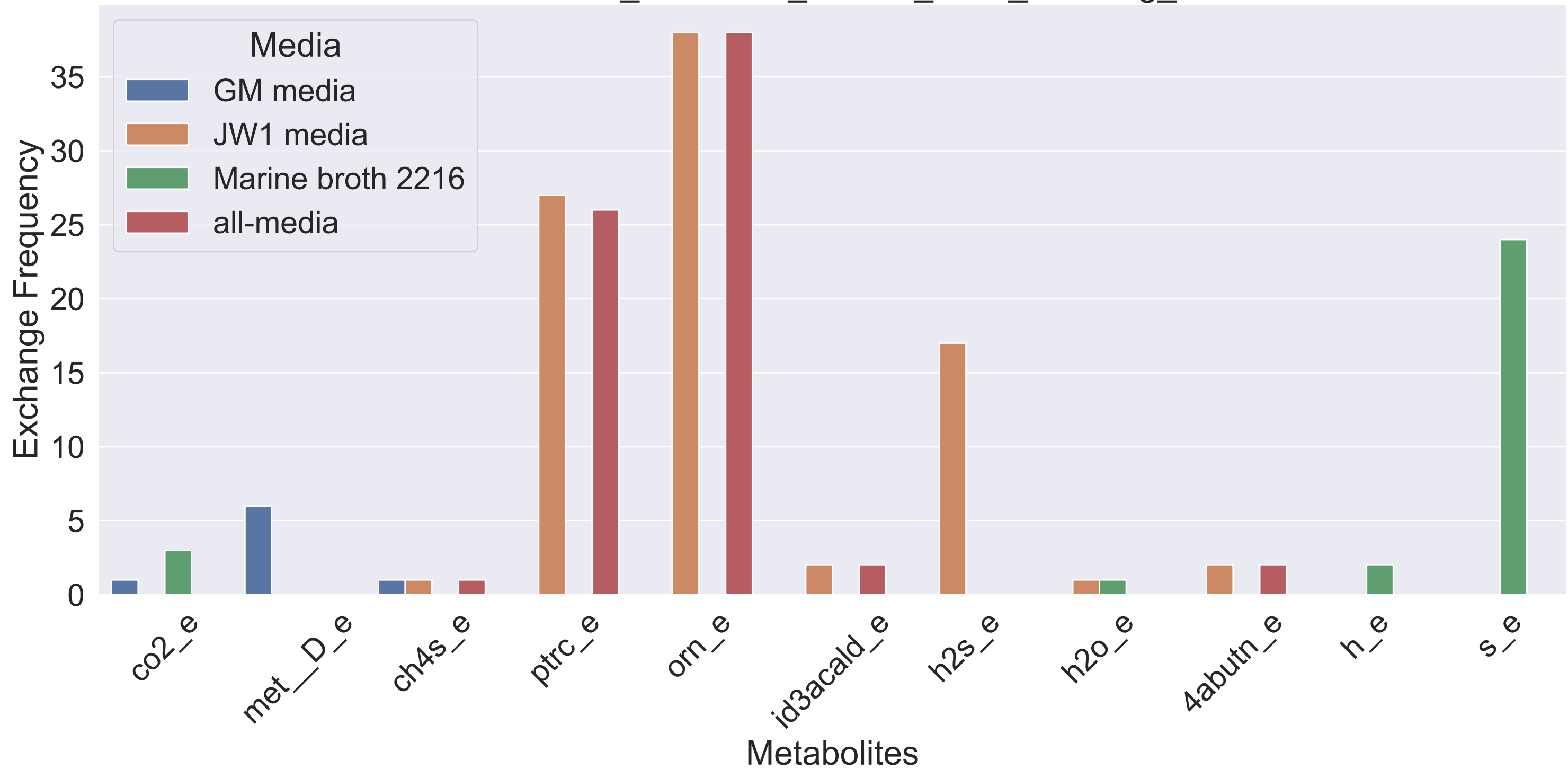

Supplement: Supplementary file 7 — Supplementary File S6 [file 43705_2023_242_MOESM7_ESM.zip › Supplementary Figure S149 Gemmatimonadetes bacterium UWMA 0296 donating metabolites.pdf]

# Gemmatimonadetes\_bacterium\_UWMA\_0308\_donating\_metabolites

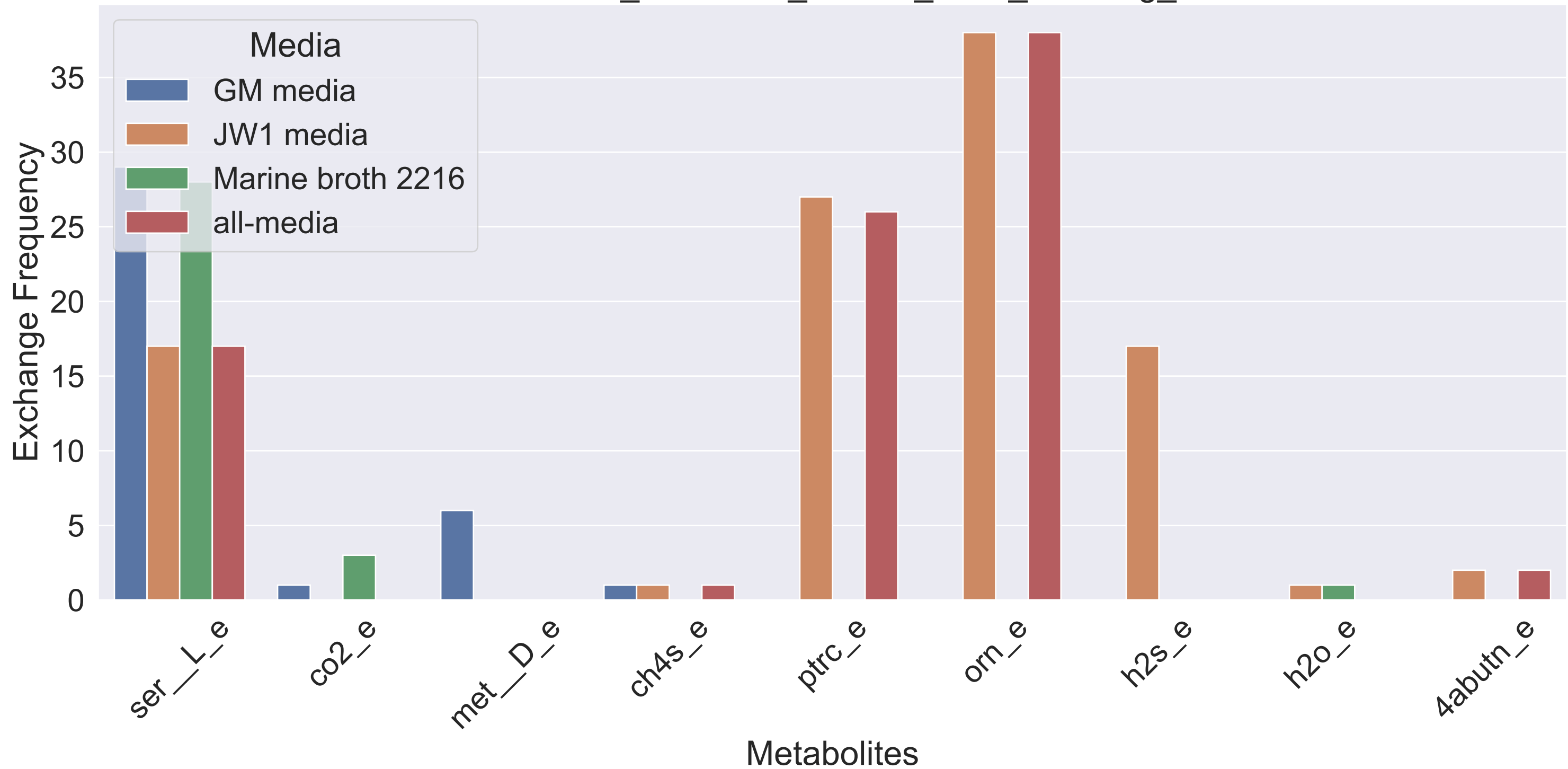

Supplement: Supplementary file 7 — Supplementary File S6 [file 43705_2023_242_MOESM7_ESM.zip › Supplementary Figure S150 Gemmatimonadetes bacterium UWMA 0308 donating metabolites.pdf]

# Gemmatimonadetes\_bacterium\_UWMA\_0334\_donating\_metabolites

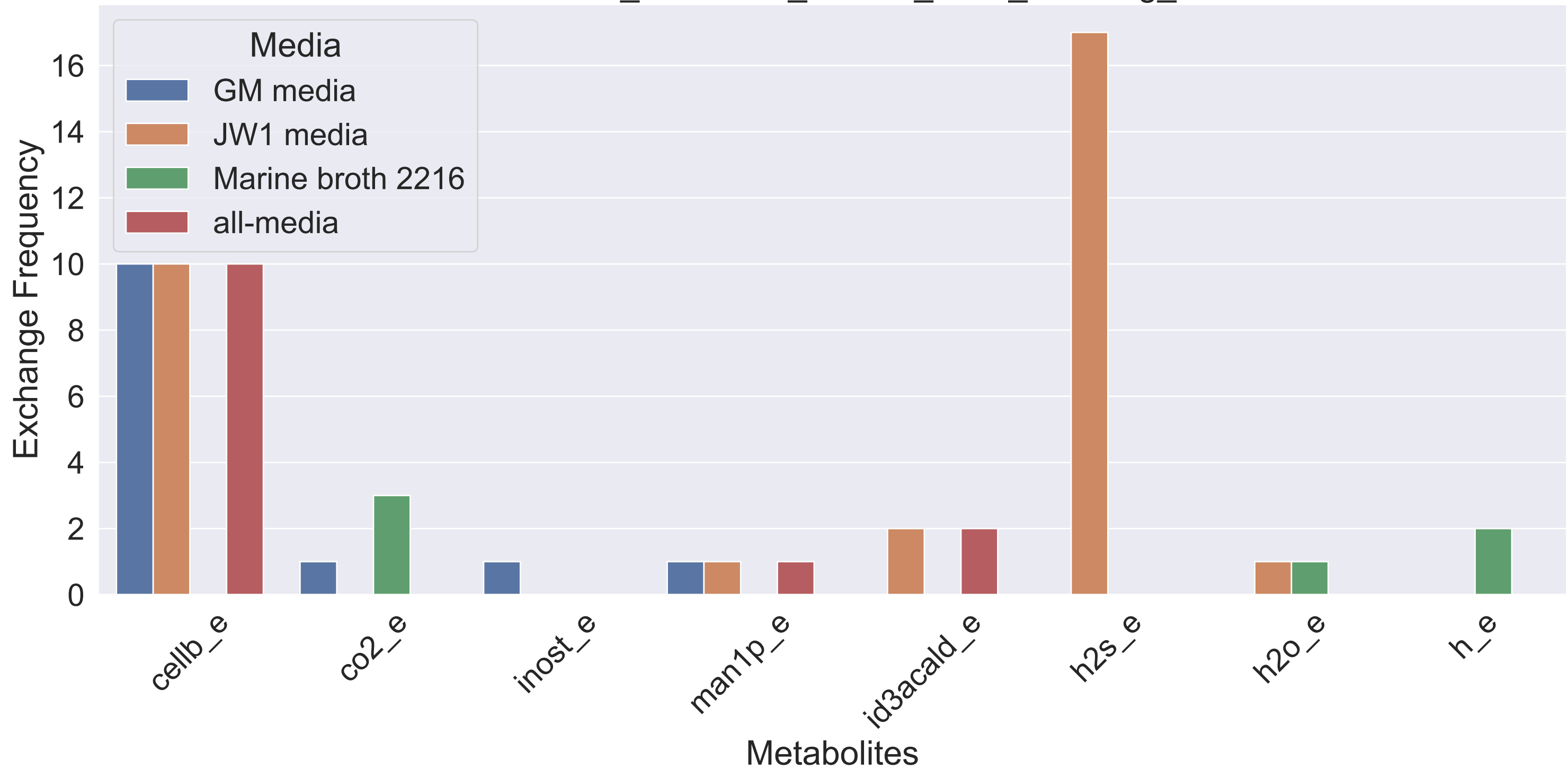

Supplement: Supplementary file 7 — Supplementary File S6 [file 43705_2023_242_MOESM7_ESM.zip › Supplementary Figure S151 Gemmatimonadetes bacterium UWMA 0334 donating metabolites.pdf]

Gemmatimonadetes\_bacterium\_UWMA\_0339\_donating\_metabolites

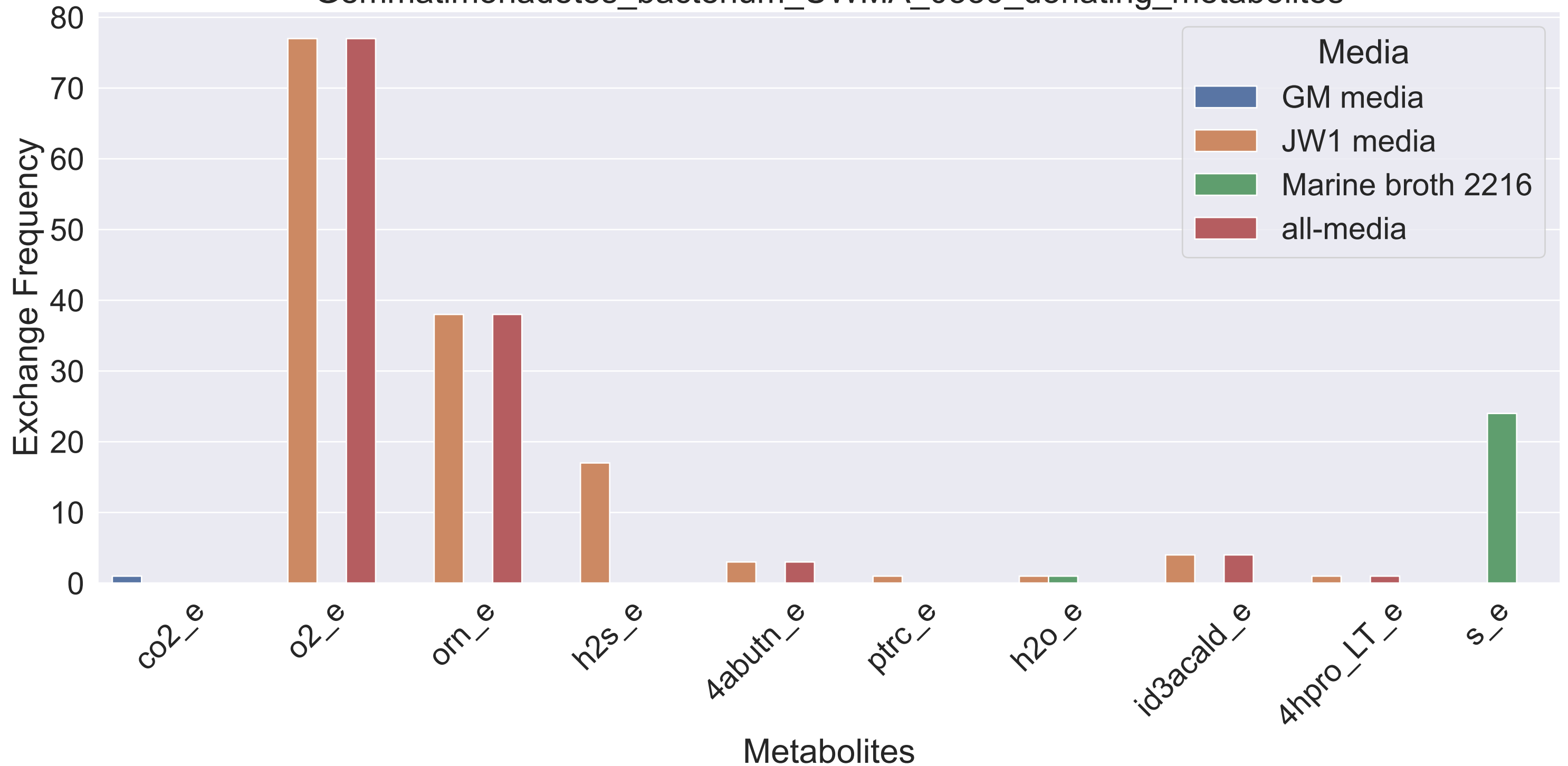

Supplement: Supplementary file 7 — Supplementary File S6 [file 43705_2023_242_MOESM7_ESM.zip › Supplementary Figure S152 Gemmatimonadetes bacterium UWMA 0339 donating metabolites.pdf]

# Henriciella\_sp\_UWMA\_0297\_donating\_metabolites

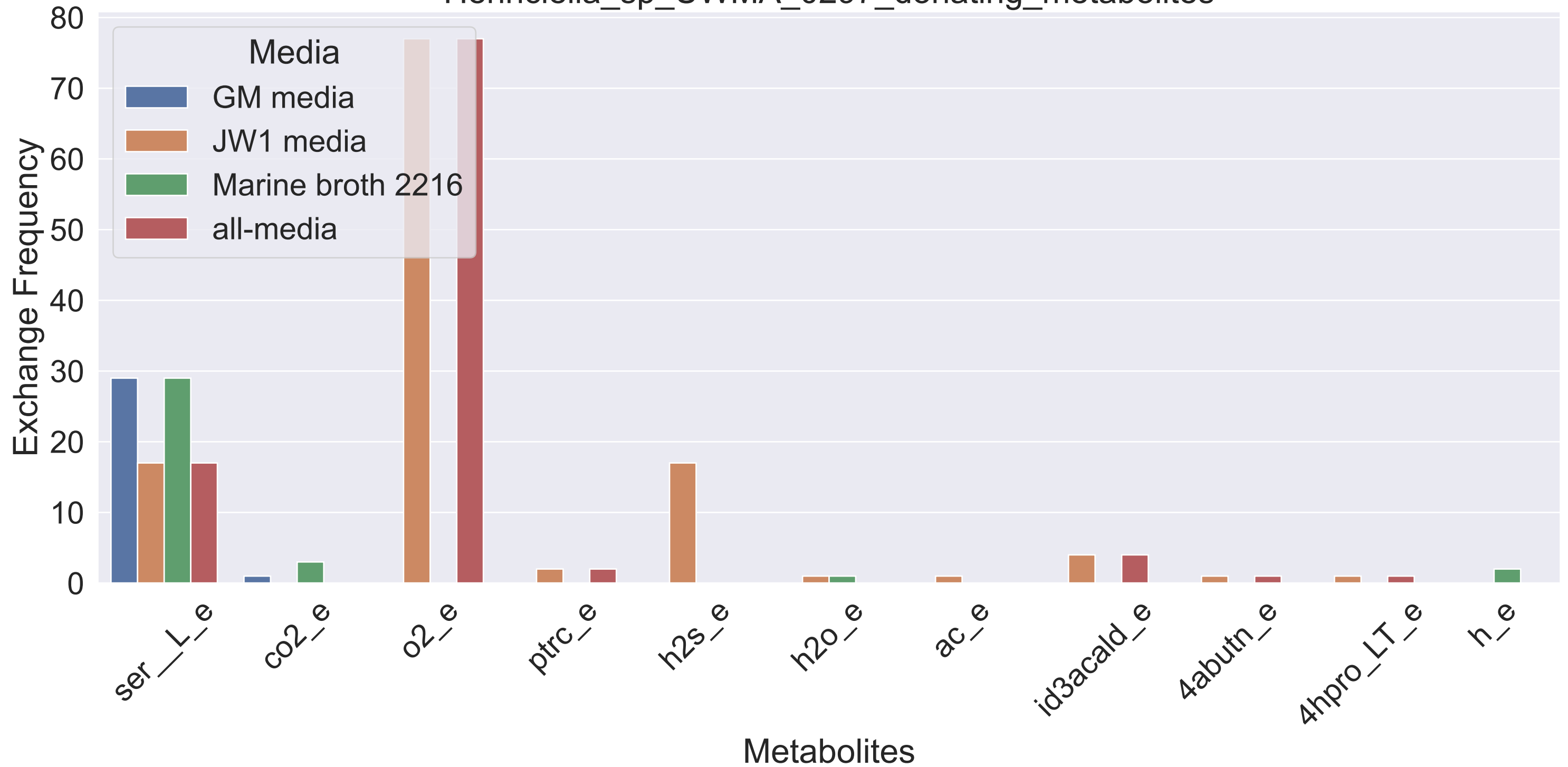

Supplement: Supplementary file 7 — Supplementary File S6 [file 43705_2023_242_MOESM7_ESM.zip › Supplementary Figure S153 Henriciella sp UWMA 0297 donating metabolites.pdf]

Marine\_Group\_III\_euryarchaeote\_UWMA\_0284\_donating\_metabolites

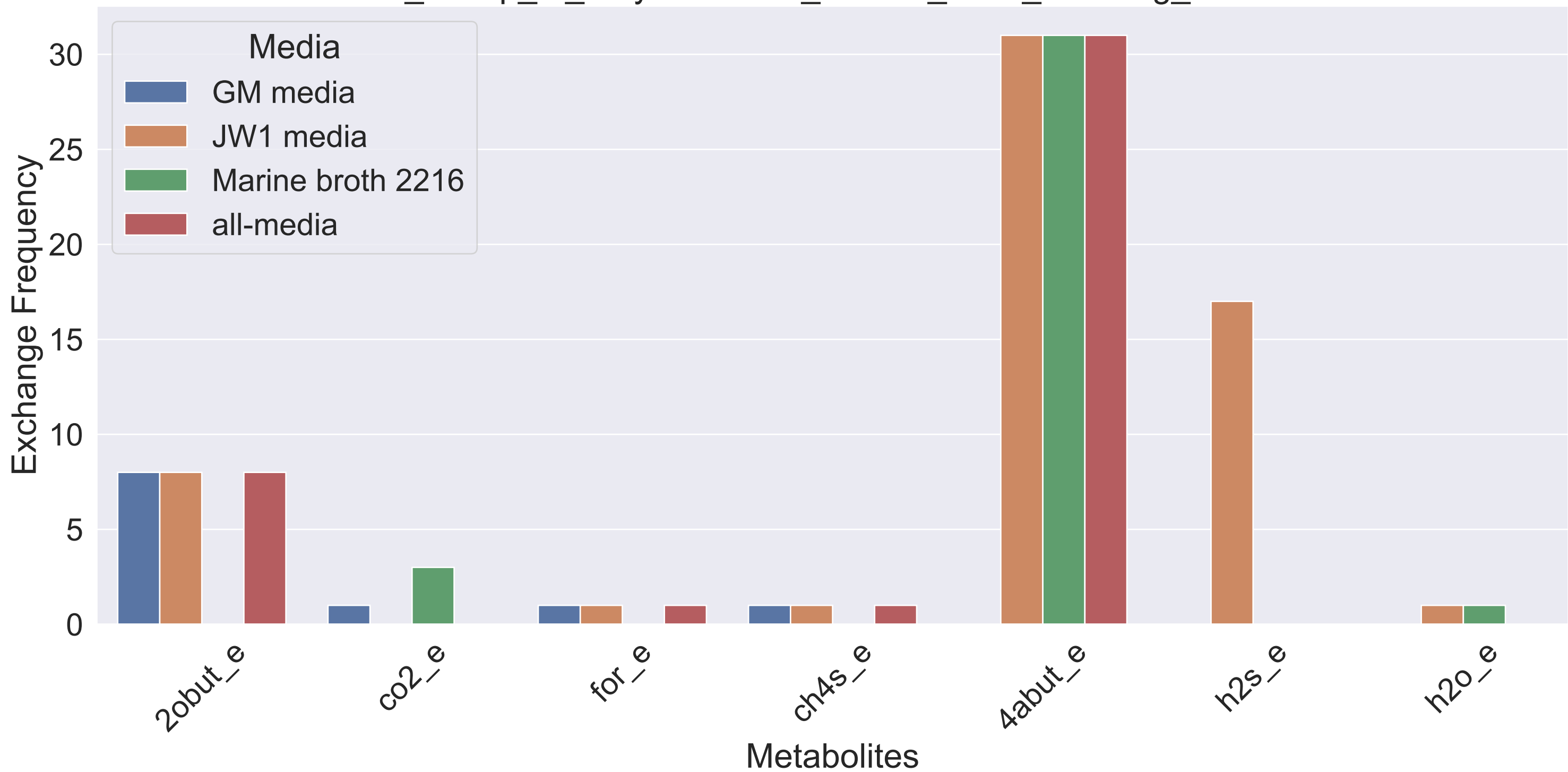

Supplement: Supplementary file 7 — Supplementary File S6 [file 43705_2023_242_MOESM7_ESM.zip › Supplementary Figure S154 Marine Group III euryarchaeote UWMA 0284 donating metabolites.pdf]

# Marine\_Group\_III\_euryarchaeote\_UWMA\_0340\_donating\_metabolites

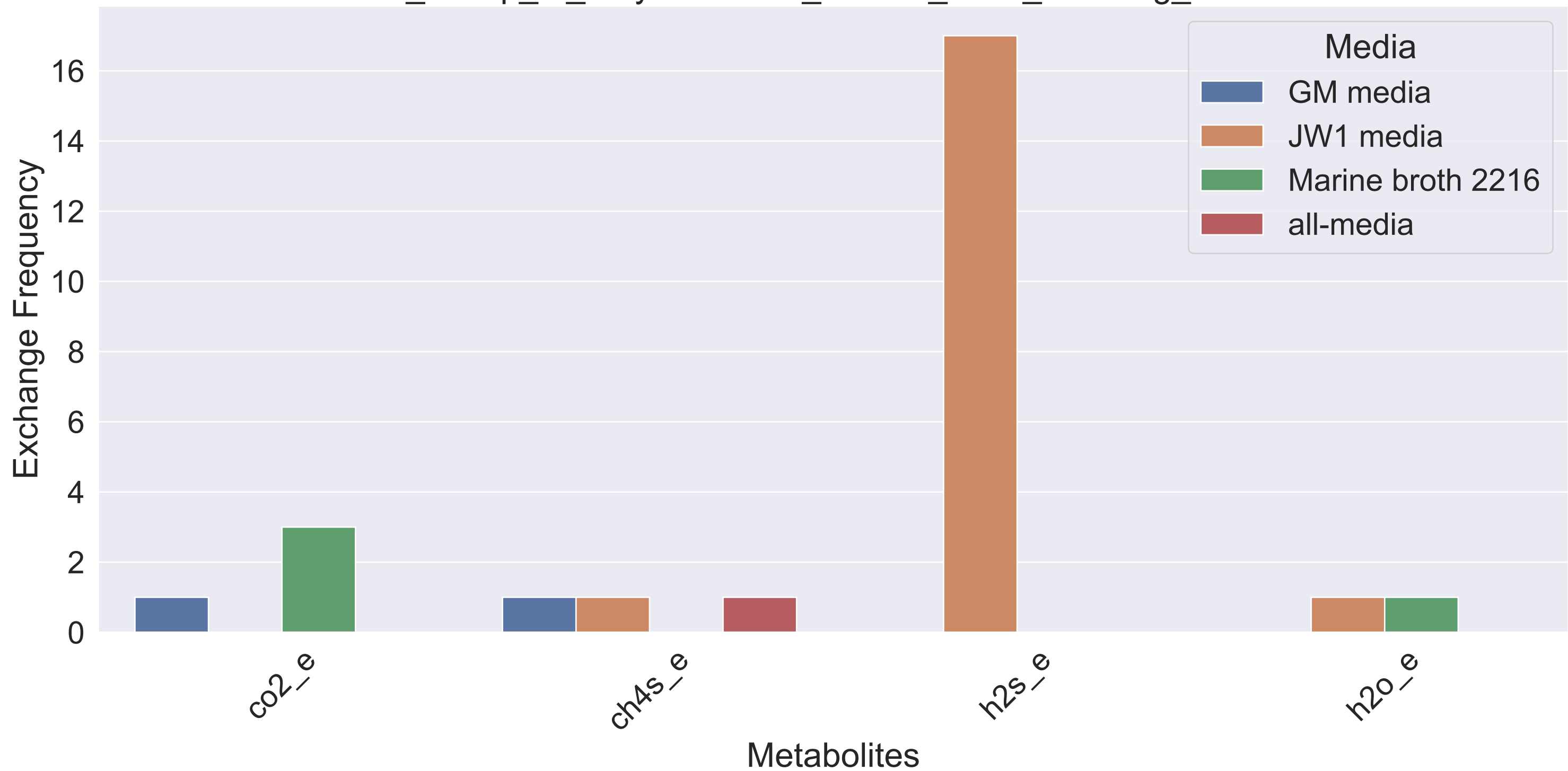

Supplement: Supplementary file 7 — Supplementary File S6 [file 43705_2023_242_MOESM7_ESM.zip › Supplementary Figure S155 Marine Group III euryarchaeote UWMA 0340 donating metabolites.pdf]

Marine\_Group\_II\_euryarchaeote\_UWMA\_0266\_donating\_metabolites

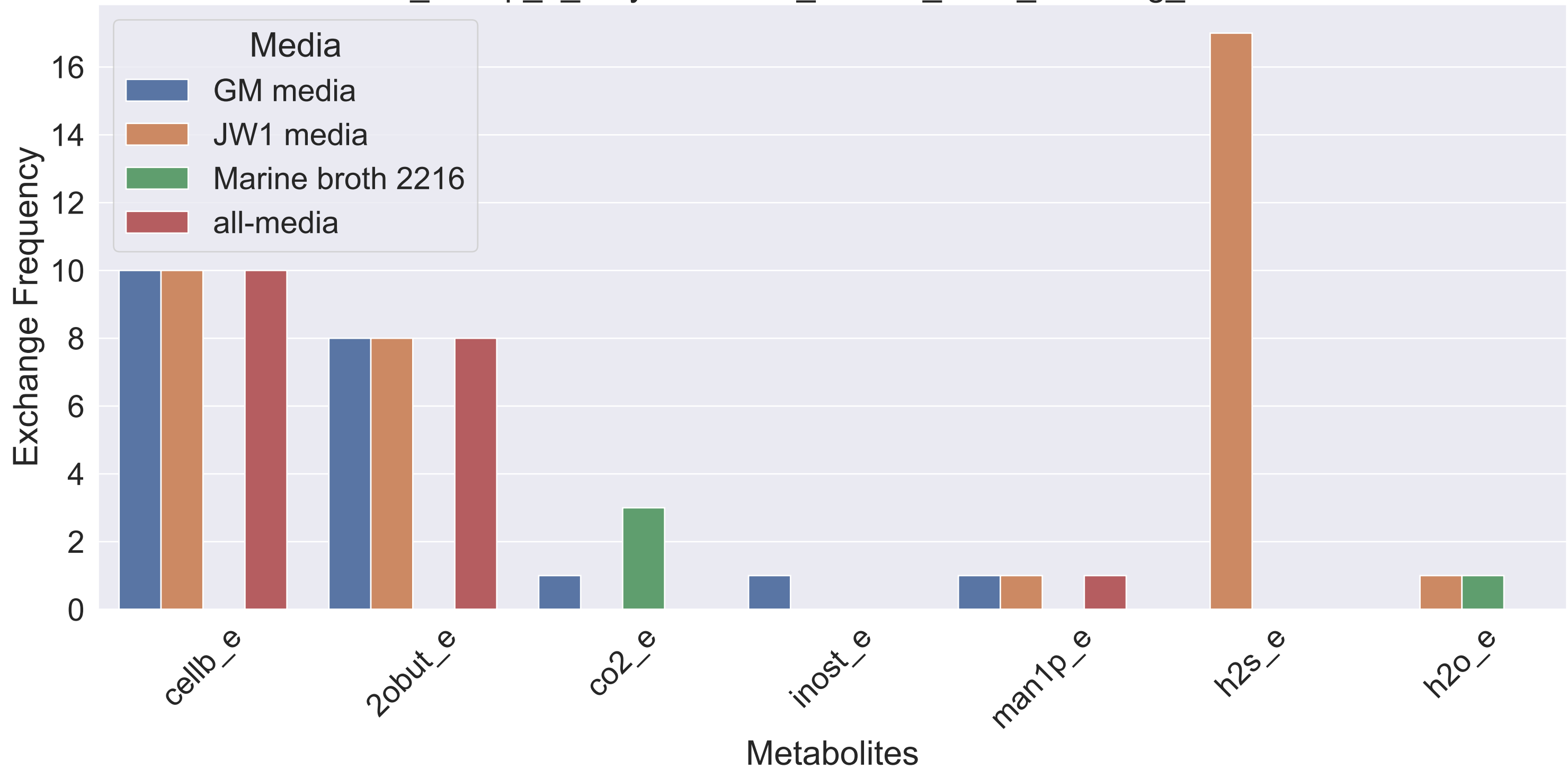

Supplement: Supplementary file 7 — Supplementary File S6 [file 43705_2023_242_MOESM7_ESM.zip › Supplementary Figure S156 Marine Group II euryarchaeote UWMA 0266 donating metabolites.pdf]

Marine\_Group\_II\_euryarchaeote\_UWMA\_0275\_donating\_metabolites

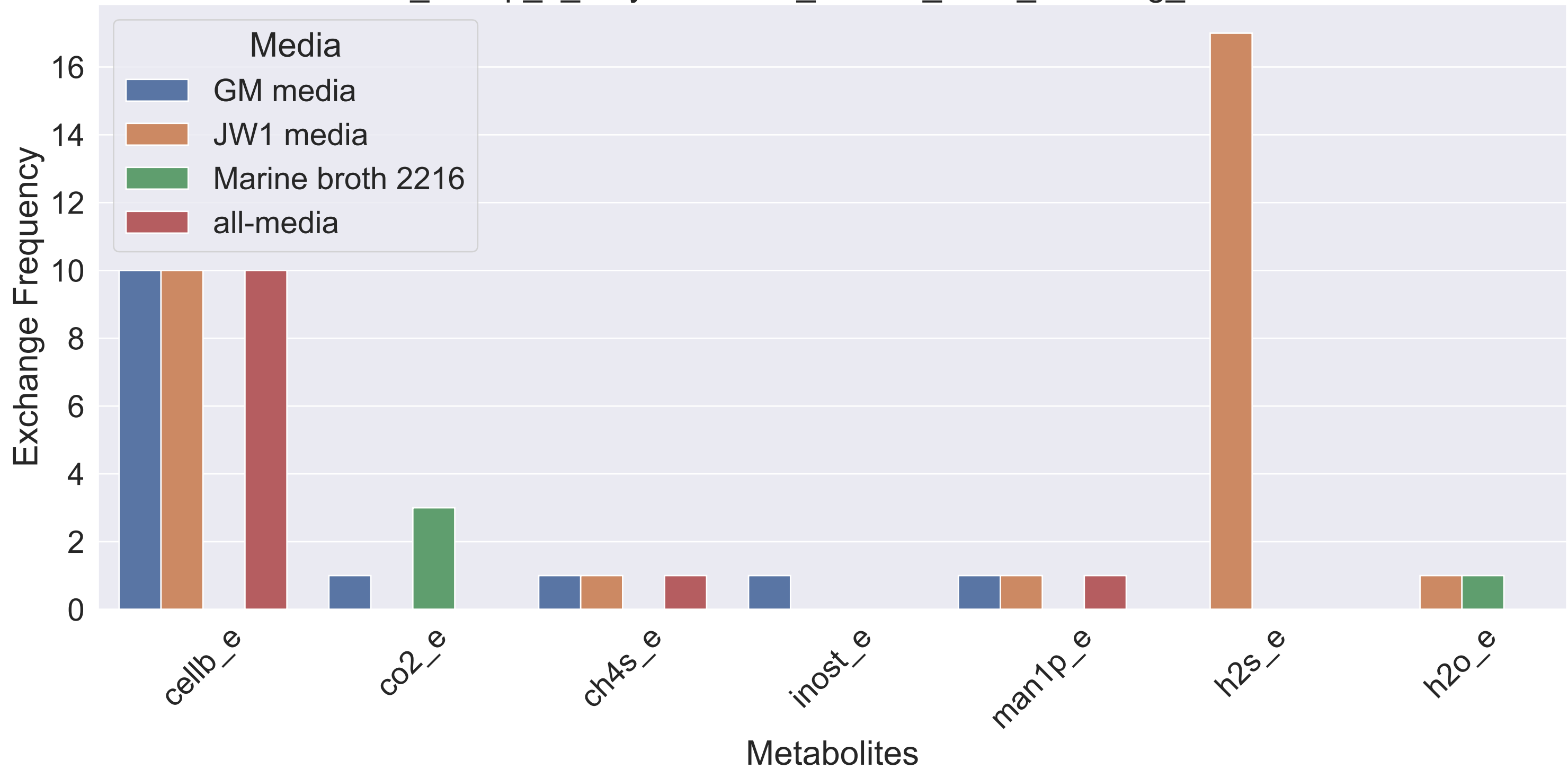

Supplement: Supplementary file 7 — Supplementary File S6 [file 43705_2023_242_MOESM7_ESM.zip › Supplementary Figure S157 Marine Group II euryarchaeote UWMA 0275 donating metabolites.pdf]

# Marine\_Group\_II\_euryarchaeote\_UWMA\_0279\_donating\_metabolites

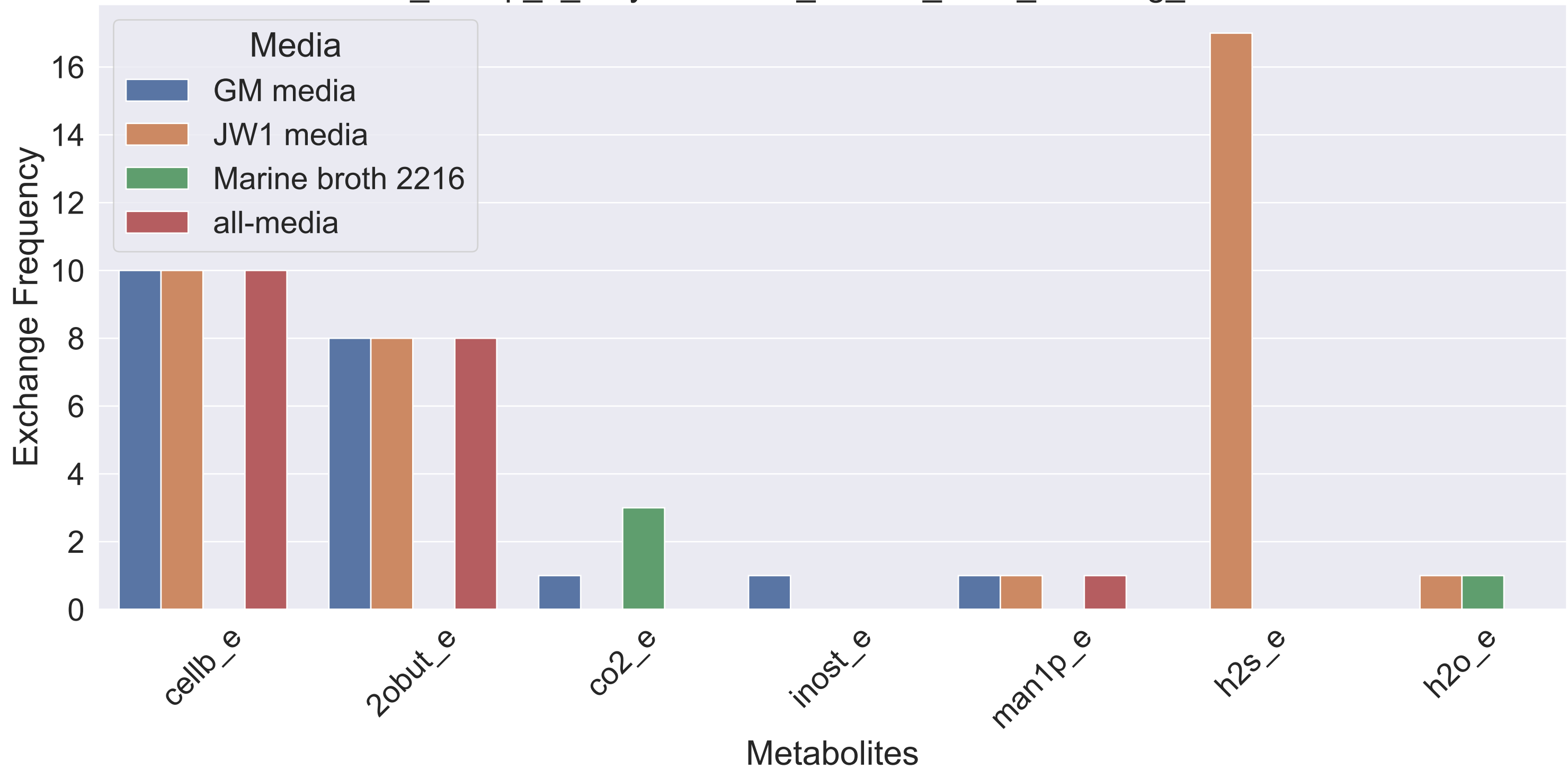

Supplement: Supplementary file 7 — Supplementary File S6 [file 43705_2023_242_MOESM7_ESM.zip › Supplementary Figure S158 Marine Group II euryarchaeote UWMA 0279 donating metabolites.pdf]

# Marine\_Group\_II\_euryarchaeote\_UWMA\_0283\_donating\_metabolites

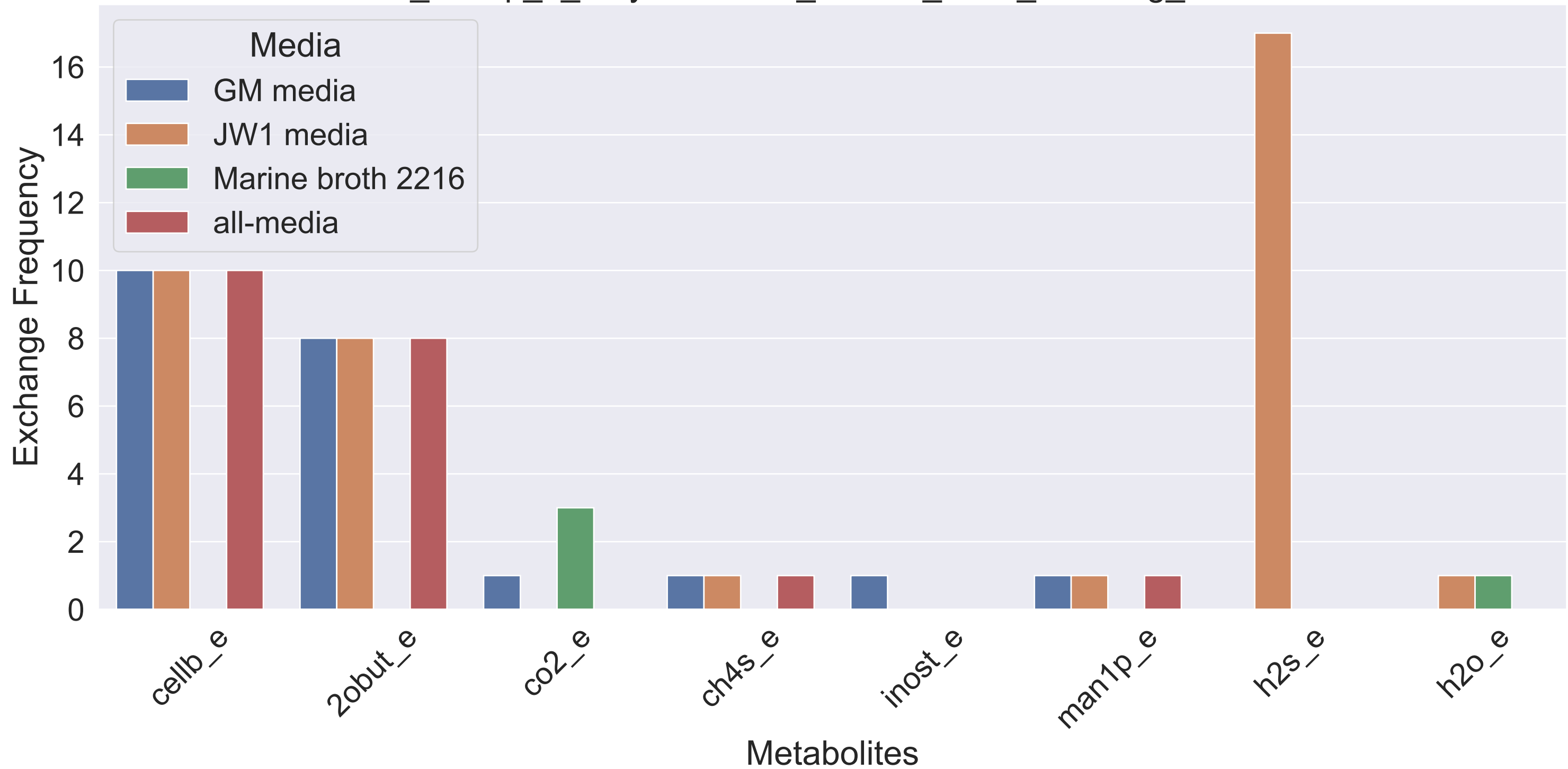

Supplement: Supplementary file 7 — Supplementary File S6 [file 43705_2023_242_MOESM7_ESM.zip › Supplementary Figure S159 Marine Group II euryarchaeote UWMA 0283 donating metabolites.pdf]

# Marine\_Group\_II\_euryarchaeote\_UWMA\_0323\_donating\_metabolites

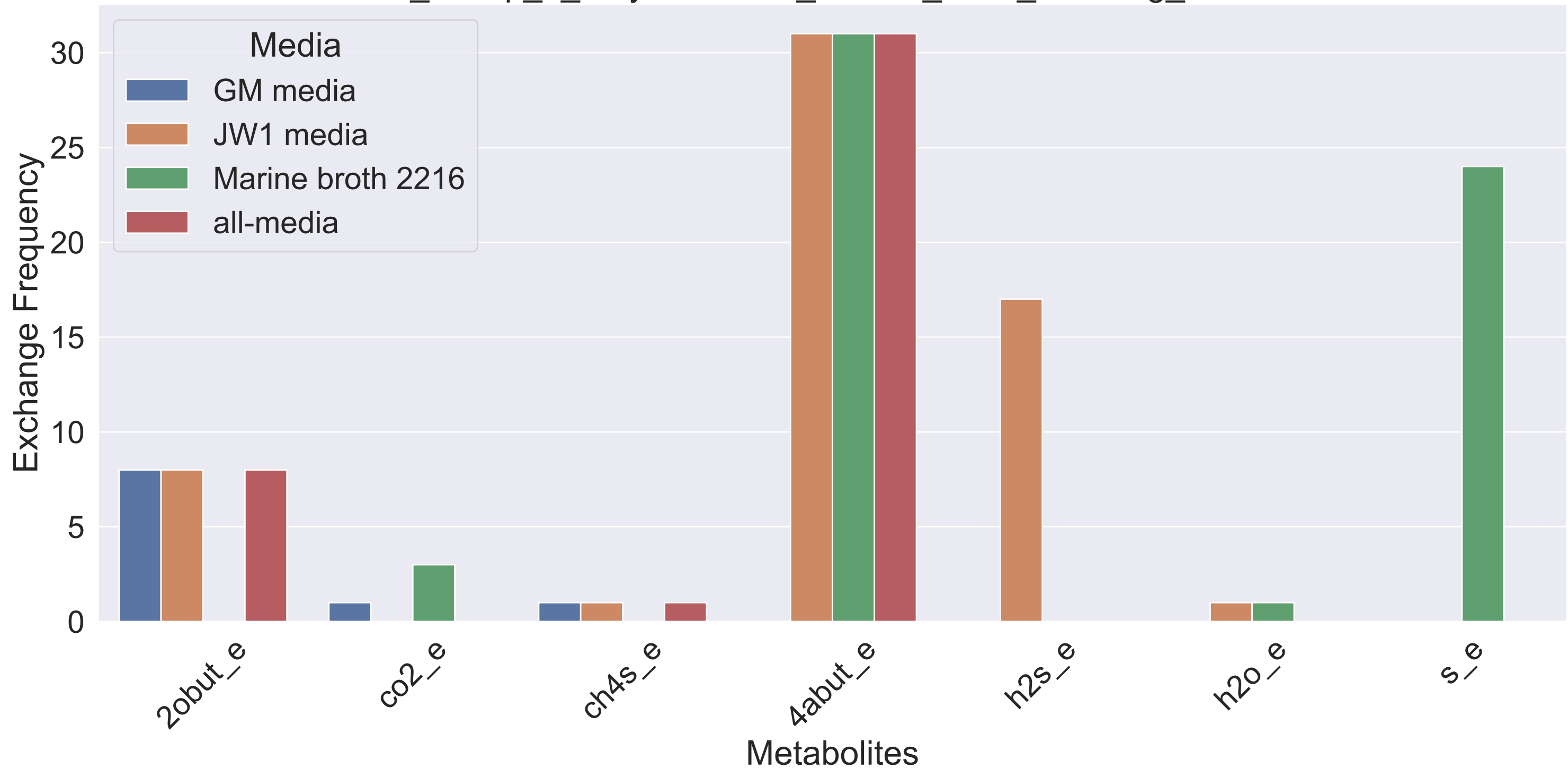

Supplement: Supplementary file 7 — Supplementary File S6 [file 43705_2023_242_MOESM7_ESM.zip › Supplementary Figure S160 Marine Group II euryarchaeote UWMA 0323 donating metabolites.pdf]

Marine\_Group\_II\_euryarchaeote\_UWMA\_0328\_donating\_metabolites

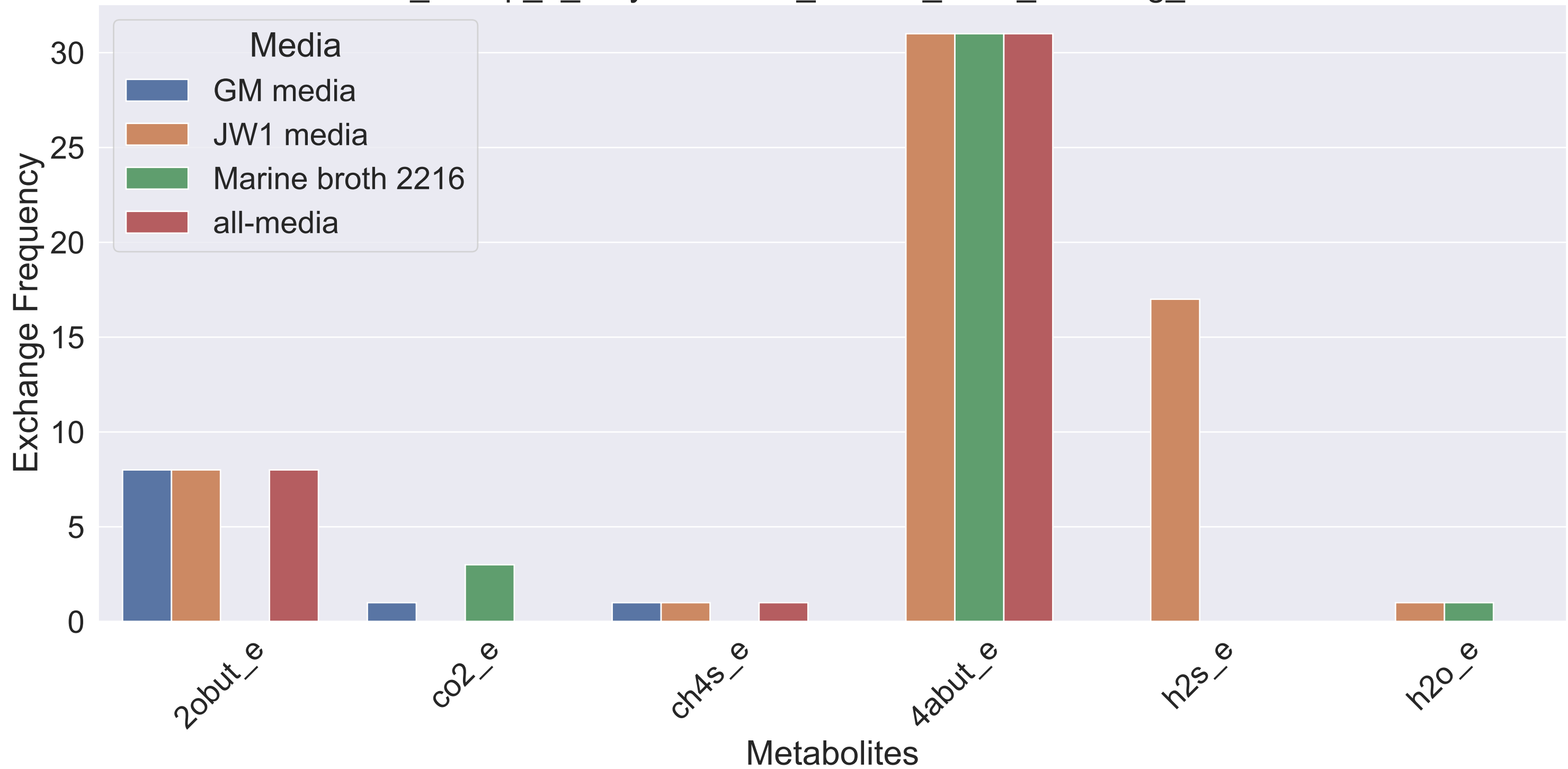

Supplement: Supplementary file 7 — Supplementary File S6 [file 43705_2023_242_MOESM7_ESM.zip › Supplementary Figure S161 Marine Group II euryarchaeote UWMA 0328 donating metabolites.pdf]

# Marine\_Group\_II\_euryarchaeote\_UWMA\_0344\_donating\_metabolites

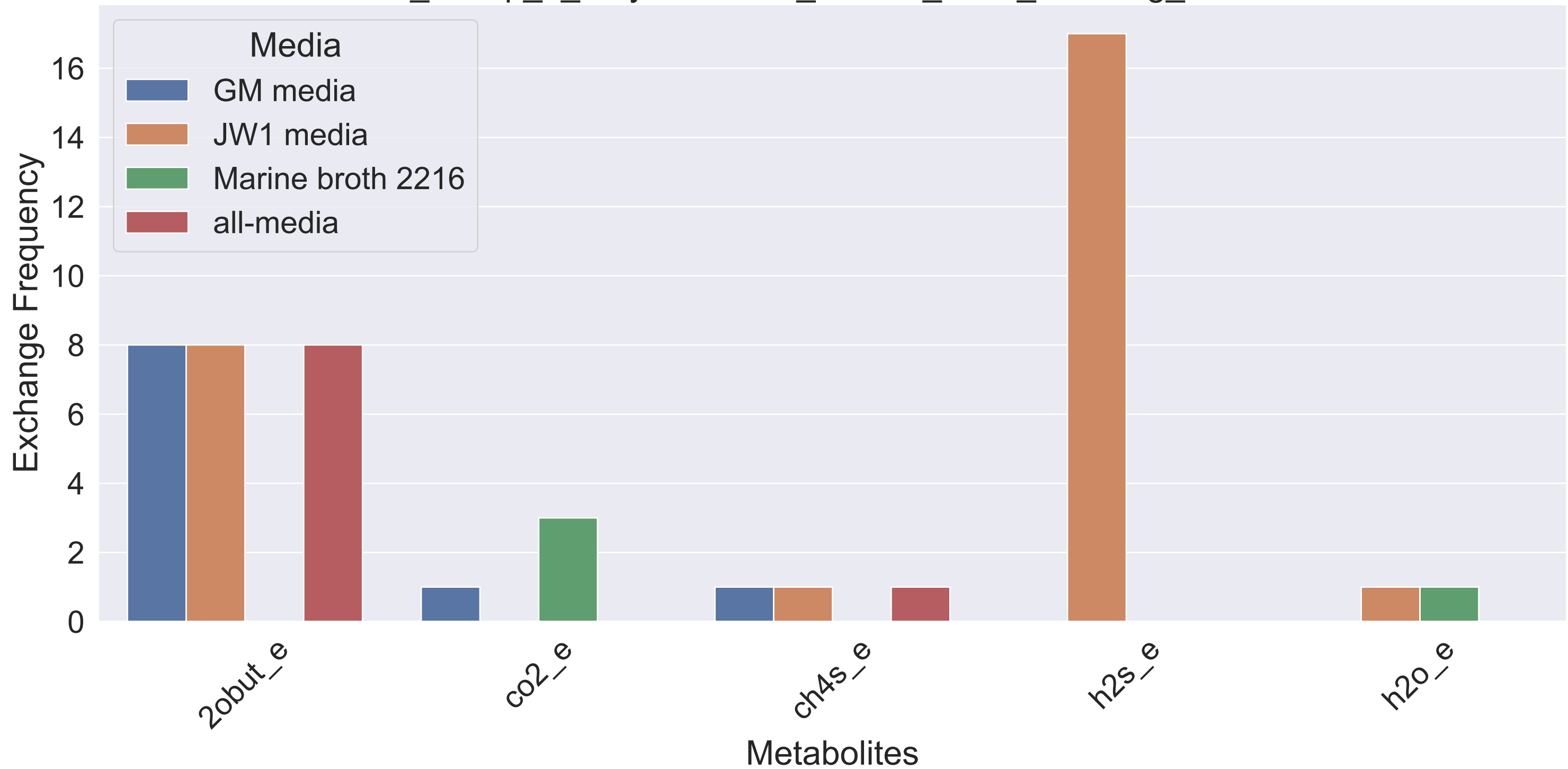

Supplement: Supplementary file 7 — Supplementary File S6 [file 43705_2023_242_MOESM7_ESM.zip › Supplementary Figure S162 Marine Group II euryarchaeote UWMA 0344 donating metabolites.pdf]

# Marine\_Group\_II\_euryarchaeote\_UWMA\_0350\_donating\_metabolites

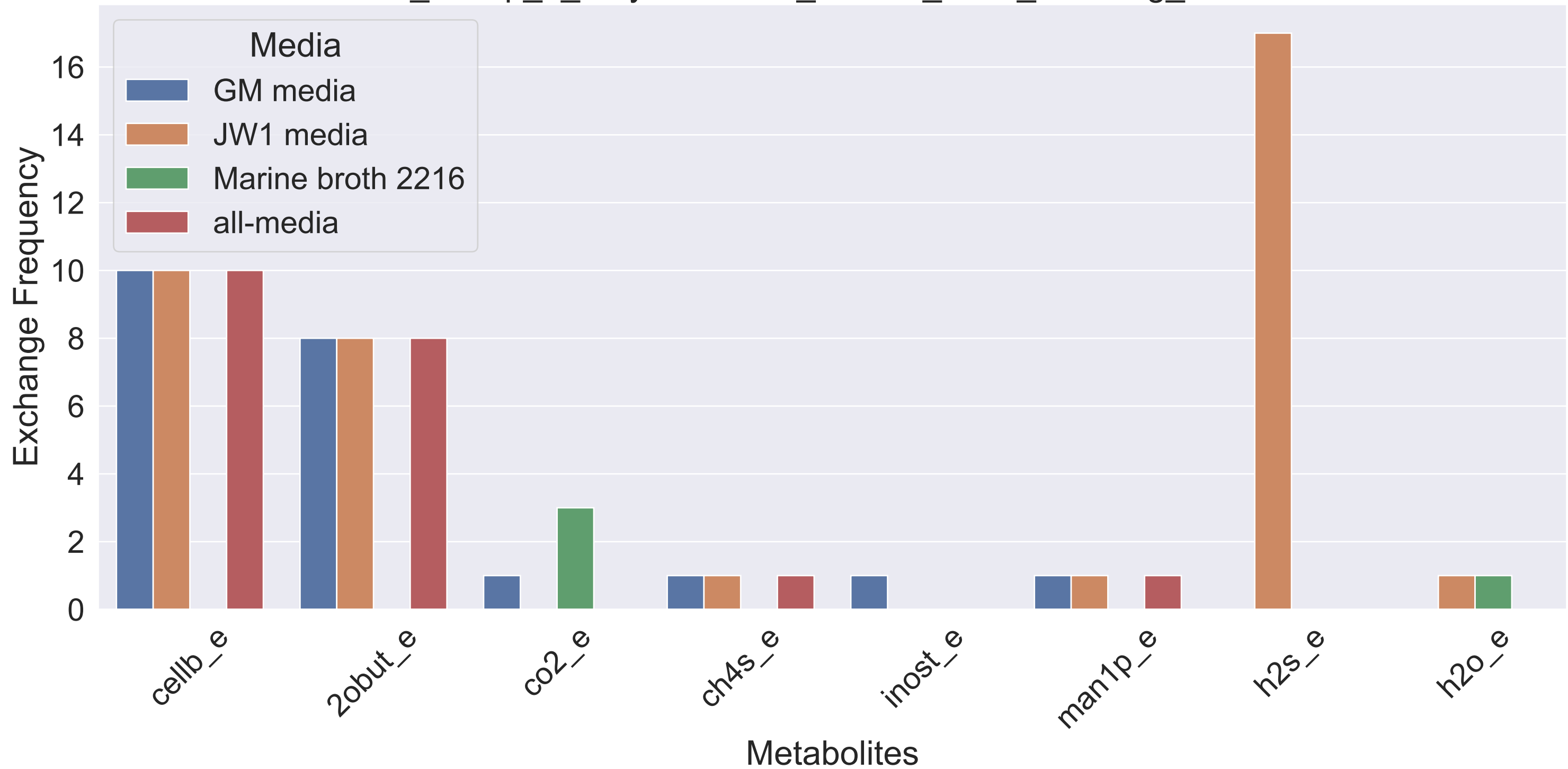

Supplement: Supplementary file 7 — Supplementary File S6 [file 43705_2023_242_MOESM7_ESM.zip › Supplementary Figure S163 Marine Group II euryarchaeote UWMA 0350 donating metabolites.pdf]

Marine\_Group\_II\_euryarchaeote\_UWMA\_0352\_donating\_metabolites

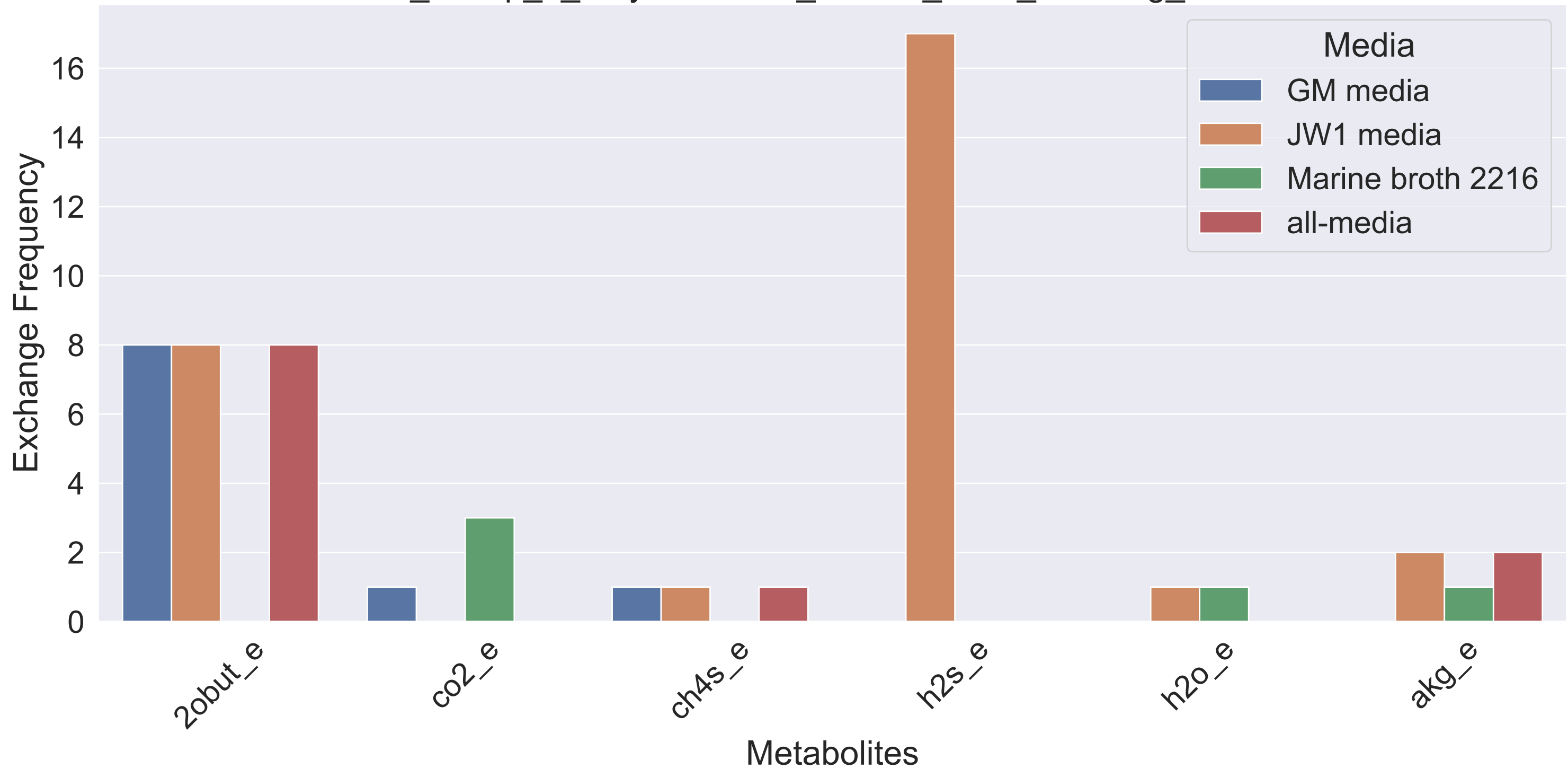

Supplement: Supplementary file 7 — Supplementary File S6 [file 43705_2023_242_MOESM7_ESM.zip › Supplementary Figure S164 Marine Group II euryarchaeote UWMA 0352 donating metabolites.pdf]

# Marine\_Group\_II\_euryarchaeote\_UWMA\_0357\_donating\_metabolites

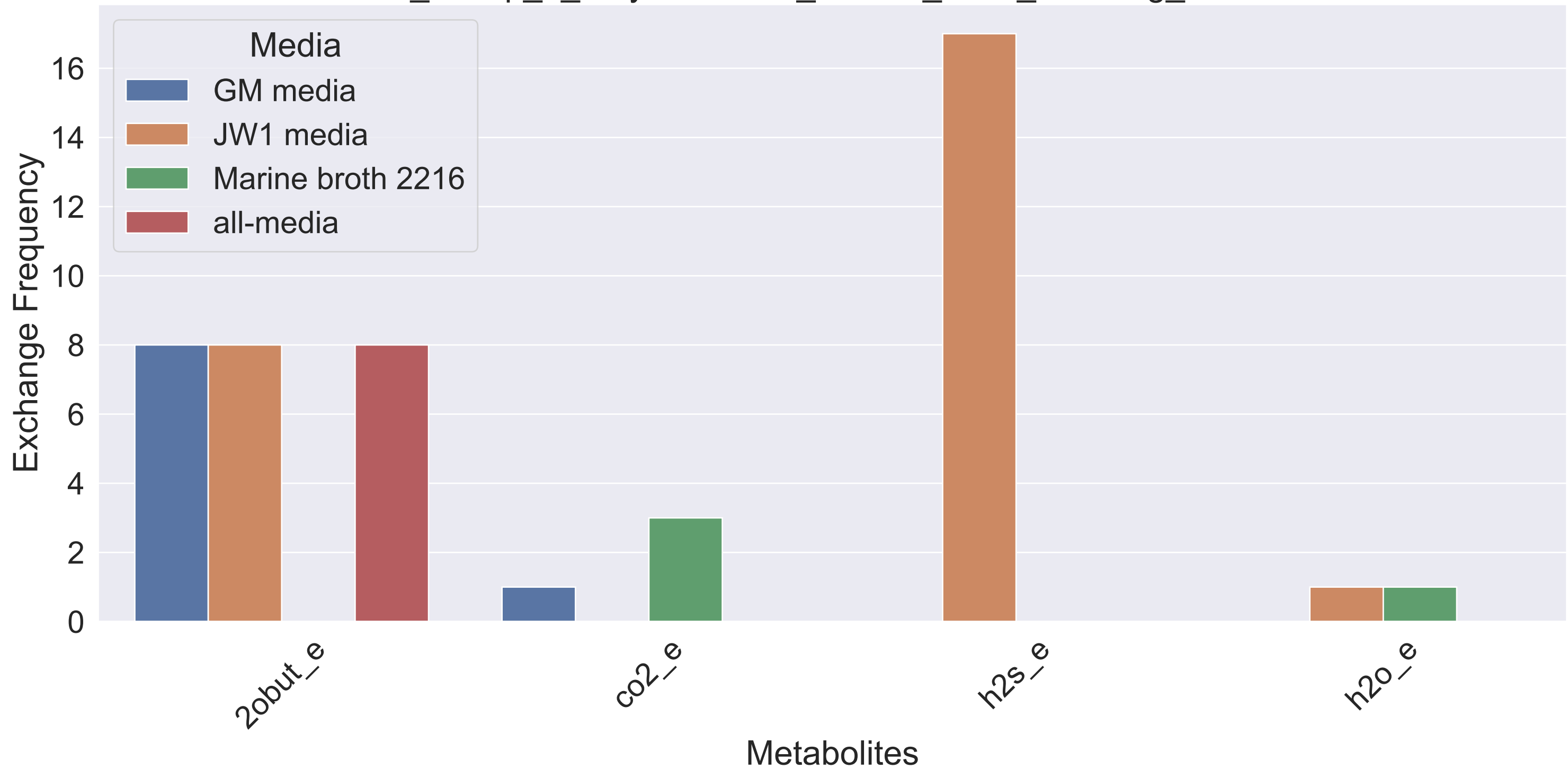

Supplement: Supplementary file 7 — Supplementary File S6 [file 43705_2023_242_MOESM7_ESM.zip › Supplementary Figure S165 Marine Group II euryarchaeote UWMA 0357 donating metabolites.pdf]

# Methylococcaceae\_bacterium\_UWMA\_0325\_donating\_metabolites

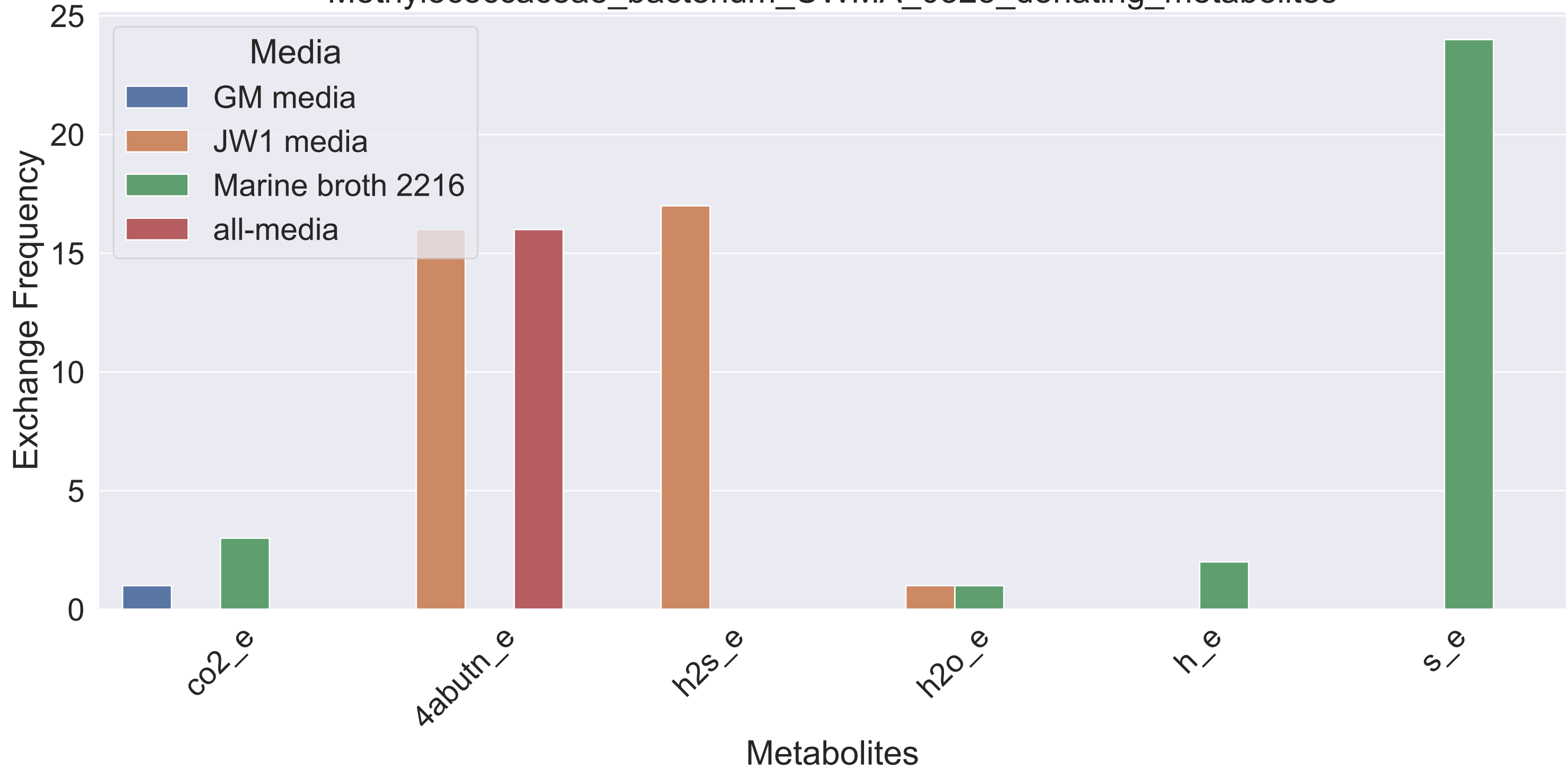

Supplement: Supplementary file 7 — Supplementary File S6 [file 43705_2023_242_MOESM7_ESM.zip › Supplementary Figure S166 Methylococcaceae bacterium UWMA 0325 donating metabolites.pdf]

# Methylococcaceae\_bacterium\_UWMA\_0327\_donating\_metabolites

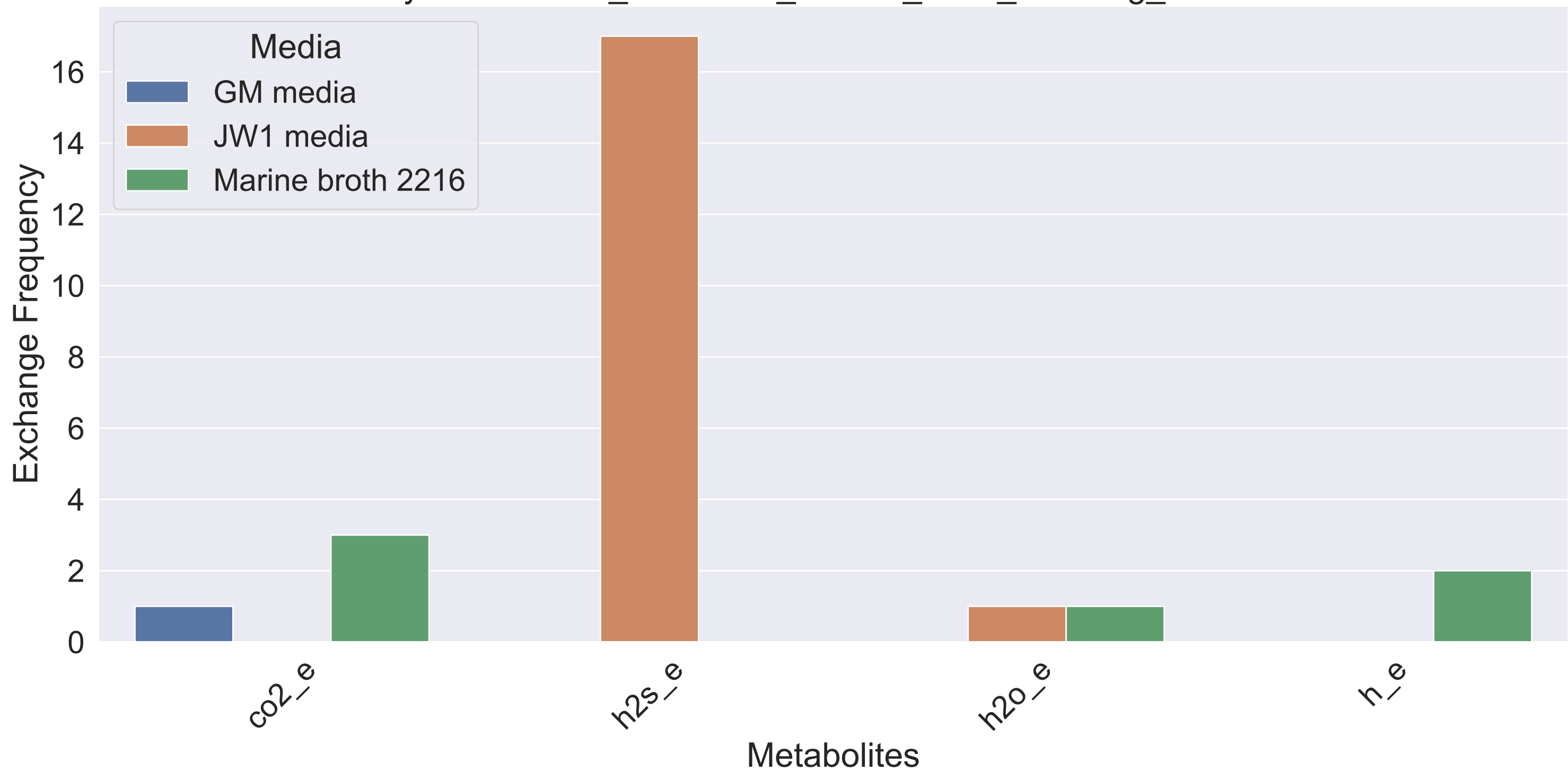

Supplement: Supplementary file 7 — Supplementary File S6 [file 43705_2023_242_MOESM7_ESM.zip › Supplementary Figure S167 Methylococcaceae bacterium UWMA 0327 donating metabolites.pdf]

# Methylococcaceae\_bacterium\_UWMA\_0348\_donating\_metabolites

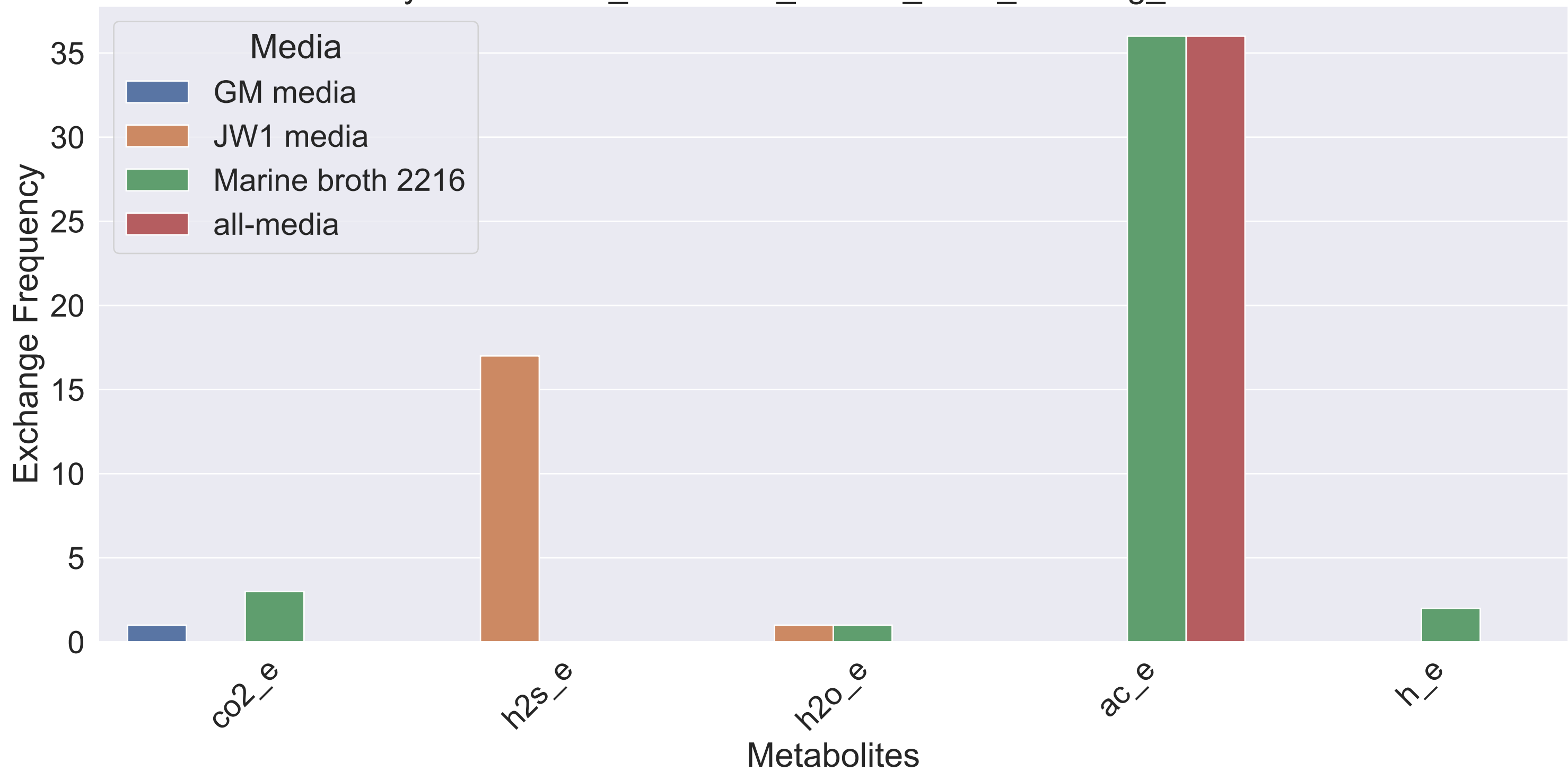

Supplement: Supplementary file 7 — Supplementary File S6 [file 43705_2023_242_MOESM7_ESM.zip › Supplementary Figure S168 Methylococcaceae bacterium UWMA 0348 donating metabolites.pdf]

# Methylophilaceae\_bacterium\_UWMA\_0326\_donating\_metabolites

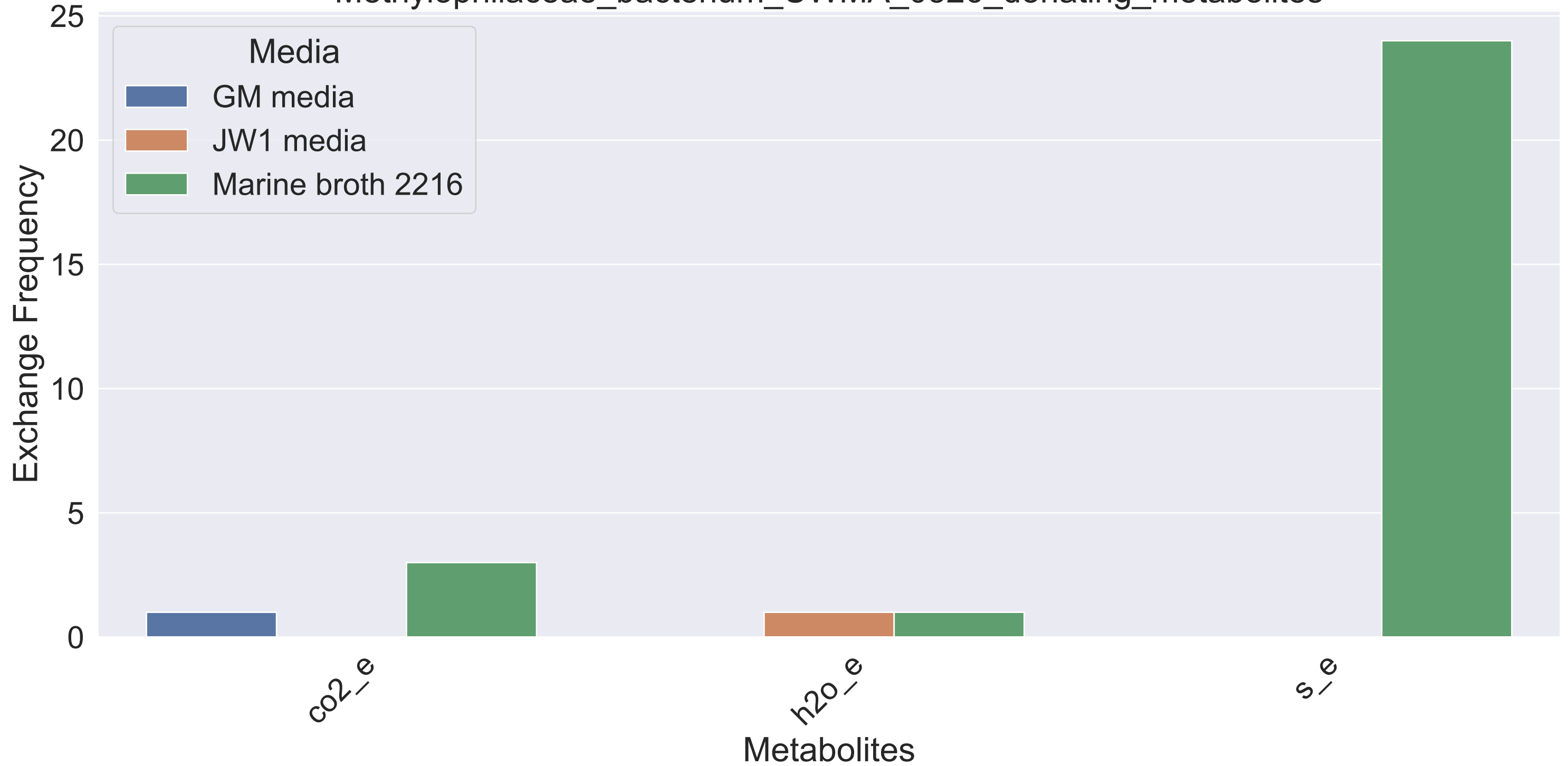

Supplement: Supplementary file 7 — Supplementary File S6 [file 43705_2023_242_MOESM7_ESM.zip › Supplementary Figure S169 Methylophilaceae bacterium UWMA 0326 donating metabolites.pdf]

# Methyloprofundus\_sp\_UWMA\_0312\_donating\_metabolites

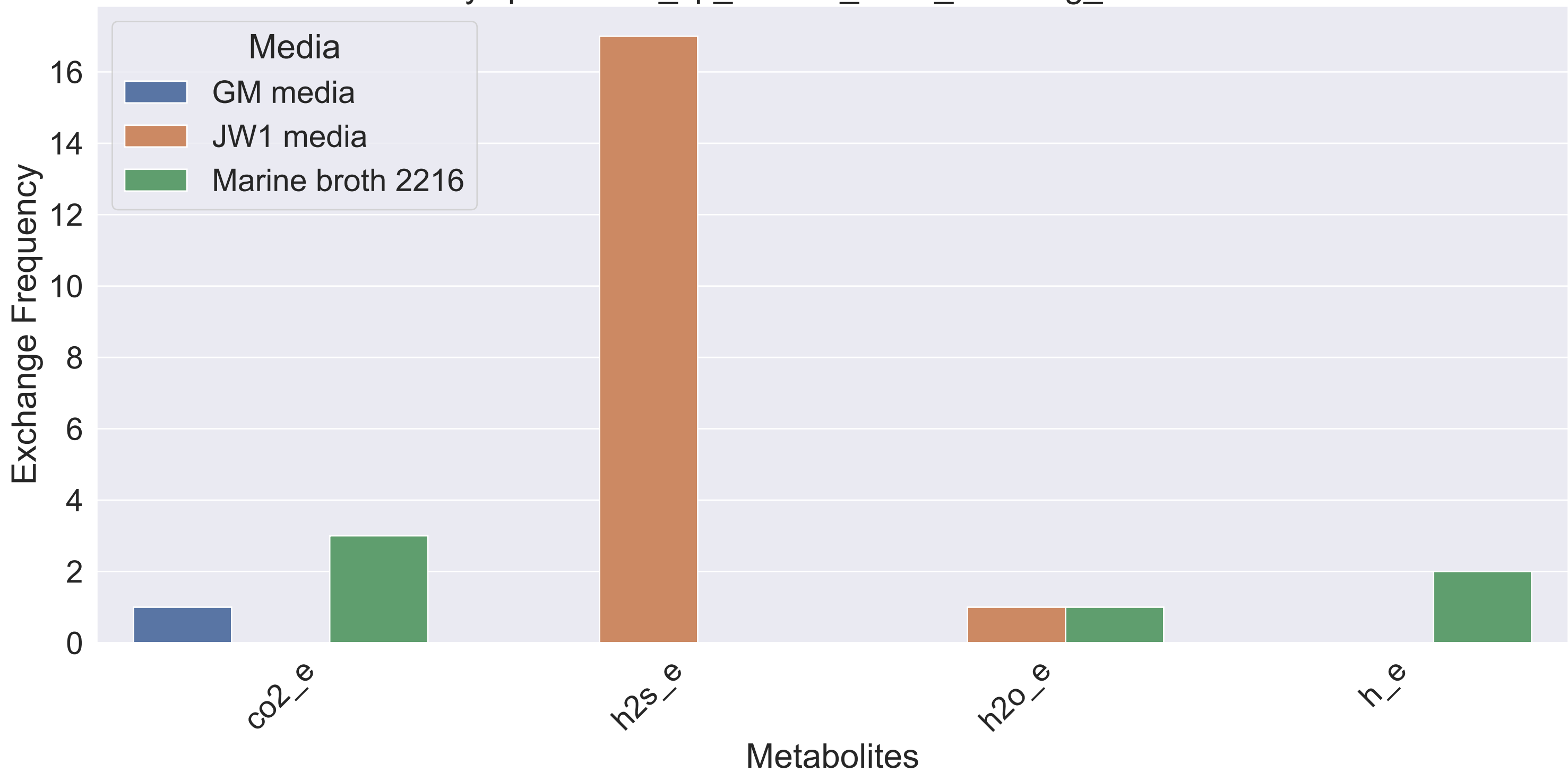

Supplement: Supplementary file 7 — Supplementary File S6 [file 43705_2023_242_MOESM7_ESM.zip › Supplementary Figure S170 Methyloprofundus sp UWMA 0312 donating metabolites.pdf]

Micavibrio\_sp\_UWMA\_0310\_donating\_metabolites

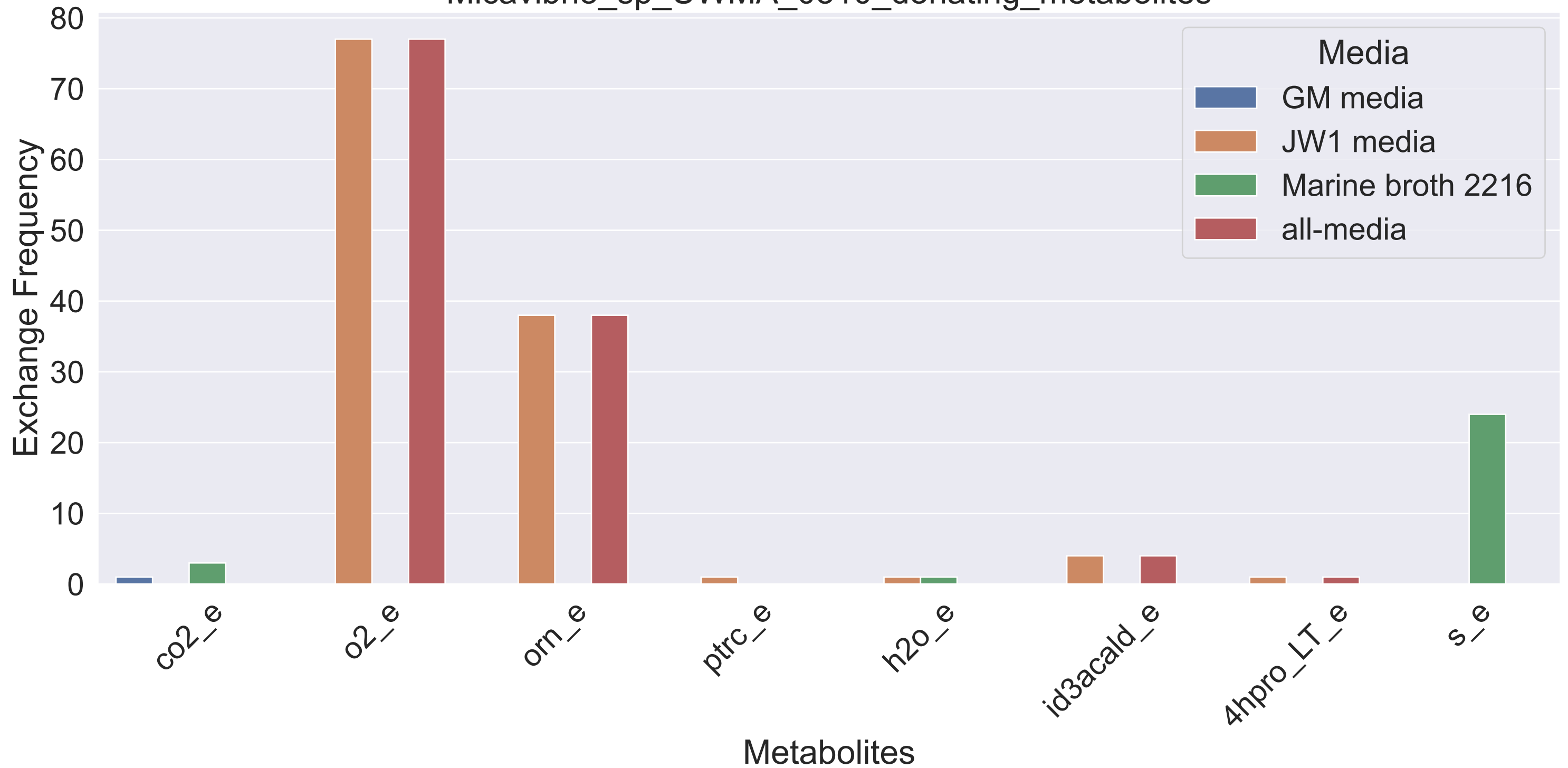

Supplement: Supplementary file 7 — Supplementary File S6 [file 43705_2023_242_MOESM7_ESM.zip › Supplementary Figure S171 Micavibrio sp UWMA 0310 donating metabolites.pdf]

Microbacterium\_sp\_UWMA\_0270\_donating\_metabolites

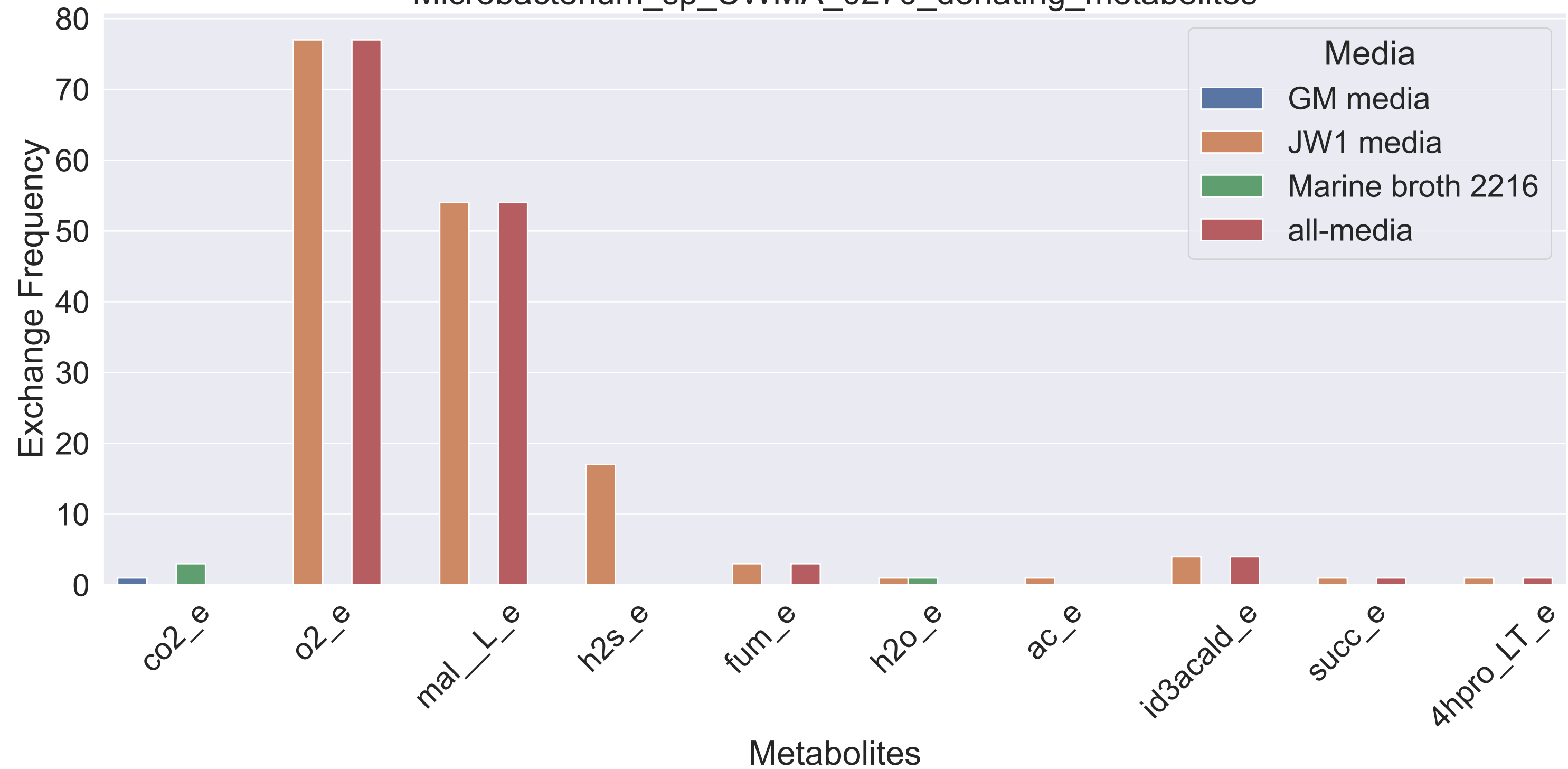

Supplement: Supplementary file 7 — Supplementary File S6 [file 43705_2023_242_MOESM7_ESM.zip › Supplementary Figure S172 Microbacterium sp UWMA 0270 donating metabolites.pdf]

# Myxococcales\_bacterium\_UWMA\_0315\_donating\_metabolites

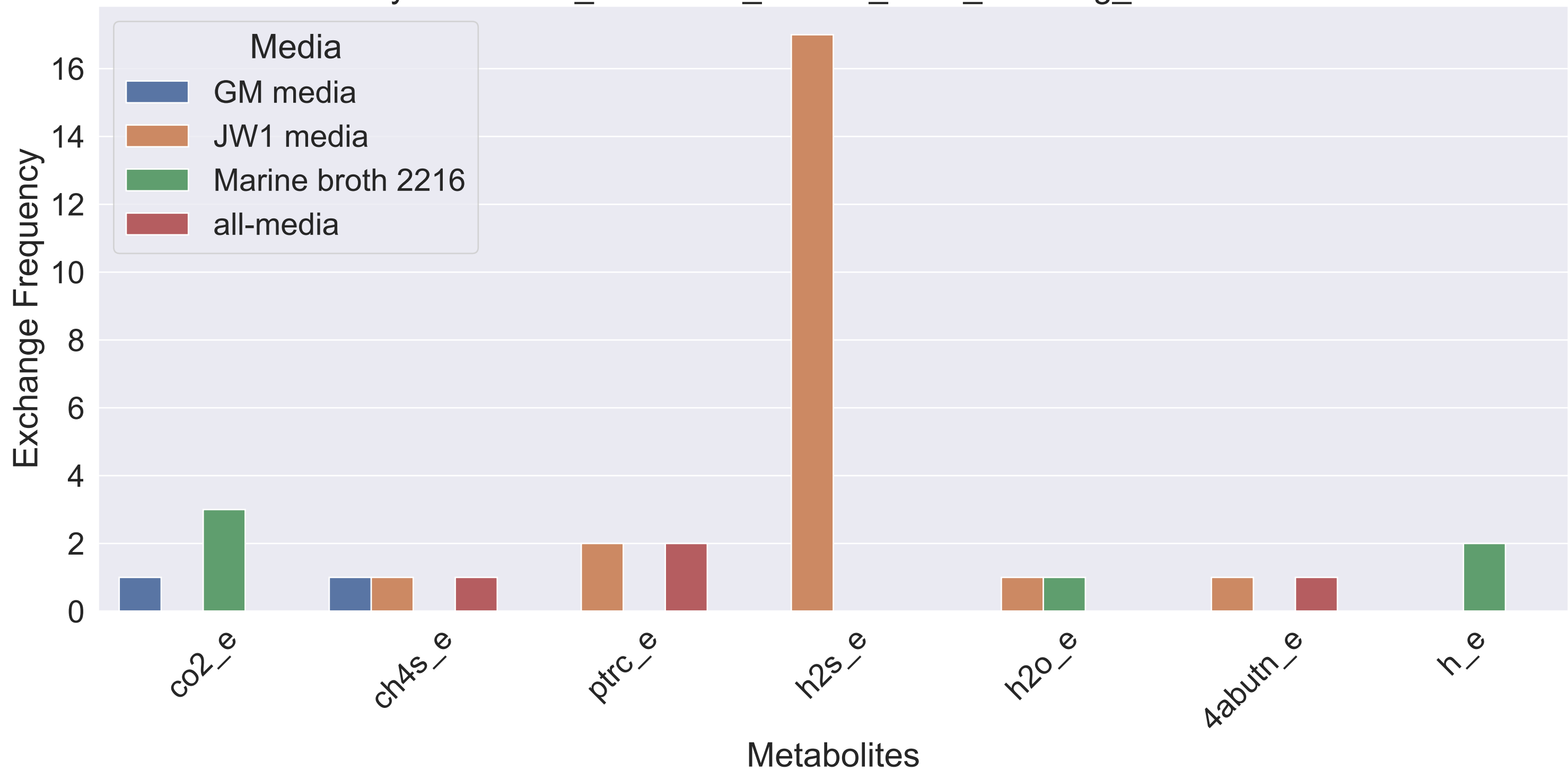

Supplement: Supplementary file 7 — Supplementary File S6 [file 43705_2023_242_MOESM7_ESM.zip › Supplementary Figure S173 Myxococcales bacterium UWMA 0315 donating metabolites.pdf]

# Myxococcales\_bacterium\_UWMA\_0324\_donating\_metabolites

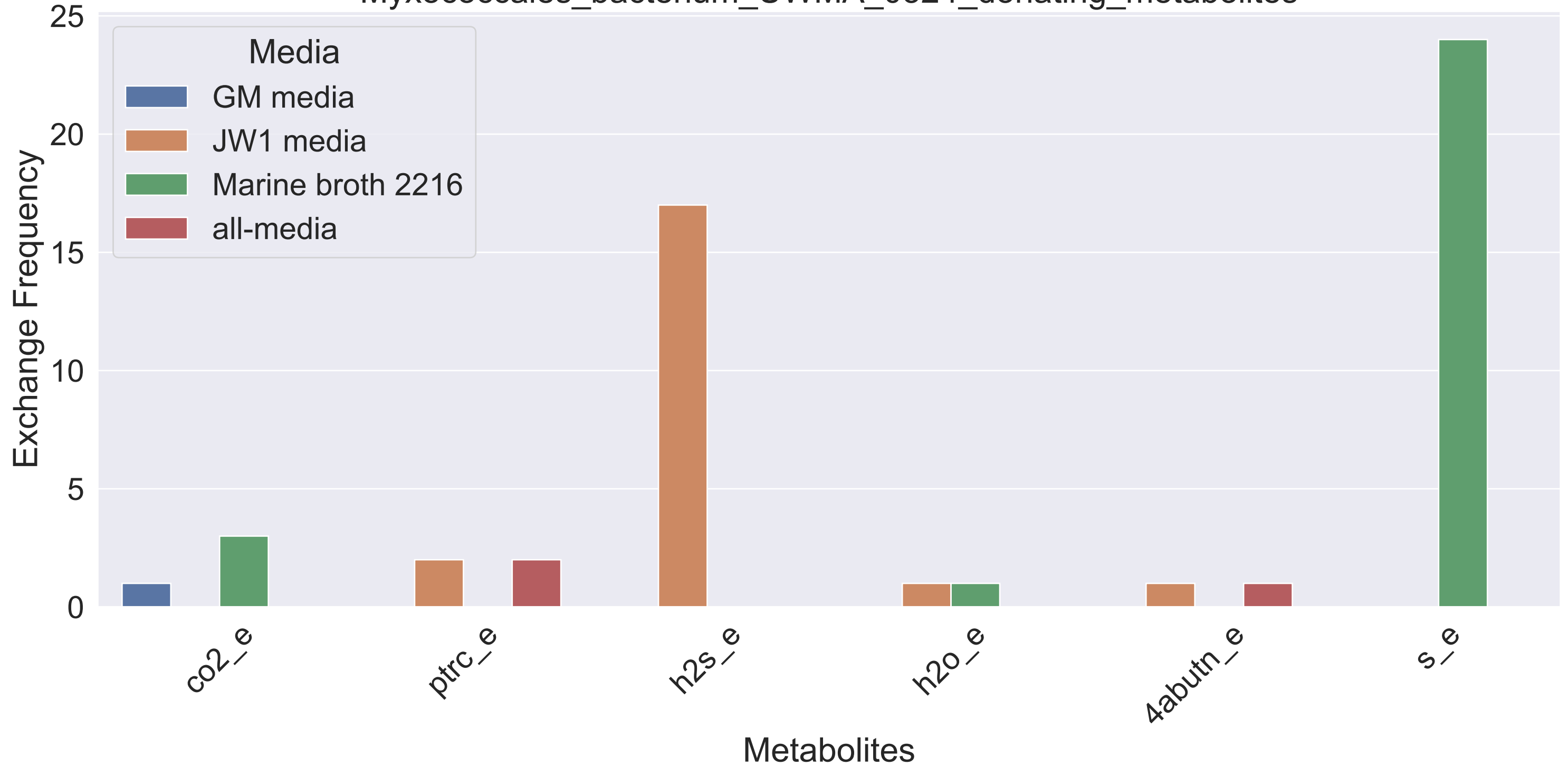

Supplement: Supplementary file 7 — Supplementary File S6 [file 43705_2023_242_MOESM7_ESM.zip › Supplementary Figure S174 Myxococcales bacterium UWMA 0324 donating metabolites.pdf]

# Myxococcales\_bacterium\_UWMA\_0330\_donating\_metabolites

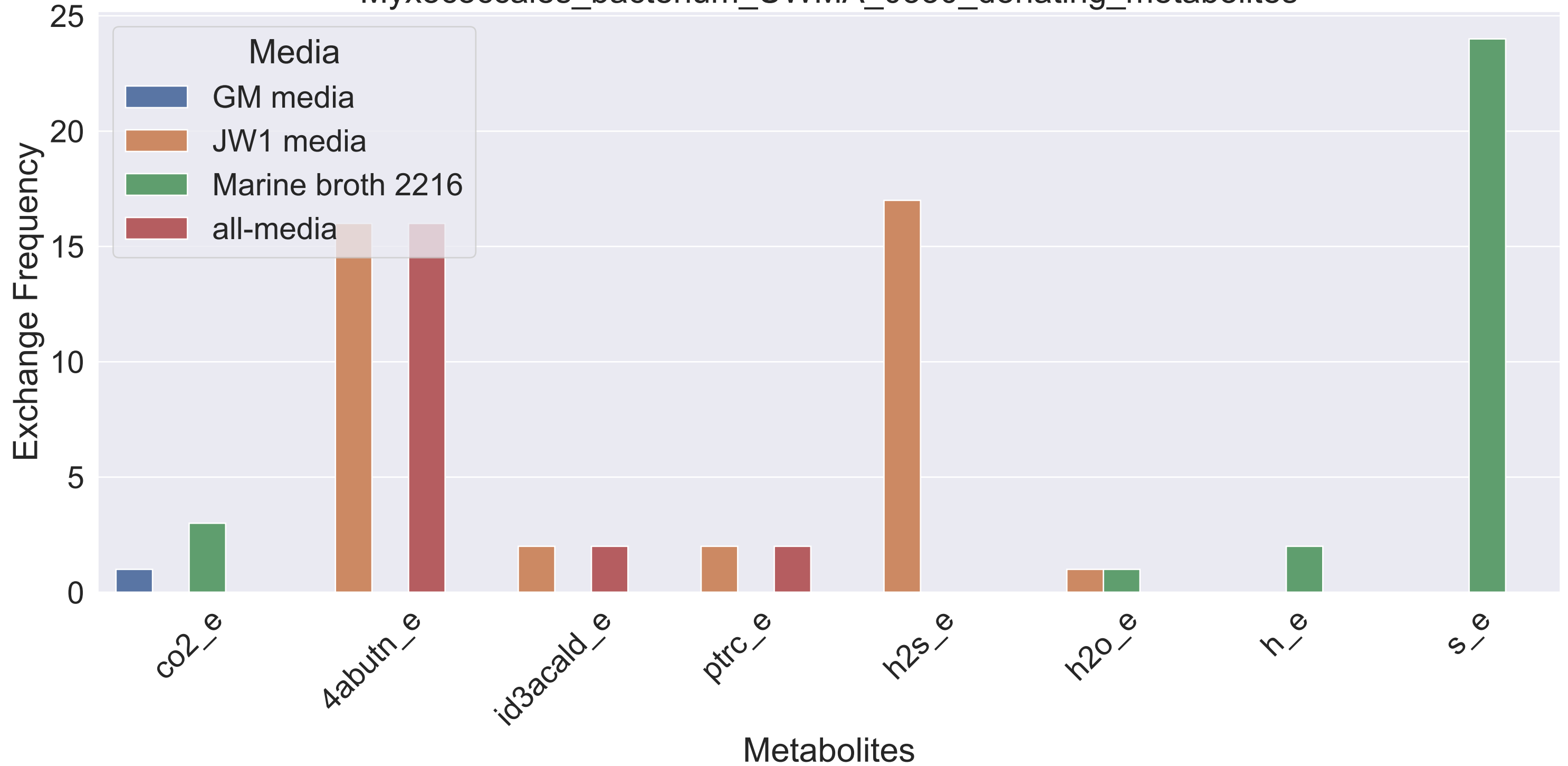

Supplement: Supplementary file 7 — Supplementary File S6 [file 43705_2023_242_MOESM7_ESM.zip › Supplementary Figure S175 Myxococcales bacterium UWMA 0330 donating metabolites.pdf]

# Myxococcales\_bacterium\_UWMA\_0336\_donating\_metabolites

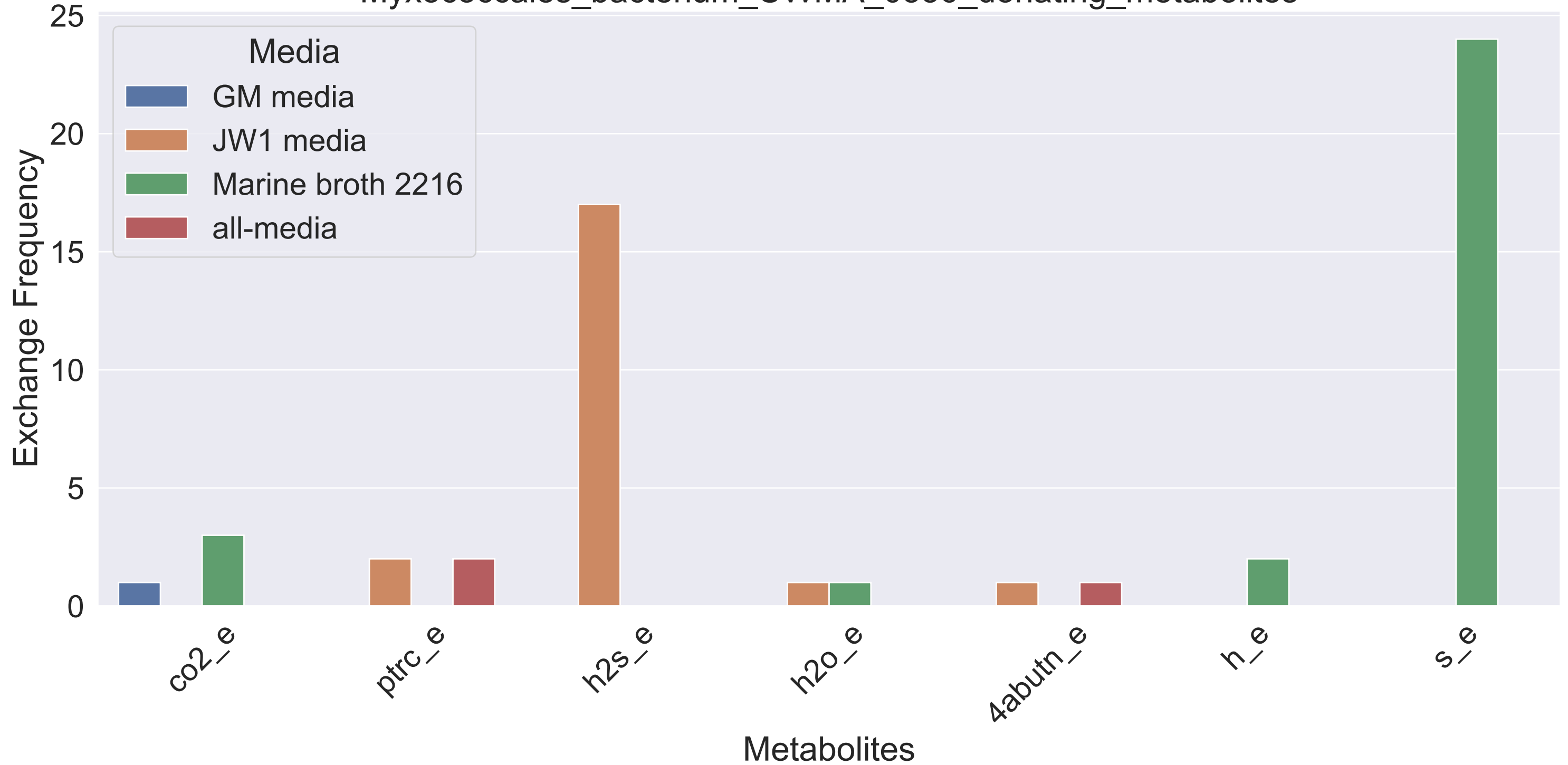

Supplement: Supplementary file 7 — Supplementary File S6 [file 43705_2023_242_MOESM7_ESM.zip › Supplementary Figure S176 Myxococcales bacterium UWMA 0336 donating metabolites.pdf]

# Nitrosopumilus\_sp\_UWMA\_0263\_donating\_metabolites

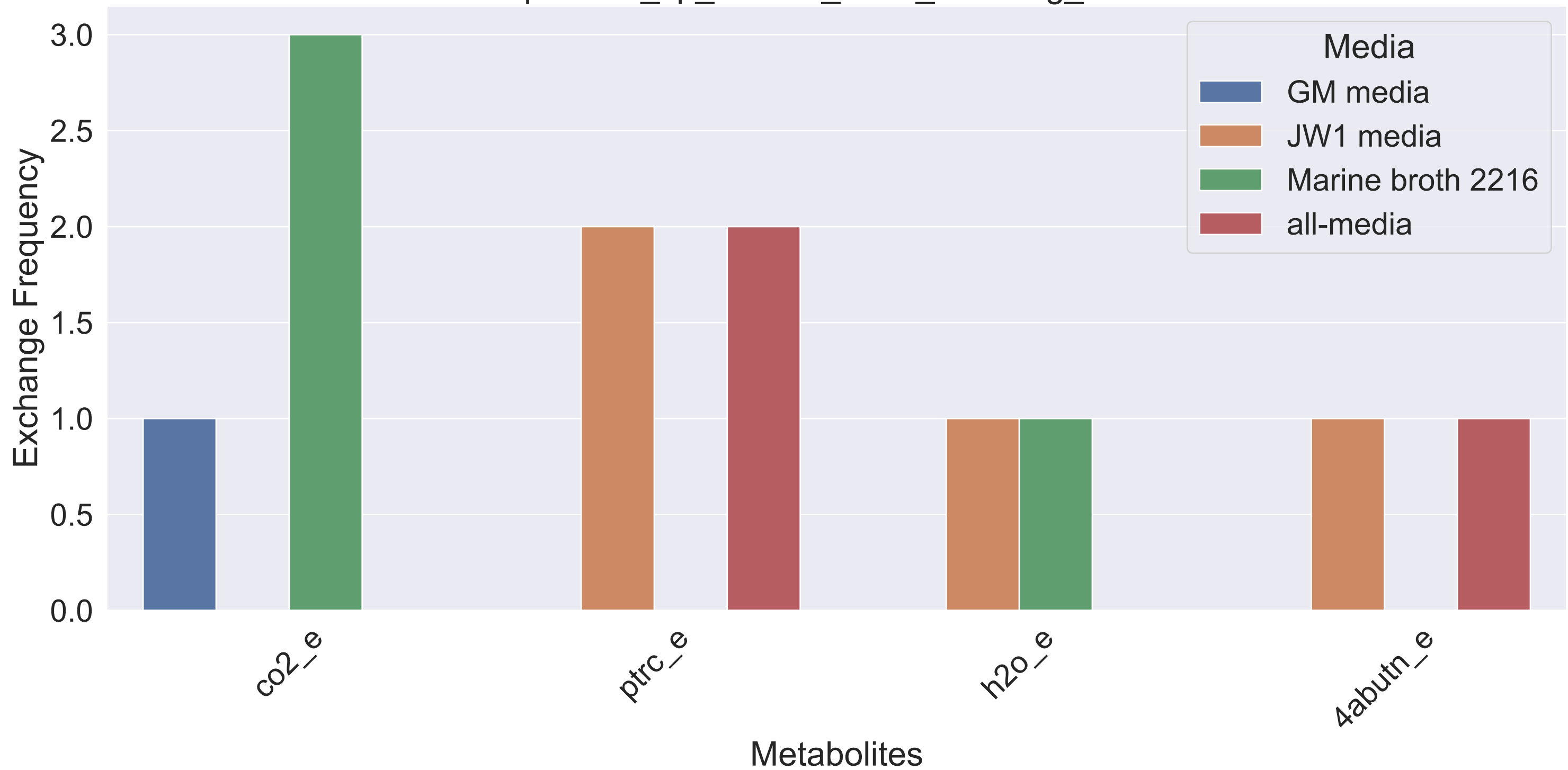

Supplement: Supplementary file 7 — Supplementary File S6 [file 43705_2023_242_MOESM7_ESM.zip › Supplementary Figure S177 Nitrosopumilus sp UWMA 0263 donating metabolites.pdf]

Nitrospinaeaceae\_bacterium\_UWMA\_0262\_donating\_metabolites

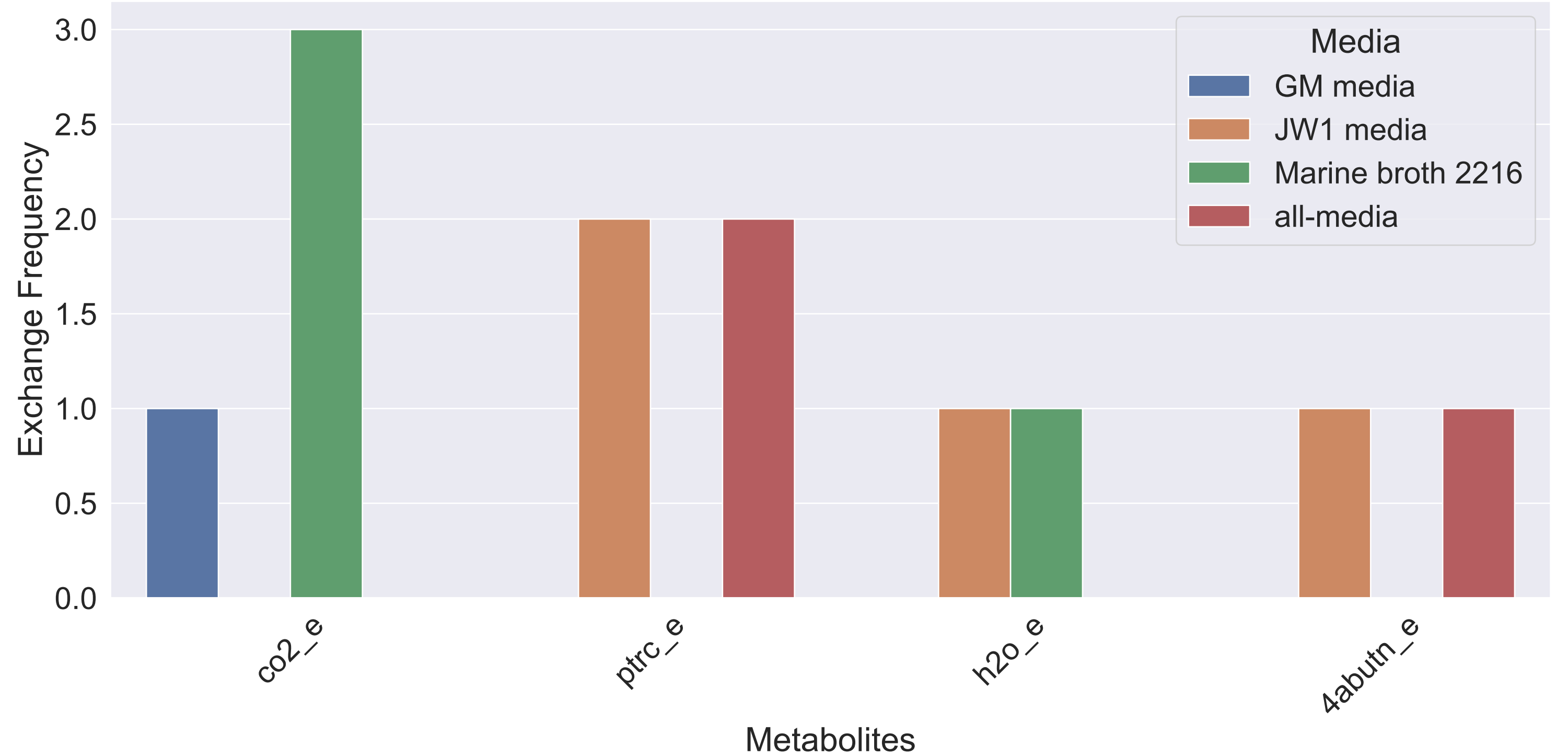

Supplement: Supplementary file 7 — Supplementary File S6 [file 43705_2023_242_MOESM7_ESM.zip › Supplementary Figure S178 Nitrospinaceae bacterium UWMA 0262 donating metabolites.pdf]

Nitrospinaeaceae\_bacterium\_UWMA\_0271\_donating\_metabolites

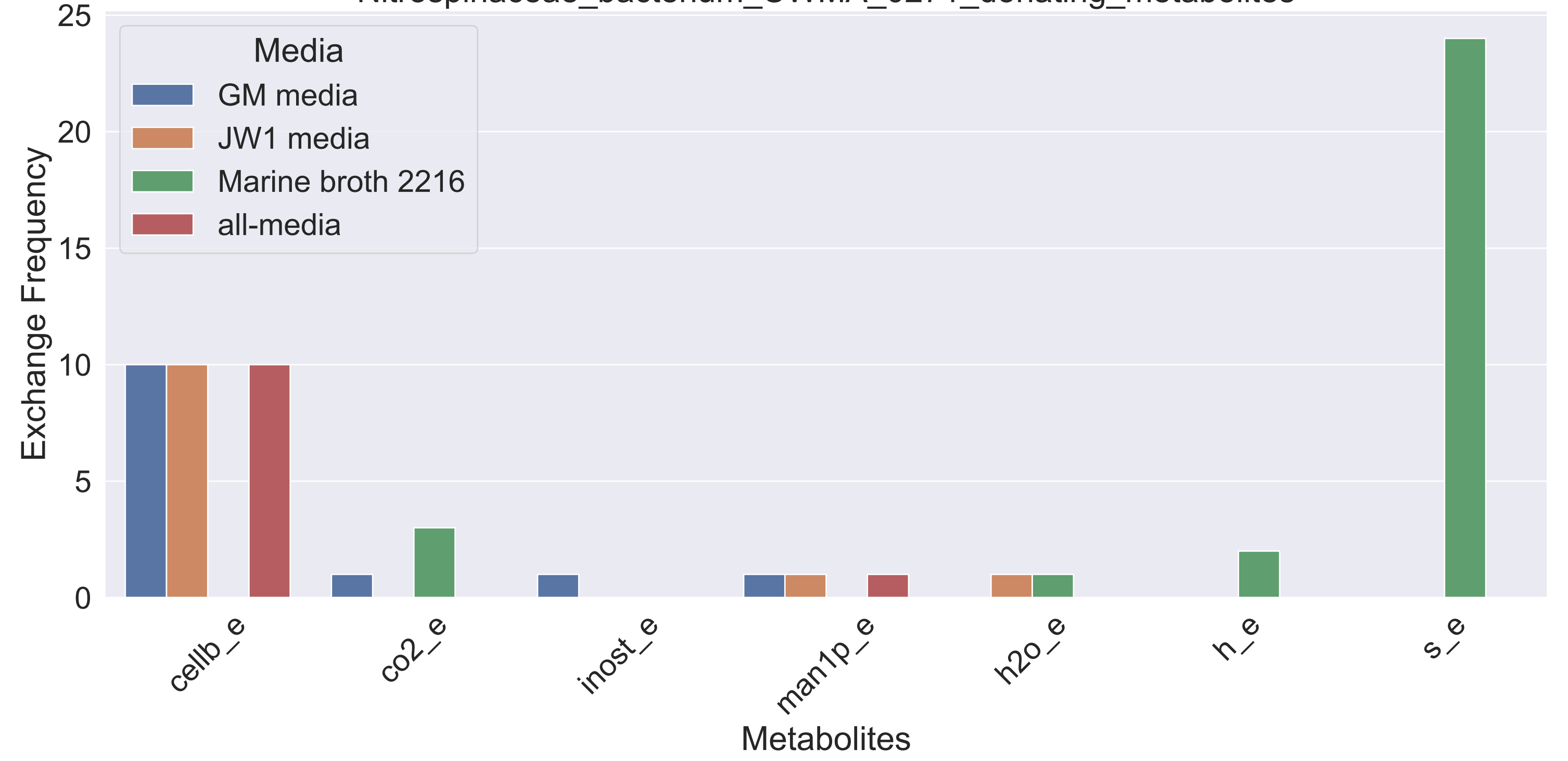

Supplement: Supplementary file 7 — Supplementary File S6 [file 43705_2023_242_MOESM7_ESM.zip › Supplementary Figure S179 Nitrospinaceae bacterium UWMA 0271 donating metabolites.pdf]

Nitrospinaeae\_bacterium\_UWMA\_0356\_donating\_metabolites

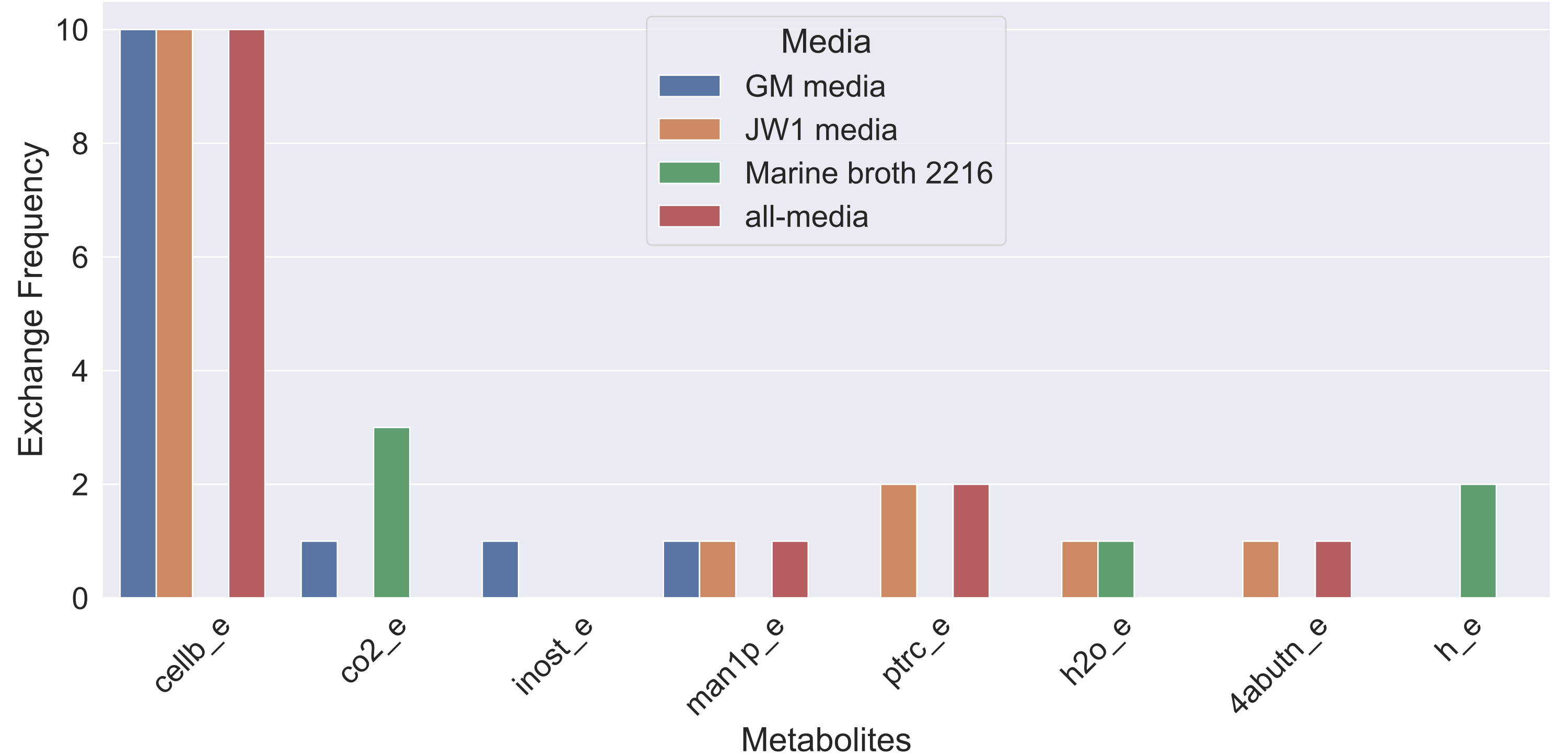

Supplement: Supplementary file 7 — Supplementary File S6 [file 43705_2023_242_MOESM7_ESM.zip › Supplementary Figure S180 Nitrospinaceae bacterium UWMA 0356 donating metabolites.pdf]

Nitrospira\_sp\_UWMA\_0268\_donating\_metabolites

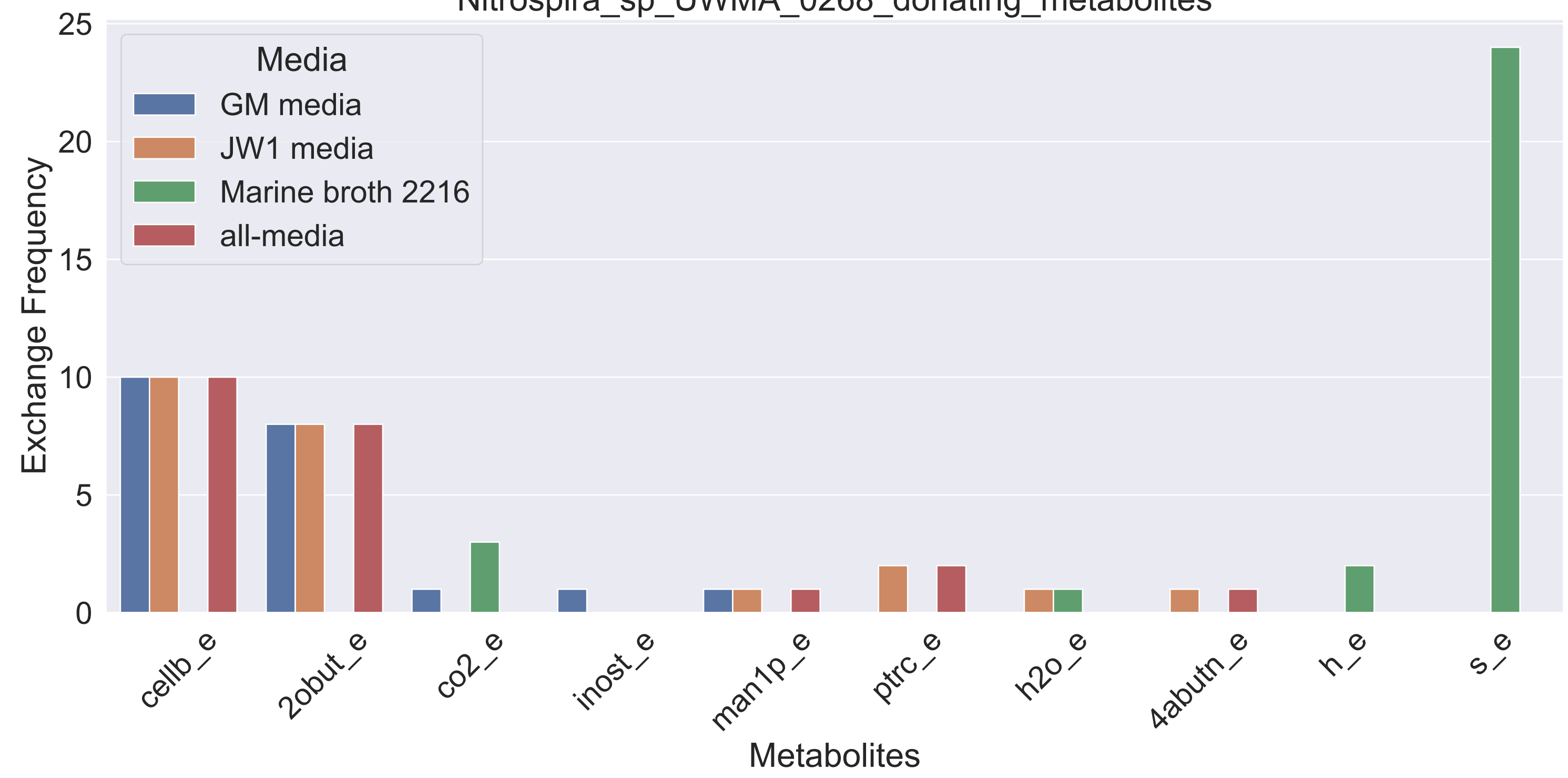

Supplement: Supplementary file 7 — Supplementary File S6 [file 43705_2023_242_MOESM7_ESM.zip › Supplementary Figure S181 Nitrospira sp UWMA 0268 donating metabolites.pdf]

# Oceanospirillaceae\_bacterium\_UWMA\_0280\_donating\_metabolites

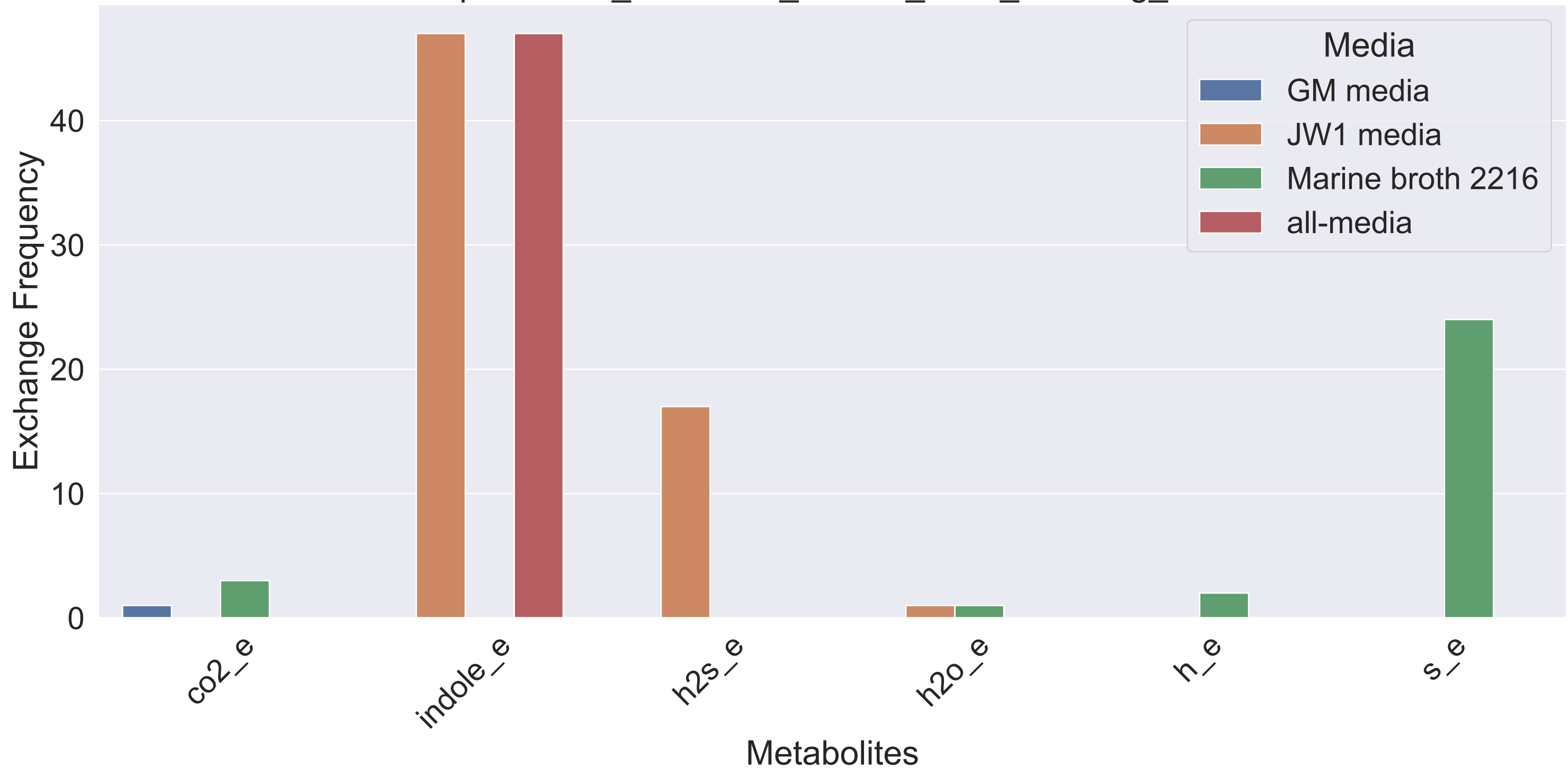

Supplement: Supplementary file 7 — Supplementary File S6 [file 43705_2023_242_MOESM7_ESM.zip › Supplementary Figure S182 Oceanospirillaceae bacterium UWMA 0280 donating metabolites.pdf]

# Planctomycetaceae\_bacterium\_UWMA\_0346\_donating\_metabolites

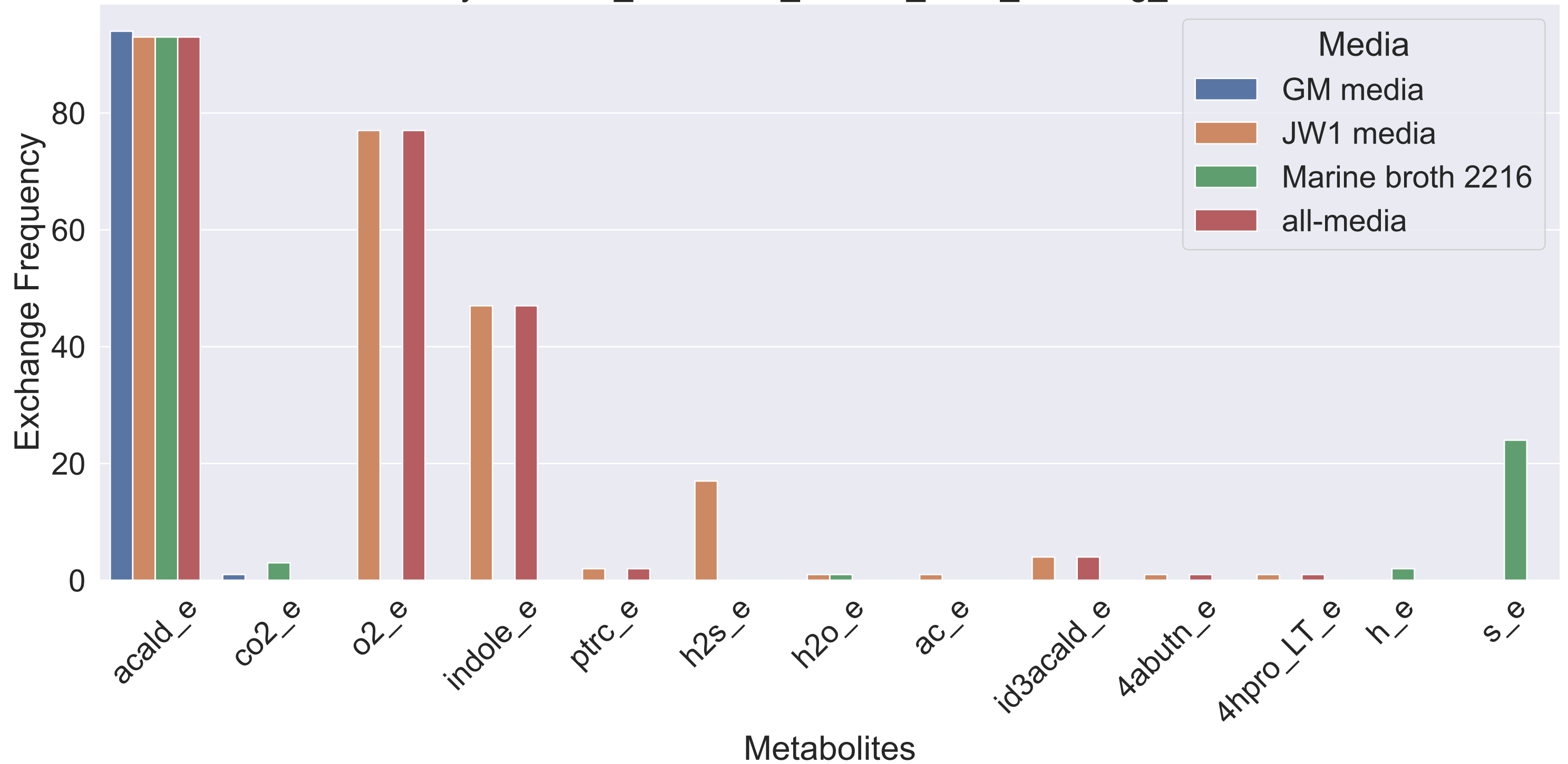

Supplement: Supplementary file 7 — Supplementary File S6 [file 43705_2023_242_MOESM7_ESM.zip › Supplementary Figure S183 Planctomycetaceae bacterium UWMA 0346 donating metabolites.pdf]

# Planctomycetes\_bacterium\_UWMA\_0265\_donating\_metabolites

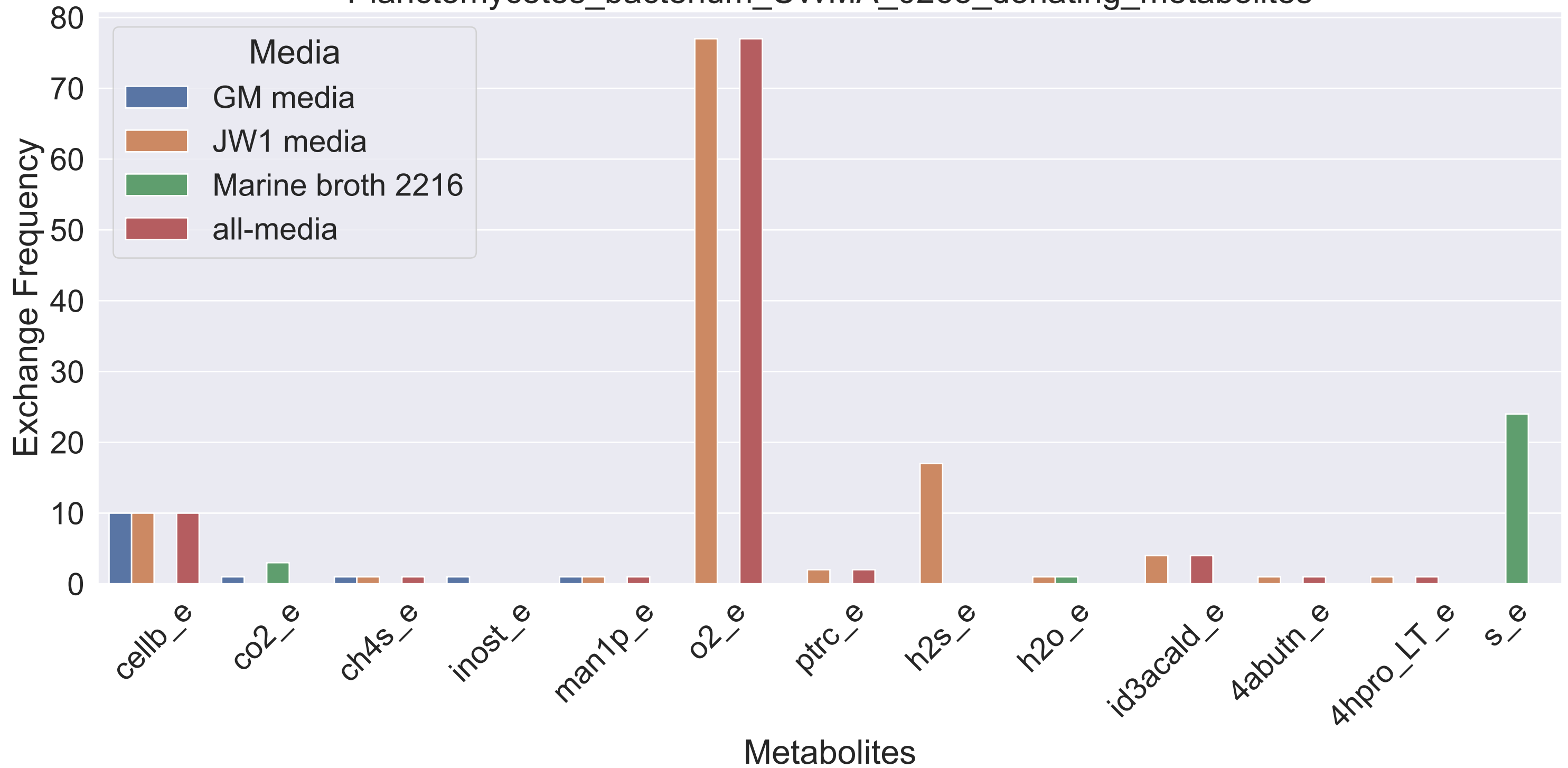

Supplement: Supplementary file 7 — Supplementary File S6 [file 43705_2023_242_MOESM7_ESM.zip › Supplementary Figure S184 Planctomycetes bacterium UWMA 0265 donating metabolites.pdf]

# Planctomycetes\_bacterium\_UWMA\_0276\_donating\_metabolites

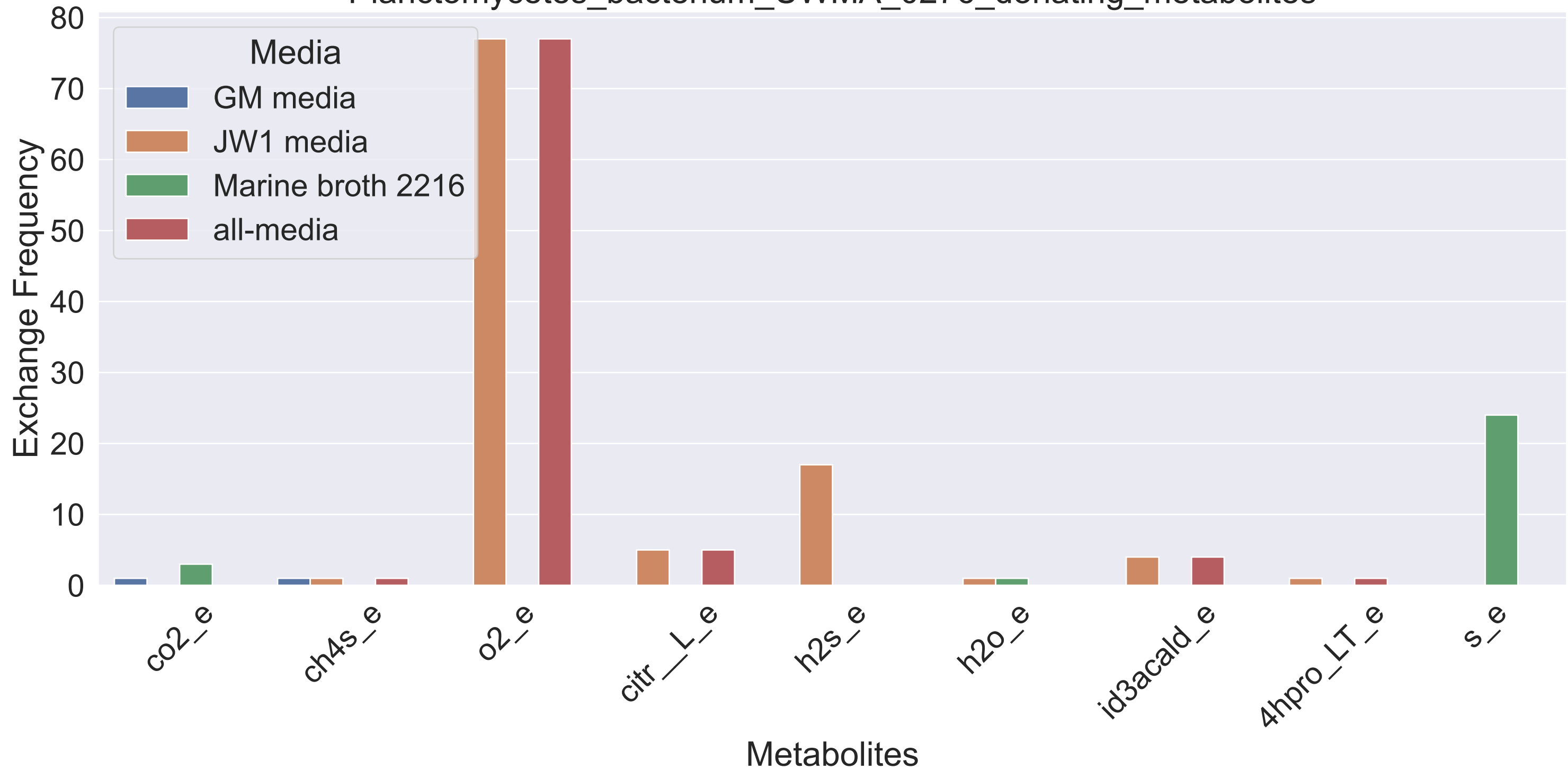

Supplement: Supplementary file 7 — Supplementary File S6 [file 43705_2023_242_MOESM7_ESM.zip › Supplementary Figure S185 Planctomycetes bacterium UWMA 0276 donating metabolites.pdf]

Planctomycetes\_bacterium\_UWMA\_0294\_donating\_metabolites

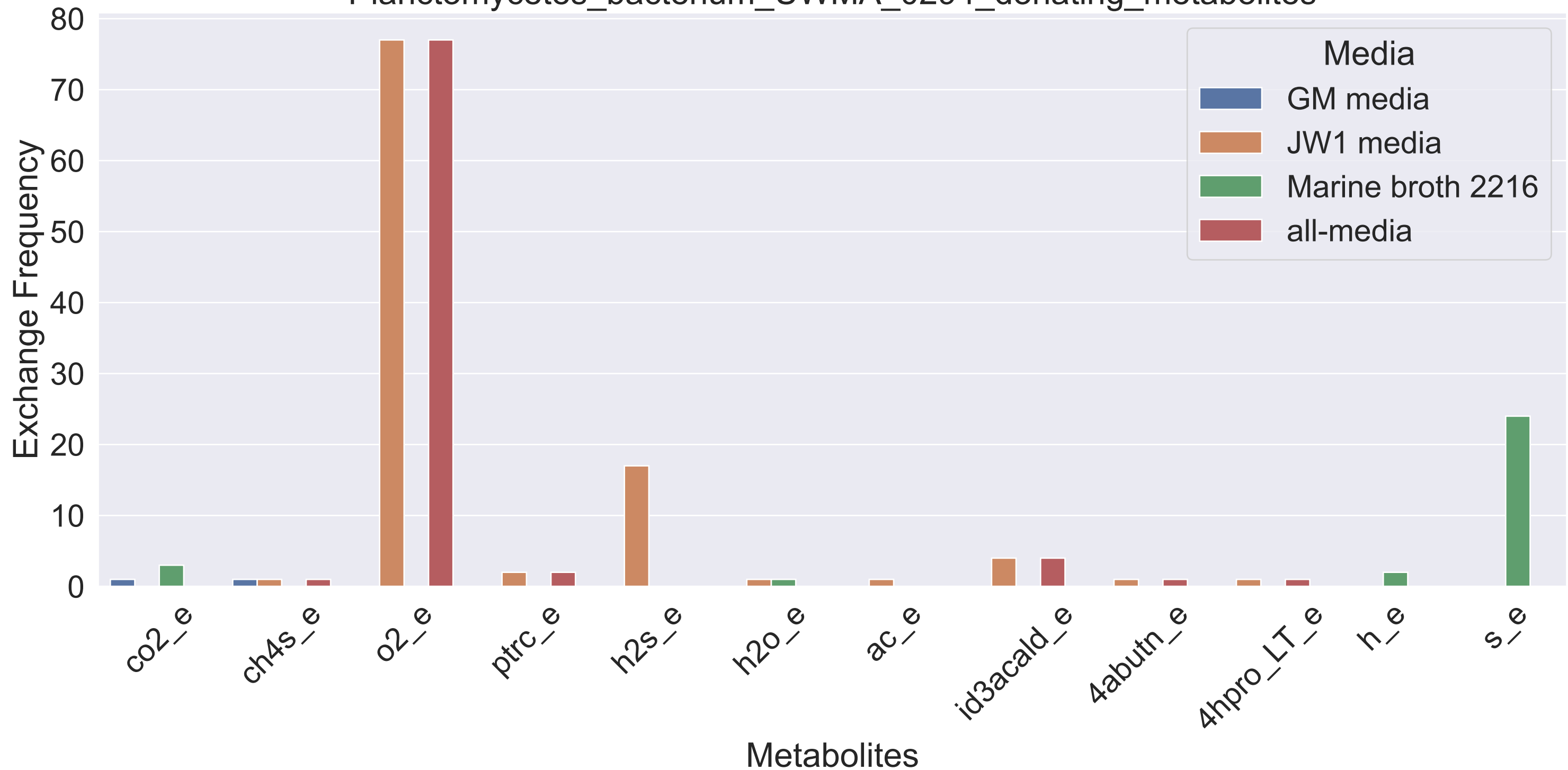

Supplement: Supplementary file 7 — Supplementary File S6 [file 43705_2023_242_MOESM7_ESM.zip › Supplementary Figure S186 Planctomycetes bacterium UWMA 0294 donating metabolites.pdf]

# Planctomycetes\_bacterium\_UWMA\_0329\_donating\_metabolites

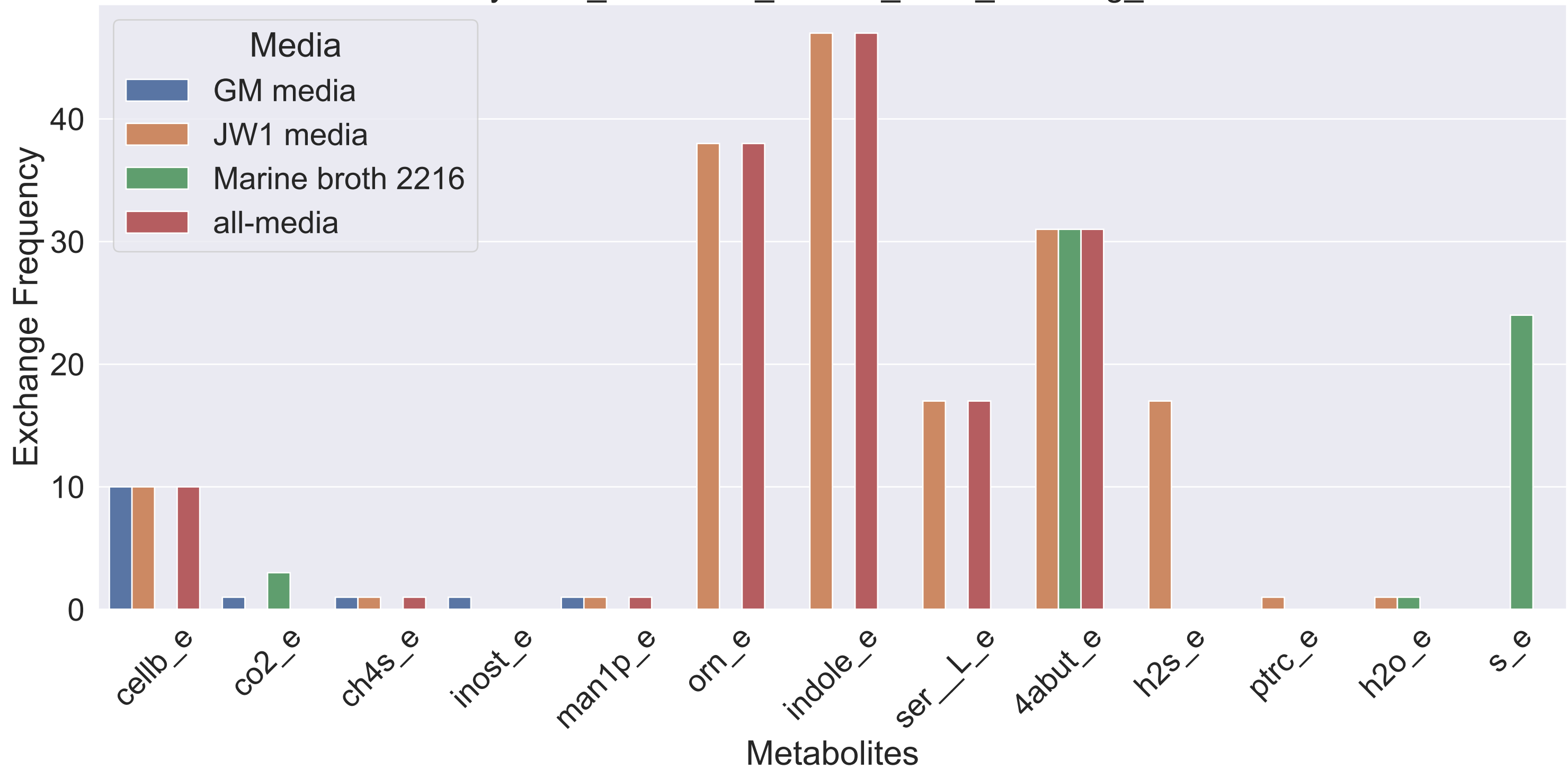

Supplement: Supplementary file 7 — Supplementary File S6 [file 43705_2023_242_MOESM7_ESM.zip › Supplementary Figure S187 Planctomycetes bacterium UWMA 0329 donating metabolites.pdf]

Planctomycetes\_bacterium\_UWMA\_0345\_donating\_metabolites

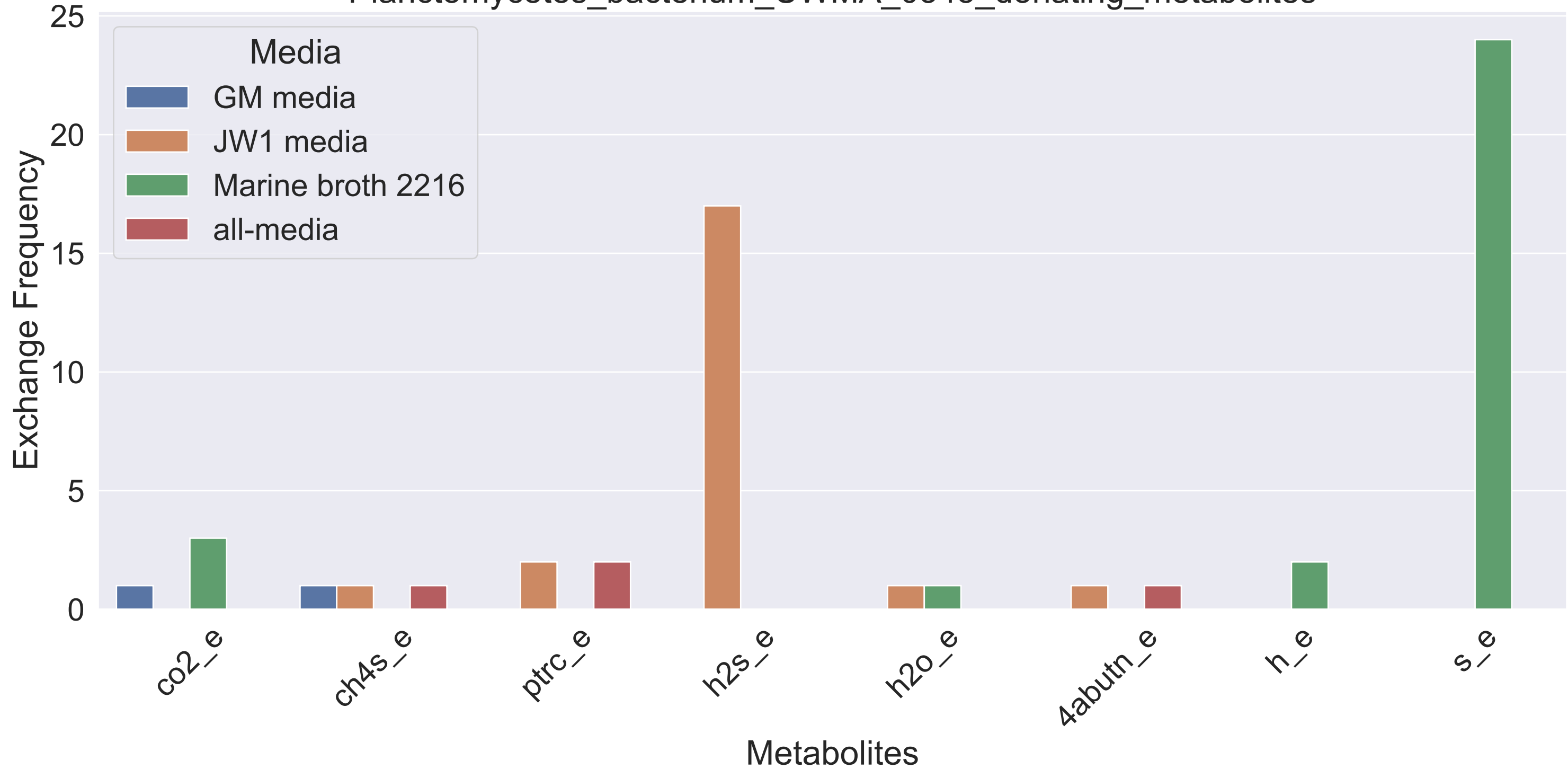

Supplement: Supplementary file 7 — Supplementary File S6 [file 43705_2023_242_MOESM7_ESM.zip › Supplementary Figure S188 Planctomycetes bacterium UWMA 0345 donating metabolites.pdf]

# Porticoccaceae\_bacterium\_UWMA\_0313\_donating\_metabolites

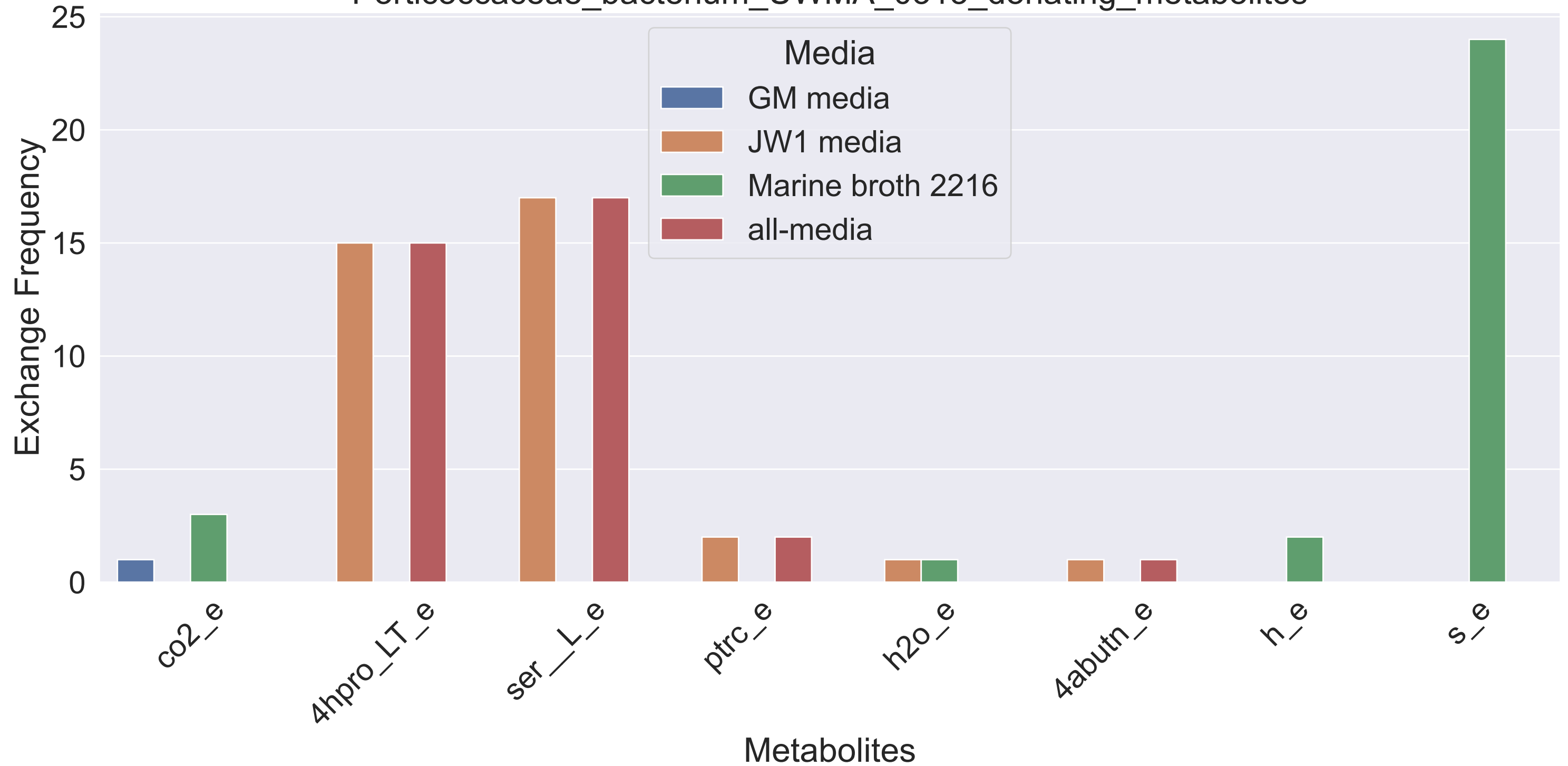

Supplement: Supplementary file 7 — Supplementary File S6 [file 43705_2023_242_MOESM7_ESM.zip › Supplementary Figure S189 Porticoccaceae bacterium UWMA 0313 donating metabolites.pdf]

Rhodospirillales\_bacterium\_UWMA\_0295\_donating\_metabolites

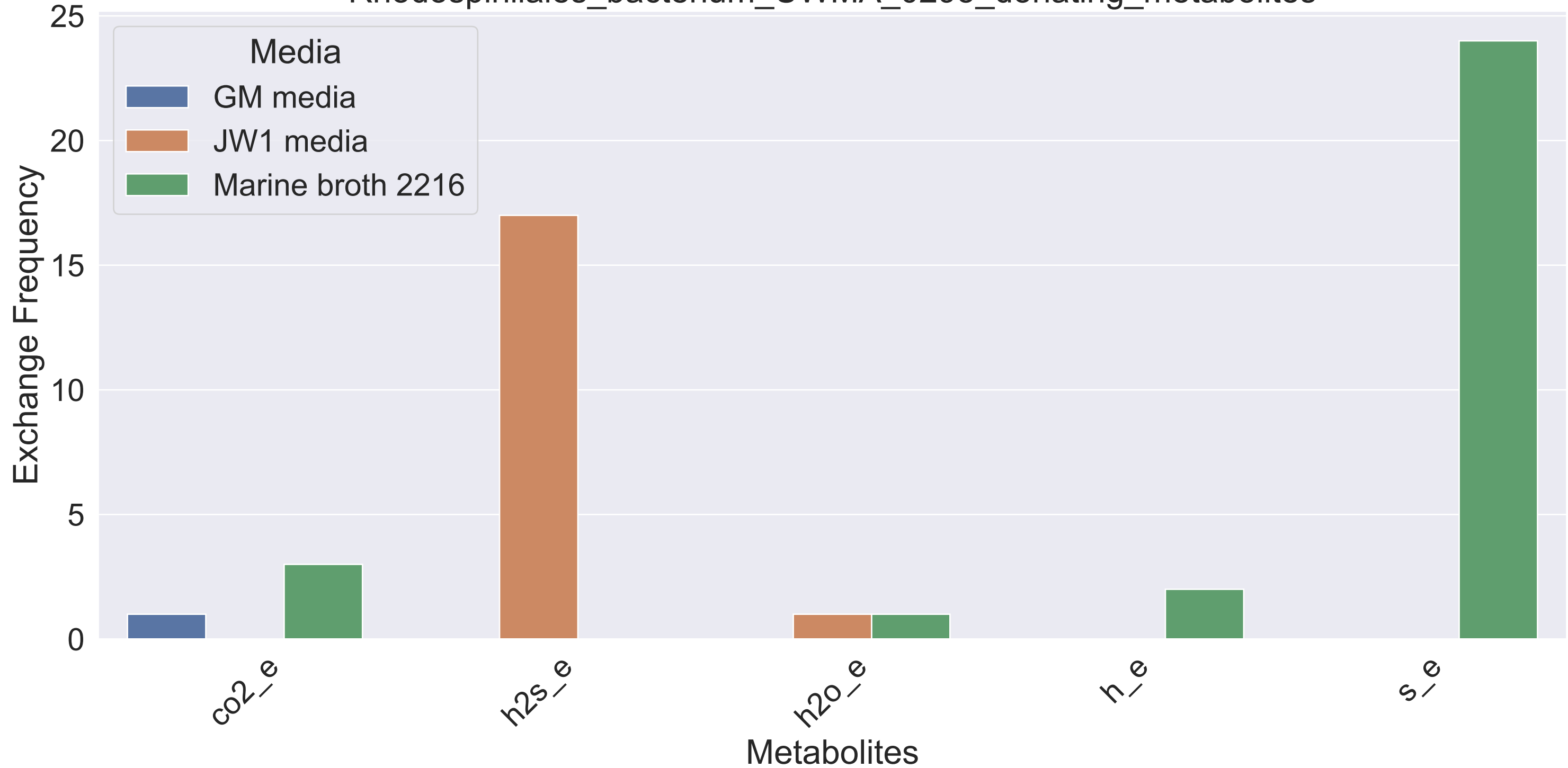

Supplement: Supplementary file 7 — Supplementary File S6 [file 43705_2023_242_MOESM7_ESM.zip › Supplementary Figure S190 Rhodospirillales bacterium UWMA 0295 donating metabolites.pdf]

# Sneathiellales\_bacterium\_UWMA\_0353\_donating\_metabolites

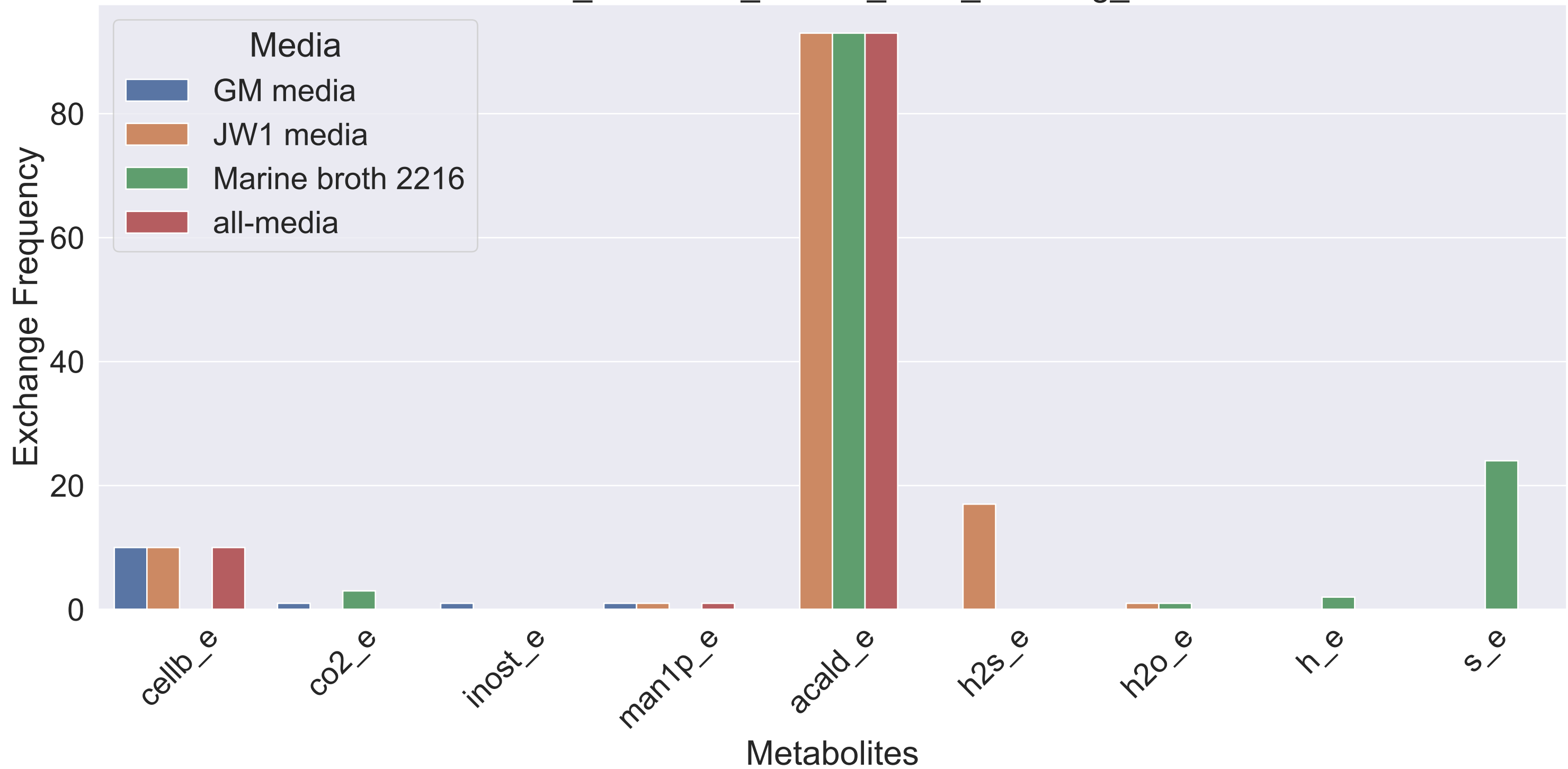

Supplement: Supplementary file 7 — Supplementary File S6 [file 43705_2023_242_MOESM7_ESM.zip › Supplementary Figure S191 Sneathiellales bacterium UWMA 0353 donating metabolites.pdf]

Sulfitobacter\_sp\_UWMA\_0305\_donating\_metabolites

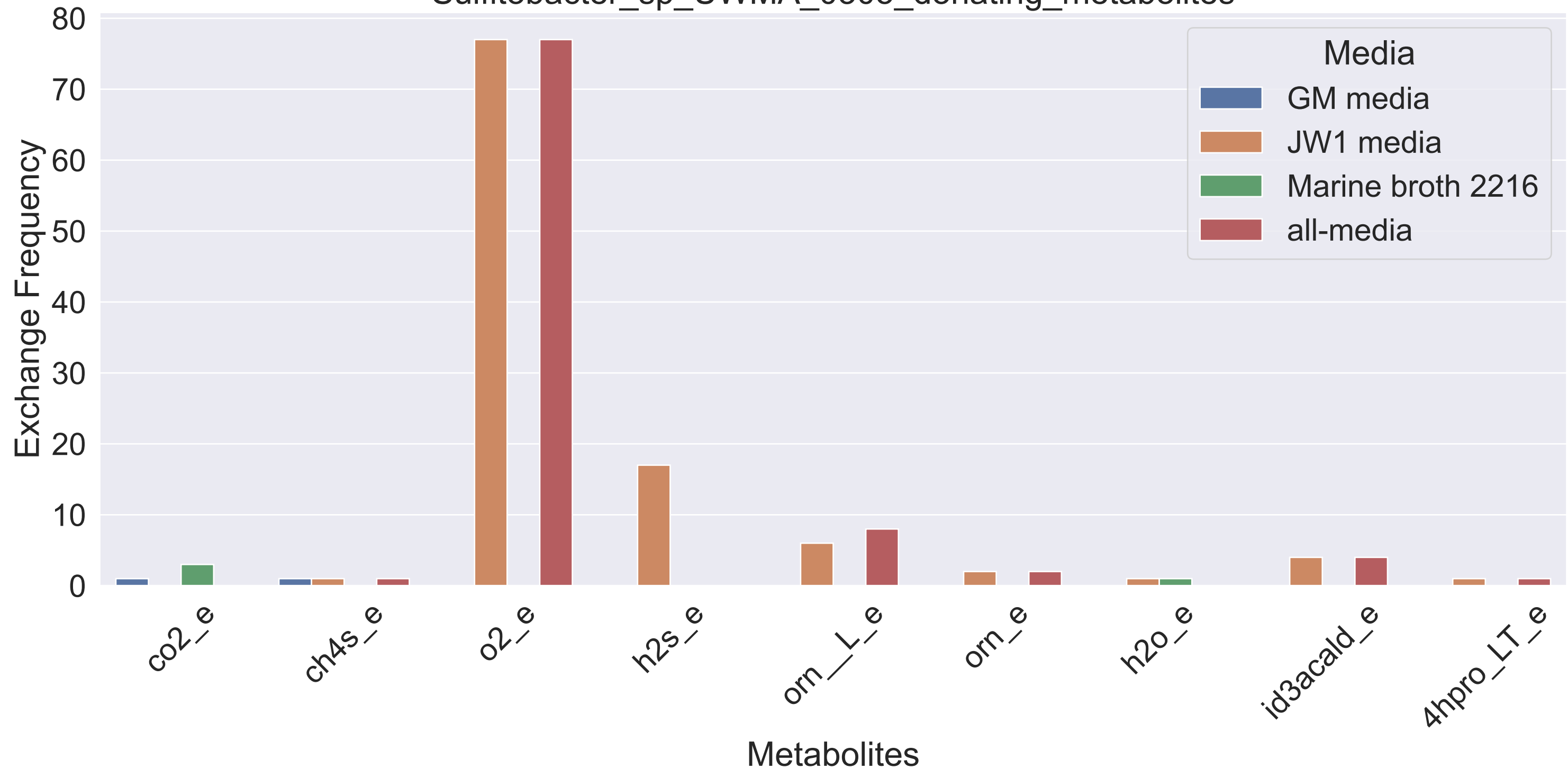

Supplement: Supplementary file 7 — Supplementary File S6 [file 43705_2023_242_MOESM7_ESM.zip › Supplementary Figure S192 Sulfitobacter sp UWMA 0305 donating metabolites.pdf]

Thiotrichaceae\_bacterium\_UWMA\_0311\_donating\_metabolites

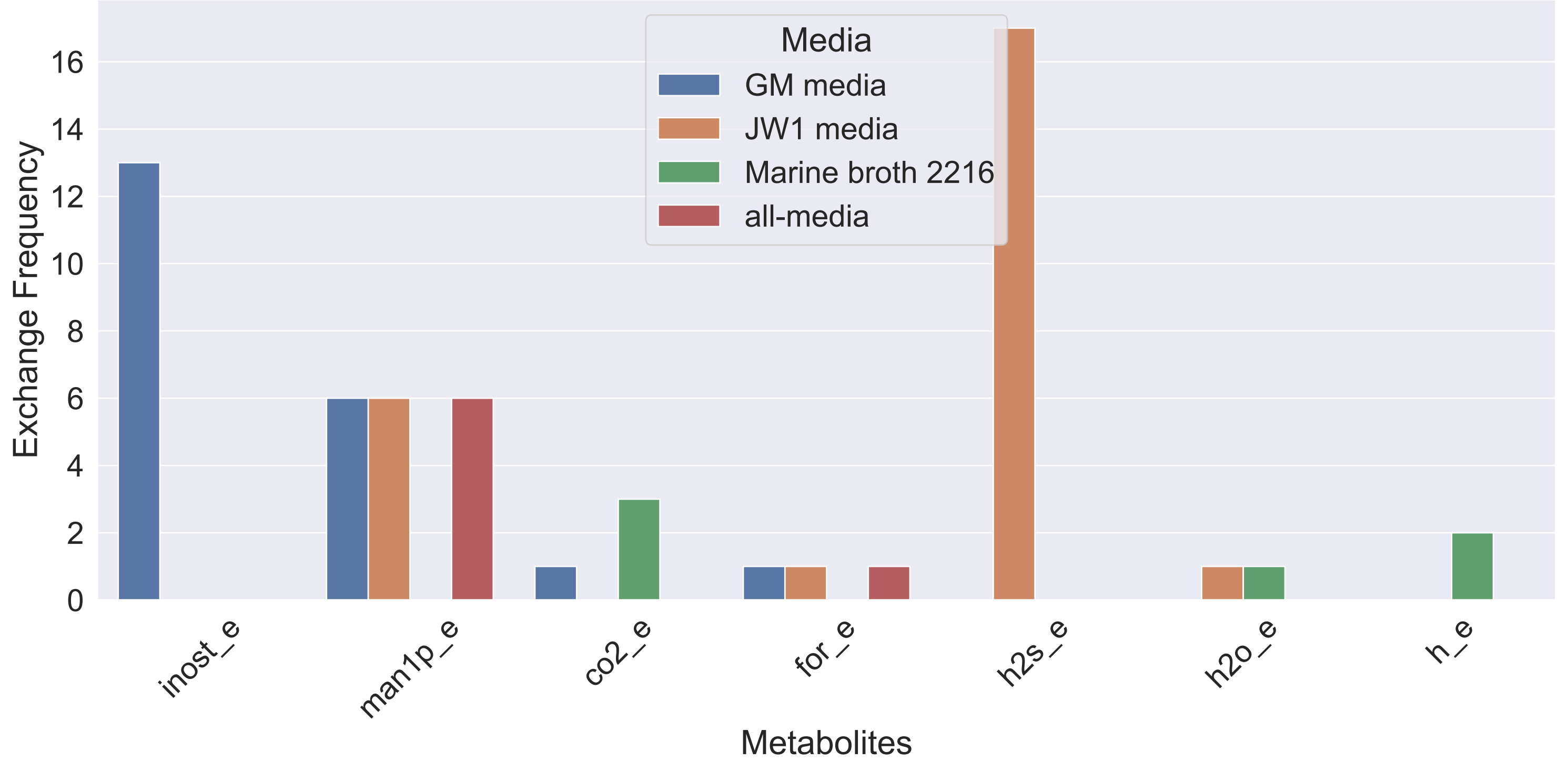

Supplement: Supplementary file 7 — Supplementary File S6 [file 43705_2023_242_MOESM7_ESM.zip › Supplementary Figure S193 Thiotrichaceae bacterium UWMA 0311 donating metabolites.pdf]

# Verrucomicrobiales\_bacterium\_UWMA\_0292\_donating\_metabolites

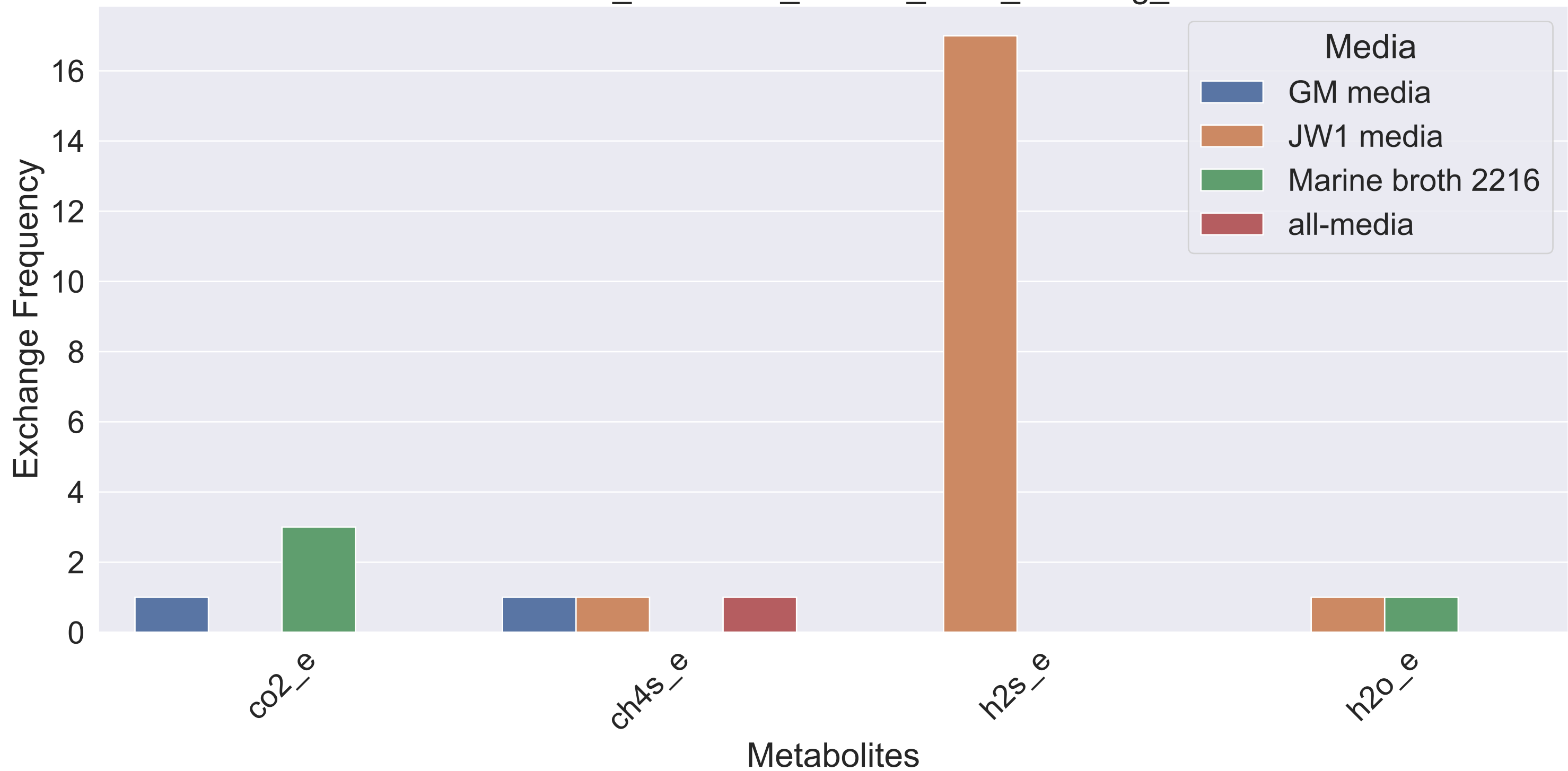

Supplement: Supplementary file 7 — Supplementary File S6 [file 43705_2023_242_MOESM7_ESM.zip › Supplementary Figure S194 Verrucomicrobiales bacterium UWMA 0292 donating metabolites.pdf]

Verrucomicrobiales\_bacterium\_UWMA\_0320\_donating\_metabolites

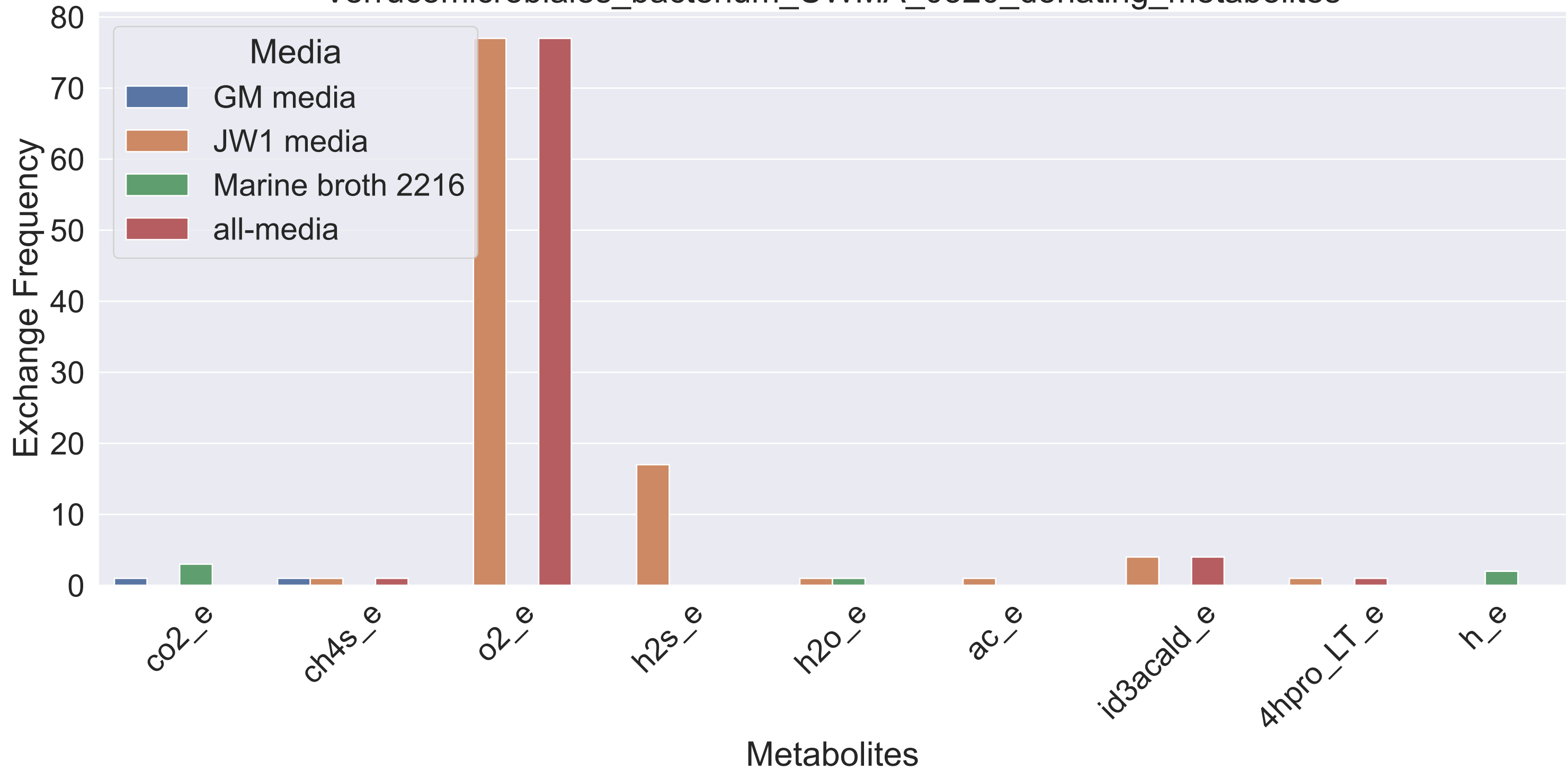

Supplement: Supplementary file 7 — Supplementary File S6 [file 43705_2023_242_MOESM7_ESM.zip › Supplementary Figure S195 Verrucomicrobiales bacterium UWMA 0320 donating metabolites.pdf]

# Verrucomicrobiales\_bacterium\_UWMA\_0332\_donating\_metabolites

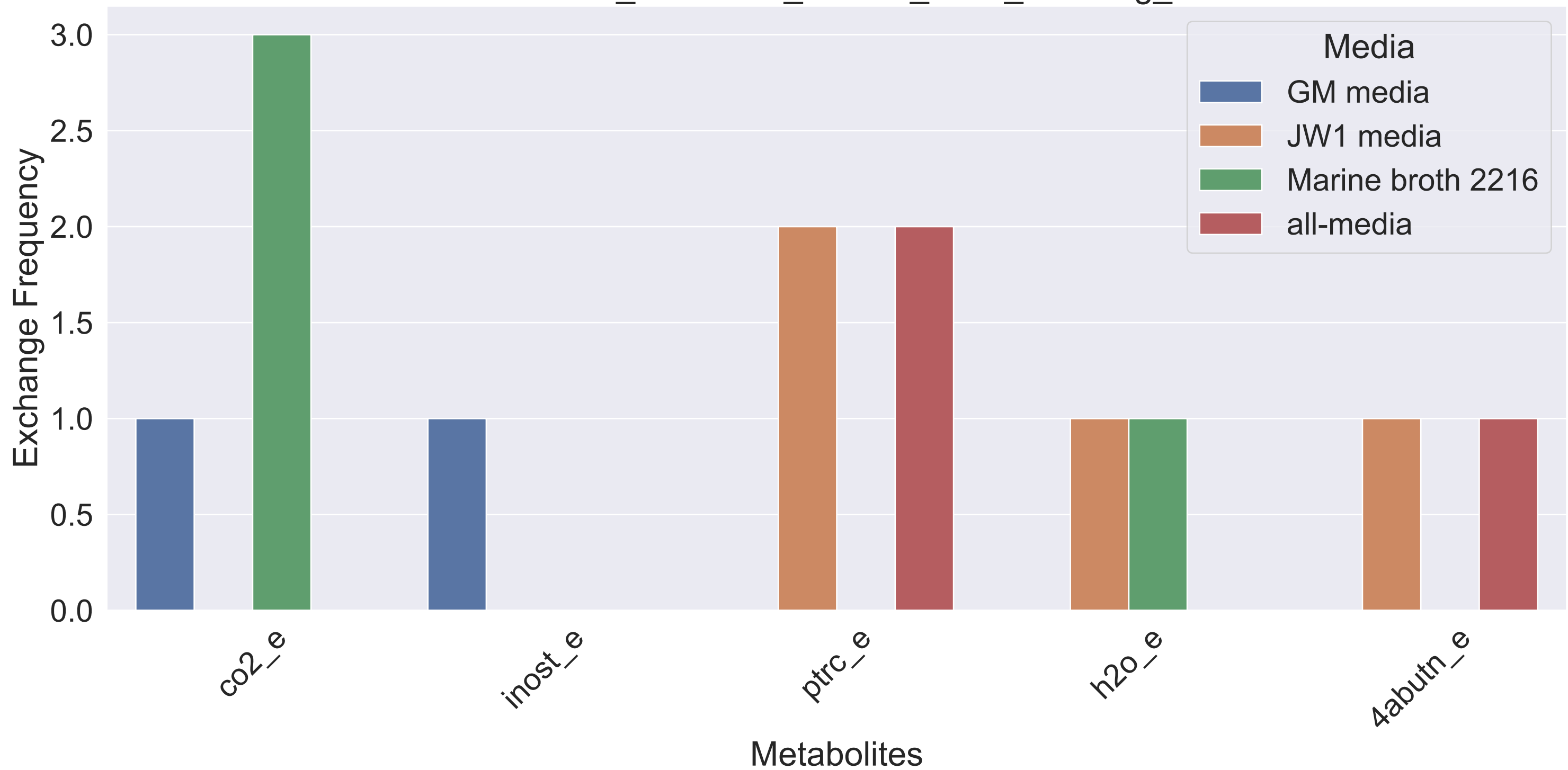

Supplement: Supplementary file 7 — Supplementary File S6 [file 43705_2023_242_MOESM7_ESM.zip › Supplementary Figure S196 Verrucomicrobiales bacterium UWMA 0332 donating metabolites.pdf]

# Acidimicrobiia\_bacterium\_UWMA\_0264\_donating\_metabolites

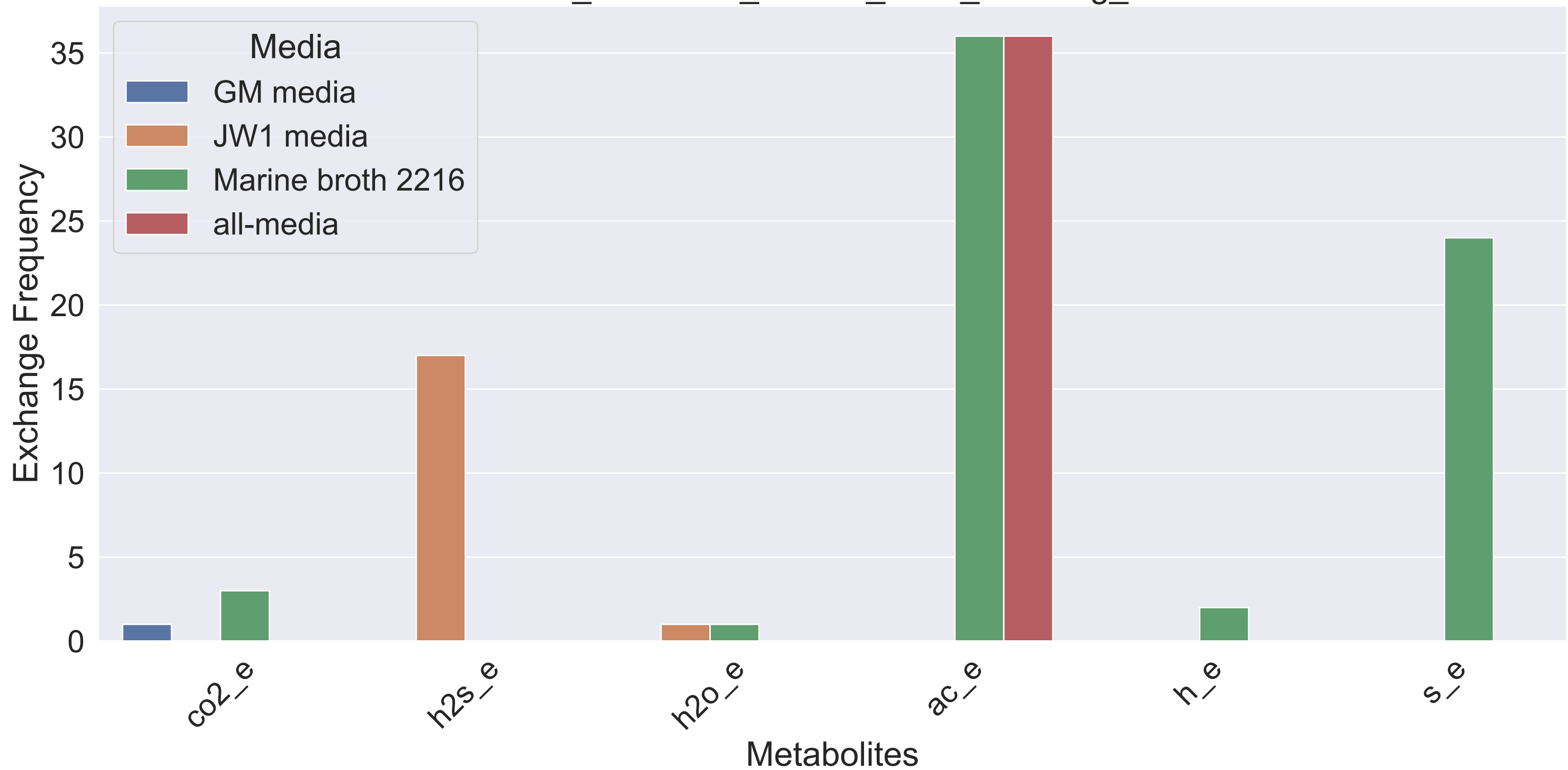

Supplement: Supplementary file 7 — Supplementary File S6 [file 43705_2023_242_MOESM7_ESM.zip › Supplementary Figure S99 Acidimicrobiia bacterium UWMA 0264 donating metabolites.pdf]
